# Endocytosis-mediated healing: recombinant human collagen type III chain-induced wound healing for scar-free recovery

**DOI:** 10.1093/rb/rbae149

**Published:** 2025-01-08

**Authors:** Jian Jin, Haihang Li, Zhengli Chen, Qingsong Liu, Jiqiu Chen, Zihan Tao, Xudong Hong, Yinjia Ding, Yue Zhou, Aifen Chen, Xudong Zhang, Kaiyang Lv, Liangliang Zhu, Shihui Zhu

**Affiliations:** State Key Laboratory of Molecular Engineering of Polymers, Department of Macromolecular Science, Fudan University, Shanghai 200438, China; Shanghai Depeac Biotechnology Co., Ltd, Shanghai 200444, China; Jiangsu Chuangjian Medical Technology Co., Ltd, Changzhou 213100, China; Department of Burn Surgery, Changhai Hospital, The Naval Medical University, Shanghai 200433, China; Department of Burn Surgery, Changhai Hospital, The Naval Medical University, Shanghai 200433, China; Department of Burn Surgery, Changhai Hospital, The Naval Medical University, Shanghai 200433, China; Department of Burn Surgery, Changhai Hospital, The Naval Medical University, Shanghai 200433, China; Department of Burns and Plastic Surgery, 903rd Hospital of PLA, Hangzhou 310012, China; Department of Burns and Plastic Surgery, 903rd Hospital of PLA, Hangzhou 310012, China; Department of Burns and Plastic Surgery, 903rd Hospital of PLA, Hangzhou 310012, China; Department of Burns and Plastic Surgery, 903rd Hospital of PLA, Hangzhou 310012, China; Department of Burns and Plastic Surgery, 903rd Hospital of PLA, Hangzhou 310012, China; Department of Plastic Surgery, Xinhua Hospital, Shanghai Jiao Tong University School of Medicine, Shanghai 200092, China; State Key Laboratory of Molecular Engineering of Polymers, Department of Macromolecular Science, Fudan University, Shanghai 200438, China; Department of Burns and Plastic Surgery, Shanghai Children's Medical Center, Shanghai Jiao Tong University School of Medicine, Shanghai 200127, China

**Keywords:** collagen, endocytosis, recombinant human collagen type III chain, scar formation

## Abstract

Scar formation can be effectively prevented when the proportion of collagen type I (Col I)/type III (Col III) is reduced. Unlike Col III, recombinant human collagen type III chain (RHC III chain) does not possess a triple helical structure. This study aimed to elucidate the capacity of fibroblasts to uptake RHC III chain, reduce the Col I/Col III ratio and determine its effects on wound healing and scar. RHC III chain demonstrates qualified cell compatibility. In cell experiments, immunofluorescence and western blot (WB) analyses revealed an increase in the polyhistidine tag level, indicating that RHC III chain in internalized by these cells. Transmission electron microscopy showed increased intracellular phagocytic activity, indicating that RHC III chain enters fibroblasts by endocytosis. The immunofluorescence and WB showed that Col III synthesis enhanced, and Col I/Col III ratio reduced. However, the polyhistidine tag disappeared with time, indicating that RHC III chain degraded within cells and then synthesized into Col III. The content of newly synthesized Col III increases, but real-time fluorescence quantitative showed a decrease in Col III related gene content suggests the formation of negative feedback. However, due to the sufficient raw materials, the amount of Col III synthesis is still increasing, leading to the reduction of the ratio of type I collagen/type III collagen, which beneficial to wound healing and reduce scar hyperplasia. In animal experiments, the SD rat full-thickness skin defect model of wound suggests that RHC III chain also takes effect through endocytosis and ultimately promotes wound healing. The rabbit ear scar model suggests that RHC III chain inhibits scar proliferation by reducing the ratio of Col I/Col III. In summary, RHC III chain was endocytosed by fibroblasts to promote native Col III synthesis, as well as promote wound healing and reduce scar hyperplasia.

## Graphical abstract

**Figure:**
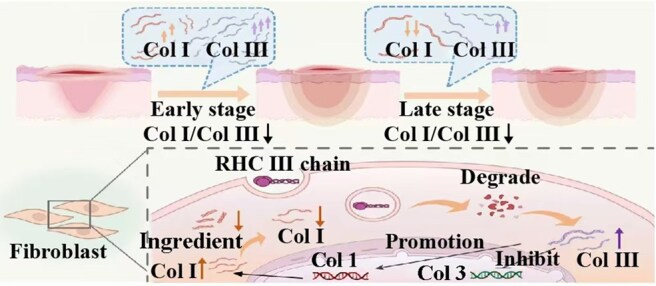

## Introduction

Wounds may lead to hypertrophic scars, affecting skin appearance and function and burdening the patients and society [1–3]. Regulating collagen secretion and arrangement during wound healing is vital for ameliorating scars. Increasing collagen type III (Col III) level or reducing collagen type I (Col I)/Col III ratio enhances scar appearance and softness [4–6]. Fetuses rarely develop pathological scars owing to higher levels of Col III in the skin [7]. Direct regulation of Col III without affecting Col I is challenging. Once Col I synthesis increases, it can lead to collagen deposition, further increasing the degree of scar hyperplasia [8–10].

**Figure 1.**
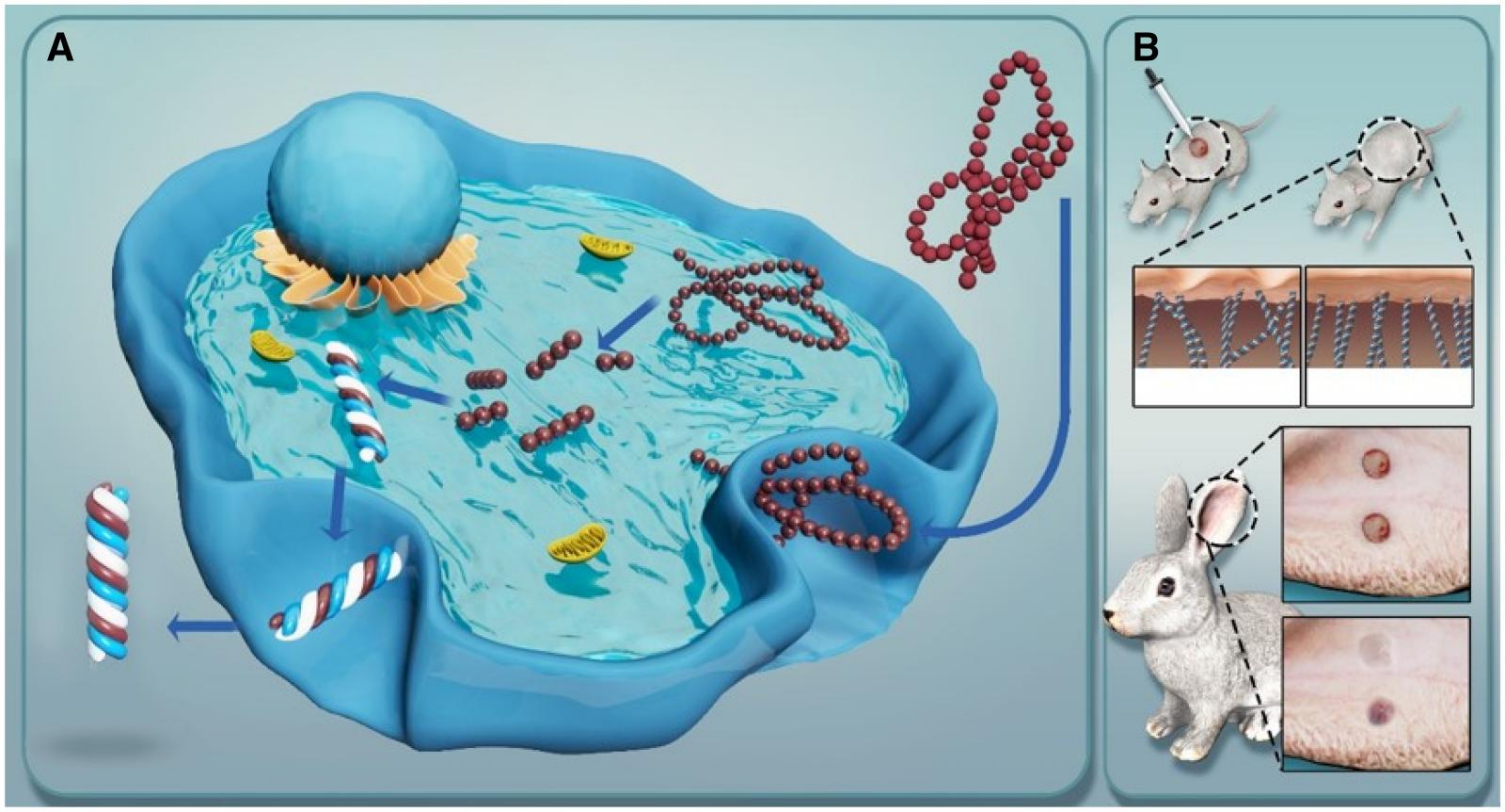
RHC III chain can be internalized by fibroblasts as a raw material for Col III synthesis (**A**), thereby promoting wound healing (**B**), improving healing quality (B) and even inducing scarless healing by promoting Col III synthesis. Col III, collagen type III; RHC III chain, recombinant human collagen type III chain.

With the advancement of gene recombination technology and fermentation processes, the homology and purity have been significantly improved, providing possibilities for clinical applications. Recombinant human collagen type III (RHC III chain) is named according to the industry standard YY/T 1888-2023 in China. RHC III chain is a single-stranded fragment of Col III synthesized using fermentation technology by introducing human Col III into yeasts (relevant patents can be referred to: Liang Liang, Jiangsu Chuangjian Medical Technology Co., Ltd, CN 103102407 B, 2014.04.30) [11]. Although RHC III chain lacks a triple helical structure, as observed in Col III, it possesses modified physicochemical properties and bioactivities (e.g. increased solubility), expanding its clinical applications. Early clinical studies have indicated that RHC III chain effectively improves wound healing and hypertrophic scars [12]. Based on previous research, we have proposed a corresponding scientific hypotheses: the mechanism of action involves RHC III chain being endocytosed by wound fibroblasts and serving as a precursor for Col III synthesis with a triple helical structure, ultimately enhancing wound healing and reducing hypertrophic scars (Figure 1).

In this study, we investigated RHC III chain endocytosis by fibroblasts, the subsequent effect of endocytosis on the activities of fibroblasts and the mechanism through which RHC III chain functions. We accomplished this by assessing the physicochemical properties of RHC III chain synthesized via fermenting genetically engineered yeasts and conducting cell-based experiments and using wound and scar models.

## Materials and methods

### Reagents and materials

Human skin fibroblasts (HSFs), human keratinocytes (HaCaTs) and human microvascular endothelial cells (HMVECs) were purchased from Qingqi (Shanghai) Biotechnology Development Co., Ltd (Shanghai, China). All cells were tested using the third to seventh generations. Anti-polyhistidine (His) tag antibody (ab213204), anti-collagen I antibody (ab138492), and anti-collagen III antibody (ab184993) were purchased from Abcam Trading Co., Ltd (Shanghai, China). Cell counting kit-8 (CCK-8) was purchased from Thermo Fisher Scientific Inc. (Shanghai, China).

### Physicochemical tests

We measured the solubility of RHC III chain and pH of 1 mg/ml RHC III chain; the molecular weight and purity were measured using high-performance liquid chromatography (Shimadzu i-Series LC-2060, Shimadzu Corporation, Japan) [13, 14]. In addition, we measured exogenous DNA content using quantitative polymerase chain reaction (PCR) and residual yeast protein level using an enzyme-linked immunosorbent assay (ELISA) kit. We detected characteristic peaks using Raman spectroscopy (ST-LM, Shandong Three Body Instrument Co., Ltd, China) [15]. We analyzed the amino acid sequences using an amino acid sequence analyzer (M31-L-3000, Oriental Glass (Beijing) Technology Co., Ltd, China). All tests are strictly carried out by the industry standard YY/T 1888-2023 in China.

### Cytocompatibility testing

Cytocompatibility testing was tested following the requirements of ISO10993. ISO10993-5 for Cytotoxicity, ISO10993-6 for intracutaneous irritation, ISO10993-10 for sensitization and ISO10993-11 for pyrogenicity. The cytotoxicity of RHC III chain was tested at concentrations of 0.1%, 0.5%, 1%, 2.5% and 5%. Supplementary Data S1 for specific operations.

### Cell experiments

#### Cell culture and grouping

HSFs were classified into control and experimental groups. The experimental group cells were treated with 1 mg/ml RHC III chain, whereas the control group cells were conventionally cultured. The culture environments for both groups were identical and cultivated with DMEM complete medium, 10% fetal bovine serum and dual antibodies at 37°C and 5% CO_2_.

#### Cell proliferation for HSFs

After 12, 24 h and 7 days of culture, cell proliferation was measured using CCK-8 and 5-ethynyl-2′-deoxyuridine (EdU) staining [16]. CCK-8 assay: Add 100 μl of HSF with 2 × 10³/μl to a 96-well plate. Cultivate for 12 and 24 h. Add 10 μ CCK-8 to each well and culture for 1 h. The absorbance of the samples was measured at a wavelength of 450 nm. The relative proliferation rates of the experimental and control group cells were compared. Relative proliferation rate (%) = (absorbance in the experimental group/absorbance in the control group) × 100 [2]. EdU staining: 10 μM EdU was added to the HSF cultivated for 12 and 24 h, and the mixture was further cultured for 2 h. Subsequently, the PBS with 3.7% formaldehyde was fixed at room temperature for 15 min, and 1 ml of 0.5% TritonX-100 room temperature membrane was permeated for 20 min. After 30 min of incubation in Apollo staining solution, the cells were washed and incubated in Hoechst 33342 staining solution for 30 min. Thereafter, five visual fields were randomly selected using a fluorescence microscope at 400-fold magnification to count the positively stained cells. The mean value of these counts was determined.

Endocytosis of RHC III chain for HSF. After 12, 24 h and 7 days of culture of HSF, cells were collected to measure the intracellular histidine (His) level of endocytosed RHC III chain using a transmission electron microscope (TEM) and western blotting, immunofluorescence assay of His [17]. TEM: Fix cells with 2.5% glutaraldehyde for 15 min and rinse. 1% citric acid and 0.1 M phosphate buffer (pH 7.2) were fixed at room temperature for 2 h. Rinse with 30%, 50%, 70%, 90%, 100% ethanol and 100% acetone for 20 min in sequence. A mixture of acetone and epoxy resin (1:1) was used to permeate the cells for 1 h, followed by a 1:3 mixture of acetone and epoxy resin for 3 h. Pure epoxy resin was then used to permeate for 12 h. After adding the embedding agent (epoxy resin), samples were polymerized at 60°C for 48 h. Ultra-thin sections were prepared and stained with a saturated aqueous solution of 2% uranium acetate and lead citrate at room temperature for 15 min before imaging the cell ultrastructure. Photos of the cell ultrastructure were taken. Immunofluorescence assay: Cells were fixed with 4% (w/v) paraformaldehyde for 15 min. To permeabilize the cells, 0.5% (v/v) Triton X-100 was added and allowed to penetrate at room temperature (approximately 20°C–25°C) for 15 min. Following this, 5% (v/v) normal serum was used to block non-specific binding at room temperature for 1 h. The primary antibody was added dropwise and incubated in a wet box at 4°C overnight. Afterward, a fluorescent secondary antibody was added dropwise and incubated at 37°C in a wet box for 1 h. For nuclear staining, 4′,6-diamidino-2-phenylindole (DAPI) was added dropwise and incubated in the dark for 5 min. The slides were then sealed with an anti-fluorescence quenching agent and observed under a fluorescence microscope. The target protein content can be evaluated based on the average optical density as follows: average optical density = total fluorescence intensity/target area, where the target area denotes the occupied area of cells (or tissues). Western blotting: The cells were lysed with lysis buffer at 4°C for 30 min, then centrifuged at 14 000 g for 15 min to obtain the supernatant. Protein concentration was determined using the bicinchoninic acid (BCA) method. Separation and concentration gels were prepared, and the samples were loaded with electrophoresis buffer before performing electrophoresis at a voltage of 100 V. After electrophoresis, the concentrated gel was removed, and the separation gel was marked, with the area measured. The gel was balanced in transfer solution before transferring the membrane at 260 mA for 2 h. The membrane was then placed in a sealing solution (TBST containing 5% (w/v) skim milk) and sealed at room temperature for 2 h. The primary antibody was diluted in the sealing solution and gently shaken at 4°C overnight. Following this, the membrane was incubated with the secondary antibody at room temperature for 2 h. The gray value was analyzed using ImageJ to determine the semi-quantitative His level. The ratio of the density of His bands to the density of GAPDH bands can be regarded as the relative concentration of His to evaluate its concentration.

The grouping was the same as HSFs in the endocytosis of RHC III chain for HaCaTs and HMVECs. After 12 and 24 h, the cells were acquired to measure intracellular His level of endocytosed RHC III chain using TEM and western blotting, immunofluorescence assay of His.

Col I and Col III levels and related gene expression. At 12, 24 h and 7 days after culture, Col 1 and Col 3-related gene expression levels were measured using real-time quantitative polymerase chain reaction (qPCR), whereas Col I and Col III levels were measured using western blotting [18, 19]. qPCR: Glyceraldehyde 3-phosphate dehydrogenase (GAPDH) was used as the control, and the primers were human-GAPDH-F (ATTCCACCCATGGCAAATTCC) and human-GAPDH-R (GACTCCACGACGTACTCAGC), hCOL1α1F (TTGCTTCCCAGATGTCCT) and hCOL1α1R (TGTCCCTTCATTCC), and hCOL3α1F (CTACTGGGCCTGGTGGTGA) and hCOL3α1R (GACCTGGTTCCCCAGGTTTT). The relative concentration was calculated [18]. Western blotting: The analysis was performed as described above.

### Wound healing assay

Thirty male Sprague–Dawley (SD) rats weighing 200 ± 20 g (laboratory animal production license number: SCXK [Shanghai] 2012-0003) were purchased from the experimental center of Second Military Medical University [2]. The rats were randomized into experimental and control groups, with 15 per group. A full-thickness skin defect wound of diameter 3 cm was made on the back of each rat [2]. A total of 1 ml of 1 mg/ml RHC III chain was injected into rats locally in the experimental group, whereas 1 ml of physiological saline was injected into rats in the control group. The dressing was changed once every 2 days. During the dressing change on Days 8, 16 and 24, the wound area was measured to calculate the wound-healing rate using the following formula: wound-healing rate (%) = [(initial wound area − measured wound area during dressing change)/initial wound area] × 100 [2].

Five rats from each group were randomly selected to collect wound tissues at 2 and 24 h after the dressing change on Day 8. The His tag level in the wound tissues was measured using immunofluorescence assay and western blotting [17, 18]. Endocytosis was observed using a transmission electron microscope. At 2 and 24 h after the dressing change on Day 8, Col 1 and Col 3-related gene expression levels were measured using qPCR. At 24 h after the dressing change on Day 8, Col I, Col III and total collagen levels and Col I/Col III ratio were measured using Sirius red-picric acid (SR-PC) staining and western blotting [19]. And evaluate the histological changes of the wound using HE staining. The dressing was changed in the remaining five rats in each group until the wounds healed, and the healing time was recorded. SR-PC staining: The tissues were fixed using formalin, conventionally dehydrated, embedded and sectioned. After drip staining with Sirius red staining solution for 1 h, the cell nuclei were stained with Mayer’s hematoxylin staining solution for 10 min and mounted. Thereafter, strong orange or bright red Col I and green Col III fibers were observed using a polarized light microscope. The remaining operations were performed as described above.

After the wound was completely healed, the fully healed and normal skin tissue was harvested and cut into dimensions 1 × 0.2 cm. One end one end of the long axis of each tissue sample was fixed, while tension was applied to the other. The amount of tension applied at the moment the tissue broke was recorded to evaluate the mechanical strength of the healed tissue.

### Hypertrophic scar induction

Five male New Zealand albino rabbits weighing 2750 ± 250 g were obtained from the same source as SD rats [2]. Two wounds of diameter 1 cm, which extended to the perichondrium, were made on each rabbit ear. The wound samples were classified into experimental and control groups, with 10 samples per group [20]. A total of 1 ml of 1 mg/ml RHC III chain was injected into rats in the experimental group, while 1 ml of physiological saline was injected into rats in the control group. The dressing was changed once every 3 days, and the healed wounds were left untreated. Ninety days after wound healing, the Vancouver Scar Scale (VSS) assessed the scars. His tag level was measured in the scar tissues using immunofluorescence assay and western blotting. Col 1 and Col 3-related gene expression levels were measured using qPCR. Col I, Col III, total collagen levels and Col I/Col III ratio were measured using SR-PC staining and western blotting. The operations were performed as described above [17–20].

### Ethics approval

This study was approved by the Laboratory Animal Management and Use Committee of Huizhi Yinghua Medical Technology R&D (Shanghai, China) Co., Ltd (approval number: SH2022-07024).

### Statistical analyses

Data are expressed as mean ± standard deviation. All data were statistically analyzed using SPSS (version 26.0., IBM Corp., Armonk, NY, USA). Except for scar hyperplasia-related data, the independent sample *t*-test was used for other data if they were normally distributed. Otherwise, the Wilcoxon signed rank-sum test was used. The data related to scar hyperplasia were analyzed using the self-control *t*-test if the data were normally distributed; otherwise, the Wilcoxon signed rank-sum test of paired samples was used. *P* < 0.05 indicated a statistically significant difference [2, 21].

## Results and discussion

### Physicochemical tests

The molecular weight of RHC III chain was 43 kDa. RHC III chain was readily soluble in purified water, acid and physiological saline. The pH of 1 mg/ml RHC III chain was 3.83 ± 0.06 in purified water and 4.27 ± 0.12 in physiological saline (*n* = 3), and the osmolarity of 1 mg/ml RHC III chain was 300.67 ± 5.51 mOsmol/kg (*n* = 3). Natural collagen has a triple helix structure, which makes it difficult to dissolve [21, 22]. RHC III chain does not have a triple helical structure and has a smaller molecular weight; therefore, it could be provided for clinical use in convenient dosage forms such as liquid dressings and local injections, which is conducive to expanding application scenarios.

The purity of RHC III chain was > 95% (*n* = 3). Quantitative PCR revealed the residual DNA content to be 7.97 ± 6.96 pg/mg (*n* = 3). The residual yeast protein level, measured using an ELISA kit, was 0.0250% ± 0.0058% (*n* = 3). These parameters indicate that the RHC III chain developed in this project can meet the requirements for raw material used as a clinical product (refer to YY/T 1888-2023) and will not be affected by the residual effects or risks of other components in the production process.

**Figure 2.**
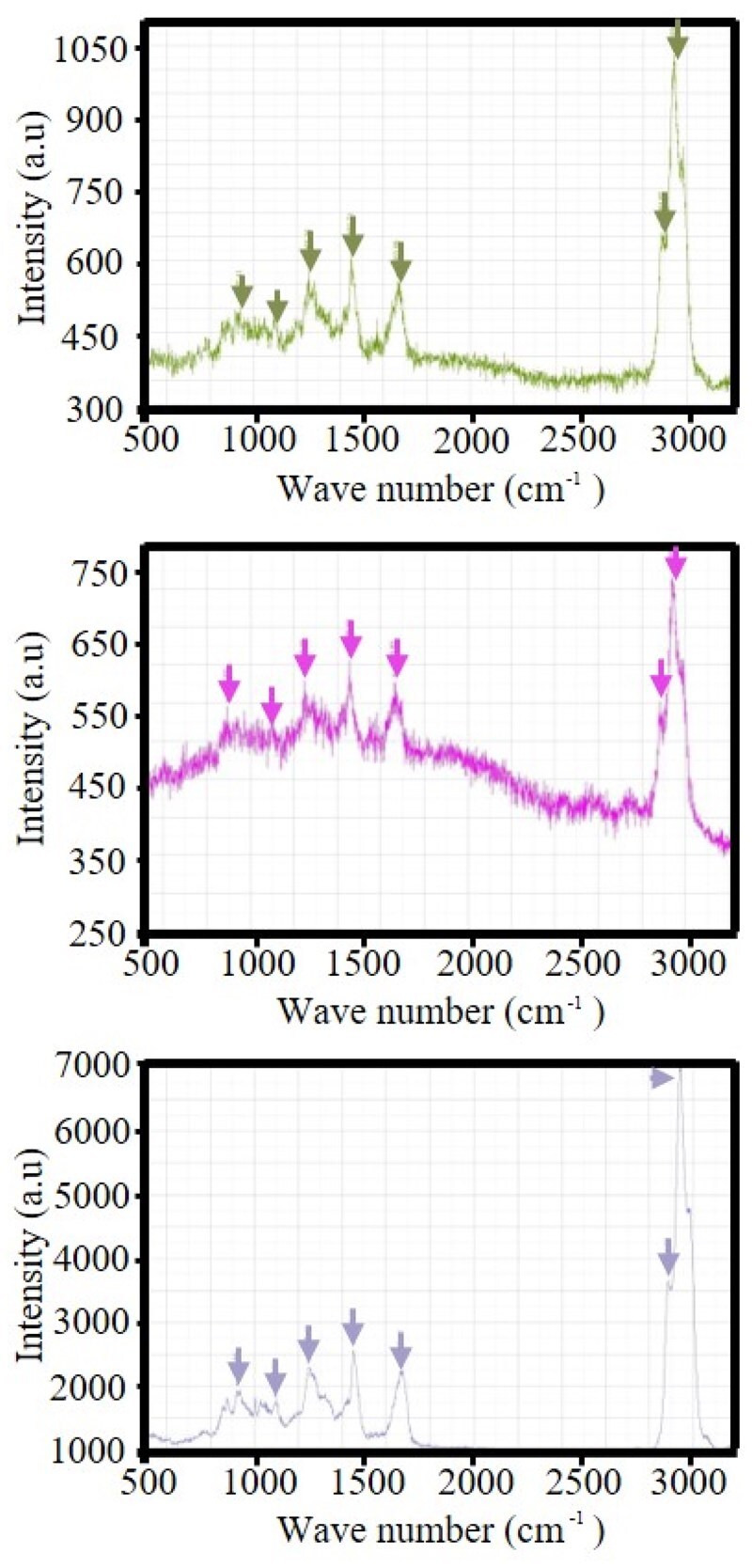
Raman spectroscopy. Three batches of RHC III chain displayed consistent characteristic peaks, indicating adequate batch stability. Among them, characteristic peaks were observed at 3500–3300 cm^−1^, 1700–1600 cm^−1^, 1600–1500 cm^−1^ for amide bonds, and at 1500–1400 cm^−1^, 1300–1200 cm^−1^ and 900–1000 cm^−1^. RHC III chain, recombinant human collagen type III chain.

The detected amino acid sequence (*n* = 3) was AGNTGAPGSPGVSGPKGDAGQPGEKGSPGAQGPPGAPGPLGIAGITGARGLAGPPGMPGPRGSPGPQGVKGESGKPGANGLSGERGPPGPQGLPGLAGTAGEPGRDGNPGSDGLPGRDGSPGGKGDRGENGSPGAPGAPGHPGPPGPVGPAGKSGDRGESGPAGPAGAPGPAGSRGAPGPQGPRGDKGETGERGAAGIKGHRGFPGNPGAPGSPGPAGQQGAIGSPGPAEFTAGNTGAPGSPGVSGPKGDAGOPGEKGSPGAQGPPGAPGPLGIAGTGARGLAGPPGMPGPRGSPGPQGVKGESGKPGANGLSGERGPPGPQGLPGLAGTAGEPGRDGNPGSDGLPGRDGSPGGKGDRGENGSPGAPGAPGHPGPPGPVGPAGKSGDRGESGPAGPAGAPGPAGSRGAPGPQGPRGDKGETGERGAAGIKGHRGFPGNPGAPGSPGPAGQQGAIGSPGPADHHHHHHTGLARF. Its homology to the amino acid sequence of the human collagen fragment was 100%. Raman spectroscopy showed that the characteristic spectral peaks (Figure 2) of multiple batches of samples were identical.

RHC III chain, a collagen peptide obtained using gene recombination and fermentation technologies, is highly soluble in water, which is beneficial for expanding its clinical application [11, 22]. Here, RHC III chain was produced using engineered yeasts. We found that (i) RHC III chain was soluble in water, acid and physiological saline; (ii) RHC III chain was highly pure, and the residual exogenous DNA and yeast protein levels were low; (iii) its parameters (e.g. pH and osmolality) complied with clinical-use requirements; and (4) A comparison of characteristic spectra obtained using Raman spectroscopy showed that the multiple characteristic spectra of RHC III chain produced via fermentation were identical, implying that RHC III chain could be fermented in a standardized manner. The above four points indicate that the RHC III chain developed in this study is feasible for clinical application. The analytical results indicated that the amino acid sequence of RHC III chain was identical to that of the corresponding fragment of Col III [11]. However, we failed to obtain Col III monomers and thus could not compare the characteristic spectra of Col III monomers and those of RHC III chain to further validate the homology between RHC III chain and Col III. Nonetheless, this also suggests that it has certain biological activity.

### Cytocompatibility

Under 0.1, 0.5, 1, 2.5 and 5 mg/ml RHC III chain treatment, individual cells were denatured only at the sample contact sites, yet they grew well, as determined using cytotoxicity assay. The cell viability for 0.1, 0.5, 1, 2.5 and 5 mg/ml RHC III chain were 99.59 ± 4.77, 99.33 ± 6.79, 99.64% ± 6.81%, 102.34 ± 6.36 and 100.11 ± 5.95, and all cytotoxicity were level 0 (*n* = 3), which complied with cytocompatibility requirements. Under 1 mg/ml RHC III chain treatment, at 24 h after intracutaneous irritation, the erythema and edema scores were 0 in the experimental group (*n* = 3), as determined using the intracutaneous irritation test. The results observed 48 and 72 h after intracutaneous irritation were comparable and negative (Supplementary Figure S2-1A). At 24 and 48 h after the testing, no positive results were observed in the experimental groups, and the sensitization testing results were negative (*n* = 3) (Supplementary Figure S2-1B). The pyrogenicity testing results indicated that the temperatures of rabbits increased by 0.35°C, 0.45°C and 0.35°C (*n* = 3), respectively, suggesting no pyrogenicity. Cytocompatibility is the foundation of clinical applications. The project tested in this study was selected based on ISO 10993 for wound treatment products. The RHC III chain developed in this study meets the requirements of cytocompatibility and can be applied in clinical practice [23–26]. It is also the foundation for subsequent research.

### Cell experiment

#### Cell proliferation

After 12, 24 h and 7 days of culture, the proliferation rates of HSFs (113.32% ± 7.17%, 118.71% ± 8.30% and 119.22 ± 7.87, *n* = 5) determined using the CCK-8 assay in the experimental group were greater than those in the control group (*P* = 0.003 and <0.001). The EdU assay results suggested that the positive rate for HSFs in the experimental group was greater than that in the control group (*n* = 5, *P* < 0.001 for 12, 24 h and 7 days respectively) (Supplementary Figure S2-2). The results indicate that it had the activity of promoting HSF proliferation under *in vitro* conditions, which is consistent with previous research results and clinical manifestations [27, 28].

#### Endocytosis of RHC III chain

**Figure 3.**
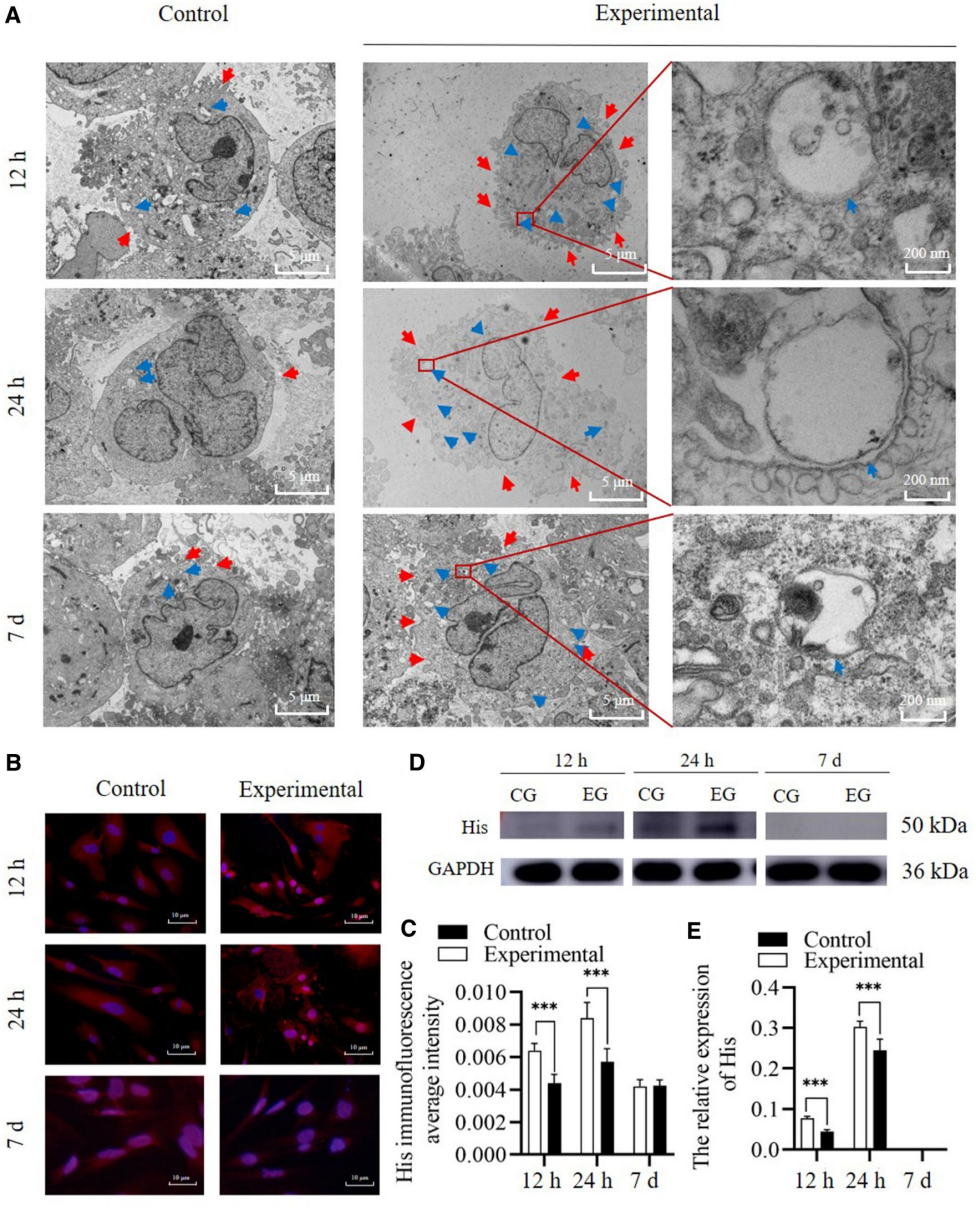
Endocytosis of RHC III chain in a cell-based experiment. (**A**) Scanning electron microscopy of endocytosis after HSFs was co-cultured with RHC III chain. The experimental group exhibited an increase in endocytic vesicles, whereas no significant endocytosis was observed in the control group. (**B**, **C**) The mean optical density of intracellular His was assessed using an immunofluorescence assay. (**D**, **E**) WB (CG, control group; EG, experimental group) assessing His content. *Significant differences (*P *<* *0.05). **Significant differences (*P *<* *0.01). ***Significant differences (*P *<* *0.001). GAPDH, glyceraldehyde 3-phosphate dehydrogenase; HSF, human skin fibroblasts; RHC III chain, recombinant human collagen type III chain.

At 12, 24 h and 7 days after co-culture, TEM showed that the endocytosis activity of HSFs increased, and numerous endocytic vesicles were intracellularly observed in the experimental group. However, on Day 7, a relative decrease was observed. TEM cannot directly determine the endocytic content of Col III or RHC III chain [29]; it can only indicate that the endocytic activity of HSF is enhanced due to the addition of RHC III chain. Therefore, the His tag is further used to evaluate whether RHC III chain enters cells. The immunofluorescence assay results indicated that the mean optical density of intracellular His in the experimental group of 12 and 24 h were higher than that in the control group (*n* = 5, *P* < 0.001 and = 0.001). Nevertheless, no significant difference was observed on Day 7. The results of western blot (WB) analysis of His level were consistent with those of the immunofluorescence assay; a significant difference was observed at 12 and 24 h (*n* = 5, *P* < 0.001 and =0.003); however, it was not detected in both the experimental and control groups on the seventh day (Figure 3). The His tag, while absent in native proteins, is a characteristic of recombinant proteins and can, therefore, be used to differentiate between RHC III chain and native collagen [30, 31]. The immunofluorescence assay and western blotting showed increased levels of His in HSFs in the experimental group at 12 and 24 h, indicating the entry of RHC III chain into HSFs in an intact form. Immunofluorescence also localized to the cytoplasm, indicating that RHC III chain mainly exists in the cytoplasm after entering the cell. However, at 7 days, the His tag was not detected in the experimental group, indicating that RHC III chain degraded after HSF's internalization, resulting in the disappearance of the His tag.

No significant endocytosis was observed in HaCaT and HMVEC under transmission electron microscopy (Supplementary Figure S2-3A). Moreover, the intensity of His immunofluorescence in HaCaT and HMVEC cells cultured with RHC III chain did not significantly increase (Supplementary Figure S2-3B and C). WB also did not detect the presence of His tags inside the cells.

Overall, the findings suggest that RHC III chain may enter the cell through endocytosis. However, it demonstrated cell selectivity. Fibroblasts, the main secreting cells of collagen, are important mediators of RHC III chain bioavailability. However, neither HaCaT nor HMVEC has the activity of endocytosis of RHC III chain. Hence, we speculate that this endocytosis is specific, but further research is needed to confirm it.

**Figure 4.**
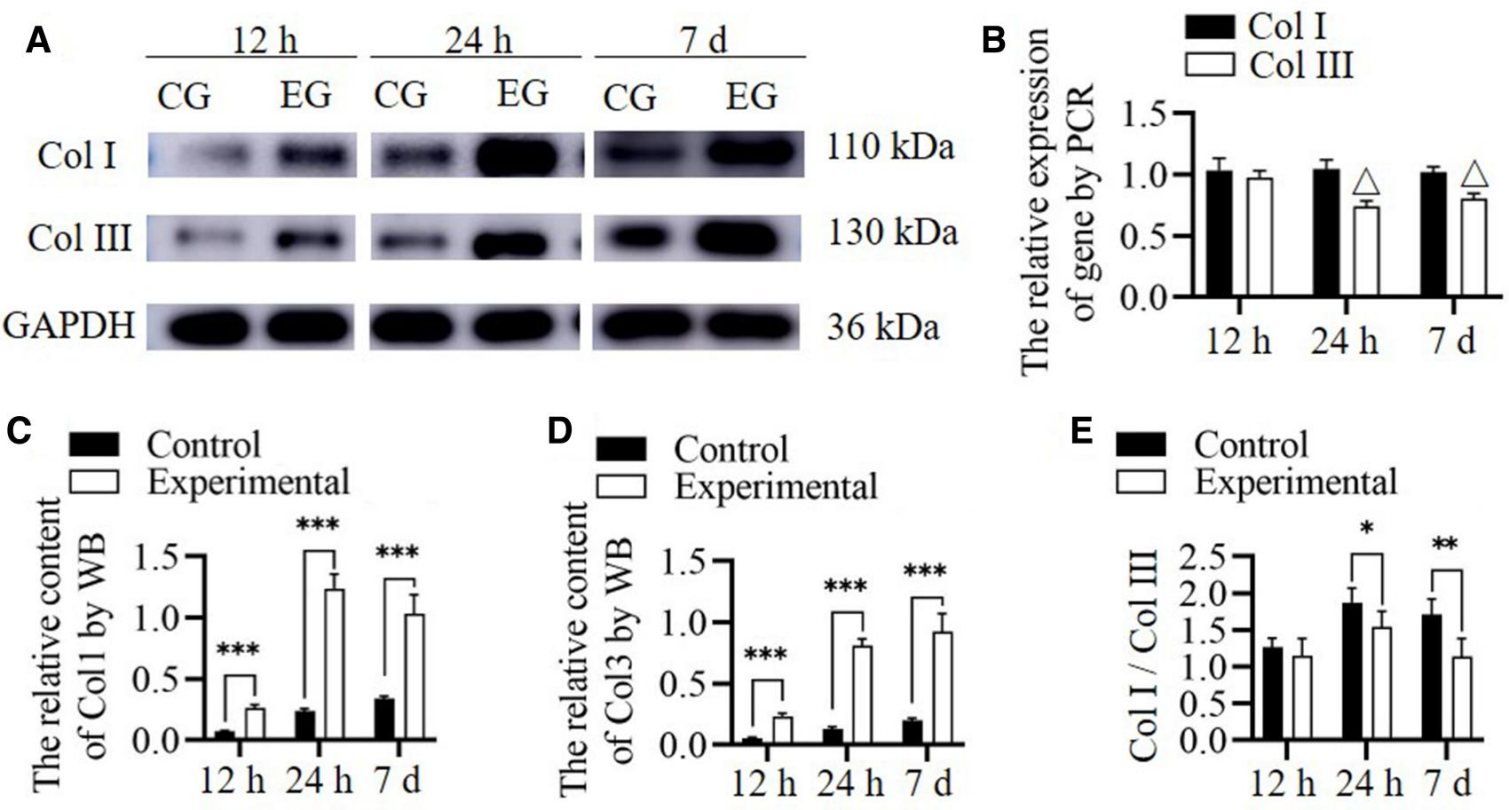
Collagen content and expression of collagen synthesis related genes in cell experiment. (**A**) WB (CG, control group; EG, experimental group) assessing Col I and Col III content. (**B**) Expression of synthetic genes for Col I and Col III. (**C**–**E**) WB (CG, control group; EG, experimental group) assessing Col I content (C), Col III content (D) and Col I/Col III (E). *Significant differences (*P *<* *0.05). **Significant differences (*P *<* *0.01). ***Significant differences (*P *<* *0.001). △Presence of significant differences compared to the control group (*P *<* *0.001). GAPDH, glyceraldehyde 3-phosphate dehydrogenase; Col I, collagen type I; Col III, collagen type III; HSF, human skin fibroblasts; RHC III chain, recombinant human collagen type III chain.

Col I and Col III expression secretion and Col I/Col III ratio. At 12 h after culture, the PCR analysis showed that the content of the genes related to the synthesis of Col 1 and Col 3 in HSFs were not significantly different between the experimental and control groups (*n* = 5, *P* = 0.453 and = 0.364, respectively). At 24 h and 7 days after culture, the PCR analysis showed that the genes related to the synthesis of Col 1 were not significantly different between the experimental and control groups (*n* = 5, *P* = 0.250 for 24 h, and = 0.350 for 7 days). However, the genes related to the synthesis of Col 3 in the experimental group were lower than those in the control group (*n* = 5, *P* < 0.001, respectively). At 12 and 24 h and 7 days, the WB analysis results showed that the content of Col I and Col III in the experimental group was higher than in the control group (*n* = 5, *P* < 0.001 for all). At 24 h and 7 days, the Col I/Col III ratio was notably lower in the experimental group than in the control group (*n* = 5, *P* = 0.035 and = 0.004, respectively). The Col I/Col III ratio in the control group increased at 24 h and 7 days compared with that at 12 h (*n* = 5, *P* < 0.001 and = 0.002 respectively) (Figure 4). The results suggest that the DNA content related to the synthesis of Col I and Col III has a different trend than the synthesis amount of Col I and Col III. The DNA content related to the synthesis of Col 1 increased, although no significant difference was observed, which may be due to a decrease in the compensatory Col I/Col III ratio. Col I synthesis confirms this hypothesis; however, compensation does not significantly change the Col I/Col III ratio. For Col 3I, the DNA content related to the synthesis of Col 3 remained unchanged in the early stages but decreased with time because of the negative feedback caused by increased Col III content. This finding was contrary to the continuously increased synthesis of Col III, which was possible because endocytosed RHC III chain was degraded into raw material for Col III synthesis. Although the DNA content was inhibited via the feedback of the newly synthesized Col 3, its post-transcription utilization rate increased because of adequate levels of raw materials and intermediate products, resulting in generally increased Col III synthesis. The ratio of Col I/Col III in the control group significantly increased over time, which is also an important reason for Col I deposition, leading to scar hyperplasia in wounds without effective treatment intervention [32, 33].

In addition, after replacing the culture medium for re-cultivation, no His label was detected in both the culture medium and cells. The newly synthesized Col III does not have a His tag, suggesting that RHC III chain is not used as a raw material for Col III synthesis in a single-stranded form for triple helix but is degraded and then used as a raw material for Col III synthesis.

### Wound healing

The wound healing rate was markedly greater (*n* = 15, *P* < 0.001 for 8 days; *n* = 5, *P* < 0.001 for 16 days; *n* = 5, *P* = 0.001 for 24 days) and healing time (22.40 ± 1.67 days vs 27.60 ± 1.67 days, *n* = 5, *P* = 0.001) was shorter in the experimental group than in the control, reminder that RHC III chain promotes wound healing. Although SD rats, which are rodents, were employed, pathological hypertrophic scars were not formed in the wound model [20]; however, the scar area formed in the experimental group was smaller than that in the control group. Elongation detection during break showed that normal skin had the highest breaking strength (48.08 ± 1.58N). The breaking strength of the experimental group (42.16 ± 1.12N) was lower than that of normal skin (*n* = 5, *P* = 0.016) but significantly higher than that of the control group (36.72 ± 1.09N) (*n* = 5, *P* = 0.008).

There was no significant difference in HE staining. Collagen deposition is an important cause of scar hyperplasia. Masson's test results theoretically suggest that the experimental group has more obvious scar hyperplasia, but the collagen type also influences the relationship between increased collagen content and scar hyperplasia. This was validated in subsequent content and proportion testing for Col I and Col III. At 24 h after the local injection of RHC III chain, SR-PC staining showed that the Col 1 level was not significantly different between the experimental and control groups (*n* = 5, *P* = 0.378), but the Col 3 level increased considerably and was significantly higher in the experimental group than in the control group (*n* = 5, *P* < 0.001), and a significant decrease in Col I/Col III was observed (*n* = 5, *P* < 0.001). The western blotting results also showed an increase in Col III secretion (*n* = 5, *P* < 0.001) and a significant decrease in the Col I/Col III ratio (*n* = 5, *P* < 0.001). Hence, the increase in total collagen content, but the more significant increase in Col III, is an important reason for the significant improvement in scar hyperplasia.

**Figure 5.**
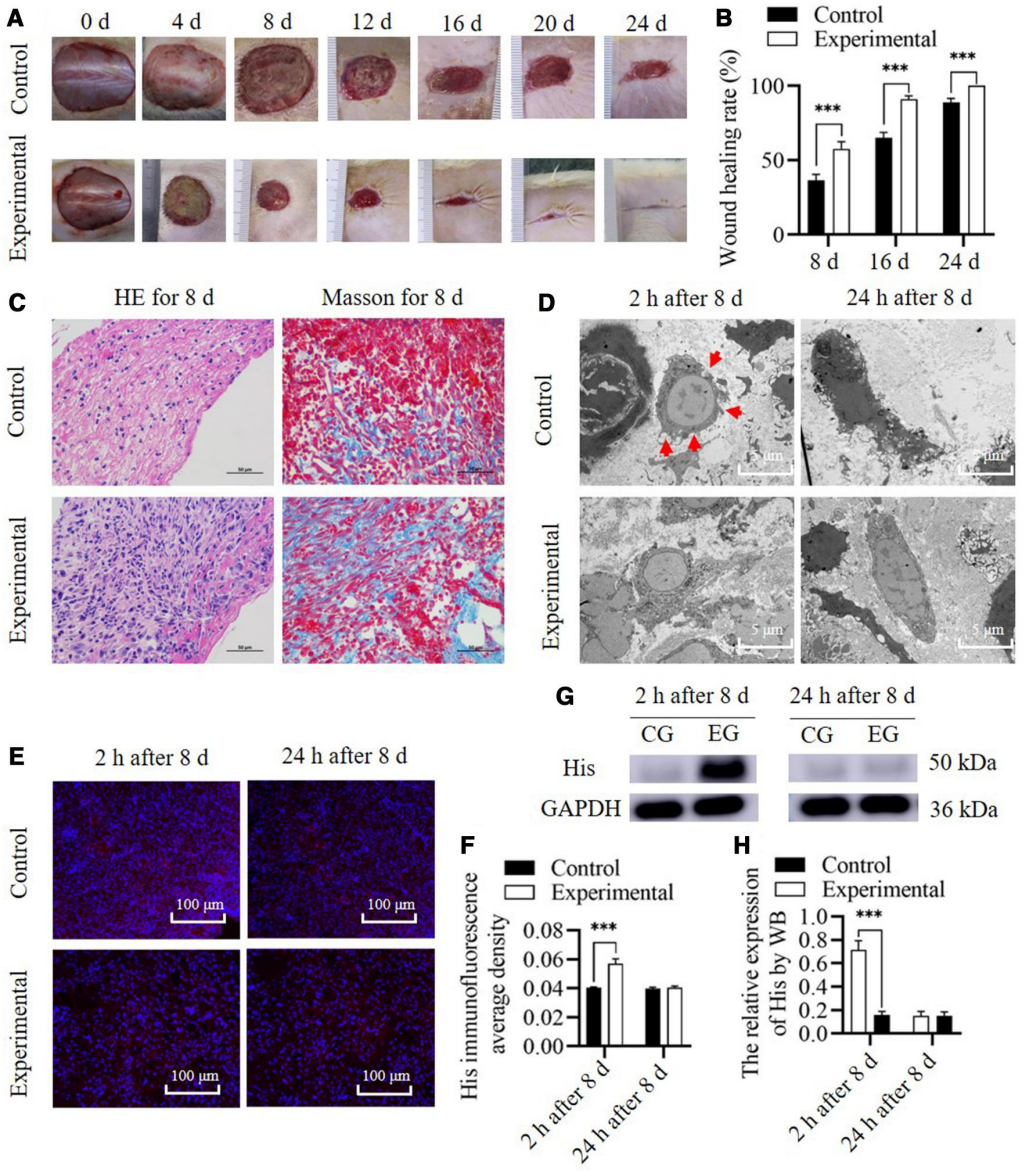
Wound model in animal experiment. (**A**, **B**) Wound healing rate. (**C**) HE and Masson staining. (**D**) Bio-TEM of tissues. Enhanced endocytosis was observed early (2 h) after local injection of RHC III chain (arrow indicates ongoing endocytosis). (**E**, **F**) Mean optical density of His was assessed using immunofluorescence assay at 2 and 24 h after 8 days. (**G**, **H**) WB (CG, control group; EG, experimental group) assessing the levels of His content at 2 and 24 h after 8 days. ***Significant differences (*P *<* *0.001). GAPDH, glyceraldehyde 3-phosphate dehydrogenase; RHC III chain, recombinant human collagen type III chain; His, histidine.

At 2 h after the local injection of RHC III chain, increased endocytosis activity was observed via a transmission electron microscope in the experimental group. His immunofluorescence assay and western blotting results demonstrated that His level was higher in the experimental group than in the control group (*n* = 5, *P* < 0.001 for both immunofluorescence and western blotting). At 24 h after the local injection of RHC III chain, no notably increased endocytosis activity was observed in the experimental and control groups, and the His levels were not significantly different between the experimental and control groups, as determined using the immunofluorescence assay and western blotting (*n* = 5, *P* = 0.23 and =0.996 respectively). Based on cellular experiments, *in vivo*, animal experiments further confirmed that RHC III chain is internalized into the cytoplasm and degraded, and as a result, a series of biological activities are generated. The newly synthesized Col III did not contain a His tag, suggesting that intact RHC III chain was not the raw material for synthesizing Col III (Figure 5).

**Figure 6.**
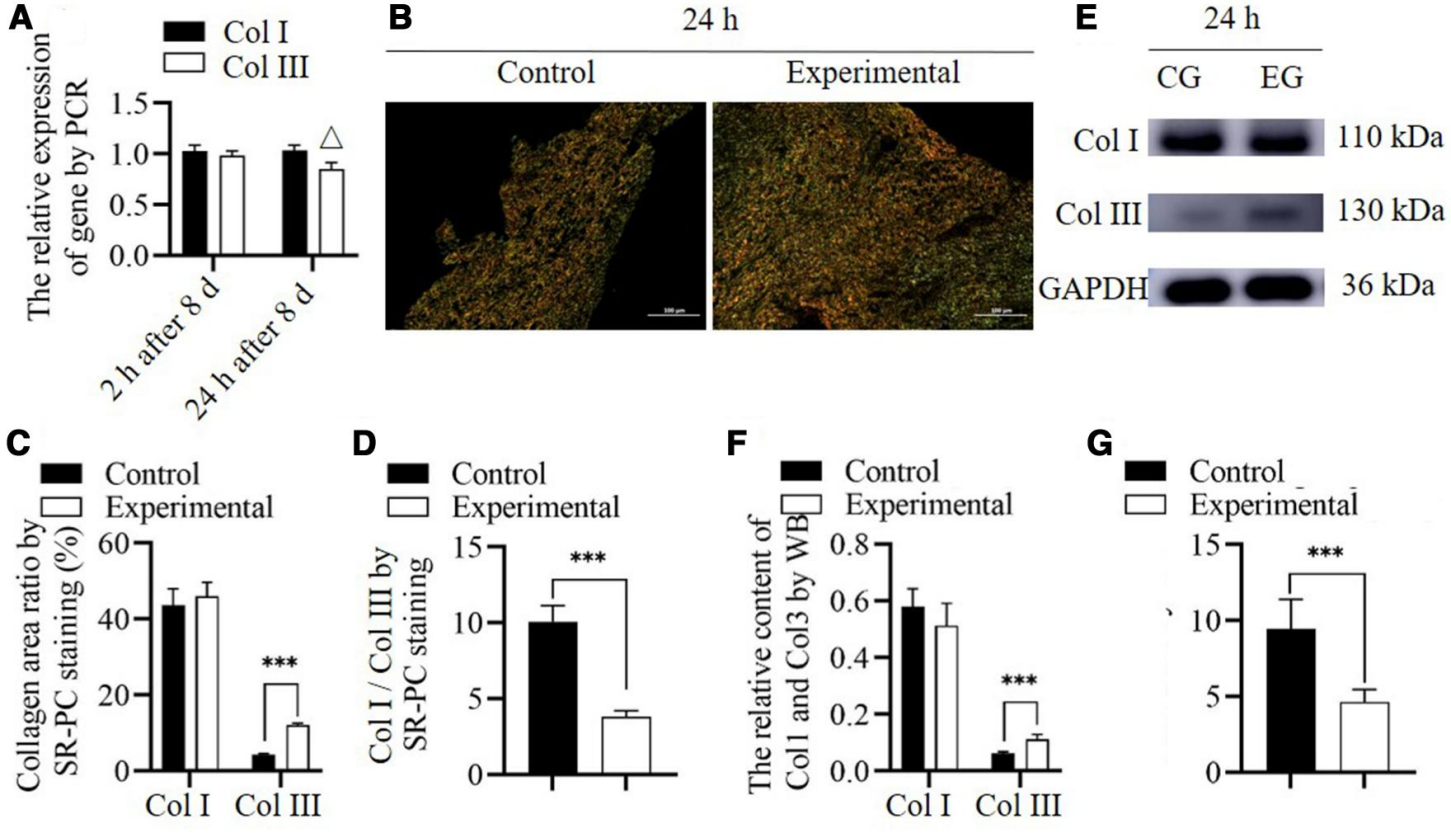
Collagen content and expression of collagen synthesis related genes in animal experiment. (**A**) Expression of synthetic genes for Col 1 and Col 3. (**B**–**D**) Sirius red-picric acid staining assessing the levels of Col I and III (C), and Col I/Col III content ratio (D) at 24 h after the local injection of RHC III chain at 8 days. (**E**–**G**) WB (CG, control group; EG, experimental group) assessing the levels of Col I and Col III content (F), and Col I/Col III content ratio (G). ***Significant differences (*P *<* *0.001). △Significant differences compared to the control group (*P *<* *0.001). GAPDH, glyceraldehyde 3-phosphate dehydrogenase; Col I, collagen type I; Col III, collagen type III; RHC III chain, recombinant human collagen type III chain.

At 2 h after culture, the PCR analysis showed that the relative expression levels of Col 1 and Col 3 were not significantly different between the experimental and control groups (*n* = 5, *P* = 0.166 and =0.219, respectively). At 24 h after culture, the PCR analysis showed that the relative expression level of Col 1 was not significantly different between the experimental and control groups (*n* = 5, *P* = 0.104), but Col 3 expression in the experimental group was lower than that in the control group (*n* = 5, *P* < 0.001). The expression levels of Col 1 and Col 3 were consistent with the results of cell experiments. At 24 h after 8 D, SR-PC and WB both showed no significant change in Col I, but a significant increase in Col III content (*n* = 5, *P* < 0.001 for both SR-PC and western blotting), leading to a significant decrease in the Col I/Col III ratio (*n* = 5, *P* < 0.001 for both SR-PC and western blotting). But it also suggests that at the level of gene expression, Col III is suppressed. However, due to having sufficient raw materials, the synthesis of Col III shows an increasing effect (Figure 6).

### Hyperplastic scars

**Figure 7.**
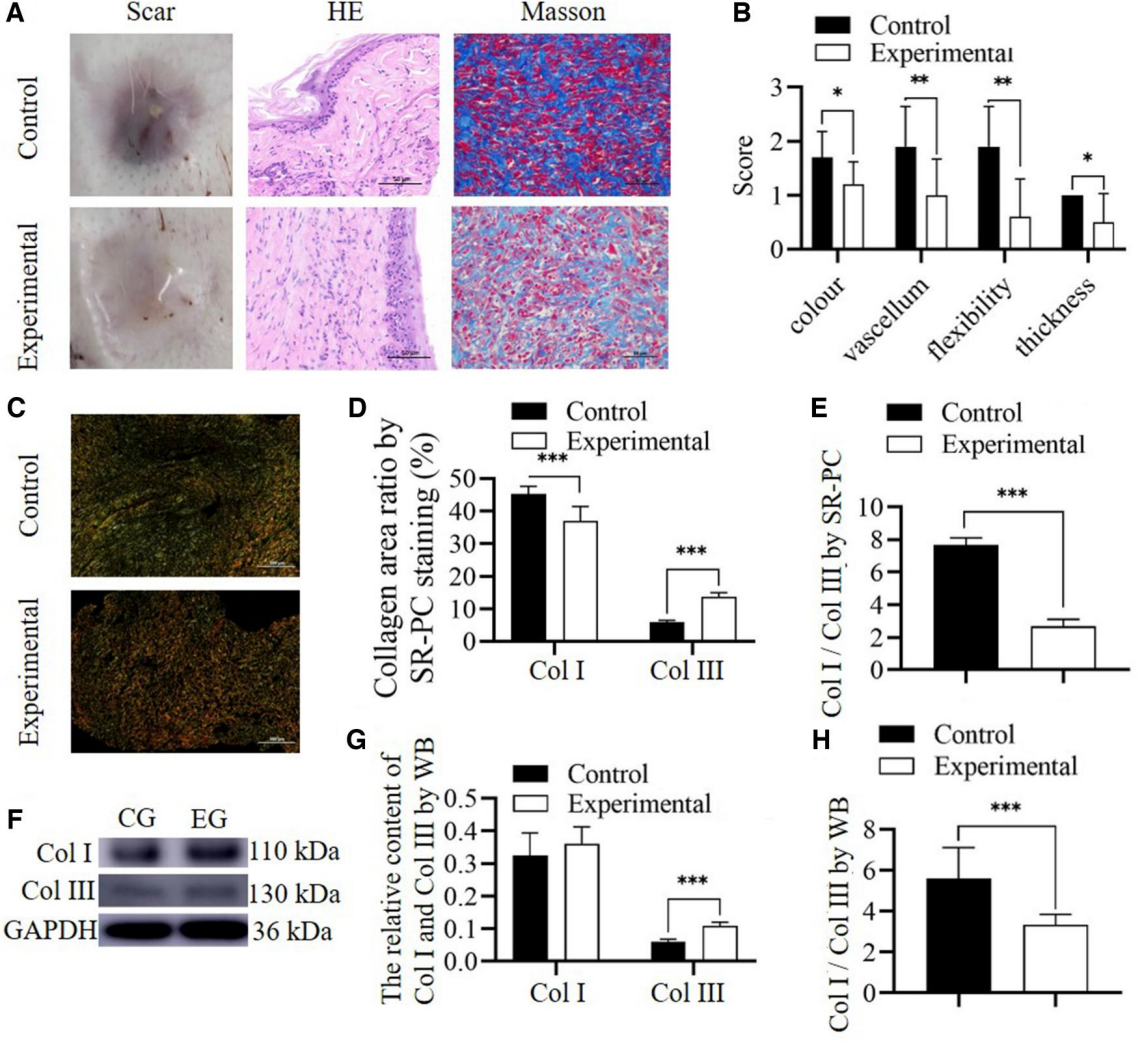
Rabbit ear scars. (**A**) Gross images of scars, HE and Masson-stained tissue sections. Scars in the experimental group were soft, not markedly different in color from peripheral skin, did not significantly increase above the skin surface, and the surface was smooth. HE and Masson staining revealed the absence of pathological hypertrophic scars and abundance of cutaneous ridges. (**B**) VSS scores. (**C**–**E**) Sirius red-picric acid staining assessing the levels of Col I and III (D), and the proportion of Col I/Col III (E). (**F**∼**H**) WB (CG, control group; EG, experimental group) assessing Col I and Col III contents(G), and Col I/Col III content ratio (H). *Significant differences compared to the control group (*P *<* *0.05). **Significant differences compared to the control group (*P *<* *0.01). ***Significant differences compared to the control group (*P *<* *0.001). HE, hematoxylin-eosin; GAPDH, glyceraldehyde 3-phosphate dehydrogenase; Col I, collagen type I; Col III, collagen type III; His, histidine; VSS, Vancouver scar scale.

As subjective scoring items in the VSS could not be completed, only the color, blood vessels, flexibility and thickness were analyzed. In the experimental group, hyperplastic scars were not apparent, the color and flexibility of scars not raised were identical to those of the peripheral skin, and various variables and the VSS scores were significantly lower than those in the control group (*n* = 10, *P* = 0.024, =0.01, <0.001 and =0.008, respectively), showed that RHC III chain markedly improved hypertrophic scars. The WB analysis results showed that His was not present in the experimental and control groups, and the immunofluorescence assay results of His indicated that the mean optical densities were not significantly different between the experimental and control groups (*n* = 5, *P* = 0.939 and =0.935, respectively). There was no significant difference in the expression of genes corresponding to Col I and Col III between the experimental group and the control group (*n* = 5, *P* = 0.078 and =0.299, respectively). The SR-PC staining results demonstrated that the Col III level increased and was noticeably higher (*n* = 5, *P* = 0.002), the Col I level decreased and was markedly lower (*n* = 5, *P* < 0.001), and the Col I/Col III ratio was lower in the experimental group than in the control group (*n* = 5, *P* < 0.001), but the total collagen level was not significantly different between the groups (*n* = 5, *P* = 0.380). The WB analysis results also showed a significant increase in Col III level in the experimental group compared with that in the control group (*n* = 5, *P* < 0.001). The Col I/Col III ratio was significantly lower in the experimental group than in the control group (*n* = 5, *P* = 0.013) (Figure 7 and Supplementary Figure S2-4). In SD rats, the Col I level did not considerably change in the wound tissues, whereas a substantial decrease was observed in the rabbit ear scars. Although there was no significant change in gene expression levels, even though the expression of Col I increased during the healing process, the raw material for Col I synthesis was insufficient, leading to a long-term decrease in the synthesis of Col I. This finding suggests that RHC III chain inhibited Col I synthesis and secretion via its long-term activity, which was conducive to reducing hypertrophic scars. This was possible because RHC III chain is not degraded into individual amino acids but rather small fragments. Col III had more amino acid sequences identical to those of small amino acid fragments compared to Col I, indicating that RHC III chain was the raw material of Col III rather than that of Col I. Furthermore, RHC III chain was utilized in the rabbit ear scar model before wound healing and was not further employed after wound healing. This finding indicated that RHC III chain had a long-acting effect on pathological hypertrophic scars. The hypertrophic scars became non-pathological after RHC III chain treatment, potentially improving the quality of wound healing. In addition, His was not detected in the tissues, further indicating that the endocytosed RHC III chain was degraded.

## Conclusion

This study investigated the general properties and cytocompatibility of RHC III chain synthesized via engineered yeasts. We conducted cell-based and animal experiments to further investigate and validate the effects of RHC III chain in promoting wound healing and alleviating and even preventing pathological hypertrophic scars. Endocytosed RHC III chain was degraded by cells to induce Col III synthesis, and the physiological activity of RHC III chain improved pathological hypertrophic scars, which merits attention. Improving pathological hypertrophic scars via the activity of RHC III chain provides a new option for wound treatment and scar-free healing. Nonetheless, the signaling pathways involved in the physiopathological activities following intracellular degradation of RHC III chain and post-degradation activation warrant further investigation.

## Supplementary Material

rbae149_Supplementary_Data

## Data Availability

The raw/processed data can be obtained from the authors on request.
