# Supplementary material for: Endocytosis-mediated healing: recombinant human collagen type III chain-induced wound healing for scar-free recovery
Source: Regen Biomater. 2025 Jan 8;12:rbae149. doi: 10.1093/rb/rbae149 (PMC11930350; doi:10.1093/rb/rbae149)
Supplement: rbae149_Supplementary_Data [file rbae149_supplementary_data.zip › 47d07_Supplementary 1-2.ISO 10993-6-2016.pdf]

INTERNATIONAL STANDARD

ISO 10993-6

Third edition
2016-12-01

Biological evaluation of medical devices —

Part 6: Tests for local effects after implantation

Évaluation biologique des dispositifs médicaux —

Partie 6: Essais concernant les effets locaux après implantation

Reference number
ISO 10993-6:2016(E)

© ISO 2016

COPYRIGHT PROTECTED DOCUMENT

© ISO 2016, Published in Switzerland

All rights reserved. Unless otherwise specified, no part of this publication may be reproduced or utilized otherwise in any form or by any means, electronic or mechanical, including photocopying, or posting on the internet or an intranet, without prior written permission. Permission can be requested from either ISO at the address below or ISO's member body in the country of the requester.

ISO copyright office
Ch. de Blandonnet 8 • CP 401
CH-1214 Vernier, Geneva, Switzerland
Tel. +41 22 749 01 11
Fax +41 22 749 09 47
copyright@iso.org
www.iso.org

Contents

Page

Foreword ..... **iv**
1 Scope **1**
2 Normative references **1**
3 Terms and definitions **2**
4 Common provisions for implantation test methods **2**
5 Test methods, general aspects **4**
6 Test report **9**
6.1 General 9
6.2 Test laboratory 9
6.3 Implant samples 9
6.4 Animals and implantation 9
6.5 Retrieval and histological procedure 10
6.6 Macroscopic and microscopic evaluation 10
6.7 Final evaluation 10
Annex A (normative) **Test methods for implantation in subcutaneous tissue** **11**
Annex B (normative) **Test method for implantation in muscle** **13**
Annex C (normative) **Test method for implantation in bone** **15**
Annex D (normative) **Test method for implantation in brain tissue** **18**
Annex E (informative) **Examples of evaluation of local biological effects after implantation** **23**
Bibliography **27**

© ISO 2016 – All rights reserved

Foreword

ISO (the International Organization for Standardization) is a worldwide federation of national standards bodies (ISO member bodies). The work of preparing International Standards is normally carried out through ISO technical committees. Each member body interested in a subject for which a technical committee has been established has the right to be represented on that committee. International organizations, governmental and non-governmental, in liaison with ISO, also take part in the work. ISO collaborates closely with the International Electrotechnical Commission (IEC) on all matters of electrotechnical standardization.

The procedures used to develop this document and those intended for its further maintenance are described in the ISO/IEC Directives, Part 1. In particular the different approval criteria needed for the different types of ISO documents should be noted. This document was drafted in accordance with the editorial rules of the ISO/IEC Directives, Part 2 (see www.iso.org/directives).

Attention is drawn to the possibility that some of the elements of this document may be the subject of patent rights. ISO shall not be held responsible for identifying any or all such patent rights. Details of any patent rights identified during the development of the document will be in the Introduction and/or on the ISO list of patent declarations received (see www.iso.org/patents).

Any trade name used in this document is information given for the convenience of users and does not constitute an endorsement.

For an explanation on the meaning of ISO specific terms and expressions related to conformity assessment, as well as information about ISO's adherence to the WTO principles in the Technical Barriers to Trade (TBT) see the following URL: Foreword - Supplementary information

The committee responsible for this document is ISO/TC 194, *Biological and clinical evaluation of medical devices*.

This third edition cancels and replaces the second edition (ISO 10993-6:2007), which has been technically revised with the following changes:

a) addition of guidance on biological evaluation of absorbable medical devices;

b) new Annex D.

ISO 10993 consists of the following parts, under the general title *Biological evaluation of medical devices*:

— *Part 1: Evaluation and testing within a risk management process*

— *Part 2: Animal welfare requirements*

— *Part 3: Tests for genotoxicity, carcinogenicity and reproductive toxicity*

— *Part 4: Selection of tests for interactions with blood*

— *Part 6: Tests for local effects after implantation*

— *Part 7: Ethylene oxide sterilization residuals*

— *Part 9: Framework for identification and quantification of potential degradation products*

— *Part 10: Tests for irritation and skin sensitization*

— *Part 11: Tests for systemic toxicity*

— *Part 12: Sample preparation and reference materials*

— *Part 13: Identification and quantification of degradation products from polymeric medical devices*

— *Part 14: Identification and quantification of degradation products from ceramics*

 © ISO 2016 – All rights reserved

— *Part 15: Identification and quantification of degradation products from metals and alloys*

— *Part 16: Toxicokinetic study design for degradation products and leachables*

— *Part 17: Establishment of allowable limits for leachable substances*

— *Part 18: Chemical characterization of materials*

— *Part 19: Physico-chemical, morphological and topographical characterization of materials* [Technical specification]

— *Part 20: Principles and methods for immunotoxicology testing of medical devices* [Technical specification]

— *Part 33: Guidance on tests to evaluate genotoxicity — Supplement to ISO 10993-3* [Technical Report]

The following parts are under preparation:

— *Part 5: Tests for in vitro cytotoxicity*

© ISO 2016 – All rights reserved

Biological evaluation of medical devices —

Part 6: Tests for local effects after implantation

1 Scope

This part of ISO 10993 specifies test methods for the assessment of the local effects after implantation of biomaterials intended for use in medical devices.

This part of ISO 10993 applies to materials that are

— solid and non-absorbable,

— non-solid, such as porous materials, liquids, gels, pastes, and particulates, and

— degradable and/or absorbable, which may be solid or non-solid.

The test sample is implanted into a site and animal species appropriate for the evaluation of the biological safety of the material. These implantation tests are not intended to evaluate or determine the performance of the test sample in terms of mechanical or functional loading. This part of ISO 10993 can also be applied to medical devices that are intended to be used topically in clinical indications where the surface or lining might have been breached, in order to evaluate local tissue responses.

The local effects are evaluated by a comparison of the tissue response caused by a test sample to that caused by control materials used in medical devices whose clinical acceptability and biocompatibility characteristics have been established. The objective of the test methods is to characterize the history and evolution of the tissue response after implantation of a medical device/biomaterial including final integration or absorption/degradation of the material. In particular for degradable/absorbable materials, the degradation characteristics of the material and the resulting tissue response should be determined.

This part of ISO 10993 does not deal with systemic toxicity, carcinogenicity, teratogenicity or mutagenicity. However, the long-term implantation studies intended for evaluation of local biological effects might provide insight into some of these properties. Systemic toxicity studies conducted by implantation might satisfy the requirements of this part of ISO 10993. When conducting combined studies for evaluating local effects and systemic effects, the requirements of both standards is to be fulfilled.

2 Normative references

The following documents, in whole or in part, are normatively referenced in this document and are indispensable for its application. For dated references, only the edition cited applies. For undated references, the latest edition of the referenced document (including any amendments) applies.

ISO 10993-1, *Biological evaluation of medical devices — Part 1: Evaluation and testing within a risk management process*

ISO 10993-2, *Biological evaluation of medical devices — Part 2: Animal welfare requirements*

ISO 10993-4, *Biological evaluation of medical devices — Part 4: Selection of tests for interactions with blood*

ISO 10993-12, *Biological evaluation of medical devices — Part 12: Sample preparation and reference materials*

© ISO 2016 – All rights reserved

ISO 10993-16, *Biological evaluation of medical devices — Part 16: Toxicokinetic study design for degradation products and leachables*

3 Terms and definitions

For the purposes of this document, the terms and definitions given in ISO 10993-1, ISO 10993-2, ISO 10993-12, ISO 10993-16 and the following apply.

3.1
absorb/absorption
action of a non-endogenous (foreign) material or substance, or its decomposition products passing through or being assimilated by cells and/or tissue over time

3.2
degradation
decomposition of a material

[SOURCE: ISO 10993-9:2009, 3.1]

3.3
degradation product
any intermediate or final by-product which results from the physical, metabolic, and/or chemical decomposition of a material or substance

[SOURCE: ISO/TR 37137:2014, 2.2, modified]

3.4
degrade
to physically, metabolically, and/or chemically decompose a material or substance

[SOURCE: ISO/TR 37137:2014, 2.3]

3.5
biomaterial
material or substance intended to interface with biological systems to evaluate, treat, augment or replace any tissue, organ or function of the body.

[SOURCE: European Society Biomaterials Conference II]

4 Common provisions for implantation test methods

4.1 General

It is important that the study be planned in sufficient detail such that all relevant information can be extracted from the use of each animal and each study (see ISO 10993-2, ISO 10993-11 and ISO 10993-16).

All animal studies shall be performed in a facility approved by a nationally recognized organization and in accordance with all appropriate regulations dealing with laboratory animal welfare to comply with the requirements of ISO 10993-2. These studies shall be performed under good laboratory practices or other recognized, quality assurance systems.

The provisions of this Clause shall apply to the test methods specified in Annex A, Annex B, Annex C, and Annex D.

4.2 Preparation of samples for implantation

4.2.1 Test sample and reference or control material preparation shall be in accordance with ISO 10993-12. The implant size and shape shall be documented and justified. Test samples for various implant sites are described in Annex A, Annex B, Annex C, and Annex D. Physical characteristics (such as form, density,

© ISO 2016 – All rights reserved

hardness, surface) can influence the character of the tissue response to the test material and shall be recorded and taken into account when the response is characterized. Control articles should be matched as closely as reasonably possible for physical characteristics.

4.2.2 Each implant shall be manufactured, processed, cleaned of contaminants, and sterilized by the method intended for the final product and this shall be confirmed in the study documentation. After final preparation and sterilization, the implant samples shall be handled aseptically and in such a way as to ensure that they are not damaged or contaminated in any way prior to or during implantation.

4.2.3 For materials used as scaffolds for tissue-engineered medical products, it may be appropriate not to use the final preparation pre-populated with cells and/or proteins, as the immune reaction of the animal to the cellular/protein components of such products and the reaction of the cells to the animal may interfere with the resulting local tissue response, making it difficult to interpret.

4.2.4 For composite materials (e.g. bone cements, dental materials), the components may be mixed before use and allowed to set before implantation. For multicomponent materials designed to be cured prior to placement, the components may be mixed before use and allowed to set before implantation. However, materials that are designed to polymerize *in situ* (e.g. bone cements, many dental materials) shall be introduced in a manner such that *in situ* polymerization occurs. The procedure used shall be documented and justified

4.2.5 Non-solid materials (including powders) may be contained in open-ended cylindrical tubes for the purpose of testing for local effects after implantation (see ISO 10993-12 for the selection of materials for tubes). Prepare the test material according to the manufacturer's instructions and insert the material into the tube until level with the end, taking care not to contaminate the outer surface of the tube with the test material. If contamination occurs, the sample shall not be implanted. Avoid entrapment of air in the tube and ensure that the end surfaces of the inserted material in the tube and the tube ends are smooth.

Polyethylene (PE), polypropylene (PP), or polytetrafluoroethylene (PTFE) tubes are commonly used for this purpose. PE tubes can be deformed by autoclaving.

4.2.6 Evaluation shall be performed by comparing the tissue reaction to that of a similar sample/material whose clinical acceptability and biocompatibility characteristics have been established.

NOTE For further guidance, see ISO 10993-12.

4.2.7 The physical characteristics such as shape, and especially the surface condition of the control(s), shall be as similar to that of the implant test samples as is practical, with any deviations being explained and justified. When the test material is contained in a tube, the control shall be of the same material as the tube and have the same diameter as the outer diameter of the tube. The choice of the control rod or tube shall be documented and justified.

4.2.8 For implantation studies, the amount or size of the test and control article shall be documented.

4.3 Study design

For devices comprising/composed of two or more different materials, the test articles should be of similar composition or multiple implants may be needed, e.g. if a device is made of HDPE and titanium then the test article should be made of HDPE and titanium.

© ISO 2016 – All rights reserved

5 Test methods, general aspects

5.1 Tissue and implantation site

5.1.1 The test sample shall be implanted into the tissues most relevant to the intended clinical use of the material. The justification for the choice of sample numbers, tissue and implantation sites shall be documented. Test methods for various implantation sites are given in Annex A, Annex B, Annex C, and Annex D. If other implantation sites are chosen, the general scientific principles behind the test methods described in Annex A, Annex B, Annex C, and Annex D shall still be adhered to and the justification provided.

NOTE For some devices, there are vertical standards prescribing specific implant studies to evaluate local tissue responses, e.g. intraocular lens implant[47] and dental usage tests[12]. These studies can be used to satisfy the requirements in ISO 10993-6.

5.1.2 For absorbable materials, the implantation site shall be marked in a manner suitable for identification of the site at the end of the designated time periods. The use of a non-invasive permanent skin marker and/or a template marking the placement of the sample is recommended for short-term study intervals only. In most circumstances, a location marker comprised of an appropriate non-absorbable negative control (e.g. HDPE 1 mm by 2 mm by 5 mm, PP suture, gold band, clips) may be used to identify the location of the implant site. These location markers can be removed without inducing artefacts to the test article-tissue interface prior to histology processing.

Exceptionally, a sham surgical procedure might be used to evaluate the impact of the procedure on the tissue involved; in these cases, the specific justification shall be provided.

5.2 Animals

5.2.1 All aspects of animal care and accommodation shall be in accordance with ISO 10993-2. In general, small laboratory animals such as mice, rats, hamsters, or rabbits are preferred.

5.2.2 The use of larger animals may be justified based upon special scientific considerations of the particular biomaterial under study, or if needed to accommodate implant size, with whole device testing.

5.2.3 Select an animal species in line with the principles set out in ISO 10993-2, giving due consideration to the size of the implant test samples, the number of implants per animal, the intended duration of the test in relation to the expected lifespan of the animals, as well as potential species' differences regarding biological response.

5.2.4 For short-term testing, animals such as rodents or rabbits are commonly used. For long-term testing, animals such as rodents, rabbits, dogs, sheep, goats, pigs, and other animals with a relatively long life expectancy are suitable.

5.2.5 Before starting an animal study with degradable materials, relevant information from in vitro degradation studies should be considered. For absorbable materials, a pilot study in rodents may be considered to determine the expected rate of degradation before embarking on studies on larger animals.

5.2.6 The samples of test and control materials shall be implanted under the same conditions in animals of the same species and of the same age, sex, and strain in corresponding anatomical sites. The number and size of implants inserted into an animal depends on the size of the species and the anatomical location. Whenever possible, the reference control and the test samples should be implanted into the same animal.

 © ISO 2016 – All rights reserved

5.2.7 However, when a neuroimplantation study (see Annex D) is conducted or when the local effects after implantation are investigated as part of a systemic toxicity study by implantation, control and test samples shall not be placed in the same animal.

5.3 Test periods

5.3.1 The test period shall be determined by the likely clinical exposure time or be continued until or beyond when a steady-state with respect to the biological response has been reached. The time points selected shall be explained and justified.

5.3.2 For non-absorbable materials, the short-term responses are normally assessed from 1 week up to 4 weeks and the long-term responses in tests exceeding 12 weeks. The local biological response to implanted materials depends both on the properties of the materials and on the response to the associated trauma of surgery. The tissue configuration in the vicinity of an implant changes with the time elapsed after surgery. During the first two weeks after implantation, the reaction due to the surgical procedure itself may be difficult to distinguish from the tissue reaction evoked by the implant. In muscle and connective tissue, depending on the species, and the severity of the surgical trauma, a steady-state is seen in the cell population after 9 weeks to 12 weeks. Implantation in bone tissue may need longer observation periods before a steady-state is reached.

5.3.3 For absorbable materials, the test period shall be related to the estimated degradation time of the test product at a clinically relevant implantation site. When determining the time points for sample evaluation, an estimation of the degradation time shall be made. This can be accomplished *in vitro* by real-time or accelerated degradation studies or in certain circumstances by mathematical modelling. In general, study duration should extend up to or beyond the point of complete absorption. The evaluation period for absorbable materials will depend in part on the degradation rate of the materials. Study intervals should span a significant portion of the degradation time frame for the implant, and shall include, as a minimum, the following time points:

a) early time frame (where there is no or minimal degradation) — For absorbable materials, usually a study interval of between 1 week and 2 weeks post-implantation should be used to assess the early tissue response.

b) mid time frame (when degradation is taking place) — Subsequent study intervals for absorbable devices should be guided by the degradation profile of the specific absorbable material. The target interval should allow assessment of histological response when the tissue response is expected to be most pronounced (e.g. substantial structural disruption and/or fragmentation of the device is most likely to occur). Implants with longer-term degradation profiles may require multiple assessment time points, with intervals targeted in accordance with the expected pattern of degradation.

   When a device with multiple materials with differing absorption rates is implanted, implant intervals reflecting the degradation profile of those components should be included.

c) late time frame (when the implant is essentially absorbed) — This interval is targeted to observe when minimal amounts of the absorbable component remain at the implant site.

   Gross and microscopic evaluation after complete implant absorption is highly desirable. However, in the absence of complete absorption, the overall data collected should be sufficient to allow characterization of the local effects after implantation if:

   — the affected tissue's response, structure, and function have achieved an acceptable steady-state condition, and

   — the absorbable material and/or its degradation products are in a state of limited visually-identifiable presence.

© ISO 2016 – All rights reserved

NOTE *In vivo* degradation can occur over a long period of time, sometimes more than one year. Additional animals to extend the observation period (intervals "to-be-determined" group) can be beneficial if the implant has not been completely absorbed within the expected investigational time period and cannot be observed microscopically.

In those situations when the material is not fully absorbed within the late time frame, an appropriate scientific justification can be included for ending the study and the estimated percentage (%) of remaining absorbable material should be reported.

Long term studies that span a significant portion of the degradation time frame for the implant are recommended. Implantation of *in vitro* pre-degraded material (for instance, up to 50 % weight loss or 50 % loss of mechanical strength) may be considered on a case-by-case basis in order to more rapidly observe late stage events after implantation. However, these studies do not replace studies that characterize the real-time *in vivo* degradation profile of the absorbable device.

5.3.4 Characterization of an absorbable device's degradation process may not be applicable to the evaluation of the local effects of the same absorbable material when used in combination: with a drug as carrier for drug release, a scaffold for tissue-engineered medical products, or a surface coating for non-absorbable implants. Since combinations of devices with drugs and/or cells can introduce new issues, the appropriate regulatory authorities should be consulted regarding study designs for absorbable combination products.

5.3.5 Although this part of ISO 10993 does not address the issues of systemic toxicity given in ISO 10993-11, it is recommended that the information required to meet this part of ISO 10993 be obtained from any systemic toxicity studies using implantation.

5.3.6 For long-term studies, generally accepted observation periods for non-absorbable biomaterials are given in Table 1. Animals should be humanely sacrificed at each time point, in line with ISO 10993-2. Serial harvest under general anaesthesia with recovery may be acceptable under special circumstances, which shall be documented and justified.

Table 1 — Possible test periods for long-term implantation of biomaterials

Species	Implantation period in weeks[a]				
	**13**	**26**	**52**	**78**	**104**
Mice	X	X	X	—	—
Rats	X	X	X	—	—
Guinea-pigs	X	X	X	—	—
Rabbits	X	X	X	X	X
Dogs	X	X	X	X	X
Sheep	X	X	X	X	X
Goats	X	X	X	X	X
Pigs	X	X	X	X	X

[a] These implantation periods are commonly used; however, other periods may be applicable based on the specific characteristics of the test material. Depending on the intended use of the test material, not all implantation periods may be necessary.

5.4 Surgery and testing conditions

5.4.1 Surgery shall be performed under general anaesthesia. If another type of anaesthesia is used, this shall be justified and shall be in compliance with ISO 10993-2. The specific insertion or implantation procedures for subcutaneous, intramuscular, bone or neural implantation are described in Annex A, Annex B, Annex C, and Annex D, respectively.

 © ISO 2016 – All rights reserved

5.4.2 The number of implants per animal and the number of animals per observation period are described in Annex A, Annex B, Annex C, and Annex D. A sufficient number of test and control samples shall be implanted to ensure that the final number of samples to be evaluated will give valid results.

5.4.3 The surgical technique may profoundly influence the result of any implantation procedure. Surgery shall be carried out under aseptic conditions and in a manner that minimizes trauma at the implant site. Remove the hair from the surgical area by clipping, shaving, or other mechanical means. Disinfect the exposed area of skin with an appropriate antiseptic. Ensure that the implants or wound surfaces do not come in contact with the hair. After surgery close the wound using either sutures or wound clips, taking precautions to maintain aseptic conditions. Use of antibiotics should be justified.

5.4.4 The health of the animals shall be observed and recorded at regular intervals during the study. Following surgery, each animal shall be observed at appropriate intervals during the test period, and any abnormal findings shall be recorded, including local, systemic, and behavioural abnormalities, and their potential influence on the results obtained described in the test reports.

5.4.5 Body-mass measurements should be taken at appropriate intervals. The use of post-operative analgesics shall be in line with the requirements of ISO 10993-2.

5.4.6 At the end of the experimental period, euthanize the animals with an overdose of anaesthetic or by some other humane method in line with the principles set out in ISO 10993-2.

5.5 Evaluation

5.5.1 General

Evaluate the biological response by documenting the macroscopic and histopathological responses as a function of time. Compare the responses to the test sample to the responses obtained at the control sample or sham operated sites.

NOTE Examples of grading systems are given in Annex E and in the Bibliography.

Carry out comparison of the control and test implants at equivalent locations relative to each implant, so that the effect of relative motion between the tissue and implant is at a minimum.

For a cylindrical sample, this region is midway between its ends. With grooved cylindrical implants, the centre portions between the grooves, as well as the flat top end surfaces of the implant are suitable for evaluation.

For each of the implant intervals, a sufficient number of samples shall be evaluated as defined in Annex A, Annex B, Annex C, and Annex D. These samples shall be obtained from at least three different animals.

In exceptional circumstances, where less than the original number of implanted sites are available for evaluation, or in cases of animal loss, the evaluating pathologist may determine if the number of sites are uniform in their response, such that an accurate overall evaluation can be made.

5.5.2 Macroscopic assessment

Each implant site shall be examined for alterations of the normal structure. This should include assessment of the regional draining lymph nodes[32]. Use of a lens with low magnification is recommended. Record the nature and extent of any tissue reaction observed, such as haematoma, oedema, encapsulation, and/or additional gross findings. Record the presence, form, and location of the implant, including possible remnants of degradable materials. The use of colour macro photography can be useful for documentation.

In addition to the inspection of the implant site, whenever an animal has shown signs of ill health or reactions to the implant, a gross necropsy shall be conducted.

© ISO 2016 – All rights reserved

5.5.3 Implant retrieval and tissue sample collection

After the animal has been humanely euthanized, excise the implant site together with sufficient unaffected surrounding tissue (2 mm to 5 mm) to enable evaluation of the local histopathological response. If the candidate material is not evident at the site examined (absorbable materials), extend the explantation site to include several millimetres of normal tissue on all sides of the expected implant site. Chemical fixation of the implant site containing the test and/or control material may be done at this stage. Chemical fixation in 10 % formalin solution is suitable for most materials and stains. Fixation for 24 h to 72 h is reasonable depending on tissue sample size. Once chemically fixed, hard materials, like metals or dense plastics can be carefully removed from the peri-implant capsule. The capsule marks the implant cavity. Some soft materials may be able to be trimmed and left *in situ* for processing and sectioning under paraffin microtomy. This can be preferable if the materials are porous and there is tissue ingrowth over time.

For non-degradable implants, draining lymph nodes should be collected as indicated by the gross pathology. For degradable implants, draining lymph nodes should be collected, when feasible, as evaluation of draining lymph nodes is important to demonstrate migration of degradable materials.

NOTE 1 It is recognized that it is not always possible to locate the draining lymph nodes of all samples.

If indicated by ill health, and gross pathology, or by experimental design to assess systemic toxicity, other organs shall be collected as appropriate.

Process the excised tissue samples according to appropriate procedures needed for histological evaluation, including fixation, excision, embedding, sectioning, and staining. If appropriate, record the orientation of the implant, number of sections, section thickness, and cutting geometry.

When conventional techniques (paraffin embedding) are used, the tissue envelope may be opened before or after exposure to a fixative and the condition of the implant surface and tissue bed shall be reported. Take care not to destroy the implant/tissue interface if the envelope is opened on fresh unfixed tissues. When the implant/tissue interface is to be studied in hard materials like metals or dense plastics, embedding of the intact tissue envelope with the implant *in situ* using hard plastics instead of paraffin is preferred; appropriate sectioning or grinding techniques are used for the preparation of histological sections.

When the tissues or implants cannot be sectioned in paraffin, other embedding/sectioning techniques (e.g. plastic embedding) may be necessary for tissue/implant interface evaluation. If embedding techniques alter the tissue/implant interface, any observations at the interface shall be documented.

NOTE 2 For “soft” implants in soft tissues, processing of the tissue samples can be performed without removing the implant.

5.5.4 Microscopic assessment

The scoring system used for the histological evaluation shall take into account the extent of the area affected, either quantitatively (e.g. in micrometres) or semiquantitatively (see Annex E). The implant orientation, number of sections and cutting geometry should be recorded.

The biological response parameters, which shall be assessed and recorded, include the following:

a) the extent of fibrosis/fibrous capsule; layer in micrometres or semiquantitatively (see Annex E) and inflammation;

b) the degeneration as determined by changes in tissue morphology;

c) the number and distribution as a function of distance from the material/tissue interface of the inflammatory cell types, namely, polymorphonuclear cells, lymphocytes, plasma cells, eosinophils, macrophages, and multinucleated cells;

d) the presence and extent of necrosis;

 © ISO 2016 – All rights reserved

e) other tissue alterations, such as vascularization, fatty infiltration, granuloma formation, mineralization, and bone formation;

f) material parameters, such as fragmentation and/or debris presence, form and location of remnants of degraded material;

g) the quality and quantity of tissue ingrowth, for porous and absorbable implant materials.

Histological responses, including any adverse findings, shall be documented. Photomicrographs can be useful for documentation.

For degradable/absorbable materials, at the intermediate or nearly complete degradation levels, some residual material of the degradable implant shall be present in the tissue samples examined. In addition, for evaluation of the restoration to normal structure, representative areas of the implant site shall be evaluated, as indicated by marker or template.

For implants in bone, the interface between the tissue and the material is of special interest. Evaluate the area of bone contact and the amount of bone in the vicinity of the implant, as well as the presence of intervening non-calcified tissues. The presence of bone resorption or new bone formation shall be recorded.

In addition to the standard Hematoxylin and Eosin histopathology assessment, additional analysis is recommended in the event of adverse histopathology findings (e.g. immune cell infiltration).

5.5.5 Evaluation of responses

Examples of quantitative scoring systems are given in References [25] and [26].

Examples of semiquantitative scoring systems are given in Annex E and in References [17], [18], and [20].

In addition, examples of other scoring systems are included in the Bibliography.

6 Test report

6.1 General

The test report shall have sufficient detail to allow an independent assessment of the results. When there is more than one device material, the pathologist should evaluate and report on each material individually. The report shall include the items listed in 5.1 to 5.5. In addition, the following items shall be reported.

6.2 Test laboratory

a) Name of the testing laboratory and the certifications of the laboratory.

b) Date, name, and signature of the person(s) responsible for the report.

6.3 Implant samples

a) Description of test and control materials, such as identification, surface condition, and the shape, size, weight, and form of the implants.

b) The rationale for choice of control sample and the physical form of the material implanted shall be given.

6.4 Animals and implantation

a) Species, strain, sex, age, and/or weight and origin shall be reported and justified.

© ISO 2016 – All rights reserved

b) Test conditions, including housing and diet shall be reported.

c) All animal welfare observations during the study shall be recorded and documented.

d) Insertion techniques, including the surgical procedure, anaesthesia and post-surgical analgesia, and the location and number of implants per animal shall be recorded and reported.

e) Problems associated with implantation or explantation and all observations made during the study shall be recorded.

6.5 Retrieval and histological procedure

a) The report shall include a description of the retrieval technique. The number of implants retrieved per animal and per observation period shall be recorded.

b) Implant evaluation, including gross observations of implants, tissues, and organs, shall be recorded. Techniques employed for the fixation and preparation of the histological sections shall be described.

c) Methods and results of histological evaluation of implant site and any organs showing alterations at necropsy, when indicated.

d) For absorbable materials, the report shall include, but not be limited to, a description of the degree of degradation, including material characteristics at explant (free particles, fibre formation, amorphous gel, crystallinity). Potentially relevant additional observations, such as molecular weight changes and mass loss, should be considered if the implant can be removed without damaging the implant/tissue interface.

e) When the ultimate goal of an implant is to result in tissue remodelling, evaluation of the formation of the expected normal tissue at the site rather than complete degradation might be considered.

6.6 Macroscopic and microscopic evaluation

a) Macroscopic observations shall include the observations made on each implant as well as the macroscopic appearance of the tissue surrounding the implant. When applicable, this shall include observation of the draining lymph nodes, especially for absorbable materials.

b) The report shall include the results obtained from each histological examination and (statistical) analysis when applied. When applicable, this shall include observation of the draining lymph nodes, especially for absorbable materials.

6.7 Final evaluation

The report shall include a comparative evaluation of the local effects after implantation in terms of the biological responses to test and control materials.

© ISO 2016 – All rights reserved

Annex A
(normative)

Test methods for implantation in subcutaneous tissue

A.1 Field of application

This test method is used for assessing the biological response of subcutaneous tissue to an implanted material.

The study may be used to compare the effect of different surface textures or conditions of the same material, or to assess the effect of various treatments or modifications of a material.

A.2 Principle

The biological response to implants of test samples is compared with the biological response to implants of control samples. The control materials are those used in medical devices whose clinical acceptability and biocompatibility characteristics have been established.

A.3 Test samples

Common provisions for the preparation of test and control samples are described in 4.2. Implant sizes are based on the size of the test animal. The following shall be considered minimum dimensions.

a) When using discs, test samples of 10 mm to 12 mm in diameter and from 0,3 mm to 1,0 mm in thickness.

   NOTE The subcutaneous site, deep to the panniculus carnosa muscle, is particularly suitable for the evaluation of polymeric sheet material. In an intramuscular site sheet, material may become folded, which makes it difficult to assess the effect of the material per se.

b) When using rods and cylinders, test samples shall be 1,5 mm to 2 mm in diameter, 5 mm to 10 mm in length and have rounded ends.

c) Non-solid samples (including powders) should be prepared in tubes 1,5 mm in diameter and 5 mm in length (see 4.2). If appropriate, these materials may be implanted directly into the tissues. However, a location marker is recommended for absorbable materials.

d) Other dimensions that are anatomically compatible may be utilized, when conducting implantation tests in conjunction with systemic toxicity studies with clinically relevant samples.

A.4 Test animals and implant sites

The implants shall be inserted in the dorsal subcutaneous tissue of adult mice, rats, guinea-pigs, or rabbits. Select one species among these in accordance with the provisions of ISO 10993-2.

Use at least three animals for each material and sufficient sites to yield a total of 10 tests and 10 control samples for each material and implantation period. When multiple tissue samples are taken from a single implant site, sections for histology shall be at least 1 cm apart.

© ISO 2016 – All rights reserved

Tissue samples to be evaluated for a material shall originate from at least three animals. A non-absorbable control sample shall be evaluated at each time point. A single time control point is acceptable provided an acceptable scientific justification is documented, which shall address the following:

— the control sample;

— the implantation duration;

— animal model;

— study protocol;

— the historical control data.

A.5 Implantation procedure

A.5.1 General

Select one of the procedures described in A.5.2 and A.5.3.

A.5.2 Implantation alongside dorsal midline

Make an incision in the skin and make one or more subcutaneous pockets by blunt dissection. The base of the pocket shall be more than 10 mm from the line of incision. Place one implant in each pocket. The implants shall not be able to touch one another. Alternatively, both flanks may be used.

NOTE Alternatively, the implants can be delivered by a trocar to the desired site or, when indicated, multiple small incisions can be made.

A.5.3 Implantation in the neck

In mice, make a 10 mm long incision above the sacrum and prepare a subcutaneous tunnel by blunt dissection towards the neck. Push one implant through the tunnel to position it at the neck[23][24].

In rats, insert one implant of each of the control and candidate materials separately on each side of the neck. The implants shall not be able to touch one another. Alternatively, both flanks and/or hind legs may be used.

At some distance from the implant, close the tunnel with stitches of appropriate suture material to prevent the implant from moving.

A.6 Implantation period

To ensure a steady-state of biological tissue response, the implantation period(s) shall be selected as specified in 5.3.

A.7 Evaluation of biological response

The evaluation shall take into account the items specified in Clause 5.

A.8 Test report

The presentation of the test results and final test report shall include the items specified in Clause 6 and shall include justifications for the specific methods selected.

 © ISO 2016 – All rights reserved

Annex B
(normative)

Test method for implantation in muscle

B.1 Field of application

This test method is used for assessing the biological response of muscle tissue to an implanted material.

B.2 Principle

The implant is inserted into the muscles of test animals. The biological response to implants of test samples is compared with the biological response to implants of control samples. The control materials are those used in medical devices whose clinical acceptability and biocompatibility characteristics have been established.

B.3 Test samples

Common provisions for preparation of test and control samples are described in 4.2. Implant sizes are based on the size of the muscle group chosen.

For rabbit paravertebral muscles, implants of a width of 1 mm to 3 mm with a length of approximately 10 mm are typically used. Alternatively, larger samples up to 10 mm in diameter and 3 mm in thickness may be surgically implanted.

Other dimensions that are anatomically compatible may be utilized, when conducting implantation tests in conjunction with systemic toxicity studies with clinically relevant samples.

The samples shall have rounded edges and the ends finished to a full radius.

B.4 Test animals and implant sites

Ensure that the muscles are of sufficient size to accommodate the implant samples. Use only one species per test. Insert the implants in the muscle of the animals under anaesthesia.

NOTE The paravertebral muscles of rabbits are the preferred implant sites. Alternatively, for smaller samples, the gluteal muscles of rats or the thigh muscles of rabbits can be used.

Use at least three animals and sufficient implant sites to yield a total of 10 test samples and 10 control samples for each implantation period.

The test and control samples to be evaluated shall be from at least three different animals.

In cases where a comparative control material is expected to elicit more than a minimal response, use an additional control material known to evoke a minimal tissue reaction in a location opposite the test materials.

A non-absorbable control sample shall be evaluated at each time point. A single time control point is acceptable provided an acceptable scientific justification is documented, which shall address the following:

— the control sample;

— the implantation duration;

© ISO 2016 – All rights reserved

— animal model;

— study protocol;

— the historical control data.

B.5 Implantation procedure

Implantation shall be by hypodermic needle or trocar. For larger implants, other appropriate surgical implantation techniques may be used.

Implant test samples into the body of the muscle with the long axis parallel to the muscle fibres.

For rabbit paravertebral muscles, implant sufficient samples of the test materials along one side of the spine, 25 mm to 50 mm from the midline and parallel to the spinal column, and about 25 mm apart from each other. In similar fashion, implant sufficient samples of the control material in the contralateral muscle of each animal.

B.6 Implantation period

To ensure a steady-state of biological tissue response, the implantation period(s) shall be as specified in 5.3.

B.7 Evaluation of biological response

The evaluation shall take into account the requirements specified in 5.5.

B.8 Format of test report

The presentation of the test results and final test report shall include the requirements specified in Clause 6.

© ISO 2016 – All rights reserved

Annex C
(normative)

Test method for implantation in bone

C.1 Field of application

This test method is used for assessing the biological response of bone tissue to an implanted material. The implantation site in cancellous ("spongy") or dense compact bone should be selected in accordance with the end use of the material.

The study may be used to compare the effect of different surface textures or conditions of the same material, or to assess the effect of various treatments or modifications of a material.

C.2 Principle

The implant is inserted into the bone tissue of test animals. The biological response to implants of test samples is compared with the biological response to implants of control samples. The control materials are those used in medical devices of which the clinical acceptability and biocompatibility characteristics have been established.

C.3 Test samples

C.3.1 General

Common provisions for preparation of test and control samples are specified in 4.2.

C.3.2 Shape of implant samples

Solid samples may be screw-shaped or threaded to provide initial stability of the implants in the bone. If preparation of a screw shape is impractical, a cylinder shape may be used.

Other sample forms (e.g. rods, pastes) may be used depending on the nature of the materials and study objective.

C.3.3 Size of test samples

Implant sizes are based on the size of the test animal and bone chosen. The following typical dimensions shall be considered for implants in mid-shaft cortical bone.

a) **Rabbits:** cylindrical implants 2 mm in diameter and 6 mm in length;

b) **Dogs, sheep and goats:** cylindrical implants of 4 mm in diameter and 12 mm in length;

c) **Rabbits, dogs, sheep, goats, and pigs:** 2 mm to 4,5 mm orthopaedic bone screw-type implants;

Other dimensions that are anatomically compatible may be utilized, when conducting implantation tests in conjunction with systemic toxicity studies with clinically relevant samples.

© ISO 2016 – All rights reserved

C.4 Test animals and implant sites

C.4.1 Test animals

The implants shall be inserted into the bone of rodents, dogs, sheep, goats, pigs, or rabbits. Select one species among these in line with the principles set out in ISO 10993-2. Species differences are important in bone physiology and should be assessed before implantation procedures are initiated. In addition, bone quality may vary between non-purpose-bred animals of the same species, and bone densitometry may be required to identify suitable test animals and to interpret the test results. Selection shall be justified and documented.

C.4.2 Implant sites

Equivalent anatomical sites shall be used for test and control samples. The test implants shall be contralateral to the control implants. Select the implant site to minimize the risk of mobility of the implant. At least 10 test samples and 10 control samples shall be evaluated for each implantation period. Tissue samples to be evaluated for a material shall originate from at least three animals.

A non-absorbable control sample shall be evaluated at each time point. A single time control point is acceptable provided an acceptable scientific justification is documented, which shall address the following:

— the control sample;

— the implantation duration;

— animal model;

— study protocol;

— the historical control data.

The femur and tibia are typically utilized. Other sites may be suitable.

The number of implant sites shall be as follows.

a) In each rabbit there shall be a maximum of six implant sites:

 — three for test samples;

 — three for control samples.

b) In each dog, sheep, goat, or pig, there shall be a maximum of 12 implant sites:

 — six for test samples;

 — six for control samples.

Do not insert more than 12 samples in any one animal.

The size, mass and age of the animal and the implant site chosen should ensure that the implant placement does not cause significant risk of pathological fracture of the test site. In younger animals, it is especially important to ensure that the epiphyseal area or other immature bone be avoided.

C.5 Implantation procedure

Perform bone preparation using low drilling speed and intermittent drilling using profuse irrigation with physiological saline solution and suction, because overheating will result in local tissue necrosis.

It is important that the diameter of the implant and the implant bed in the bone match well enough to avoid ingrowth of fibrous tissue.

© ISO 2016 – All rights reserved

Expose the cortex of each femur or tibia and drill the appropriate number of holes to receive implants. For rabbits, prepare up to three holes; for larger animals prepare up to six holes. Ream to final diameter or tap screw thread before insertion. Insert cylinders by finger pressure to allow press fit. Tighten screw-shaped implants in place with an instrument capable of delivering a predetermined torque. Record the torque.

C.6 Implantation period

To ensure a steady-state of biological tissue response, the implantation period(s) shall be as specified in 5.3.

C.7 Evaluation of biological response

The evaluation shall take into account the requirements specified in 5.5.

C.8 Test report

The presentation of the test results and final test report shall include the requirements specified in Clause 6.

© ISO 2016 – All rights reserved

Annex D
(normative)

Test method for implantation in brain tissue

D.1 Field of application

This test method is used for assessing the biological response of brain tissue to an implanted material. The implantation site in the brain should be selected in accordance with the end use of the material.

The material for a neuro-interventional device, which is in contact with the vessel wall but not with neural tissue directly, shall be evaluated in accordance with ISO 10993-4.

EXAMPLE An electrode implanted into the brain, shunts for hydrocephalus correction, drains.

D.2 Principle

The implant is inserted into the neural tissue of test animals. The biological response to implants of test samples is compared with the biological response to implants of control samples. The control materials are those used in medical devices whose clinical acceptability and biocompatibility characteristics have been established.

D.3 Test samples

D.3.1 General

Common provisions for preparation of test and control samples are described in 4.2.

A non-absorbable control sample shall be evaluated at each time point. A single time control point is acceptable provided an acceptable scientific justification is documented, which shall address the following:

— the control sample;

— the implantation duration;

— animal model;

— study protocol;

— the historical control data.

In cases where the test sample is expected to elicit more than a minimal response, an alternative control using a comparative material with an acceptable response may be selected. The use of a comparative control material shall be justified in terms of material properties and intended use.

D.3.2 Implant size and shape

Implant sizes are based on the animal species and the site chosen. The following typical dimensions shall be considered for rats and rabbits.

Intraparenchymal

— rod or wedge-shaped implants: 1 mm × 1 mm or less in diameter/section and 2 mm to 6 mm in length;

© ISO 2016 – All rights reserved

— an 8 mm diameter disc can be appropriate.

The thickness of the disc shall be justified in relation to the use of the material. For medical devices that are intended to contact primarily the parenchymal surface, the test article shall be implanted on the parenchymal surface.

D.4 Test animals and implant sites

D.4.1 Test animals

This protocol is for studies using rats or rabbits. If other species are used based on device considerations, modifications of the protocol may be necessary. Both sexes should be represented in equal numbers unless sufficient justification for a single sex is provided.

Animals that are healthy and have not been subjected to previous experimental procedures shall be used. Species and age are important factors to consider based on differences in neuro physiology and biological response and should be assessed before implantation procedures are initiated.

D.4.2 Implant sites

There should be a minimum of 8 tests and 8 negative control sites at each time point (split equally between male and female) to permit an evaluation of local neural effects. Equivalent anatomical sites shall be used for test and control samples. Careful selection of the implant site and surgical procedure is critical to minimize the risk of mechanical trauma. Only one hemisphere shall be implanted per animal, and shall include only one type of implant, either test or control. One site per hemisphere can be implanted in each rat, and two sites can be implanted in each rabbit.

With many bone, muscle, and subcutaneous implant designs, test and control materials are typically implanted in the same animal. However, with materials placed in neural tissues, tissue responses are not always localized, but may affect a wide region and may even manifest across the brain hemisphere. In experimental animal models of brain injury, microglia can be induced to migrate along the myelinated sheaths of the corpus callosum to the contralateral hemisphere. Therefore, the implant-activated microglia may migrate across the corpus callosum and influence the biological response on the contralateral side. This could serve to exacerbate the response to a negative control implanted site, resulting in a shift in the baseline response. In addition, influence from the contralateral hemisphere injury could exacerbate the injury response at the test article implanted site. In the first case, one could shift the normal baseline response to a negative control material, resulting in a false-negative result for the test article. In the second case, the response occurring in the contralateral hemisphere could exacerbate the response to a “test article” leading to a false positive. Given the small number of animals required for testing, the ability to control for such factors and to decrease the variability of the data is critical. As such, it is useful to have separate test and control animals. Sex differences have been documented in responses that may be relevant to evaluation of response to implantation devices (see References [43], [44], and [45]). To control for potential variability due to sex differences, similar numbers of males and females of each species and strain should be used for the study.

D.5 Implantation procedure

Each animal should be weighed prior to implantation and periodically thereafter. Following species-appropriate analgesia and anaesthesia, surgically prepare the skull of the animal. Stable analgesia and anaesthesia shall be maintained during the full surgical procedure and for an identified time period after implantation.

Animals should be appropriately restrained during the operation procedure. Using aseptic surgical techniques, the skull is exposed and holes of sufficient similar diameter to insert the implant samples are made in the cranium. In addition, a small hole is prepared in the dura and the implant is gently introduced into the appropriate part of the brain.

© ISO 2016 – All rights reserved

The surgical technique profoundly influences the result of any brain implantation procedure as the severity of injury response (both glial and neuronal) is related to the level of physical trauma and may render a study uninterpretable (see References [45], [46], and [48]).

Stereotactic methods may allow for a significant level of control with regards to accurate placement and minimizing physical damage to the puncture location. An alternative control method for restraining the animals may be considered.

D.6 Implantation period

A one-week implantation period is necessary, as well as other appropriate time intervals, to adequately characterize the response. Neurodegenerative processes can be rapid and transient as cell death can occur in the first few days following administration, as shown with certain chemicals (see Reference [45]).

Longer implantation periods shall be considered in view of the clinical applications of the material.

D.7 Post-implantation observation

Animals should be initially housed individually and observed twice daily to ensure proper healing of the implant sites, return to normal eating and drinking behaviour, and for any abnormal clinical signs due to the surgical procedure. The observation frequency is adjusted based on initial observations. If animals are treated with antibiotics, this needs to be stated as some compounds like minocycline can directly modulate the response of brain microglia and macrophages (see Reference [49]).

As damage to neural tissue can result in abnormal behaviour, clinical observations should be included in the evaluation of the effects of brain implants.

A detailed (weekly) physical examination shall be performed on each animal to monitor general health. Observations shall be recorded and include all abnormal clinical signs, abnormal behaviour, or clinical systemic or central nervous system manifestations of the implant. To help with assessment of central nervous system disorder, a functional observation battery (FOB) or modified Irwin's can be used. Clinical signs could include, but are not limited to, changes in skin, fur, eyes or mucous membranes, and occurrence of secretions and excretions or other evidence of autonomic activity (e.g. lacrimation, piloerection, pupil size, unusual respiratory pattern). Additionally, changes in gait, posture, and response to handling, as well as the presence of clonic or tonic seizures, stereotypes (e.g. excessive grooming, repetitive circling) or bizarre behaviour (e.g. self-mutilating, walking backwards) shall be recorded. For the behaviour and neurological signs, time of first observation and subsequent progression or resolution should be recorded. The initial finding of abnormal behaviour, neurological signs, ambulation, posture, or reflexes shall initiate a daily observation schedule for the relevant signs.

End points for early removal of an animal from the test should be set prior to the test. Once severe clinical effects have been identified, an attending or qualified laboratory animal veterinarian, or personnel trained to identify the clinical lesions, should be consulted for a clinical examination. The attending or qualified laboratory animal veterinarian should determine whether test animals should be removed from the test and euthanized.

D.8 Evaluation of biological response

The evaluation shall take into account the requirements specified in 5.5.

All gross changes observed macroscopically, shall be further evaluated microscopically. Vascular perfusion fixation should be used when possible to reduce immersion fixation artefact in the tissue. Additionally, the cervical (draining) lymph node(s) shall be examined grossly, immersion fixed and examined microscopically. Brain tissues from the animals in the control and treatment groups shall be examined. In addition, tissues from animals which died prematurely or were sacrificed during the study shall be grossly examined and any lesions shall be examined microscopically.

© ISO 2016 – All rights reserved

The neuropathological evaluation should assess the tissue for gliosis and neurodegeneration using appropriate histological stains, biochemical indicators of damage or both. The use of a specific stain/damage indicator should be recorded and supported with appropriate peer-reviewed references describing the stains used in evaluating neurodegeneration or gliosis. The following stains and biomarkers are examples that can be used to assess the histopathologic effect of the implant (see Reference [50]).

Table D.1 — Examples brain biomarkers and stains

Stain and biomarker	Cell type or cellular component evaluated
Hematoxylin and eosin (H&E)	All CNS and lymph node tissue
Fluoro-jade	Degenerating neurons
Autofluorescence	Neurodegeneration
Anti-glial fibrillary acidic protein (GFAP) antibody	GFAP (astrocyte biomarker)
Anti-iba-1 antibody	Ionized calcium binding adaptor molecule 1 (microglia-specific)
Luxol fast blue	Myelin
Amino cupric silver stain	Degenerating neuron

Specific antibodies are not available for all species.

High resolution images from implant sites should be provided that are representative of the pathologist's diagnosis or score observations and can demonstrate the morphological detail of the cellular response specific to the implant.

Specific cellular subpopulations shall be identifiable. Images should include a scale bar indicating magnification.

The study pathologist should define the cellular criteria and characteristics used to identify inflammation. The qualitative and quantitative parameters should be predefined. An example of a scoring system for inflammatory changes in neural tissue is given in Table E.4

Cellular criteria — identify the major cell type(s) or structures involved in the biological response, such as astrocytes, neutrophils, microglia cells, fibroses, and myelin. Identify characteristics of the morphological response of microglia and astrocytes, such as process-bearing, hypertrophy, decrease in ramifications in progression of an amoeboid morphology to identify the stage of the microglia response. Identify the presence of macrophage-like cells. When indicated, use cell-specific staining.

The following characteristics in the tissue surrounding the implant shall be addressed:

a) disruption in the neuronal processes surrounding the implants;

b) zone of astrocytosis and connective tissue around the implant;

c) increase in the number of large blood vessels;

d) infiltrating lymphocytes;

e) microglia activation — in a staging characterization;

f) capsule formation, presence of giant cells and macrophages;

g) areas of mineralization/calcification;

h) changes in the ependymal layer and changes as above in the arachnoid granulations

In addition, it can be useful to examine the brain adjacent to implant track (~3 mm, ipsilateral) and brain away from the implant track.

© ISO 2016 – All rights reserved

Parameters for the brain adjacent to the implant track includes the following:

- inflammatory cell/infiltrates;
- haemorrhage;
- necrosis;
- gliosis, grey matter;
- gliosis, white matter;
- other.

Parameters for the brain away from the implant track include other non-local effects from each animal.

D.9 Test report

The presentation of the test results and final test report shall include the requirements specified in Clause 6 and in addition, the following:

- representative high resolution images of the tissue surrounding the implant site;
- a descriptive narrative of each implant site;
- a semiquantitative scoring.

 © ISO 2016 – All rights reserved

Annex E
(informative)

Examples of evaluation of local biological effects after implantation

E.1 General

For examples of semiquantitative and quantitative scoring systems, see References [17], [18], [20], [25], and [26].

For each histological characteristic evaluated (such as capsule formation, inflammation, presence of polymorphonuclear cells, giant cells, plasma cells, and/or degradation of material), the semiquantitative scoring system used shall be described in the evaluation report. In addition to the scoring of the reaction components, the extent of the whole reaction should also be evaluated.

Some examples of such semiquantitative scoring systems are described below and in References [21], [25], [26], [31], and [40]. The evaluation system as specified in Table E.1 and Table E.2 may be converted to an implant evaluation system as specified in Table E.3. An example of a histological evaluation system for neural tissue responses is provided in Table E.4.

In this semiquantitative scoring scheme, inflammatory cell infiltrates and necrosis are scored using the scoring scheme in Table E.1. Neovascularizaton, fibrosis, and fatty infiltration are scored using the scoring scheme in Table E.2. In the Table E.3 example, due to the greater importance of inflammatory cell infiltrates and necrosis, these parameters (see Table E.1) are multiplied by a factor of 2 to provide a weighted value as compared to neovascularizaton, fibrosis, and fatty infiltration parameters (see Table E.2). The values are totalled, and then an average score for test and control treatments is calculated. The average score for the control treatment is subtracted from the test treatment average to determine a reactivity grade based on the scale in Table E.3.

The study report should comment on each cell type and neovascularization response. For each cell type where there is a significant difference in value between the treatment and control sites, the study report should explain the relevance of the difference.

An example of a scoring system used for biological evaluation of absorbable materials is described in Reference [20].

Table E.1 — An example of a histological evaluation system — Cell type/response

Cell type/response	Score 0	Score 1	Score 2	Score 3	Score 4
Polymorphonuclear cells	0	Rare, 1 to 5/phf[a]	5 to 10/phf	Heavy infiltrate	Packed
Lymphocytes	0	Rare, 1 to 5/phf[a]	5 to 10/phf	Heavy infiltrate	Packed
Plasma cells	0	Rare, 1 to 5/phf[a]	5 to 10/phf	Heavy infiltrate	Packed
Macrophages	0	Rare, 1 to 5/phf[a]	5 to 10/phf	Heavy infiltrate	Packed
Giant cells	0	Rare, 1 to 2/phf	3 to 5/phf	Heavy infiltrate	Sheets
Necrosis	0	Minimal	Mild	Moderate	Severe

[a] phf = per high-powered (400×) field.

© ISO 2016 – All rights reserved

Table E.2 — An example of a histological evaluation system — Tissue response

Response	Score				
	**0**	**1**	**2**	**3**	**4**
Neovascularisation	0	Minimal capillary proliferation, focal, 1 to 3 buds	Groups of 4 to 7 capillaries with supporting fibroblastic structures	Broad band of capillaries with supporting fibroblastic structures	Extensive band of capillaries with supporting fibroblastic structures
Fibrosis	0	Narrow band	Moderately thick band	Thick band	Extensive band
Fatty infiltrate	0	Minimal amount of fat associated with fibrosis	Several layers of fat and fibrosis	Elongated and broad accumulation of fat cells about the implant site	Extensive fat completely surrounding the implant

© ISO 2016 – All rights reserved

Table E.3 — Example of semiquantitative scoring scheme

**Test sample:**	Polymer XYZ					
**Implantation interval:**	2 weeks					
**Control sample:**	HDPE					
**Animal number**	**Test sample**			**Control sample**		
	**1001**	**1002**	**1003**	**1001**	**1002**	**1003**
**F.1 Inflammation**						
Polymorphonuclear cells	2	1	2	1	1	1
Lymphocytes	1	1	0	0	1	0
Plasma cells	0	0	0	0	0	0
Macrophages	2	2	2	1	1	1
Giant cells	1	1	1	0	0	0
Necrosis	0	0	0	0	0	0
**SUB TOTAL (×2)**	12	10	10	4	6	4
**F.2 Neovascularization**	0	0	0	0	0	0
Fibrosis	1	1	1	1	1	1
Fatty infiltrate	0	0	0	0	0	0
**SUB TOTAL**	1	1	1	1	1	1
**TOTAL (F.1 and F.2)**	13	11	11	5	7	5
**GROUP TOTAL**	35			17		
**AVERAGE**[a]	11,7 (–)			5,7 = 6		
Traumatic necrosis	0	0	0	0	0	0
Foreign debris	0	0	0	0	0	0
Number of implants examined[b]	4	4	4	4	4	4

[a] Used to determine reactivity ranking shown below as the conclusion. A negative difference is recorded as zero.

[b] Histological evaluation score represents the averaged score for that animal across the number of implants examined.

NOTE Additional observations can be needed for degradable materials, i.e. extent of degradation.

© ISO 2016 – All rights reserved

E.2 Conclusion

Under the conditions of this study, the test sample was considered to demonstrate the following:

__ minimal or no reaction (0,0 to 2,9);

X slight reaction (3,0 to 8,9);

__ moderate reaction (9,0 to 15,0);

__ severe reaction (15,1)

to the tissue as compared to the negative control sample within Table E.3.

E.3 Comments

Additional comments may be added to provide a description of observations about the tissue response and/or changes to the implant material, if applicable.

Table E.4 — An example of a histological evaluation system — Neural tissue response

<table>
<tr><th rowspan="2">Histologic feature</th><th colspan="5">Score</th></tr>
<tr><th>0</th><th>1</th><th>2</th><th>3</th><th>4</th></tr>
<tr><td>Inflammatory cell type/ response
— Polymorphonuclear cells
— Lymphocytes</td><td rowspan="3">0</td><td rowspan="3">Rare, 1 to 5/hpf[a]</td><td rowspan="3">Rare, 5 to 10/ hpf</td><td rowspan="4">Moderate infiltrate</td><td rowspan="4">Marked infiltrate</td></tr>
<tr><td>Plasma cells</td></tr>
<tr><td>Macrophages/gitter cells</td></tr>
<tr><td>Multinucleated giant cells (MGC)</td><td>0</td><td>Rare, 1 to 2/hpf</td><td>Rare, 3 to 5/hpf</td></tr>
<tr><td>Necrosis</td><td>0</td><td>Minimal</td><td>Mild</td><td>Moderate</td><td>Marked</td></tr>
<tr><td>Neovascularization</td><td>0</td><td>Minimal capillary proliferation, focal, 1 to 3 buds</td><td>Groups of 4 to 7 capillaries with supporting fibroblastic structures</td><td>Broad band of capillaries with supporting fibroblastic structures</td><td>Extensive band of capillaries with supporting fibroblastic structures</td></tr>
<tr><td>Fibrosis</td><td rowspan="2">0</td><td rowspan="2">Narrow band</td><td rowspan="2">Moderately thick band</td><td rowspan="2">Thick band</td><td rowspan="2">Extensive band</td></tr>
<tr><td>Astrocytosis/fatty infiltration</td></tr>
<tr><td colspan="6">[a] hpf = high-powered (400×) field.</td></tr>
</table>

 © ISO 2016 – All rights reserved

Bibliography

[1] ISO 5832-1, *Implants for surgery — Metallic materials — Part 1: Wrought stainless steel*

[2] ISO 5832-2, *Implants for surgery — Metallic materials — Part 2: Unalloyed titanium*

[3] ISO 5832-3, *Implants for surgery — Metallic materials — Part 3: Wrought titanium 6-aluminium 4-vanadium alloy*

[4] ISO 5832-4, *Implants for surgery — Metallic materials — Part 4: Cobalt-chromium-molybdenum casting alloy*

[5] ISO 5832-5, *Implants for surgery — Metallic materials — Part 5: Wrought cobalt-chromium-tungsten-nickel alloy*

[6] ISO 5832-6, *Implants for surgery — Metallic materials — Part 6: Wrought cobalt-nickel-chromium-molybdenum alloy*

[7] ISO 5832-7, *Implants for surgery — Metallic materials — Part 7: Forgeable and cold-formed cobalt-chromium-nickel-molybdenum-iron alloy*

[8] ISO 5832-8, *Implants for surgery — Metallic materials — Part 8: Wrought cobalt-nickel-chromium-molybdenum- tungsten-iron alloy*

[9] ISO 5834-2, *Implants for surgery — Ultra-high-molecular-weight polyethylene — Part 2: Moulded forms*

[10] ISO 6474-1, *Implants for surgery — Ceramic materials — Part 1: Ceramic materials based on high purity alumina*

[11] ISO 6474-2, *Implants for surgery — Ceramic materials — Part 2: Composite materials based on a high-purity alumina matrix with zirconia reinforcement*

[12] ISO 7405, *Dentistry — Evaluation of biocompatibility of medical devices used in dentistry*

[13] ISO 10993-9, *Biological evaluation of medical devices — Part 9: Framework for identification and quantification of potential degradation products*

[14] ASTM F748, *Standard Practice for Selecting Generic Biological Test Methods for Materials and Devices*

[15] ASTM F763, *Standard Practice for Short-Term Screening of Implant Materials*

[16] ASTM F981, *Standard Practice for Assessment of Compatibility of Biomaterials for Surgical Implants with Respect to Effect of Materials on Muscle and Bone*

[17] ASTM F1983, *Standard Practice for Assessment of Selected Tissue Effects of Absorbable Biomaterials for Implant Applications*

[18] MHLW Notification, *Basic Principles of Biological Safety Evaluation Required for Application for Approval to Market Medical Devices*: YAKUSHOKUKI-HATSU 0301 No. 20 (2012.03.01)

[19] Cohen J. Assay of Foreign-Body Reaction. *Journal of Bone and Joint Surgery*. 1959, **41A** pp. 152–166

[20] De Jong W.H., Bergsma J.E., Robinson J.E., Bos R.R.M. Tissue response to partially in vitro predegraded poly-L-lactide implants. *Biomaterials*. 2005, **26** pp. 1781–1791

[21] Wise D.L. et al., edsWallin R.F., & Upman P.J. *Evaluating the Biological Effects of Medical Devices and Materials, Encyclopedic Handbook of Biomaterials and Bioengineering, Part A*. Marcel Dekker Inc, New York, Vol. 1, 1995, pp. 422–4.

© ISO 2016 – All rights reserved

[22] Ferguson A.B., Laing P.G., Hodge E.S. The Ionization of Metal Implants in Living Tissues. *Journal of Bone and Joint Surgery*. 1960, **42A** pp. 77–90

[23] Geret V., Rahn B.A., Mathys R., Straumann F., Perren S.M. In: *In vivo Testing of Tissue Tolerance of Implant Materials: Improved Quantitative Evaluation through Reduction of Relative Motion at the Implant Tissue Interface, from Current Concepts of Internal Fixation of Fracture*. (Uhthoff H.K. ed.). Springer Verlag, 1980

[24] Geret V., Rahn B.A., Mathys R., Straumann F., Perren S.M. Chapter 35: A Method for Testing Tissue Tolerance for Improved Quantitative Evaluation Through Reduction of Relative Motion at the Implant-Tissue Interface. *Evaluation of Biomaterials*. (Winter G.D., Leray J.L., de Groot K. eds.), John Wiley & Sons Ltd., 1980

[25] Ikarashi Y., Toyoda K., Ohsawa N., Uchima T., Tsuchiya T., Kaniwa M. Comparative Studies by Cell Culture and in vivo Implantation Test on the Toxicity of Natural Rubber Latex Materials. *J. Biomed. Mater. Res.* 1992, **26** pp. 339–356

[26] Ikarashi Y., Tsuchiya T., Toyoda K., Kobayashi E., Doi H., Yoneyama T. Tissue Reactions and Sensitivity to Iron Chromium Alloys. *Mater. Trans.* 2002, **43** pp. 3065–3071

[27] Kaminski E.J., Oglesby R.J., Wood N.K., Sandrik J. The Behaviour of Biological Materials at Different Sites of Implantation. *J. Biomed. Mater. Res.* 1968, **2** pp. 81–88

[28] Laing P.G., Ferguson A.B., Hodge E.S. Tissue Reaction in Rabbit Muscle Exposed to Metallic Implants. *J. Biomed. Mater. Res.* 1967, **1** pp. 135–149

[29] Langeland K., Guttuso J., Langeland L.L., Tobon G. Methods in the study of biological response to endodontic materials. Tissue response to N2 (root canal sealer). *Oral Surg. Oral Med. Oral Pathol.* 1969, **27** pp. 522–542

[30] Preul M.C., Bichard W.D., Muench T., Spetzler R.F. Toward optimal tissue sealants for neurosurgery: use of a novel hydrogel in a canine durotomy repair model. *Neurosurgery*. 2003, **53** pp. 1189–1198

[31] Rahn B.A., Geret V., Capaul C., Lardi M., Solothurnmann B Morphometric Evaluation of Tissue Reaction to Implants Using Low Cost Digitizing Techniques, *Clinical Applications of Biomaterials*, (Lee A.J.C., Albrektsson T., Branemark P.I. eds.), John Wiley & Sons Ltd., 1982

[32] Tilney N.L. Patterns of lymphatic drainage in the adult laboratory rat. *J. Anat.* 1971, **109** (3) pp. 369–383

[33] Torneck C.D. Reaction of Rat Connective Tissue to Polyethylene Tube Implants: Part I. *Oral Surg. Oral Med. Oral Pathol.* 1966, **21** pp. 379–387

[34] Torneck C.D. Reaction of Rat Connective Tissue to Polyethylene Tube Implants: Part II. *Oral Surg. Oral Med. Oral Pathol.* 1967, **24** pp. 674–683

[35] Turner E., Lawrence W.H., Autian J. Subacute Toxicity Testing of Biomaterials Using Histological Evaluation of Rabbit Muscle Tissue. *J. Biomed. Mater. Res.* 1973, **7** pp. 39–58

[36] Upman P.J. Toxicity Testing (of medical devices), *Handbook of Biomaterials Evaluation*, A. Von Recum (ed.), 2nd ed., Taylor & Francis, 1998, pp. 285-286

[37] Upman P.J., & Muench T. Comprehensive Histopathology Scoring System for Biomaterial Implants, Am. College of Toxicology meeting, Palm Springs, CA, Nov 7-10, 2004; Abstract published. *Int. J. Toxicol.* 2004, **23** p. 384

[38] US FDA-CDRH. Guidance document for testing biodegradable polymer implant devices (DRAFT); available under: www.fda.gov/MedicalDevices/DeviceRegulationandGuidance/GuidanceDocuments/ucm080265.htm

[39] U.S. Pharmacopeia, Biological reactivity tests, in-vivo

 © ISO 2016 – All rights reserved

[40] Pizzoferrato A., Savarino L., Stea S., Tarabusi C. Result of Histological Grading on 100 cases of Hip Prosthesis Failure. *Biomaterials*. 1988, **9** pp. 314–318

[41] Yamada T., Nakaoka R., Sawada R., Matsuoka A., Tsuchiya T. Effects of Intracerebral Microinjection of Hydroxylated –[60]Fullerence on Brain Monoamine Concentrations and Locomotor Behaviour in Rats. *J. Nanotechnol.* 2009, **9** pp. 1–8

[42] Fanning N.F., Willinsky R.A., ter Brugge K.G. Wall Enhancement, Edema, and Hydrocephalus After Endovascular Coil Occlusion of Intradural Cerebral Aneurysms. *J. Neurosurg.* 2008, **108** (6) pp. 1074–1086

[43] Wiesenfeld-Hallin Z. Sex differences in pain perception. *Gend. Med.* 2005, **2** (3) pp. 137–145

[44] Exploring the Biological Contribution to Human Health. In: *Does Sex Matter*Wizemann T.M., & Pardue M.L. eds.). Institute of Medicine, National Academy Press, 2001

[45] Carmichael N.M., Charlton M.P., Dostrovsky J.O. Sex Differences in Inflammation Evoked by Noxious Chemical, Heat and Electrical Stimulation. *Brain Res.* 2009, **1276** pp. 103–111

[46] Bolon B. “Current Pathology Techniques” Symposium Review: Advances and Issues in Neuropathology. *Toxicol. Pathol.* 2008, **36** pp. 871–889

[47] ISO 11979-5, *Ophthalmic implants — Intraocular lenses — Part 5: Biocompatibility*

[48] Polikov V.S., Tresco P.A., Reichert W.M. Response of Brain Tissue to Chronically Implanted Neural Electrodes. *J. Neurosci. Methods*. 2005, **148** pp. 1–18

[49] Tikka T., Fiebich B.L., Goldsteins G., Keinanen R., Koistinaho J. Minocycline, a Tetracycline Derivative, is Neuroprotective Against Excitotoxicity by Inhibiting Activation and Proliferation of Microglia. *J. Neurosci.* 2001, **15** pp. 2580–2588

[50] Jordan W.H., Young J.K., Hyten M.J., Hall D.G. Preparation and analysis of the central nervous system. *Toxicol. Pathol.* 2011, **39** pp. 58–65

[51] Therin M., Christel P., Meunier A. Analysis of the general features of the soft tissue response to some metals and ceramics using quantitative histomorphometry. *J. Biomed. Mater. Res.* 1994, **28** (11) pp. 1267–1276

[52] ISO 10993-11, *Biological evaluation of medical devices — Part 11: Tests for systemic toxicity*

© ISO 2016 – All rights reserved

ICS 11.100.20
Price based on 29 pages

© ISO 2016 – All rights reserved
