# Supplementary material for: Endocytosis-mediated healing: recombinant human collagen type III chain-induced wound healing for scar-free recovery
Source: Regen Biomater. 2025 Jan 8;12:rbae149. doi: 10.1093/rb/rbae149 (PMC11930350; doi:10.1093/rb/rbae149)
Supplement: rbae149_Supplementary_Data [file rbae149_supplementary_data.zip › 48869_Supplementary 1-4.ISO 10993-11-2017.pdf]

INTERNATIONAL STANDARD

ISO 10993-11

Third edition
2017-09

Biological evaluation of medical devices —

Part 11:
Tests for systemic toxicity

Évaluation biologique des dispositifs médicaux —

Partie 11: Essais de toxicité systémique

Reference number
ISO 10993-11:2017(E)

© ISO 2017

COPYRIGHT PROTECTED DOCUMENT

© ISO 2017, Published in Switzerland

All rights reserved. Unless otherwise specified, no part of this publication may be reproduced or utilized otherwise in any form or by any means, electronic or mechanical, including photocopying, or posting on the internet or an intranet, without prior written permission. Permission can be requested from either ISO at the address below or ISO's member body in the country of the requester.

ISO copyright office
Ch. de Blandonnet 8 • CP 401
CH-1214 Vernier, Geneva, Switzerland
Tel. +41 22 749 01 11
Fax +41 22 749 09 47
copyright@iso.org
www.iso.org

Contents

Page

Foreword ..... **v**
Introduction ..... **vi**
1 Scope **1**
2 Normative references **1**
3 Terms and definitions **1**
4 General considerations **3**
4.1 General 3
4.2 Selection of animal species 3
4.3 Animal status 3
4.4 Animal care and husbandry 3
4.5 Size and number of groups 4
4.5.1 Size of groups 4
4.5.2 Number of groups 4
4.5.3 Treatment controls 4
4.6 Route of exposure 5
4.7 Sample preparation 5
4.8 Dosing 5
4.8.1 Test sample administration 5
4.8.2 Dosage volumes 5
4.8.3 Dosage frequency 6
4.9 Body weight and food/water consumption 6
4.10 Clinical observations 6
4.11 Clinical pathology 6
4.12 Anatomic pathology 7
4.13 Study designs 7
4.14 Quality of investigation 7
5 Acute systemic toxicity **7**
5.1 General 7
5.2 Study design 8
5.2.1 Preparations 8
5.2.2 Experimental animals 8
5.2.3 Test conditions 8
5.2.4 Body weights 9
5.2.5 Clinical observations 9
5.2.6 Pathology 9
5.3 Evaluation criteria 10
5.3.1 General 10
5.3.2 Evaluation of results 10
5.4 Final report 10
6 Repeated exposure systemic toxicity (subacute, subchronic and chronic systemic toxicity) **12**
6.1 General 12
6.2 Study design 12
6.2.1 Preparations 12
6.2.2 Experimental animals 12
6.2.3 Test conditions 13
6.2.4 Body weights 13
6.2.5 Clinical observations 13
6.2.6 Pathology 13
6.3 Evaluation criteria 14
6.3.1 General 14
6.3.2 Evaluation of results 14

© ISO 2017 – All rights reserved

6.4 Final report 15
Annex A (informative) **Routes of administration** 16
Annex B (informative) **Dosage volumes** 18
Annex C (informative) **Common clinical signs and observations** 19
Annex D (informative) **Suggested haematology, clinical chemistry and urinalysis measurements** 20
Annex E (informative) **Suggested organ list for histopathological evaluation** 22
Annex F (informative) **Organ list for limited histopathology for medical devices subjected to systemic toxicity testing** 24
Annex G (informative) **Information on material-mediated pyrogens** 25
Annex H (informative) **Subchronic rat — Dual routes of parenteral administration** 26
Bibliography 28

 © ISO 2017 – All rights reserved

Foreword

ISO (the International Organization for Standardization) is a worldwide federation of national standards bodies (ISO member bodies). The work of preparing International Standards is normally carried out through ISO technical committees. Each member body interested in a subject for which a technical committee has been established has the right to be represented on that committee. International organizations, governmental and non-governmental, in liaison with ISO, also take part in the work. ISO collaborates closely with the International Electrotechnical Commission (IEC) on all matters of electrotechnical standardization.

The procedures used to develop this document and those intended for its further maintenance are described in the ISO/IEC Directives, Part 1. In particular the different approval criteria needed for the different types of ISO documents should be noted. This document was drafted in accordance with the editorial rules of the ISO/IEC Directives, Part 2 (see www.iso.org/directives).

Attention is drawn to the possibility that some of the elements of this document may be the subject of patent rights. ISO shall not be held responsible for identifying any or all such patent rights. Details of any patent rights identified during the development of the document will be in the Introduction and/or on the ISO list of patent declarations received (see www.iso.org/patents).

Any trade name used in this document is information given for the convenience of users and does not constitute an endorsement.

For an explanation on the voluntary nature of standards, the meaning of ISO specific terms and expressions related to conformity assessment, as well as information about ISO's adherence to the World Trade Organization (WTO) principles in the Technical Barriers to Trade (TBT) see the following URL: www.iso.org/iso/foreword.html.

This document was prepared by Technical Committee ISO/TC 194 *Biological and clinical evaluation of medical devices*.

This third edition cancels and replaces the second edition (ISO 10993-11:2006), which has been technically revised with the following changes:

a) reduction in group size for chronic toxicity testing in Table 1;

b) a new Annex F was added;

c) the original Annex F was moved to Annex G;

d) a new Annex H was added;

e) the Bibliography was updated.

A list of all parts in the ISO 10993 series can be found on the ISO website.

© ISO 2017 – All rights reserved

Introduction

Systemic toxicity is a potential adverse effect of the use of medical devices. Generalized effects, as well as organ and organ system effects can result from absorption, distribution and metabolism of leachates from the device or its materials to parts of the body with which they are not in direct contact. This document addresses the evaluation of generalized systemic toxicity, not specific target organ or organ system toxicity, even though these effects may result from the systemic absorption and distribution of toxicants.

Because of the broad range of medical devices, and their materials and intended uses, this document is not overly prescriptive. While it addresses specific methodological aspects to be considered in the design of systemic toxicity tests, proper study design has to be uniquely tailored to the nature of the device's materials and its intended clinical application.

Other elements of this document are prescriptive in nature, including those aspects that address compliance with good laboratory practices and elements for inclusion in reporting.

While some systemic toxicity tests (e.g. long term implantation or dermal toxicity studies) can be designed to study systemic effects as well as local, carcinogenic or reproductive effects, this document focuses only on those aspects of such studies, which are intended to address systemic effects. Studies which are intended to address other toxicological end points are addressed in ISO 10993-3, ISO 10993-6, ISO 10993-10 and ISO/TS 10993-20.

Prior to conducting a systemic toxicity study, all reasonably available data and scientifically sound methods in the planning and refinement of the systemic toxicity study design should be reviewed. This includes the suitability of use of input data such as existing toxicological data, data from chemical characterization studies and/or other biological tests (including *in vitro* tests and less invasive *in vivo* tests) for the refinement of study design, dose selection, and/or selection of pathological end points to cover in the evaluation of a study. For the repeated exposure systemic toxicity study in particular, the use of scientifically sound study design, the use of pilot studies and statistical study design and the use of unbiased, quantitative end points/methods in the pathological (including histopathological) and clinical chemistry methods are important so as to obtain data which have sufficient scientific validity.

Finally, toxicology is an imperfect science. The outcome of any single test should not be the sole basis for making a determination of whether a device is safe for its intended use.

© ISO 2017 – All rights reserved

Biological evaluation of medical devices —

Part 11:
Tests for systemic toxicity

1 Scope

This document specifies requirements and gives guidance on procedures to be followed in the evaluation of the potential for medical device materials to cause adverse systemic reactions.

2 Normative references

The following documents are referred to in the text in such a way that some or all of their content constitutes requirements of this document. For dated references, only the edition cited applies. For undated references, the latest edition of the referenced document (including any amendments) applies.

ISO 10993-1, *Biological evaluation of medical devices — Part 1: Evaluation and testing within a risk management process*

ISO 10993-2, *Biological evaluation of medical devices — Part 2: Animal welfare requirements*

3 Terms and definitions

For the purposes of this document, the terms and definitions given in ISO 10993-1 and the following apply.

ISO and IEC maintain terminological databases for use in standardization at the following addresses:

— IEC Electropedia: available at http://www.electropedia.org/

— ISO Online browsing platform: available at http://www.iso.org/obp

3.1
dose
dosage
amount of test sample administered (e.g. mass, volume) expressed per unit of body weight or surface area

3.2
dose-effect
relationship between the dosage and the magnitude of a defined biological effect either in an individual or in a population sample

3.3
dose-response
relationship of dosage to the spectrum of effects related to the exposure

Note 1 to entry: There are two types of dose-response relationships. The first type is the response of an individual to a range of doses. The second type is the distribution of responses of a population of individuals to a range of doses.

© ISO 2017 – All rights reserved

3.4
leachable substance
chemical removed from a device or material by the action of water or other liquids related to the use of the device

Note 1 to entry: Examples of leachable substances are additives, sterilant residues, process residues, degradation products, solvents, plasticizers, lubricants, catalysts, stabilizers, anti-oxidants, colouring agents, fillers and monomers.

3.5
limit test
use of a single group treated at a suitable dosage of test sample in order to delineate the presence or absence of a toxic hazard

3.6
systemic toxicity
toxicity that is not limited to adverse effects at the site of contact between the body and the device

Note 1 to entry: Systemic toxicity requires absorption and distribution of a toxicant from its entry point to a distant site at which deleterious effects are produced.

3.7
acute systemic toxicity
adverse effects occurring at any time within 72 h after single, multiple or continuous exposures of a test sample for 24 h

3.8
subacute systemic toxicity
adverse effects occurring after multiple or continuous exposure between 24 h and 28 d

Note 1 to entry: Since this term is semantically incorrect, the adverse effects occurring within the specified time period may also be described as a short-term repeated exposure systemic toxicity study. The selection of time intervals between 14 d and 28 d is consistent with most international regulatory guidelines and considered a reasonable approach. Subacute intravenous studies are generally defined as treatment durations of >24 h but <14 d.

3.9
subchronic systemic toxicity
adverse effects occurring after the repeated or continuous administration of a test sample for a part of the lifespan

Note 1 to entry: Subchronic toxicity studies are usually 90 d in rodents but not exceeding 10 % of the lifespan of other species. Subchronic intravenous studies are generally defined as treatment durations of 14 d to 28 d for rodents and non-rodents, respectively.

3.10
chronic systemic toxicity
adverse effects occurring after the repeated or continuous administration of a test sample for a major part of the life span

Note 1 to entry: Chronic toxicity studies usually have a duration of 6 months to 12 months.

3.11
test sample
material, device, device portion, component, extract or portion thereof that is subjected to biological or chemical testing or evaluation

© ISO 2017 – All rights reserved

4 General considerations

4.1 General

Before a decision to perform a systemic toxicity test is made, ISO 10993-1 shall be taken into account. The decision to perform a test shall be justified on the basis of an assessment of the risk of systemic toxicity. Selection of the appropriate test(s) for a device shall be in accordance with ISO 10993-1, giving due consideration to mode and duration of contact.

Testing shall be performed on the final product and/or representative component samples of the final product and/or materials. Test samples shall reflect the conditions under which the device is normally manufactured and processed. If deviations are necessary, they shall be recorded in the test report, together with their justification. For hazard identification purposes, it may be necessary to exaggerate exposure to the test samples.

Physical and chemical properties of the test sample including, for example, pH, stability, viscosity, osmolality, buffering capacity, solubility and sterility, are some factors to consider when designing the study.

When animal tests are considered, all reasonably and practically available replacement, reduction and refinement alternatives should be identified and implemented to satisfy the provisions of ISO 10993-2. For *in vivo* acute toxicity testing, *in vitro* cytotoxicity data are useful in estimating starting doses.

4.2 Selection of animal species

There is no absolute criterion for selecting a particular animal species for systemic toxicity testing of medical devices. However, the species used shall be scientifically justified and in line with the provisions of ISO 10993-2. For acute oral, intravenous, dermal and inhalation studies of medical devices the rodent (mouse or rat) is preferred with the option of the rabbit (lagomorph) in the case of dermal and implantation studies. Other non-rodent species may also need to be considered for testing, recognizing that a number of factors might dictate the number or choice of species for study.

It is preferred that a single animal species and strain are used when a series of systemic toxicity studies of different durations are performed, e.g. acute, subacute, subchronic and/or chronic systemic toxicity. This controls the variability between species and strains and facilitates an evaluation related solely to study duration. Should multiple species or strains be used, justification for their selection shall be documented.

4.3 Animal status

Generally, healthy purpose-bred young adult animals of known origin and with defined microbiological health status should be used. At the commencement of the study, the weight variation of animals used within a sex should not exceed ±20 % of the mean weight. When females are used, they should be nulliparous and non-pregnant. Animal selection shall be justified.

4.4 Animal care and husbandry

Care and handling of animals shall conform to accepted animal husbandry guidelines. Animals shall be acclimatized to the laboratory conditions prior to treatment and the period of time documented. Control of environmental conditions and proper animal care techniques are necessary for meaningful results. Dietary constituents and bedding materials that are known to produce or influence toxicity should be properly characterized and their potential to influence test results taken into account.

© ISO 2017 – All rights reserved

4.5 Size and number of groups

4.5.1 Size of groups

The precision of the systemic toxicity test is dependent to a large extent on the number of animals used per dose level. The degree of precision needed and, in turn, the number of animals per dose group needed depends on the purpose of the study.

Group sizes should logically increase with the duration of treatment, such that at the end of the study enough animals in every group are available for thorough biological evaluation. However, the minimum number of animals should be used consistent with obtaining meaningful results (see ISO 10993-2). Recommended minimum group sizes, all routes considered, are given in Table 1.

Table 1 — Recommended minimum group sizes

Study type	Rodent	Non-rodent
Acute[a]	5	3
Subacute	10 (5 per sex)[a]	6 (3 per sex)[a]
Subchronic	20 (10 per sex)[a]	8 (4 per sex)[a]
Chronic	30 (15 per sex)[b, c]	[c]

[a] Testing in a single sex is acceptable. When a device is intended for use in only one sex, testing should be done in that sex.

[b] The recommendation for rodents refers to one dose-level group testing. Where additional exaggerated dose groups are included the recommended group size may be reduced to 10 per sex.

[c] Expert statistical consultation for chronic study non-rodent group size is recommended. The number of animals tested should be based on the minimum required to provide meaningful data. Enough animals shall remain at the termination of the study to ensure proper statistical evaluation of the results.

4.5.2 Number of groups

One dose group treated at a suitable dosage of test sample in a single species could delineate the presence or absence of a toxic hazard (i.e. limit test). However, other multi-dose or dose response studies require multiple groups to delineate the toxic response.

The number of treatment groups may be increased when attempting to characterize a dose response using exaggerated doses. The following examples for exaggerating the dose should be considered:

— multiples of the clinical surface area of exposure;

— multiples of the duration of exposure;

— multiples of the extractable fraction or the individual chemicals;

— multiple administrations within a 24-h period.

Other methods to exaggerate the dose may be acceptable. The method used shall be justified.

4.5.3 Treatment controls

Depending on the objective of the study, the nature of the test article and the route of exposure, negative, vehicle and/or sham-treated controls should be incorporated into all systemic toxicity studies. These controls shall mimic the test sample preparation and treatment procedure.

© ISO 2017 – All rights reserved

4.6 Route of exposure

Medical devices or their leachable substances may gain access to the body by multiple routes of exposure. The test route of exposure shall be the most clinically relevant to the use of the device, where possible. If an alternative route of exposure is necessary, it shall be justified. Examples of routes of administration can be found in Annex A.

4.7 Sample preparation

Guidance on sample preparation and stability is given in ISO 10993-12.

4.8 Dosing

4.8.1 Test sample administration

Procedures should be designed to avoid physiological changes or animal welfare problems not directly related to the toxicity of the test material. If a single daily dose of a sufficient volume or concentration is not possible, the dose may be given in smaller fractions over a period not exceeding 24 h.

Test samples shall be delivered at a physiologically acceptable temperature. In general, room or body temperature is a common practice. Deviations shall be justified.

Vehicles administered by a parenteral route should be physiologically compatible. When necessary, sample filtration to remove particulates should be used and documented. When medical devices and/or test samples in the form of nanomaterials are to be evaluated sample filtrations shall not be performed. (see ISO/TR 10993-22).

Restraint of animals in repeated exposure systemic toxicity studies should generally be limited to between 4 h and 6 h per day. The nature and the duration of restraint should be the minimum required to meet the scientific objectives and should not of themselves compromise the welfare of the test animals. Deviations shall be justified.

When restraint is required animals should be acclimatized to the restraint device prior to test sample administration.

4.8.2 Dosage volumes

Guidance on dosage volume is summarized in Annex B. When multiple dosage groups are used, variability in the test volume may be minimized by adjusting the concentration to ensure a constant volume at all doses. Use of dosage volumes greater than those given in Annex B shall be justified.

Large dose volumes administered by the oral route should be avoided because they have been shown to overload the stomach capacity and pass immediately into the small bowel. Large volumes may also reflux into the oesophagus.

Intramuscular administration is also volume-limited, depending on size of the animal and the muscular site. Species-specific intramuscular administration volumes are addressed in Annex B.

Bolus intravenous injection volumes are usually given over a period of approximately 1 min. The rate of injection is an important factor and it is suggested that, for rodents, the rate shall not exceed 2 ml/min.

Slow or timed injection, or intravenous infusion, may be required for large volume administration. Regardless of the calculated rate, the rate of fluid administration shall be stopped or decreased if the animal demonstrates a marked change in clinical condition.

Slow intravenous injection rates may be necessary for test samples limited by solubility or irritancy.

Continuous infusion may be used if clinically indicated. The volume and rate of administration will depend on the substance being given and take into account standard fluid therapy practice. As a guide,

© ISO 2017 – All rights reserved

the volume administered on a single occasion will be <10 % of the circulating blood volume over 2 h. Minimal effective restraint of test animals is a key factor to be considered for prolonged infusion.

For subcutaneous administration of test article, refer to Annex B. The rate and extent of absorption depends on the test sample formulation.

4.8.3 Dosage frequency

The dosage frequency should be based on clinical relevancy. Exaggerated procedures shall be clearly specified and justified.

In acute systemic toxicity studies, the animals should be exposed to the test sample in a single dose or with multiple fractions of the dose given within a 24 h period.

In repeated exposure studies the animals should be dosed with the test sample daily, seven days each week for the duration of the test. Other dosage regimens may be acceptable but shall be justified.

4.9 Body weight and food/water consumption

Body weight change and changes in food and water consumption may be attributed to the effects of a test article. Consequently, individual weights of the animals shall be determined shortly before the test sample is administered (e.g. usually within 24 h for single or acute dosing, and no more than 7 d for repeated exposure studies), at regular intervals throughout the study and at study termination. When dosing by body weight, the most recent body weight should be utilized.

Measurements of food and water consumption, as appropriate, shall be considered for longer-term repeated exposure studies.

4.10 Clinical observations

Clinical observations should be performed by trained individuals to ensure consistent reporting. The frequency and duration of observation should be determined by the nature and severity of the toxic reactions, rate of onset and recovery period. Increased frequency of observation may be necessary in the early phase of a study, especially acute studies. The time at which signs of toxicity appear and disappear, their duration and the time of death are important, especially if there is a tendency for adverse clinical signs or deaths to be delayed. Humane end points, as defined by national or international animal welfare guidelines, should be used in order to avoid unnecessary suffering. General clinical observations shall consider the peak period of anticipated effects after dosing.

Observations shall be recorded systematically as they are made. Records shall be maintained for each animal.

Cage-side observations for viability or overt clinical signs shall be recorded at least once each day using common laboratory descriptors of clinical effects (see Annex C).

Morbidity and mortality observations shall be recorded at least twice daily for long-term repeated exposure studies. A more extensive screening for adverse clinical signs may be considered on at least a weekly basis for longer-term repeated exposure studies.

4.11 Clinical pathology

Haematology and clinical chemistry analyses are performed to investigate toxic effects in tissues, organs and other systems. When indicated, these analyses shall be performed on blood samples obtained from repeated exposure study animals at least just prior to, or as a part of, the procedure for scheduled animal termination. Fasting of animals prior to blood sampling may be necessary in some cases. When scientifically indicated, urinalysis can be performed during the last week of a long-term repeated exposure study using timed (e.g. 16 h to 24 h) urine volume collection.

© ISO 2017 – All rights reserved

Suggested haematology, clinical chemistry and urinalysis parameters for evaluation are listed in Annex D.

4.12 Anatomic pathology

When clinically indicated, gross pathological evaluations should be considered for acute systemic toxicity studies.

All animals in repeated exposure studies shall be subjected to a full, detailed gross necropsy which includes careful examination of the external surface of the body, all orifices, and the cranial, thoracic, and abdominal cavities and their contents. Selected organs for weighing should be trimmed of any adherent tissue, as appropriate, and their wet weight taken as soon as possible to avoid drying.

Annex E suggests the tissues that should be weighed and preserved in an appropriate fixation medium for histopathological examination.

A summary of minimum observations for each type of study is given in Table 2.

Table 2 — Summary of observations

Observation	Acute	Subacute/subchronic	Chronic [a]
Body weight change	+	+	+
Clinical observations	+	+	+
Clinical pathology	b	a, b	+
Gross pathology	b	+	+
Organ weights	b	+	+
Histopathology	b	a, b	+

\+ Data should be provided.

[a] Chronic systemic toxicity testing is generally a time extension of subacute/subchronic testing, justified by the human exposure period. Many of the same parameters are recorded and reported. Group sizes may be increased to include satellite groups for which some, or all, of these observations may be made.

[b] Consideration should be given to these measurements when clinically indicated or if longer exposure testing is not anticipated. Lists of suggested bodily fluids and organ/tissue analyses are included in Annex D, Annex E and Annex F.

4.13 Study designs

Study designs are listed in subsequent clauses of this document. Expert consultation for study design is recommended.

4.14 Quality of investigation

Good laboratory practices deal with the organization, process and conditions under which laboratory studies are planned, performed, monitored, recorded and reported. These practices are intended to promote the quality and validity of the test data. They also support the global harmonization effort by facilitating the memoranda of understanding between trading nations. Systemic toxicity studies shall be conducted following such principles.

5 Acute systemic toxicity

5.1 General

Acute systemic toxicity provides general information on health hazards likely to arise from an acute exposure by the intended clinical route. An acute toxicity study might be an initial step in establishing a dosage regimen in subacute/subchronic and other studies and may provide information on the mode of toxic action of a substance by the intended clinical exposure route. Subsequent to test sample

© ISO 2017 – All rights reserved

administration in acute systemic toxicity testing, observations are made of effects (e.g. adverse clinical signs, body weight change, gross pathological findings) and deaths. Animals showing severe and enduring signs of distress and pain need to be euthanized immediately. Corrosive or irritating materials known to cause marked pain or distress should be reported as such and need not be tested.

The Interagency Coordinating Committee on the Validation of Alternative Methods (ICCVAM) and the European Centre for the Validation of Alternative Methods (ECVAM) have validated the *in vitro* cytotoxicity test as an alternative to acute oral toxicity testing. Humane end points, as defined by national or international animal welfare guidelines, should be used in order to avoid unnecessary suffering.

5.2 Study design

5.2.1 Preparations

Healthy young adult animals are acclimatized to the laboratory conditions for at least 5 d prior to the test. Shorter durations shall be justified. Animals are then randomized and assigned to the treatment groups.

5.2.2 Experimental animals

5.2.2.1 Selection of species

Typically, a rodent species (rat, mouse) will be used. Characteristics of the model (age, weight, etc.) are as specified in 4.2 and 4.3. If non-rodent species are used their use shall be scientifically justified.

5.2.2.2 Number and sex

The number and type of group, animals per group, and sex are as specified in 4.5.

5.2.2.3 Housing and feeding conditions

The temperature and the relative humidity in the experimental animal rooms should be appropriate for the species, e.g. (22 ± 3) °C and 30 % to 70 % RH, for mice. Typically, the artificial lighting sequence should be 12 h light, 12 h dark.

For feeding, standardized commercial laboratory diets may be used with an unlimited supply of drinking water. Animals should be caged in-groups by sex or individually, as appropriate; for group housing not more than five animals shall be housed per cage.

5.2.3 Test conditions

5.2.3.1 Dose levels

Dose levels shall be as specified in 4.8.

Animals in the control group should be handled in an identical manner to the test group subjects with the exception of not being dosed with the test sample.

5.2.3.2 Procedure

The animals receive a single dose of the test sample or, when necessary, multiple doses within a single 24 h period. Signs of toxicity should be recorded as they are observed including the time of onset, degree and duration.

Regular observation of the animals is necessary to ensure that animals are not lost from the study due to cannibalism, autolysis of tissues or misplacement. At the end of the study, all surviving animals are euthanized. Any moribund animals should be removed and euthanized when noticed to exhibit such

© ISO 2017 – All rights reserved

behaviour. Methods used for euthanasia should be in accordance with national or international animal welfare guidelines.

The observation schedules and humane end points applied should preclude the possibility of animals being found dead as a direct consequence of test sample toxicity.

5.2.4 Body weights

Body weight measurements should be made immediately before dosing, daily for the first three days after dosing, weekly after the first dose if indicated by study duration, and at the end of the study.

5.2.5 Clinical observations

The observation period for an acute systemic toxicity study shall be at least 3 d, or longer when deemed appropriate. Specifics of frequency and observation type are specified in 4.10 and Annex C. In all cases, observations shall be made at a frequency, and appropriate actions taken, to minimize the loss of animals to the study, e.g. necropsy or refrigeration of those animals found dead and isolation or sacrifice of weak or moribund animals. Cage-side observations should include, but not be limited to, changes in skin and fur, eyes and mucous membranes, and also respiratory, circulatory, autonomic and central nervous system, somatomotor activity and behaviour pattern, using the descriptors provided in Annex C.

5.2.6 Pathology

5.2.6.1 Clinical pathology

Clinical pathology evaluations shall be considered when there is an indication, such as for device materials with expected or observed toxicity (from a prior study), or for new device materials where there is no previous experience. When clinical pathology evaluations are performed, the following examinations shall be considered.

a) Haematology, as specified in Annex D, should be considered for investigation at the end of the test period.

b) Clinical biochemical determination on blood, as listed in Annex D, should be considered at the end of the test period. Test areas which are considered appropriate to acute exposure studies are liver and kidney function. Additional clinical biochemistry may be utilized where necessary to extend the observation of the observed effects.

Urinalysis (see Annex D) is not necessary on a routine basis but only when there is an indication based on expected or observed toxicity. Suggested parameters are listed in Annex D.

5.2.6.2 Gross pathology

Gross pathological evaluations shall be considered when there is an indication, such as for device materials with expected or observed toxicity (from a prior study), or for new device materials where there is no previous experience. This should include an examination of the external surface of the body, all orifices, and the cranial, thoracic and abdominal cavities and their contents. When appropriate, consideration should also be given to recording the weight of the brain, liver, kidneys, adrenals and testes, which should be weighed wet as soon as possible after dissection to avoid drying and subsequent falsely low values.

5.2.6.3 Histopathology

Full histopathology is not typically carried out on organs and tissues from animals in the acute systemic toxicity study, unless indicated specifically by unique gross necropsy findings.

© ISO 2017 – All rights reserved

5.3 Evaluation criteria

5.3.1 General

Depending on the test design utilized, the following evaluation criteria apply.

a) For pharmacopoeia-type testing.

  1) If during the observation period of an acute systemic toxicity test none of the animals treated with the test sample shows a significantly greater biological reactivity than animals treated with the vehicle control, the sample meets the requirements of this test.

  2) Using five animals, if two or more animals die, or if behaviour such as convulsions or prostration occurs in two or more animals, or if a final (end of study) body weight loss greater than 10 % occurs in three or more animals, the sample does not meet the requirements of the test. Any transitory body weight loss should be critically evaluated along with other clinical observations in the assessment of systemic toxicity.

  3) If any animals treated with the sample show only slight signs of biological reactivity, and not more than one animal shows gross symptoms of biological reactivity or dies, repeat the testing using groups of 10 animals.

  4) On the repeat test, if all 10 animals treated with the sample show no scientifically meaningful biological reactivity above the vehicle control animals during the observation period, the sample meets the requirements of this test.

b) For non-pharmacopoeia acute systemic toxicity tests.

  The option exists to perform evaluations using more extensive methods including clinical and anatomic pathology, which may eliminate the need for a repeat test. Acute exposure may include a re-evaluation if there are equivocal differences from concurrent controls. Differences should be explained and the study extended to include an additional five animals, if applicable.

5.3.2 Evaluation of results

The findings of an acute systemic toxicity study should be evaluated in conjunction with the findings of preceding studies, if available, and considered in terms of the toxic effects and the gross necropsy findings, if observed. The evaluation shall include the relationship between the dose of the test substance and the presence or absence and the incidence and severity of abnormalities, including behavioural and clinical abnormalities, gross lesions, body weight changes, effects on mortality and any other general or specific effects.

5.4 Final report

The following information, where applicable, shall be contained in the final test report for the acute systemic toxicity study.

a) Details of the testing laboratory and study sponsor, and rationale for selection of the study design.

b) Test sample:

  1) physical nature, purity and physiochemical properties, as appropriate;

 © ISO 2017 – All rights reserved

2) other identification data.

c) Extraction solvent or vehicle (if appropriate):

1) justification for choice of extraction solvent or vehicle if other than those listed in ISO 10993-12.

d) Test animals:

1) species/strain used;

2) number, age and sex of animals;

3) source including microbiological status (e.g. barrier raised, conventional), housing conditions (temperature, humidity, bedding, lighting, diet, etc.);

4) weights at study initiation.

e) Test conditions:

1) rationale for dose selection;

2) details of test sample formulation/preparation; achieved concentrations; stability and homogeneity, if appropriate;

3) details of the administration of the test sample;

4) conversion from test sample concentration (ppm) to the actual dose (mg/kg BW), if applicable;

5) details of food, water and bedding quality.

f) Results:

1) data may be summarized in tabular form, showing for each control and test group the number of animals at the start of the test, the number of animals showing adverse clinical signs, and the number of animals displaying body weight changes;

2) body weight/body weight change;

3) food and water consumption, if applicable;

4) toxic response data by sex and dose level, including signs of toxicity;

5) nature, severity and duration of clinical observations (whether reversible or not);

6) neurobehavioural assessments, if applicable;

7) haematological tests utilized and results with relevant control data, if applicable;

8) clinical biochemistry tests utilized and results with relevant control data, if applicable;

9) urinalysis tests utilized and results with relevant control data, if applicable;

10) terminal body weight and organ weight data, if applicable;

11) necropsy findings;

12) detailed description of all histopathological findings, if applicable;

13) statistical evaluation of results, if used, and a discussion of their biological significance.

g) Discussion of results.

h) Conclusions.

i) Quality assurance statement.

© ISO 2017 – All rights reserved

An acute systemic toxicity study will provide information on the effects of acute exposure to a test substance. Extrapolation of the results of the study to humans is valid to a limited degree but it can provide useful information on permissible exposure.

6 Repeated exposure systemic toxicity (subacute, subchronic and chronic systemic toxicity)

6.1 General

While acute toxicity deals with the adverse effects of single doses (or limited exposure), a more common form of human exposure to many medical devices is in the form of repeated or continuous exposures. Effects from repeated or continuous exposure may potentially occur due to accumulation of chemicals in tissues or by other mechanisms. Long term testing (subacute, subchronic, chronic) can identify these potential effects.

Repeated exposure systemic toxicity tests provide information on health hazards likely to arise from a prolonged exposure by the intended clinical route. It might also provide information on the mode of toxic action of a substance by the intended clinical exposure route.

Repeated exposure systemic toxicity studies will provide detailed information on toxic effects, target organs, reversibility or other effects and may serve as the basis for safety estimation. Results of these studies provide important information that is reflected in the extent of the guidance of clinical and anatomic pathology investigations.

Repeated exposure studies do not generally provide a retest criterion. Rather, group sizes are designed to accommodate a statistical assessment of the recorded observations (see Table 1).

Because of the variable durations for repeated exposure studies, test samples shall be prepared as required, to ensure their stability.

6.2 Study design

6.2.1 Preparations

Healthy young adult animals are acclimatized to the laboratory conditions for at least 5 d prior to the test. Animals are then randomized and assigned to the treatment groups.

6.2.2 Experimental animals

6.2.2.1 Selection of species

Typically the rodent (rat, mouse) will be used. Characteristics of the model (age, weight, etc.) are specified in 4.2 and 4.3. When non-rodent species are used they shall be scientifically justified.

6.2.2.2 Number and sex

The number and type of groups, animals per group, and sex are as specified in 4.5. When scientifically justified, consideration should be given to the use of satellite animals treated with the high dose level along with satellite controls for a predetermined period beyond the terminal euthanasia. This group, with its controls, may be used to examine treatment effects including reversibility, persistence or delayed toxic effects. For subchronic studies the satellite animals shall be maintained for not less than 28 d.

6.2.2.3 Housing and feeding conditions

The temperature and the relative humidity in the experimental animal rooms should be appropriate for the species, e.g. (22 ± 3) °C and 30 % to 70 % RH, for rats. Typically, the artificial lighting sequence should be 12 h light, 12 h dark.

 © ISO 2017 – All rights reserved

For feeding, standardized commercial laboratory diets may be used with an unlimited supply of drinking water. Animals may be caged in groups by sex or individually with justification, as appropriate; for group housing not more than five animals should be housed per cage.

6.2.3 Test conditions

6.2.3.1 Dose levels

The dose to use for toxicity tests of medical devices shall be defined in relation to the results of risk assessment, balancing the clinical exposure dose with the use of safety factors, as applicable. Except for treatment with the test substance, animals in the control group should be handled in an identical manner to the test group subjects.

Unlike classical chemical studies of repeated exposure systemic toxicity, repeated exposure studies with medical devices often do not result in a dose-response effect, thus a toxic effect at the dose level investigated is not mandatory.

6.2.3.2 Procedure

Animals should be dosed ideally 7 d/week for the duration of the study. For longer term repeated exposure studies, dosing on 5 d/week basis is acceptable but should be documented and justified.

6.2.4 Body weights

Body weight measurements should be made immediately before dosing, weekly after the first dose if indicated by study duration, and at the end of the study.

6.2.5 Clinical observations

The observation period for a repeated dose systemic toxicity study shall be appropriate for the duration of the study. Specifics of frequency and observation type are specified in 4.10 and Annex C. In all cases, observations shall be made at a frequency, and appropriate actions taken, to minimize the loss of animals to the study, e.g. necropsy or refrigeration of those animals found dead and isolation or euthanasia of weak or moribund animals. Cage-side observations should include, but not be limited to, changes in skin and fur, eyes and mucous membranes, and also respiratory, circulatory, autonomic and central nervous system, somatomotor activity and behaviour pattern, using the descriptors provided in Annex C.

Typically, ophthalmologic examinations, using an ophthalmoscope or equivalent suitable equipment, should be made prior to the administration of the test substance and at the termination of the study, preferably in all animals but at least in the high dose and control groups. If changes in the eyes are detected, all animals should be examined. Exception to examination should be documented and justified.

6.2.6 Pathology

6.2.6.1 Clinical pathology

The following examinations should be made.

a) Haematology, as specified in Annex D, should be investigated at the end of the test period. Depending on the length of the study, more frequent sampling should be considered.

b) Clinical biochemical determination on blood should be carried out at the end of the test period. Depending on the length of the study, more frequent sampling should be considered. Test areas that are considered appropriate to all repeated exposure studies are electrolyte balance, carbohydrate metabolism, and liver and kidney function. The selection of specific tests may be influenced by observations on the mode of action of the test substance. Suggested determinations are listed

© ISO 2017 – All rights reserved

in Annex D. Additional clinical biochemistry may be utilized where necessary to extend the observation of the observed effects.

Urinalysis (see Annex D) is not necessary on a routine basis but only when there is an indication based on expected or observed toxicity.

Historical data for normal values are useful for establishing baseline levels and for comparison with concurrent study controls. If historical baseline data are deemed inadequate, consideration should be given to the collection of this information for animals of the same age, sex, strain and source, preferably within the same laboratory.

6.2.6.2 Gross pathology

All animals should be subjected to full gross necropsy, which includes examination of the external surface of the body, all orifices, and the cranial, thoracic and abdominal cavities and their contents. The adrenals, brain, epididymis, heart, kidneys, liver, ovaries, spleen, testes, thymus and uterus should be weighed wet as soon as possible after dissection to avoid drying and subsequent falsely low values. The organs and tissues listed in Annex E should be preserved in a suitable medium for possible future histopathological examination.

6.2.6.3 Histopathology

a) Full histopathology should be carried out on organs and tissues from animals in the control and high dose groups.

b) All gross lesions should be examined.

c) The lungs of animals in the low and intermediate dose groups, if used, should be subjected to histopathological examination for evidence of infection, since this provides a convenient assessment of the state of health of the animals. Consideration should also be given to histopathological examination of the liver and kidneys in these groups. Further histopathological examination may not be required routinely on the animals in these groups but shall always be carried out in organs which showed evidence of lesions in the high dose group.

d) When a satellite group is used, histopathology may be performed on tissues and organs identified as showing effects in the treated groups.

e) In general, for chronic studies, sentinel animals should be used for monitoring the occurrence of infectious agents. Serology or histology of sentinel groups may be performed as indicated.

f) During selection of organs for histopathology, due consideration should be given to chemical characterization of device materials. For example, if the device is coated with drugs/pharmaceutical agents, then target organs for those chemicals should be studied in treated animals for any adverse effects.

6.3 Evaluation criteria

6.3.1 General

Data may be summarized in tabular form, showing, for each test group, the number of animals at the start of the test, the number of animals showing lesions, the types of lesions and the percentage of animals displaying each type of lesion. Statistical evaluations should be performed but biological relevance should be considered. Generally accepted statistical methods should be used and selected during the design phase of the study.

6.3.2 Evaluation of results

The findings of a repeated exposure study should be evaluated in conjunction with the findings of preceding studies and considered in terms of the toxic effects and the necropsy and histopathological

 © ISO 2017 – All rights reserved

findings. The evaluation shall include the relationship between the dose of the test substance and the observed effects. Observed effects including behavioural and clinical abnormalities, gross lesions, microscopic changes, effects on mortality and any other effects should be evaluated for their biological significance. Evaluation of observed effects should also consider their relevance to humans.

6.4 Final report

The information given in 5.4 shall be contained in the final report for the repeated exposure systemic toxicity study. In addition, the following information shall be provided:

— haematological tests utilized and results with relevant control data;

— clinical biochemistry tests utilized and results with relevant control data;

— histopathological findings;

— a statistical evaluation of results, where used, and a discussion of their biological significance.

A long-term systemic toxicity study will provide information on the effects of repeated exposure to a test substance. Extrapolation of the results of the study to humans is valid to a limited degree but it can provide useful information on permissible human exposure.

© ISO 2017 – All rights reserved

Annex A
(informative)

Routes of administration

A.1 General

Several routes of administration are listed in A.2 to A.10. Other routes of administration may be more clinically relevant and should be utilized. The most relevant route of administration shall be used. If an alternative route of administration is used it shall be justified. Expert consultation is suggested when designing appropriate studies.

A.2 Dermal

Tests for systemic toxicity by the dermal route may be appropriate for surface devices. Consideration should be given to limiting animal oral access to the test sample.

A.3 Implantation

Tests for systemic toxicity by implantation may be appropriate for implanted devices. The test may be appropriate for direct testing of a material by application to a general or specific area. Shape and texture of the test article should be taken into consideration. Methods for implantation can be found in ISO 10993-6.

A.4 Inhalation

Tests for systemic toxicity by the inhalation route may be appropriate for devices with a contact environment conducive to volatile chemical vapour leaching or for an aerosol/particulate test sample with potential for inhalation. Protocol specifics for this route of administration may be found in most dedicated texts for inhalation toxicology.

A.5 Intradermal

Tests for systemic toxicity by the intradermal route may be appropriate for a device with an intradermal contact environment conducive to chemical leaching. Test samples are typically administered directly to the intradermal region by injection. The use of multiple treatment sites should be clearly specified and justified.

A.6 Intramuscular

Tests for systemic toxicity by the intramuscular route may be appropriate for devices with a muscle tissue contact environment conducive to chemical leaching. Test samples are typically administered directly to the muscle tissue by injection or surgical implantation. Sites need to be chosen to minimize the loss of function or the possibility of pain from nerve damage caused by muscle fibre tension from the injected or implanted test sample. Sites should be rotated for repeated dose studies since, for example, non-aqueous formulations may remain as a depot for >24 h. The use of multiple treatment sites should be clearly specified and justified.

 © ISO 2017 – All rights reserved

A.7 Intraperitoneal

Tests for systemic toxicity by the intraperitoneal route may be appropriate for devices with a fluid-path or peritoneal cavity contact environment conducive to chemical leaching. This is also an appropriate route when the extract should not be given intravenously, such as with non-polar oil extracts and where particulates might be present. This route is preferable to filtering for an intravenous injection. Test samples are typically administered directly to the peritoneal cavity. Dose frequency calculations should consider that test articles administered by this route are absorbed primarily through the portal circulation and therefore shall pass through the liver before reaching general circulation. Care should be taken not to inject into the stomach or intestinal tract.

A.8 Intravenous

Tests for systemic toxicity by the intravenous route may be appropriate for devices with a direct or indirect fluid-path or blood contact environment conducive to chemical leaching. Test samples are typically placed in or administered directly to the vascular system. If particulates are present, delivery by the intraperitoneal route or sample filtration should be considered. For the evaluation of nanomaterials, nanomaterial dispersions themselves may be considered for intravenous administration. Recommended dosage volumes and rates of administration for intravenous studies with the most commonly used laboratory animal species are listed in Annex B.

Care should be taken to minimize the possibility of extra vascular injection of test sample. For injection taking 5 min or more, consideration should be given to the use of a butterfly needle or an intravenous cannula.

A.9 Oral

Tests for systemic toxicity by the oral route may be appropriate for devices contacting the oral mucosa directly or indirectly, or for products with other enteral application. Test samples are typically administered by gavage. Experimental animals should generally be fasted prior to test sample administration. The period of fasting may range from hours to overnight, with the shorter periods for animals with higher metabolic rates. Following the period of fasting, the animals should be weighed and then the test sample administered in a single dose based on body weight. After the test sample has been administered, food may be withheld for an additional 3 h to 4 h. Where a dose is administered in fractions over a certain period, it may be necessary to provide the animals with food and water depending on the length of the period.

A.10 Subcutaneous

Tests for systemic toxicity by the subcutaneous route may be appropriate for a device with a subcutaneous contact environment conducive to chemical leaching. Test samples are typically administered directly to the subcutaneous region by injection or by implantation. The use of multiple treatment sites should be clearly specified and justified.

© ISO 2017 – All rights reserved

Annex B
(informative)

Dosage volumes

B.1 General

The principles of humane animal research require that all reasonable efforts be made to minimize or eliminate all adverse physiological or pathological effects. The values listed in Table B.1 are intended to be informative and represent the maximum limits reported in the literature for single dose administrations. These maximum values should not be taken as a recommendation in this document but investigators should apply upper limits with regard to factors such as body weight/surface area, rate of administration, number and frequency of administrations, physical-chemical and biological properties of the test sample, and animal strain. For repeated dose administrations attempts should be made to minimize the dosage volume while taking into consideration these adjustment factors.

Table B.1 — Maximum single dosage volumes (ml/kg) for test sample administration

Species	Subcutaneous<br>ml/kg	Intramuscular<br>ml/kg	Intraperitoneal<br>ml/kg	Gavage<br>ml/kg	Intravenous<br>ml/kg
Rat	20	1	20	50	40
Mouse	50	2	50	50	50
Rabbit	10	1	20	20	10
Dog	2	1	20	20	10
Monkey	5	1	20	15	10
Regulations of individual countries may supersede the maximum volumes listed above. Intramuscular administrations in rodents should not exceed 0,1 ml/site (mouse) and 0,2 ml/site (rat), while intravenous dosage volumes should not exceed 1 ml/min.					

B.2 Dosage volume references

See References [11],[12],[13],[14],[15],[16].

© ISO 2017 – All rights reserved

Annex C
(informative)

Common clinical signs and observations

Table C.1 — Common clinical signs and observations

Clinical observation	Observed sign	Involved system(s)
Respiratory	Dyspnea (abdominal breathing, gasping), apnoea, cyanosis, tachypnea, nostril discharges	CNS, pulmonary, cardiac
Motor activities	Decrease/increase somnolence, loss of righting, catalepsy, ataxia, unusual locomotion, prostration, tremors, fasciculation	CNS, somatomotor, sensory, neuromuscular, autonomic
Convulsion	Clonic, tonic, tonic-clonic, asphyxial, opisthotonos	CNS, neuromuscular, autonomic, respiratory
Reflexes	Corneal, righting, myotactic, light, startle reflex	CNS, sensory, autonomic, neuromuscular,
Ocular signs	Lacrimation, miosis, mydriasis, exophthalmos, ptosis, opacity, iritis, conjunctivitis, chromodacryorrhea, relaxation of nictitating membrane	Autonomic, irritation
Cardiovascular signs	Bradycardia, tachycardia, arrhythmia, vasodilation, vasoconstriction	CNS, autonomic, cardiac, pulmonary
Salivation	Excessive	Autonomic
Piloerection	Rough hair	Autonomic
Analgesia	Decrease reaction	CNS, sensory
Muscle tone	Hypotonia, hypertonia	Autonomic
Gastrointestinal	Soft stool, diarrhoea, emesis, diuresis, rhinorrhoea	CNS, autonomic, sensory, GI motility, kidney
Skin	Oedema, erythema	Tissue damage, irritation

© ISO 2017 – All rights reserved

Annex D
(informative)

Suggested haematology, clinical chemistry and urinalysis measurements

D.1 Haematology

— Clotting potential (PT, APTT)

— Haemoglobin concentration

— Haematocrit

— Platelet count

— Red blood cell count

— White blood cell count

— WBC differential

D.2 Clinical chemistry

— Albumin

— ALP

— ALT

— AST

— Calcium

— Chloride

— Cholesterol

— Creatinine

— GGT

— Glucose

— Inorganic phosphorus

— Potassium

— Sodium

— Total bilirubin

— Total protein

— Triglycerides

— Urea nitrogen

© ISO 2017 – All rights reserved

— Additional enzymes, as scientifically appropriate

— Total immunoglobulin levels may be considered as an indicator for immunotoxicity

D.3 Urinalysis (timed collection, e.g. 16 h to 24 h)

— Appearance

— Bilirubin

— Glucose

— Ketones

— Occult Blood

— Protein

— Sediment

— Specific gravity or osmolality

— Volume

— Other scientifically appropriate tests if test article is suspected to cause specific organ toxicity (generally requires refrigerated sample collection)

© ISO 2017 – All rights reserved

Annex E
(informative)

Suggested organ list for histopathological evaluation

In addition to the histopathological evaluation, organs/tissues marked with an asterisk (*) below should be weighed, with other organs weighed if scientifically appropriate. The clinical and other findings may suggest the need to examine additional tissues. Also, any organs considered likely to be target organs based on the known properties of the test substance should be preserved.

Full histopathology should be carried out on the preserved organs and tissues of all animals in the control and highest dose group. These examinations, targeted to specific organs/tissues as necessary, should be extended to animals of all other dosage groups if treatment-related changes are observed in the highest dosage group.

— Adrenals*

— All gross lesions (including treatment sites)

— Aorta

— Bone marrow (femur, rib or sternum)

— Brain* (representative sections including cerebrum, cerebellum and pons)

— Caecum

— Colon

— Duodenum

— Epididymis*

— Oesophagus

— Eyes

— Gall bladder (if present)

— Heart*

— Ileum

— Jejunum

— Kidneys*

— Liver*

— Lungs and bronchi (preserved by inflation with fixative and then immersion)

— Lymph nodes (local to cover site of administration and distant to cover systemic effects)

— Mammary gland (female)

— Muscle (skeletal)

— Nasal turbinates (for inhalation studies)

— Nerve (sciatic or tibial) preferably in close proximity to the muscle

© ISO 2017 – All rights reserved

— Ovaries*
— Pancreas
— Parathyroid
— Pituitary
— Prostate
— Rectum
— Salivary glands
— Seminal vesicles
— Skin
— Spinal cord
— Spleen*
— Sternum
— Stomach
— Testes*
— Thymus*
— Thyroid
— Trachea
— Urinary bladder
— Uterus* (including cervix and oviducts)
— Vagina

© ISO 2017 – All rights reserved

Annex F
(informative)

Organ list for limited histopathology for medical devices subjected to systemic toxicity testing

F.1 General

Many medical devices employ commonly used materials differing only in the amount or type of chemical additives, processing or sterilization methods.

When a toxicological risk assessment of the device extractables/leachables determines that a biocompatibility/safety assessment for potential systemic effects is required a reduced histopathology evaluation may be considered. In this model, a limited number of potential target organs/tissues are examined using a tiered approach.

F.2 Procedure

All tissues indicated in Annex E should be collected and preserved.

Limited histopathological analysis should be completed for all Tier I organs/tissues listed in Table F.1.

If abnormal or questionable findings are observed in Tier I tissues, or in the concurrent clinical pathology (clinical chemistry and haematology), proceed to Tier II (examine the full list of organs/tissues in Annex E).

Table F.1 — Organ list for limited histopathology

Organ system	Organs/tissue (when species applicable) of system	Tier I tissues
Circulatory	Heart, arteries, veins, capillaries, blood	Heart
Digestive	Mouth, salivary glands, oesophagus, liver, stomach, gallbladder, pancreas, intestines (duodenum, transverse colon, ascending colon, descending colon, ileum, jejunum, caecum, sigmoid colon), rectum, anus	Liver
Endocrine	Hypothalamus, pituitary gland, thyroid, parathyroid, adrenals, pineal gland, pancreas	Adrenals
Excretory	Kidneys, ureters, bladder, urethra,, skin, lungs, rectum	Kidneys
Integumentary	Skin, subcutaneous tissue, hair, nails	Skin
Lymphatic	Lymph nodes, tonsils, adenoids, thymus, spleen	Spleen
Muscular	Biceps, triceps, deltoids, gluteus, hamstring, tendons	Muscle
Nervous	Brain, spinal cord, nerves, peripheral nerves, eyes, ears	Brain
Reproductive	Ovaries, fallopian tubes, uterus, vagina, mammary glands, testes, vas deferens, seminal vesicles, prostate, penis	Testes, ovaries
Respiratory	Nose, nasal cavities, pharynx, larynx, trachea, bronchi, lungs, diaphragm	Lungs, bronchi
Skeletal	Femur, humerus, radius, ulna, cranium, sternum, clavicle, fibula, tibia, vertebrae, scapula, pelvis, coccyx, phalanges, marrow, cartilage, ligaments	Femur or sternum
Haematopoietic	Bone marrow	Femur, rib or sternum
Other	Gross lesions including treatment site	As observed

© ISO 2017 – All rights reserved

Annex G
(informative)

Information on material-mediated pyrogens

Pyrogenicity is the ability of a chemical agent or other substance to produce a febrile response. Pyrogenic responses may be material-mediated, endotoxin-mediated, or mediated by other substances, such as components of gram-positive bacteria and fungi. This document is concerned with material-mediated pyrogenicity.

It is not necessary to test all new medical devices for *in vivo* pyrogenicity. However, materials containing substances that have previously elicited a pyrogenic response, and/or new chemical entities where the pyrogenic potential is unknown should be evaluated for material-mediated pyrogenicity. For medical devices that may be used in a combination product, testing to satisfy the product pyrogenicity should be considered. Endotoxin contamination may be a source of a pyrogenic response, and should not be confused with a material-mediated pyrogenic response.

— Endotoxin-mediated pyrogenicity

This form of pyrogenicity originating from biologically active endotoxin of gram-negative bacteria, which is usually a fever-inducing contamination in the manufacturing process of medical devices, is evaluated by measuring the amount of endotoxin in the devices by an endotoxin-specific LAL (Limulus Amebocyte Lysate) test without performing a rabbit test (see Reference [3]).

— Material-mediated pyrogenicity

This type of pyrogenicity originates from non-endotoxin related factors. The following is a list of substances which are known to generate a pyrogenic response, without being endotoxins:

— endogenous pyrogens (e.g. IL-1, IL-6, TNFα, INF-γ);

— prostaglandin;

— inducers (e.g. polyadenylic, polyuridylic, polybionosinic and polyribocytidylic acids);

— substances disrupting the function of thermoregulatory centres (e.g. LSD, cocaine, morphine);

— uncoupling agents of oxidative phosphorylation (e.g. 4, 6-dinitro-o-cresol, dinitrophenol, picric acid);

— *N*-phenyl-β-naphthylamine and aldo-α-naphthylamine (the febrile mechanism is unknown);

— bacterial exotoxins (e.g. TSST-1, SEA, Spe F, Spe C);

— neurotransmitters (e.g. noradrenaline, serotonin);

— metals such as nickel salts, in some instances.

For detection of material-mediated pyrogenicity, the rabbit pyrogen test, which has a wide range for detecting pyrogenic activity, is currently recommended. Methods for performing the rabbit pyrogen test can be found in the United States Pharmacopoeia, the European Pharmacopoeia and the Japanese Pharmacopoeia. The LAL test is not suitable for determining the pyrogenicity of these substances. Should other methods for detecting non-endotoxin pyrogenicity be developed, and become validated, these will be considered for replacement of the rabbit test.

Ongoing developments are assays based on cytokine release by monocytes/macrophages which are able to detect pyrogenicity related to components of gram-negative and gram-positive bacteria and fungi. These *in vitro* assays are not validated for material mediated pyrogenicity.

© ISO 2017 – All rights reserved

Annex H
(informative)

Subchronic rat — Dual routes of parenteral administration

H.1 General

Many devices requiring subacute/subchronic toxicity testing are implantable devices and thus the most clinically relevant route of exposure in the animal model is via implantation. However, when the device is not intended for implant, exposure of the device via dosing of extracts is an option. Concurrent parenteral administration of polar and nonpolar extracts can be an option.

Clinically, when a medical device is implanted or externally communicating, exposure to polar and nonpolar leachables may be concurrent. One approach to assess toxicity is to inject both polar and nonpolar extracts into the same animal. This exposure more closely simulates the clinical experience of total extractables exposure. This model may not be appropriate when there is a need to study the administration routes separately. In that case Clause 6 should be considered.

Recommended dosing parameters for the dual routes of parenteral administration model are as specified in the Table H.1.

Table H.1 — Recommended dosing parameters

Number of animals/ sex/group[a]	Route	Dose		
		Volume<br>ml/kg[b]	Frequency<br>Study days[d]	Rate<br>ml/min
6	Intravenous	10	Daily for 14 d	≤2
	Intraperitoneal	5[c]	1, 4, 7, 10, 13	Slow bolus

[a] Vehicle control animals (6/sex) should be dosed similarly.

[b] Volumes are recommended.

[c] Sesame oil is preferred.

[d] Dosing days may begin on day 0.

H.2 Procedure

The test animals are dosed with the polar test sample extract, and the control animals are dosed with polar vehicle, intravenously 7 d/week over the duration of the study (i.e. 14 doses). For nonpolar test samples the same test animals are dosed with the nonpolar test sample extract, and the control animals are dosed with the nonpolar vehicle, intraperitoneally every third day over the duration of the study (i.e. 5 doses).

H.3 Dosage volume and frequency justification

H.3.1 Intravenous

Table B.1 describes the maximum dosage volumes for test sample administration when a single or a very limited number of intravenous treatments are required. For daily-repeated or recurrent administrations by any route the test sample volume should be reduced. LASA (see Reference [17]) recommends a maximum intravenous dosage volume of 5 ml/kg for a bolus injection in the rat carried out over a relatively short period of time (less than 1 min), and for once daily dosing on a routine basis

© ISO 2017 – All rights reserved

(injection rate ≤2 ml/min). For repeated intravenous administration of medical device extracts a dose volume of 10 ml/kg is considered unlikely to cause undue stress in the animals, see Reference [13].

H.3.2 Intraperitoneal

Table B.1 describes the maximum dosage volumes for test sample administration when a single or a very limited number of intraperitoneal treatments are required. Current experience indicates that 5 ml/kg of sesame oil extract given intermittently is well tolerated. Sufficient anecdotal evidence suggests that a peritoneal residual volume (PRV) of non-aqueous injectates of 5 ml/kg to 10 ml/kg may persist for up to three days. Consequently, for repeated intraperitoneal administration of nonpolar medical device extracts, when administered concurrent with an intravenous injection of 10 ml/kg, a dose volume of 5 ml/kg is considered unlikely to cause undue stress in the animal and represents an acceptable protracted exposure volume for nonpolar extracts.

Several complications by the intraperitoneal route of administration are well documented. These include bleeding at the injection site, paralytic ileus due to substance injected, laceration of abdominal organs, peritonitis, and injection into the gastrointestinal tract or bladder. In that regard, the frequency of erroneous intraperitoneal injections by skilled investigators has been reported to range from 11 % to 20 % (see Reference [18]). In view of this, and with consideration of the PRV and the potential for increasing intraperitoneal injectate volume with too frequent dosing, a thrice weekly administration schedule is considered unlikely to cause undue stress in the animal and represents an acceptable protracted exposure frequency for non-aqueous injectates.

General aspects of study design are covered in Clause 6.

© ISO 2017 – All rights reserved

Bibliography

General

[1] ISO 10993, *Biological evaluation of medical devices*

[2] ASTM F619-03, *Standard Practice for Extraction of Medical Plastics*

[3] AAMI/ST72, *Bacterial Endotoxin — Test methods, routine monitoring, and alternatives to batch testing*

[4] U.S./EPA PB 86/108958 and 89/124077

[5] U.S./FDA Toxicological principles for the safety assessment of direct food additives, 1982

[6] U.S. Code of Federal Regulation 1500.40: Method of Testing Toxic Substances

[7] United States Pharmacopoeia 26: Biological Reactivity Tests, In Vivo; The National Formulary 21, Rockville, MD; Pharmacopoeial Convention, 2003, pp. 2028-2032

[8] European Pharmacopoeia. Eighth Edition, 2014

[9] MHLW Notification by Director, OMDE, Yakushokuki-hatsu 0301 No. 20, March 1, 2012. Basic Principles of Biological Safety Evaluation Required for Application for Approval to Market Medical Devices

[10] OECD. Series on Testing and Assessment No. 129: Guidance Document on Using Cytotoxicity Tests to Estimate Starting Doses for Acute Oral Systemic Toxicity Tests

Dosage volume references

[11] Hull R.M. Guideline limit volumes for dosing animals in the preclinical stage of safety evaluation. Human and Environmental Toxicology. 1995, **14** pp. 305–307

[12] Derelanko M.J., & Hollinger M.A. CRC Handbook of Toxicology. CRC Press, NY, Second Edition, 2001, pp. 98.

[13] Diehl K.-H., Hull R., Morton D., Pfister R., Rabemampianina Y., Smith D. Vidal, J.–M, Van De Vorstenbosch. A good practice guide to the administration of substances and removal of blood, including routes and volumes. J. Appl. Toxicol. 2001, **21** pp. 15–23

[14] Morton D. Effects of infusion rates in rats receiving repeated large volumes of intravenous saline solution. Lab. Anim. Sci. 1997, **47** pp. 656–659

[15] Richmond J.D. Dose limit volumes: The United Kingdom view — past and present. Presented at the Humane Society of the United States — Refinement in Toxicology Testing: Dosing Data: Volume and Frequency, March 14, 1999, New Orleans, LA

[16] Morton D.B. Refining procedures for the administration of substances. Report of the BVAAWF/FRAME/RSPCA/UFAW Joint Working Group on Refinement. Lab. Anim. 2001, **35** pp. 1–41

[17] Laboratory Animal Science Association (LASA) Good Practice Guidelines: Administration of Substances (Rat, Mouse, Guinea Pig, Rabbit) – Series 1/Issue 1 – October 1998

[18] Nebendahl K. Routes of Administration. In: The Laboratory Rat: A Volume in Handbook of Experimental Animals, (Krinke G.J. ed.). Elsevier Ltd, 2000, pp. 463–83.

[19] Gaines Das R., & North D. Implications of experimental technique for analysis and interpretation of data from animal experiments: outliers and variability resulting from failure of intraperitoneal injection procedures. Lab Anim. (NY). 2007, **41** (3) pp. 312–320

 © ISO 2017 – All rights reserved

[20] Coria-Avila GA, & Gavrila AM BA1, Shann Ménard S, Ismail N, Pfaus JG. Cecum location in rats and the implications for intraperitoneal injections. Lab. Anim. 2007, **36** (7) pp. 25–30

[21] OECD. (1981 – 2013): http://www.oecd-ilibrary.org/environment/oecd-guidelines-for-the-testing-of-chemicals-section-4-health-effects_20745788

© ISO 2017 – All rights reserved

ICS 11.100.20
Price based on 29 pages

© ISO 2017 – All rights reserved
