# Supplementary material for: Endocytosis-mediated healing: recombinant human collagen type III chain-induced wound healing for scar-free recovery
Source: Regen Biomater. 2025 Jan 8;12:rbae149. doi: 10.1093/rb/rbae149 (PMC11930350; doi:10.1093/rb/rbae149)
Supplement: rbae149_Supplementary_Data [file rbae149_supplementary_data.zip › beda2_Supplementary 2.docx]

Supplementary 2-1


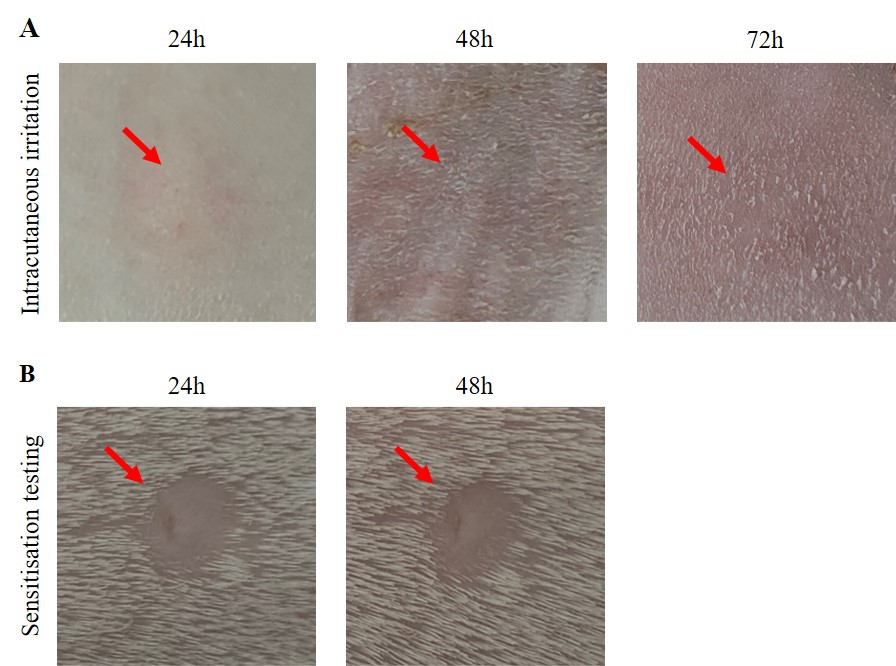


Supplementary 2-1. Biocompatibility. A. Intracutaneous irritation. No evidence of erythema and oedema at 24, 48, and 72h. B. Sensitisation testing. No evidence of erythema and oedema at 24, and 48h.

Supplementary 2-2


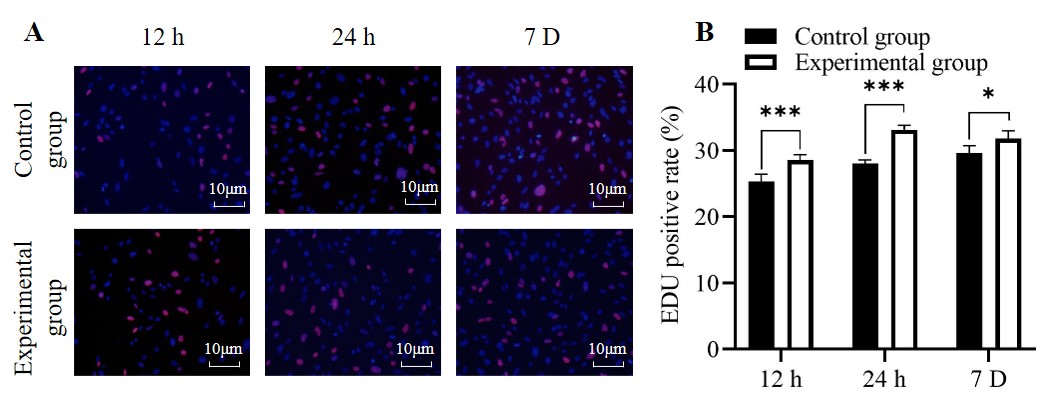


Supplementary 2-2. The effect of HSF on cell proliferation. (A) EdU immunofluorescence, (B). HSF proliferation rate (EdU). * Presence of significant differences (p < 0.05). ***Presence of significant differences (p < 0.001). HSF, human skin fibroblast; EdU, 5-ethynyl-2′-deoxyuridine.

Supplementary 2-3


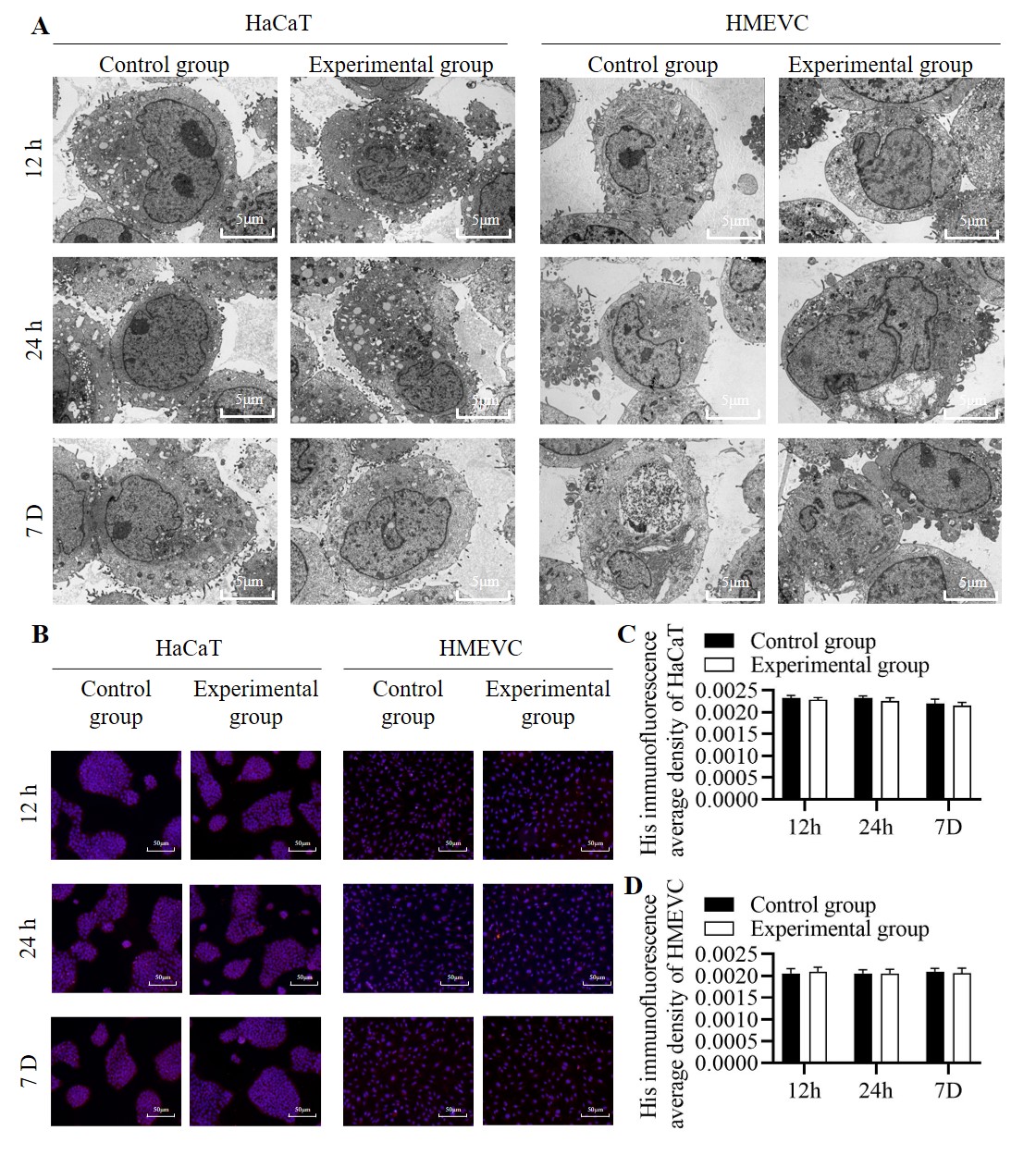


Supplementary 2-3. Endocytosis of RHC III chain in a cell-based experiment (HaCaT and HMEVC). (A) Scanning electron microscopy of endocytosis after HaCaTs and HMEVCs were co-cultured with RHC3. (B~D) Mean optical density of intracellular His was assessed using an immunofluorescence assay. HaCaT, Immortalized human keratinocytes; HMEVC, human microvascular endothelial cells; RHC III chain, recombinant human collagen type III chain.

Supplementary 2-4


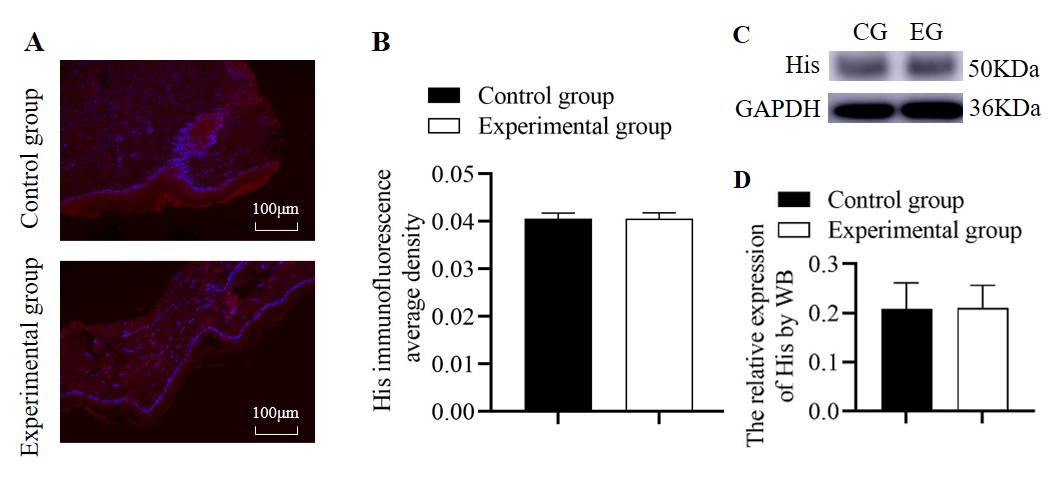


Supplementary 2-4. Intracellular endocytosis of RHC III chain in rabbit ear scars. (A, B) Immunofluorescence assay of His. (C, D) Western blot (CG, control group; EG, experimental group) assessing His. GAPDH, glyceraldehyde 3-phosphate dehydrogenase; His, histidine.
