# Supplementary material for: Endocytosis-mediated healing: recombinant human collagen type III chain-induced wound healing for scar-free recovery
Source: Regen Biomater. 2025 Jan 8;12:rbae149. doi: 10.1093/rb/rbae149 (PMC11930350; doi:10.1093/rb/rbae149)
Supplement: rbae149_Supplementary_Data [file rbae149_supplementary_data.zip › e04ec_Supplementary 1-1.ISO 10993-5-2009.pdf]

INTERNATIONAL STANDARD

ISO 10993-5

Third edition
2009-06-01

Biological evaluation of medical devices —

Part 5:
Tests for *in vitro* cytotoxicity

Évaluation biologique des dispositifs médicaux —

Partie 5: Essais concernant la cytotoxicité in vitro

Reference number
ISO 10993-5:2009(E)

© ISO 2009

PDF disclaimer

This PDF file may contain embedded typefaces. In accordance with Adobe's licensing policy, this file may be printed or viewed but shall not be edited unless the typefaces which are embedded are licensed to and installed on the computer performing the editing. In downloading this file, parties accept therein the responsibility of not infringing Adobe's licensing policy. The ISO Central Secretariat accepts no liability in this area.

Adobe is a trademark of Adobe Systems Incorporated.

Details of the software products used to create this PDF file can be found in the General Info relative to the file; the PDF-creation parameters were optimized for printing. Every care has been taken to ensure that the file is suitable for use by ISO member bodies. In the unlikely event that a problem relating to it is found, please inform the Central Secretariat at the address given below.

COPYRIGHT PROTECTED DOCUMENT

© ISO 2009

All rights reserved. Unless otherwise specified, no part of this publication may be reproduced or utilized in any form or by any means, electronic or mechanical, including photocopying and microfilm, without permission in writing from either ISO at the address below or ISO's member body in the country of the requester.

ISO copyright office
Case postale 56 • CH-1211 Geneva 20
Tel. + 41 22 749 01 11
Fax + 41 22 749 09 47
E-mail copyright@iso.org
Web www.iso.org

Published in Switzerland

Contents

Page

Foreword ... iv
Introduction ... vi
1 Scope ... 1
2 Normative references ... 1
3 Terms and definitions ... 1
4 Sample and control preparation ... 2
4.1 General ... 2
4.2 Preparation of liquid extracts of material ... 3
4.3 Preparation of material for direct-contact tests ... 4
4.4 Preparation of controls ... 5
5 Cell lines ... 5
6 Culture medium ... 5
7 Preparation of cell stock culture ... 6
8 Test procedures ... 6
8.1 Number of replicates ... 6
8.2 Test on extracts ... 6
8.3 Test by direct contact ... 7
8.4 Test by indirect contact ... 7
8.5 Determination of cytotoxicity ... 9
9 Test report ... 10
10 Assessment of results ... 11
Annex A (informative) Neutral red uptake (NRU) cytotoxicity test ... 12
Annex B (informative) Colony formation cytotoxicity test ... 19
Annex C (informative) MTT cytotoxicity test ... 24
Annex D (informative) XTT cytotoxicity test ... 29
Bibliography ... 34

© ISO 2009 – All rights reserved

Foreword

ISO (the International Organization for Standardization) is a worldwide federation of national standards bodies (ISO member bodies). The work of preparing International Standards is normally carried out through ISO technical committees. Each member body interested in a subject for which a technical committee has been established has the right to be represented on that committee. International organizations, governmental and non-governmental, in liaison with ISO, also take part in the work. ISO collaborates closely with the International Electrotechnical Commission (IEC) on all matters of electrotechnical standardization.

International Standards are drafted in accordance with the rules given in the ISO/IEC Directives, Part 2.

The main task of technical committees is to prepare International Standards. Draft International Standards adopted by the technical committees are circulated to the member bodies for voting. Publication as an International Standard requires approval by at least 75 % of the member bodies casting a vote.

Attention is drawn to the possibility that some of the elements of this document may be the subject of patent rights. ISO shall not be held responsible for identifying any or all such patent rights.

ISO 10993-5 was prepared by Technical Committee ISO/TC 194, *Biological evaluation of medical devices*.

This third edition cancels and replaces the second edition (ISO 10993-5:1999) which has been technically revised.

ISO 10993 consists of the following parts, under the general title *Biological evaluation of medical devices*:

— *Part 1: Evaluation and testing within a risk management process*

— *Part 2: Animal welfare requirements*

— *Part 3: Tests for genotoxicity, carcinogenicity and reproductive toxicity*

— *Part 4: Selection of tests for interactions with blood*

— *Part 5: Tests for* in vitro *cytotoxicity*

— *Part 6: Tests for local effects after implantation*

— *Part 7: Ethylene oxide sterilization residuals*

— *Part 9: Framework for identification and quantification of potential degradation products*

— *Part 10: Tests for irritation and skin sensitization*

— *Part 11: Tests for systemic toxicity*

— *Part 12: Sample preparation and reference materials*

— *Part 13: Identification and quantification of degradation products from polymeric medical devices*

— *Part 14: Identification and quantification of degradation products from ceramics*

— *Part 15: Identification and quantification of degradation products from metals and alloys*

© ISO 2009 – All rights reserved

— *Part 16: Toxicokinetic study design for degradation products and leachables*

— *Part 17: Establishment of allowable limits for leachable substances*

— *Part 18: Chemical characterization of materials*

— *Part 19: Physico-chemical, morphological and topographical characterization of materials* [Technical Specification]

— *Part 20: Principles and methods for immunotoxicology testing of medical devices* [Technical Specification]

© ISO 2009 – All rights reserved

Introduction

Due to the general applicability of *in vitro* cytotoxicity tests and their widespread use in evaluating a large range of devices and materials, it is the purpose of this part of ISO 10993, rather than to specify a single test, to define a scheme for testing which requires decisions to be made in a series of steps. This should lead to the selection of the most appropriate test.

Three categories of test are listed: extract test, direct contact test, indirect contact test.

The choice of one or more of these categories depends upon the nature of the sample to be evaluated, the potential site of use and the nature of the use.

This choice then determines the details of the preparation of the samples to be tested, the preparation of the cultured cells, and the way in which the cells are exposed to the samples or their extracts.

At the end of the exposure time, the evaluation of the presence and extent of the cytotoxic effect is undertaken. It is the intention of this part of ISO 10993 to leave open the choice of type of evaluation. Such a strategy makes available a battery of tests, which reflects the approach of many groups that advocate *in vitro* biological tests.

The numerous methods used and endpoints measured in cytotoxicity determination can be grouped into the following categories of evaluation:

— assessments of cell damage by morphological means;

— measurements of cell damage;

— measurements of cell growth;

— measurements of specific aspects of cellular metabolism.

There are several means of producing results in each of these four categories. The investigator should be aware of the test categories and into which category a particular technique fits, in order that comparisons be able to be made with other results on similar devices or materials both at the intra- and interlaboratory level. Examples of quantitative test protocols are given in annexes. Guidance for the interpretation of the results is given in this part of ISO 10993.

© ISO 2009 – All rights reserved

Biological evaluation of medical devices —

Part 5: **Tests for *in vitro* cytotoxicity**

1 Scope

This part of ISO 10993 describes test methods to assess the *in vitro* cytotoxicity of medical devices.

These methods specify the incubation of cultured cells in contact with a device and/or extracts of a device either directly or through diffusion.

These methods are designed to determine the biological response of mammalian cells *in vitro* using appropriate biological parameters.

2 Normative references

The following referenced documents are indispensable for the application of this document. For dated references, only the edition cited applies. For undated references, the latest edition of the referenced document (including any amendments) applies.

ISO 10993-1, *Biological evaluation of medical devices — Part 1: Evaluation and testing within a risk management system*

ISO 10993-12, *Biological evaluation of medical devices — Part 12: Sample preparation and reference materials*

3 Terms and definitions

For the purposes of this document, the terms and definitions given in ISO 10993-1 and the following apply.

3.1
culture vessels
vessels appropriate for cell culture including glass petri dishes, plastic culture flasks or plastic multiwells and microtitre plates

NOTE These can be used interchangeably in these methods provided that they meet the requirements of tissue culture grade and are suitable for use with mammalian cells.

3.2
positive control material
material which, when tested in accordance with this part of ISO 10993, provides a reproducible cytotoxic response

© ISO 2009 – All rights reserved

NOTE The purpose of the positive control is to demonstrate an appropriate test system response. For example, an organotin-stabilized polyurethane[1)] has been used as positive control for solid materials and extracts. Dilutions of phenol, for example, have been used as a positive control for extracts. In addition to a material, pure chemicals can also be used to demonstrate the performance of the test system.

3.3
blank
extraction vehicle not containing the test sample, retained in a vessel identical to that which holds the test sample and subjected to conditions identical to those to which the test sample is subjected during its extraction

NOTE The purpose of the blank is to evaluate the possible confounding effects due to the extraction vessel, vehicle and extraction process.

3.4
negative control material
material which, when tested in accordance with this part of ISO 10993, does not produce a cytotoxic response

NOTE The purpose of the negative control is to demonstrate background response of the cells. For example, high-density polyethylene[2)] for synthetic polymers, and aluminium oxide ceramic rods for dental material have been used as negative controls.

3.5
test sample
material, device, device portion, component, extract or portion thereof that is subjected to biological or chemical testing or evaluation

3.6
subconfluency
approximately 80 % confluency, i.e. the end of the logarithmic phase of growth

4 Sample and control preparation

4.1 General

The test shall be performed on

a) an extract of the test sample

and/or

b) the test sample itself.

Sample preparation shall be in accordance with ISO 10993-12.

Negative and positive controls shall be included in each assay.

1) The ZDEC and ZDBC polyurethanes are available from the Food and Drug Safety Center, Hatano Research Institute, Ochiai 729-5, Hadanoshi, Kanagawa 257, Japan.

2) High-density polyethylene can be obtained from the U.S. Pharmacopeia (Rockville, MD, USA) and from the Food and Drug Safety Center, Hatano Research Institute (Ochiai 729-5, Hadanoshi, Kanagawa 257, Japan).

The information given in 1) and 2) is for the convenience of the user of this part of ISO 10993 and does not constitute an endorsement by ISO of these products. Equivalent products may be used if they can be shown to lead to the same results.

© ISO 2009 – All rights reserved

4.2 Preparation of liquid extracts of material

4.2.1 Principles of extraction

Extracting conditions should attempt to simulate or exaggerate the clinical use conditions so as to determine the potential toxicological hazard without causing significant changes in the test sample, such as fusion, melting or any alteration of the chemical structure, unless this is expected during clinical application. Due to the nature of certain materials (e.g. biodegradable materials), alteration of the chemical structure can occur during the extraction procedure.

NOTE The concentration of any endogenous or extraneous substances in the extract, and hence the amount exposed to the test cells, depends on the interfacial area, the extraction volume, pH, chemical solubility, diffusion rate, osmolarity, agitation, temperature, time and other factors.

For devices that involve mixing two or more components in the patient to arrive at the final device (for example bone cement), the final device should not be washed prior to extraction. Washing the test sample can reduce or remove residuals present on the device. If the test sample is to be used in a sterile environment, a sterilized test sample should be used to extract chemical constituents.

4.2.2 Extraction vehicle

The choice of the extraction vehicle(s) taking into account the chemical characteristics of the test sample shall be justified and documented. For mammalian cell assays one or more of the following vehicles shall be used:

a) culture medium with serum;

b) physiological saline solution;

c) other suitable vehicle.

The choice of vehicle should reflect the aim of the extraction. Consideration shall be given to the use of both a polar and a non-polar vehicle. Culture medium with serum is the preferred extraction vehicle. The use of culture medium with serum is preferred for extraction because of its ability to support cellular growth as well as extract both polar and non-polar substances. In addition to culture medium with serum, use of medium without serum should be considered in order to specifically extract polar substances (e.g. ionic compounds). Other suitable vehicles include purified water and dimethyl sulfoxide (DMSO). DMSO is cytotoxic in selected assay systems at greater than 0,5 % (volume fraction). The cellular exposure concentration of extractables in DMSO will be lower due to the greater dilution as compared to extraction in culture medium with serum.

NOTE 1 Different types of serum (e.g. foetal, bovine/calf serum, newborn calf serum) might be used and the choice of the serum is dependent on the cell type.

NOTE 2 It is important to recognise that serum/proteins are known to bind, to some extent, extractables.

4.2.3 Extraction conditions

4.2.3.1 The extraction shall be performed in sterile, chemically inert, closed containers by using aseptic techniques, in accordance with ISO 10993-12.

4.2.3.2 With the exception of circumstances given below, the extraction shall be conducted under one of the following conditions and shall be applied according to the device characteristics and specific conditions for use:

a) (24 ± 2) h at (37 ± 1) °C;

b) (72 ± 2) h at (50 ± 2) °C;

c) (24 ± 2) h at (70 ± 2) °C;

d) $(1 \pm 0,2)$ h at (121 ± 2) °C.

© ISO 2009 – All rights reserved

Extraction conditions described above, which have been used to provide a measure of the hazard potential for risk estimation of the device or material, are based on historical precedent. Other conditions, e.g. prolonged or shortened extraction times at 37 °C, which simulate the extraction that occurs during clinical use or provide an adequate measure of the hazard potential, may be used, but shall be justified and documented. For medical devices that are in short-term contact (no greater than 4 h cumulative contact duration) with intact skin or mucosa and that are not implanted, this may include extraction times of less than 24 h but no less than 4 h, as given in a) to c).

Cell culture medium with serum should only be used in accordance with a) because extraction temperatures greater than (37 ± 1) °C can adversely impact chemistry and/or stability of the serum and other constituents in the culture medium.

For polymeric test samples, the extraction temperature should not exceed the glass transition temperature as the higher temperature can change the extractant composition.

4.2.3.3 If the extract is filtered, centrifuged or processed by other methods prior to being applied to the cells, these details shall be recorded in the final report along with a rationale for the additional steps (see Clause 9). Any pH adjustment of the extract shall be reported. Manipulation of the extract, such as by pH adjustment, should be avoided because it could influence the result.

4.3 Preparation of material for direct-contact tests

4.3.1 Form of test samples

Materials that have various shapes, sizes or physical states (i.e. liquid, gels, solids, etc.) may be tested without modification in the cytotoxicity assays.

The preferred test sample of a solid material should have at least one flat surface. If not, adjustments shall be made to achieve flat surfaces.

4.3.2 Sterility of test samples

4.3.2.1 Sterility of the test sample shall be taken into account.

4.3.2.2 Test samples from sterilized devices shall be handled aseptically throughout the test procedure.

4.3.2.3 Test samples from devices that are normally supplied non-sterile but are sterilized before use shall be sterilized by the method recommended by the manufacturer and handled aseptically throughout the test procedure.

The effect of sterilization methods or agents on the device should be considered in defining the preparation of the test sample prior to use in the test system.

4.3.2.4 Test samples from devices not required to be sterile in use shall be used as supplied and handled aseptically throughout the test procedure. It may be justifiable to sterilize the test material in order to avoid microbial contamination of the cell culture; however, the sterilization process shall not alter the properties of the test material.

If non-sterile test samples are used, they should be checked for bacterial contamination because the contamination can lead to a false assessment of cytotoxicity.

4.3.3 Liquid test samples

Liquid test samples shall be tested by either

a) direct deposition

or

b) deposition on a biologically inert absorbent matrix.

Filter discs have been found to be suitable for use as inert absorbent matrices.

© ISO 2009 – All rights reserved

4.3.4 Absorbent test samples

If appropriate, test samples that are absorbent shall be soaked with culture medium prior to testing to prevent adsorption of the culture medium in the testing vessel.

4.4 Preparation of controls

Controls should be selected so that they can be prepared by the same procedure as the test sample.

5 Cell lines

Established cell lines are preferred and where used shall be obtained from recognised repositories[3].

Where specific sensitivity is required, primary cell cultures, cell lines and organotypic cultures obtained directly from living tissues shall only be used if reproducibility and accuracy of the response can be demonstrated.

If a stock culture of a cell line is stored, storage shall be at −80 °C or below in the corresponding culture medium but containing a cryoprotectant, e.g. dimethylsulfoxide or glycerol. Long-term storage (several months up to many years) is only possible at −130 °C or below.

Only cells free from mycoplasma shall be used for the test. Before use, stock cultures should be tested for the absence of mycoplasma.

It is important to check cells regularly (e.g. morphology, doubling time, modal chromosome number) because sensitivity in tests can vary with passage number.

Good cell culture practices should be used. See Reference [5].

6 Culture medium

The culture medium shall be sterile.

The culture medium with or without serum shall meet the growth requirements of the selected cell line.

Antibiotics may be included in the medium provided that they do not adversely affect the assays.

Storage conditions shall be validated.

NOTE The stability of the culture medium varies with the composition and storage conditions.

The culture medium shall be maintained at a pH of between 7,2 and 7,4.

---

3) For example, cell lines American Type Culture Collection CCL 1 (NCTC clone 929), CCL 163 (Balb/3T3 clone A31), CCL 171 (MRC-5) and CCL 75 (WI-38), CCL 81 (Vero) and CCL 10 [BHK-21 (C-13)] and V-79 379A are endorsed by ISO experts to be suitable.

This information is given for the convenience of the user of this part of ISO 10993 and does not constitute an endorsement by ISO of the products named. Other cell lines may be used if they can be shown to lead to the same or more relevant results.

© ISO 2009 – All rights reserved

7 Preparation of cell stock culture

Using the chosen cell line and culture medium, prepare sufficient cells to complete the test. If the cells are to be grown from cultures taken from storage, remove the cryoprotectant, if present. Subculture the cells at least once before use.

When subculturing cells, remove and resuspend the cells by enzymatic and/or mechanical disaggregation using a method appropriate for the cell line.

8 Test procedures

8.1 Number of replicates

A minimum of three replicates shall be used for test samples and controls.

8.2 Test on extracts

8.2.1 This test allows both qualitative and quantitative assessment of cytotoxicity.

8.2.2 Pipette an aliquot of the continuously stirred cell suspension into each of a sufficient number of vessels for exposure to the extracts. Distribute the cells evenly over the surface of each vessel by gentle rotation.

8.2.3 Incubate the cultures at (37 ± 1) °C in air with or without carbon dioxide as appropriate for the buffer system chosen for the culture medium.

The test should be performed on a subconfluent monolayer or on freshly suspended cells.

In the colony-forming assay only an appropriate low cell density shall be used.

8.2.4 Verify the subconfluency and the morphology of the cultures with a microscope before starting the test.

In exceptional cases, exponentially growing cells (e.g. primary cells, high proliferating cells) may be seeded at the starting point of the test.

8.2.5 Perform the test on

a) the original extract

and/or

b) the original extract and a dilution series of the extracts using the extract vehicle as diluent.

Alternatively, where materials of limited solubility are known or suspected to be present, dilution should be achieved by varying the original extraction ratio of test sample to extraction medium.

If monolayers are used for the test, remove and discard the culture medium from the cultures and add an aliquot of the extract or dilution thereof into each of the vessels.

If suspended cells are used for the test, add the extract or dilution thereof into each of the replicate vessels, immediately after preparation of the cell suspension.

8.2.6 When a non-physiological extract is used, e.g. water, the extract shall be tested at the highest physiologically compatible concentration after dilution in culture medium.

NOTE Concentrated culture medium, e.g. 2×, 5×, is recommended for use in diluting aqueous extracts.

© ISO 2009 – All rights reserved

8.2.7 Add known aliquots of the blank and the negative and positive controls to additional replicate vessels.

NOTE A fresh culture medium control can also be tested, if appropriate.

8.2.8 Incubate the vessels using the same conditions as described in 8.2.3 for an appropriate interval corresponding to the selected specific assay.

8.2.9 After an incubation period of at least 24 h, determine the cytotoxic effects in accordance with 8.5.

8.3 Test by direct contact

8.3.1 This test allows both qualitative and quantitative assessment of cytotoxicity.

8.3.2 Pipette a known aliquot of the continuously stirred cell suspension into each of a sufficient number of vessels for direct exposure to the test sample. Distribute the cells evenly over the surface of each vessel by gentle horizontal rotation.

8.3.3 Incubate the culture at (37 ± 1) °C in air, with or without carbon dioxide as appropriate for the buffer system chosen for the culture medium, until the cultures have grown to subconfluency.

8.3.4 Verify the subconfluency and the morphology of the cultures with a microscope before starting the test.

In exceptional cases, exponentially growing cells (e.g. primary cells, high proliferating cells) may be seeded at the starting point of the test.

8.3.5 Remove and discard the culture medium. Then add fresh culture medium to each vessel.

8.3.6 Carefully place individual specimens of the test sample on the cell layer in the centre of each of the replicate vessels. Ensure that the specimen covers approximately one tenth of the cell layer surface.

Other ratios of specimen surface to cell layer surface may be used if justified.

Exercise care to prevent unnecessary movement of the specimens, as this could cause physical trauma to the cells. For example, patches of dislodged cells can result from unnecessary movement.

NOTE When appropriate, the specimen can be placed in the culture vessel prior to the addition of the cells.

8.3.7 Prepare replicate vessels for both the negative control and positive control material.

8.3.8 Incubate the vessels under the same conditions as described in 8.3.3 for an appropriate interval (a minimum of 24 h) corresponding to the selected specific assay.

8.3.9 Discard the supernatant culture medium before adding chemicals/dyes in order to determine the cytotoxic effects in accordance with 8.5.

8.4 Test by indirect contact

8.4.1 Agar diffusion

8.4.1.1 This test allows a qualitative assessment of cytotoxicity. This assay is not appropriate for leachables that cannot diffuse through the agar layer, or that may react with agar. The use of the agar diffusion assay for the assessment of cytotoxicity shall be justified.

8.4.1.2 Pipette a known aliquot of the continuously stirred cell suspension into each of a sufficient number of replicate vessels for the test. Distribute the cells evenly over the surface of each vessel by gentle horizontal rotation.

© ISO 2009 – All rights reserved

8.4.1.3 Incubate the cultures at (37 ± 1) °C in air, with or without carbon dioxide as appropriate for the buffer system chosen for the culture medium, until the cultures have grown to approximate subconfluency at the end of the logarithmic phase of the growth curve.

8.4.1.4 Verify the subconfluency and the morphology of the cultures with a microscope before starting the test.

8.4.1.5 Remove and discard the culture medium from the vessel. Then mix fresh culture medium containing serum with melted agar to obtain a final mass concentration of agar of 0,5 % to 2 % and pipette an appropriate volume into each vessel. Use only agar that is suitable for the growth of mammalian cells in culture. The agar/culture medium mixture should be in a liquid state and at a temperature that is compatible with mammalian cells.

NOTE Agar is available in various molecular weight ranges and purities.

8.4.1.6 Carefully place replicate specimens of the test sample on the solidified agar layer in each vessel. Ensure that the specimen covers approximately one tenth of the cell layer surface.

Other ratios of specimen surface to cell layer surface may be used if justified.

Pre-soak any absorbent material with the culture medium before placing it on the agar to prevent dehydration of the agar.

8.4.1.7 Prepare replicate vessels with both the negative control and positive control material.

8.4.1.8 Incubate the vessels using the same conditions as described in 8.4.1.3 for 24 h to 72 h.

8.4.1.9 Examine the cells to determine cytotoxic effect before and after carefully removing the specimens from the agar.

Use of a vital stain, e.g. neutral red, can aid in the detection of cytotoxicity. The vital stain may be added before or after the incubation with the specimen. If the stain is added before the incubation, protect the cultures from light to prevent cell damage elicited by photoactivation of the stain.

8.4.2 Filter diffusion

8.4.2.1 This test allows a qualitative assessment of cytotoxicity.

8.4.2.2 Place a surfactant-free filter with 0,45 µm pore size into each vessel and add a known aliquot of the continuously stirred cell suspension into each of a sufficient number of replicate vessels for the test. Distribute the cells evenly over the surface of each filter by gentle rotation.

8.4.2.3 Incubate the cultures at (37 ± 1) °C in air, with or without carbon dioxide as appropriate for the buffer system chosen for the culture medium, until the cultures have grown to approximate subconfluency at the end of the logarithmic phase of the growth curve.

8.4.2.4 Remove and discard the culture medium from the vessels. Then transfer the filters, cell side down, on to a layer of solidified agar (see 8.4.1.5).

8.4.2.5 Carefully place the replicate specimens of the test sample on the acellular (top) side of the filter. Retain liquid extracts and freshly mixed compounds in non-reactive rings placed on the filter.

8.4.2.6 Prepare replicate filters with both the negative control and positive control material.

8.4.2.7 Incubate the vessels using the same conditions described in 8.4.2.3 for 2 h ± 10 min.

8.4.2.8 Carefully remove the specimens from the filter and carefully separate the filter from the agar surface.

8.4.2.9 Determine the cytotoxic effects using an appropriate stain procedure.

© ISO 2009 – All rights reserved

8.5 Determination of cytotoxicity

8.5.1 Determine cytotoxic effects by either qualitative or quantitative means. Quantitative evaluation of cytotoxicity is preferable. Qualitative means are appropriate for screening purposes.

Qualitative evaluation: Examine the cells microscopically using cytochemical staining if desired. Assess changes in, for example, general morphology, vacuolization, detachment, cell lysis and membrane integrity. The change from normal morphology shall be recorded in the test report descriptively or numerically. A useful way to grade test samples is given in Tables 1 and 2.

Table 1 — Qualitative morphological grading of cytotoxicity of extracts

Grade	Reactivity	Conditions of all cultures
0	None	Discrete intracytoplasmatic granules, no cell lysis, no reduction of cell growth
1	Slight	Not more than 20 % of the cells are round, loosely attached and without intracytoplasmatic granules, or show changes in morphology; occasional lysed cells are present; only slight growth inhibition observable.
2	Mild	Not more than 50 % of the cells are round, devoid of intracytoplasmatic granules, no extensive cell lysis; not more than 50 % growth inhibition observable.
3	Moderate	Not more than 70 % of the cell layers contain rounded cells or are lysed; cell layers not completly destroyed, but more than 50 % growth inhibition observable.
4	Severe	Nearly complete or complete destruction of the cell layers.

Table 2 — Reactivity grades for agar and filter diffusion test and direct contact test

Grade	Reactivity	Description of reactivity zone
0	None	No detectable zone around or under specimen
1	Slight	Some malformed or degenerated cells under specimen
2	Mild	Zone limited to area under specimen
3	Moderate	Zone extending specimen size up to 1,0 cm
4	Severe	Zone extending farther than 1,0 cm beyond specimen

The method of evaluation and the results of the evaluation shall be included in the test report.

The achievement of a numerical grade greater than 2, based on Tables 1 and 2, is considered a cytotoxic effect.

Quantitative evaluation: Measure cell death, inhibition of cell growth, cell proliferation or colony formation. The number of cells, amount of protein, release of enzymes, release of vital dye, reduction of vital dye or any other measurable parameter may be quantified by objective means. The objective measure and response shall be recorded in the test report.

Reduction of cell viability by more than 30 % is considered a cytotoxic effect. Other criteria, including different cut-off points or an acceptable ratio of test-to-control result shall be justified for alternate cell lines or multi-layered tissue constructs. The criteria shall be justified and documented.

The protocols described in Annexes A to D may be used for the quantitative determination of cytotoxicity of extracts.

NOTE Protocols A and B have been proven to be suitable for chemicals in international validation studies and for medical devices in an international round-robin test. For cytotoxic materials, they permit the graduation of the cytotoxic effect by the calculation of an IC_{50} value (inhibitory concentration estimated to affect the endpoint in question by 50 %). Annexes C and D describe other protocols widely used for the quantitative determination of cytotoxicity.

© ISO 2009 – All rights reserved

For particular methods of determining cytotoxicity, a zero time or baseline cell culture control can be necessary.

8.5.2 Ensure that care is taken in the choice of evaluation methods, as the test results can be invalid if the test sample releases substances that interfere with the test system or measurement.

Materials that can release formaldehyde can only be reliably tested when cell viability is evaluated.

8.5.3 If there are evident differences in the test result for replicate culture vessels, then the test is either inappropriate or invalid. In this case, the test shall be repeated, or an alternative methodology used.

8.5.4 If the negative, positive and any other controls (reference, medium, blank, reagent, etc.) do not have the expected response in the test system, then repeat the entire assay(s).

9 Test report

The test report shall include at least the following details:

a) name and address of testing facility;

b) name of the person(s) who conducted the test;

c) dates of start and end of the test;

d) description of the sample;

e) cell line, justification of the choice and cell source(s);

f) name of company and batch of medium, serum and antibiotics, when added;

g) assay method and rationale;

h) extraction procedure (if appropriate) and, if possible, the nature and concentration of the leached substance(s);

i) negative, positive and other controls;

j) cell response and other observations;

k) any other relevant data necessary for the assessment of results.

© ISO 2009 – All rights reserved

10 Assessment of results

The overall assessment of the results shall be carried out by a person capable of making informed decisions based on the test data. Cytotoxicity data shall be assessed in relation to other biocompatibility data and the intended use of the product.

The interpretation of the results of the cytotoxicity test shall take into account the classification of the device as given in ISO 10993-1.

If there is a cytotoxic effect, further evaluation can be performed, for example:

a) additional tests (presence/absence of serum, changing of the level of serum in the culture medium);

b) extract analysis (e.g. residues from sterilization and other production processes), where appropriate;

c) concentration response analysis of dilutions;

d) chemical characterization of leachable components,

e) other test procedures.

Any cytotoxic effect can be of concern. However, it is primarily an indication of potential for *in vivo* toxicity and the device cannot necessarily be determined to be unsuitable for a given clinical application based solely on cytotoxicity data.

© ISO 2009 – All rights reserved

Annex A
(informative)

Neutral red uptake (NRU) cytotoxicity test

A.1 General

The following test protocol is based on, and describes only, those parts of Annex C of Reference [1], which are relevant for this test.

A.2 Experimental procedure

A.2.1 Basic procedure

BALB/c 3T3 cells are seeded into 96-well plates and maintained in culture for 24 h (~ 1 doubling period) to form a semi-confluent monolayer (see Reference [5] for more information on cell maintenance and culture procedures). They are then exposed to the test compound over a range of concentrations. After 24 h exposure, NRU is determined for each treatment concentration and compared to that determined in control cultures. For each treatment (i.e. concentration of the test chemical), the inhibition of growth percentage is calculated, if the extract exhibits a cytotoxic effect on the cells. The IC_{50} (i.e. the concentration producing 50 % reduction of NRU) is calculated from the concentration-response and expressed as a dilution percentage of the extract. The neat extract is designated as 100 % extract.

A.2.2 Material

A.2.2.1 Cell line

BALB/c 3T3 cells, clone 31 (e.g. ECACC86110401, European Collection of Cell Cultures, Salisbury, Wiltshire SP4 0JG, UK; CCL-163, American Type Culture Collection [ATCC], Manassas, VA, USA) and JCRB 9005, prepared from CCL-163[ATCC], Human Science Research Resources Bank, Osaka, Japan.

A.2.2.2 Technical equipment

A.2.2.2.1 Incubator, 37 °C, humidified, 5 % CO_2/air [alternatively, in the absence of a suitable buffer in the cell culture medium, 7,5 % CO_2/air may be used because cells are very sensitive to pH changes; however 5 % is more commonly used in most laboratories, while HEPES [acid 4-(2-hydroxyethyl)-1-piperazineethanesulfonic acid] is added for better buffering.

A.2.2.2.2 Laminar flow cabinet, standard: “biological hazard”.

A.2.2.2.3 Water bath, 37 °C.

A.2.2.2.4 Inverse phase contrast microscope.

A.2.2.2.5 Laboratory burner.

A.2.2.2.6 Centrifuge, optionally equipped with microtitre plate rotor.

A.2.2.2.7 Laboratory balance.

A.2.2.2.8 96-well plate photometer, equipped with 540 nm filter.

© ISO 2009 – All rights reserved

A.2.2.2.9 **Shaker**, for microtitre plates.

A.2.2.2.10 **Cell counter** or **hemacytometer**.

A.2.2.2.11 **Pipetting aid**.

A.2.2.2.12 **Pipettes, 8-channel pipettes, dilution block**.

A.2.2.2.13 **Cryotubes**.

A.2.2.2.14 **Tissue culture flasks**, 80 cm^2, 25 cm^2.

A.2.2.2.15 **96-well tissue culture microtitre plates**.

A.2.2.3 Chemicals, media and sera

A.2.2.3.1 **Dulbecco's Modification of Eagle's Medium (DMEM)**, without L-glutamine

A.2.2.3.2 **L-glutamine**, 200 mM, or **glutamax**.

A.2.2.3.3 **Newborn calf serum (NBCS)**.

IMPORTANT — Foetal calf serum (FCS) shall not be used. FCS causes a strongly reduced O.D. due to the formation of vacuoles in the cells.

Due to lot variability of NBCS, first check a lot for growth-stimulating properties with 3T3 cells (20 h to 25 h doubling time) and then reserve a sufficient amount of NBCS.

A.2.2.3.4 **Trypsin/EDTA solution**.

A.2.2.3.5 **Phosphate-buffered saline (PBS)**, without Ca^{2+} and Mg^{2+} (for trypsinization).

A.2.2.3.6 **HEPES** (see A.2.2.2.1).

A.2.2.3.7 **PBS**, with Ca^{2+} and Mg^{2+} (for rinsing).

A.2.2.3.8 **Penicillin/streptomycin solution**.

A.2.2.3.9 **Neutral red (NR)**.

A.2.2.3.10 **Dimethyl sulfoxide (DMSO)**, analytical grade.

A.2.2.3.11 **Ethanol (ETOH)**, analytical grade.

A.2.2.3.12 **Glacial acetic acid**, analytical grade.

A.2.2.3.13 **Distilled water** or **any purified water suitable for cell culture**.

A.2.2.4 Preparations

A.2.2.4.1 General

All solutions (except NR stock solution, NR medium and NR desorb), glassware, etc., shall be sterile and all procedures should be carried out under aseptic conditions and in the sterile environment of a laminar flow cabinet (biological hazard standard).

© ISO 2009 – All rights reserved

A.2.2.4.2 Media

DMEM (buffered with sodium bicarbonate) supplemented with (final concentrations in DMEM are quoted):

(A) For freezing

— 20 % NBCS

— 7 % to 10 % DMSO

(B) For routine culture

— 10 % NBCS

— 4 mM L-glutamine or glutamax

— 100 IU/ml penicillin

— 100 µg/ml streptomycin

— 20 mM HEPES

(C) For treatment with test samples

— 5 % NBCS

— 4 mM glutamine or glutamax

— 100 IU/ml penicillin

— 100 µg/ml streptomycin

— 20 mM HEPES

Complete media should be kept at 4 °C and stored for no longer than two weeks.

The serum concentration of treatment medium is reduced to 5 %, since serum proteins can mask the toxicity of the test substance. Serum cannot be totally excluded because cell growth is markedly reduced in its absence.

A.2.2.4.3 Neutral red (NR) stock solution

— 0,4 g NR dye

— 100 ml H_2O

Make up prior to use and store in the dark at room temperature for up to two months. Commercially prepared neutral red stock solutions may be used up to their expiration dates when stored according to the label.

 © ISO 2009 – All rights reserved

A.2.2.4.4 Neutral red (NR) medium

— 1 ml NR stock solution

— 79 ml DMEM

The NR medium should be incubated overnight at 37 °C and centrifuged at 600g for 10 min (to remove NR crystals) before adding to the cells. Alternative procedures (e.g. millipore filtering) can be used as long as they guarantee that the NR medium is free of crystals. Aliquots of the NR medium should be maintained at 37 °C (e.g. in a water bath) before being added to the cells. They should be used within 30 min of preparation and within 15 min of removing from 37 °C storage.

A.2.2.4.5 Ethanol/acetic acid solution (NRdesorb)

— 1 % glacial acetic acid solution

— 50 % ethanol

— 49 % H_2O

Prepare immediately prior to use. Do not store for longer than 1 h.

A.2.2.4.6 Preparation of sample extract

Samples are extracted in accordance with ISO 10993-12.

A.2.3 Methods

A.2.3.1 General

For routine cell culture methods, see Annex C of Reference [1].

A.2.3.2 Quality check of assay (I); positive control (PC)

A positive control shall be included in every cytotoxicity test.

With regard to the many chemicals backed by sufficient history or intra- and interlaboratory repeat tests, sodium lauryl sulfate (SLS, CAS # 151-21-3) is one of the most frequently tested, and is therefore recommended as a PC. It is recommended that SLS be tested in a four-concentration scale: 0,05 mg/ml; 0,1 mg/ml; 0,15 mg/ml; 0,2 mg/ml.

The historical mean, IC_{50}, of SLS (Spielmann et. al., 1991[10]) is 0,093 mg/ml.

The 95 % CI (confidence interval) is 0,070 mg/ml to 0,116 mg/ml.

A test meets acceptance criteria, if the IC_{50} for SLS is within the 95 % CI.

The use of positive reference materials and negative reference materials is recommended [e.g. ZDEC and ZDBC (see footnote 1 on page 2 and 3.2 and 3.4)].

A.2.3.3 Quality check of assay (II); blank

The absolute value (not relative to the blank) of optical density (OD_{540} of NRU) obtained in the untreated blank indicates whether the 1×10^4 cells seeded per well have grown exponentially with normal doubling time during the two days of the assay.

A test meets the acceptance criteria if the mean OD_{540} of blanks is $\geqslant$ 0,3.

© ISO 2009 – All rights reserved

To check for systematic cell seeding errors, blanks are treated under extraction conditions (see A.2.2.4.6) and are placed both at the left side (row 2) and the right side (row 11) of the 96-well plate (row 1 and row 12 shall not be used; for plate layout, see Annex E in Reference [1]).

A test meets acceptance criteria if the left and the right mean of the blanks do not differ by more than 15 % from the mean of all blanks.

Checks for cell seeding errors may also be performed by examining each plate under a phase contrast microscope to ensure that cell quantity is consistent. Microscopic evaluation obviates the need for two rows of blanks.

A.2.3.4 Quality check of concentration-response

The IC_{50} derived from the concentration-response should be supported by at least two, or if possible, three responses between 10 % and 90 % inhibition of NRU. If this is not the case, and the concentration progression factor can be easily reduced, reject the experiment and repeat it with a smaller progression factor.

A.2.3.5 Concentrations of test sample extracts

A.2.3.5.1 Range finder experiment

Test eight concentrations of the sample extract by diluting the stock solution with a constant factor, covering a large range, e.g. half-log intervals. If the reduction of viability of the cell culture with the highest concentration of the sample extract is 30 % or less, then the material has to be considered non-cytotoxic and no further main experiment is necessary.

A.2.3.5.2 Main experiment

Depending on the slope of the concentration-response curve estimated from the range finder, the dilution/progression factor in the concentration series of the main experiment should be smaller (e.g. $\sqrt[6]{10} = 1{,}47$). Try to cover the relevant concentration range (between 10 % and 90 % effect) with at least three points of a graded effect, avoiding too many non-cytotoxic and/or 100 %-cytotoxic concentrations.

A.2.3.6 Test procedure

IMPORTANT — After thawing from stock, passage two to three times before using the cells in the test.

Table A.1 shows the work flow of the test procedure.

1st day

— Prepare a cell suspension of 1×10^5 cells/ml in culture medium. Using a multichannel pipette, dispense 100 µl culture medium only into the peripheral wells of a 96-well tissue culture microtitre plate (= blanks, see Appendix E in Reference [1]). In the remaining wells, dispense 100 µl of a cell suspension of 1×10^5 cells/ml (= 1×10^4 cells/well). Prepare one plate per sample extract to be tested, one plate for the PC and one plate for the negative control material if available.

— Incubate cells for 24 h (5 % CO_2, 37 °C, > 90 % humidity) so that cells form a half-confluent monolayer. This incubation period ensures cell recovery, and adherence and progression to exponential growth phase.

— Examine each plate under a phase contrast microscope to ensure that cell growth is relatively even across the microtitre plate. This check is performed to identify experimental errors.

 © ISO 2009 – All rights reserved

2nd day

— After 24 h incubation, aspirate culture medium from the cells.

— Per well, add 100 µl of treatment medium containing either the appropriate concentration of sample extract, or the negative control, or the PC or nothing but vehicle (blank).

— Incubate cells for 24 h (5 % CO_2, 37 °C, > 90 % humidity).

3rd day

After 24 h treatment, examine each plate under a phase contrast microscope to identify systematic cell seeding errors and growth characteristics of control and treated cells. Record changes in morphology of the cells due to cytotoxic effects of the test sample extract, but do not use these records for the calculation of the highest tolerable dose (HTD) or any other quantitative measure of cytotoxicity. Undesirable growth characteristics of control cells can indicate experimental error and can be cause for rejection of the assay.

The measurement of NRU is based upon that of Ellen Borenfreund (Borenfreund and Puerner[3]). The uptake of NR into the lysosomes/endosomes and vacuoles of living cells is used as a quantitative indication of cell number and viability.

— Wash the cells with 150 µl pre-warmed PBS. Remove the washing solution by gentle tapping. Add 100 µl NR medium and incubate at 37 °C in a humidified atmosphere of 5 % CO_2 for 3 h.

— After incubation, remove the NR medium, and wash cells with 150 µl PBS.

— Decant and blot PBS totally (optionally, centrifuge the reversed plate).

— Add 150 µl NR desorb (ETOH/acetic acid) solution to all wells, including blanks.

— Shake the microtitre plate rapidly on a microtitre plate shaker for 10 min until NR has been extracted from the cells and forms a homogeneous solution.

— Measure the absorption of the resulting coloured solution at 540 nm in a microtitre plate reader, using the blanks as a reference. Save raw data in a file format (e.g. ASCII, TXT, XLS) appropriate for further analysis of the concentration-response and calculation of IC_{50}.

A.2.4 Data analysis

A calculation of cell viability expressed as NRU is made for each concentration of the test sample extract by using the mean NRU of the six replicate values per test concentration. This value is compared with the mean NRU of all blank values (provided blanks have met the blank acceptance criteria). Relative cell viability is then expressed as a percentage of untreated blank. If achievable, the eight concentrations of each compound tested should span the range of no effect up to total inhibition of cell viability.

© ISO 2009 – All rights reserved

Table A.1 — 3T3 NRU cytotoxicity test work flow

Time<br>h	Procedure
00:00	Seed 96-well plates: 1×10^4 cells/100 µl DMEM culture medium/well<br>Incubate (37 °C/5 % CO_2/22 h to 24 h)<br>↓
24:00	Remove culture medium<br>↓
24:00	Treat with eight concentrations of test sample extract in treatment medium (100 µl)<br>(untreated blank = treatment medium)<br>Incubate (37 °C/5 % CO_2/24 h)<br>↓
48:00	Microscopic evaluation of morphological alterations<br>Remove treatment medium<br>Wash once with 150 µl PBS<br>Add 100 µl NR medium<br>Incubate (37 °C/5 % CO_2/3 h)<br>↓
51:00	Discard NR medium<br>Wash once with 150 µl PBS<br>Add 150 µl NR desorbing fixative<br>(ETOH/acetic acid solution)<br>↓
51:40	Shake plate for 10 min
51:50	Detect NR absorption at 540 nm (i.e. cell viability)

If the relative cell viability for the highest concentration of the sample extract (100 % extract) is less than 70 % of the control group, the concentration of a test chemical reflecting a 50 % inhibition of cell viability (i.e. IC_{50}) is determined from the concentration-response. This can be done either by applying

— a manual graphical fitting method,

The use of probability paper with "X = log" and "Y = probit" scales is recommended because in most cases the concentration-response function will become almost linear in the relevant range. Semi-log paper could also be used for this technique.

or

— any appropriate non-linear regression procedure (preferably a Hill function[4)] or a logistic regression) to the concentration-response data.

Before using the IC_{50} for further calculations, the quality of the fit should be appropriately checked.

If the relative cell viability for the highest concentration of the sample extract (100 % extract) is $\geqslant$ 70 % of the control group, then the material shall be considered non-cytotoxic.

4) Hill functions are monotonous and sigmoidal in shape and represent an acceptable model for many dose-response curves.

© ISO 2009 – All rights reserved

Annex B
(informative)

Colony formation cytotoxicity test

B.1 General

The test protocol is based on Part I of the cytotoxicity test of the Japanese guidelines for basic biological tests of medical materials and devices, see Reference [2].

NOTE The following protocol describes only those sections of Part I relevant to the cytotoxicity test.

B.2 Experimental procedure

B.2.1 Basic procedure

V79 cells are seeded into six-well plates and maintained in culture for 24 h to start growing in a logarithmic phase. They are then exposed to the test compound over a range of concentrations. They are incubated for six days to make colonies large enough to count. Colonies are fixed with methanol, stained with Giemsa solution, and counted. If the extract exhibits a cytotoxic effect on the cells, the IC_{50} (the concentration inhibiting plating efficiency to 50 %) is calculated and expressed as a percentage of the extract.

B.2.2 Material

B.2.2.1 Cell Line

V79 cells (JCRB 0603, Human Science Research Resources Bank, Osaka, Japan, available from other cell banks of USA and EU).

NOTE V79 cells are recommended because they make large and clear colonies.

B.2.2.2 Technical equipment

B.2.2.2.1 Incubator, 37 °C, humidified, 5 % CO_2/air.

B.2.2.2.2 Laminar flow cabinet, standard: “biological hazard”.

B.2.2.2.3 Water bath, 37 °C.

B.2.2.2.4 Inverted phase contrast microscope.

B.2.2.2.5 Stereomicroscope.

B.2.2.2.6 Laboratory burner.

B.2.2.2.7 Laboratory balance.

B.2.2.2.8 Cell counter or **hemocytometer**.

B.2.2.2.9 Pipetting aid.

B.2.2.2.10 Pipettes.

© ISO 2009 – All rights reserved

B.2.2.2.11 Tissue culture flasks, 75 cm^2, 25 cm^2 or **tissue culture dish**, 100 mm diameter.

B.2.2.2.12 6-well tissue culture microtitre plates.

B.2.2.3 Chemicals, media and sera

B.2.2.3.1 Eagle minimum essential medium (MEM), containing Earle's balanced salt solution.

B.2.2.3.2 Foetal calf serum (FCS).

NOTE Due to lot variability of FCS, first check a lot for growth-stimulating properties with V79 cells and then reserve sufficient amount of FCS.

B.2.2.3.3 Trypsin/EDTA solution.

B.2.2.3.4 Phosphate-buffered saline (PBS), without Ca^{2+} and Mg^{2+} (for trypsinization).

B.2.2.3.5 Penicillin/streptomycin solution.

B.2.2.3.6 Dimethyl sulfoxide (DMSO), analytical grade.

B.2.2.3.7 Methanol, analytical grade.

B.2.2.3.8 Giemsa solution.

B.2.2.3.9 Phosphate-buffered solution.

B.2.2.3.10 Distilled water or **any purified water suitable for cell culture**.

B.2.2.3.11 Sodium bicarbonate.

B.2.2.3.12 L-glutamine.

B.2.2.3.13 MEM non-essential amino-acids solution.

B.2.2.3.14 Sodium pyruvate, 100 mM.

B.2.2.4 Preparations

B.2.2.4.1 General

All solutions (except Giemsa solution), glassware, etc., shall be sterile and all procedures should be carried out under aseptic conditions and in the sterile environment of a laminar flow cabinet (biological hazard standard).

B.2.2.4.2 Media

1 000 ml of Eagle MEM supplemented as follows:

(A) For freezing and routine culture, medium with 10 % FCS is used (MEM 10)

— 111 ml FCS

— 2,2 g sodium bicarbonate

— 0,292 g L-glutamine

© ISO 2009 – All rights reserved

(B) For extraction and treatment, medium with 5 % FCS is used (MEM 05)

- 53,5 ml FCS
- 5 ml MEM non-essential amino-acids solution
- 10 ml sodium pyruvate, 100 mM
- 0,292 g L-glutamine
- 2,2 g sodium bicarbonate

Complete media should be kept at 4 °C and stored for no longer than 1 month.

The serum concentration of treatment medium is reduced to 5 %, since serum proteins can mask the toxicity of the test substance. Serum cannot be totally excluded because cell growth is markedly reduced in its absence. An appropriate amount of antibiotics may be added in the media above unless they adversely affect the assay.

B.2.2.4.3 5 % Giemsa solution

- 5 ml Giemsa solution
- 95 ml phosphate-buffered solution, any kind, pH 6,5 to pH 7,5

Prepare just before use.

B.2.2.4.4 Preparation of sample extract

Samples are extracted in accordance with ISO 10993-12, but extraction ratio of surface area per extraction volume is recommended at 6 cm^2/ml and that of mass per extraction volume is recommended at 0,1 g/ml. See References [6], [7] and [11].

The extract is separated by decantation, being the designated 100 % extract. The 100 % extract is diluted with MEM05 medium to give the various percentages of diluted extract.

B.2.3 Methods

B.2.3.1 General

For routine cell culture methods, see Annex C of Reference [1].

B.2.3.2 Quality check of assay (I); positive control (PC) and negative control (NC)

Positive and negative controls shall be included in every cytotoxicity test. Positive and negative reference materials are recommended, e.g. ZDEC and ZDBC (see footnote 1 on page 2).

The following acceptance criteria should be used for ZDEC and ZDBC:

a) IC_{50} for ZDEC should not exceed 7 %;

b) IC_{50} for ZDBC should not exceed 80 %.

B.2.3.3 Quality check of assay (II); blank

A test meets acceptance criteria if the average number of colonies in the blank is at least 70 % of the number of cells plated per well.

© ISO 2009 – All rights reserved

B.2.3.4 Quality check of concentration-response

The IC_{50} derived from the concentration-response should be backed by at least two, or if possible, three responses between 10 % and 90 % inhibition of the control. If this is not the case, and the concentration progression factor can be easily reduced, reject the experiment and repeat it with a smaller progression factor.

B.2.3.5 Concentrations of test sample extracts

B.2.3.5.1 Range finder experiment

Test four concentrations of the sample extract by diluting the stock solution with a constant factor, covering a large range, e.g. $1 \geqslant 1/10 \geqslant 1/100 \geqslant 1/1\,000$. If the reduction of viability of the cell culture with the highest concentration of the sample extract is 30 % or less, then the material has to be considered non-cytotoxic and no further main experiment is necessary.

B.2.3.5.2 Main experiment

Depending on the slope of the concentration-response curve estimated from the range finder, the dilution/progression factor in the concentration series of the main experiment should be smaller. Try to cover the relevant concentration range (between 10 % and 90 % effect) with at least three points of a graded effect, avoiding too many non-cytotoxic and/or 100 %-cytotoxic concentrations.

B.2.3.6 Test procedure

IMPORTANT — After thawing from stock, passage two to three times before using the cells in the test.

1st day

Prepare a cell suspension of 33,3 cells/ml in MEM05 medium. Place 3 ml of the suspension in each well of a 6-well plate.

2nd day

After 24 h incubation, aspirate culture medium from each well. Add 2 ml of 100 % or diluted extract prepared with MEM05 medium. Test each concentration in triplicate. Incubate them in the incubator for another 6 days.

8th day

Aspirate the treatment medium from each well, rinse the well with PBS, fix the colonies with methanol, and stain them with 5 % Giemsa solution. Count the number of colonies in each well.

B.2.3.7 Presentation of results

- Observe the wells using a stereomicroscope and count the number of colonies consisting of 50 or more cells in each well.
- Record the number of colonies in a well.
- Calculate the average colony number for each extract concentration.
- Divide this number by that of the control group and express the quotient (plating efficiency: PE) as a percentage.
- If the plating efficency for the highest concentration of the sample extract (100 % extract) is less than 70 % of the control group, the material shall be considered potentially cytotoxic and the cytotoxicity of the test samples is expressed with the IC_{50} value as a percentage.

© ISO 2009 – All rights reserved

— IC_{50} (the concentration inhibiting PE to 50 %) is calculated as the dose with a 50 % relative survival rate, which is calculated from the line that passed through a dose with higher survival and a dose with lower survival than 50 %.

— If the plating efficency is $\geqslant$ 70 % of the control group, the material shall be considered non-cytotoxic.

NOTE When the plating efficiency at the highest concentration is between 50 % and 70 %, it might not be practical to calculate the IC_{50} value over the highest concentration.

B.2.4 Test report

The test report shall be prepared according to Clause 9; additionally the following shall be given:

a) control PE in the given conditions;

b) all test data, including the colony number, the colony formation inhibition curves and, if possible, IC_{50} values obtained for the test samples.

© ISO 2009 – All rights reserved

Annex C
(informative)

MTT cytotoxicity test

C.1 General

The following test protocol is based on the measurement of the viability of cells via metabolic activity, see Reference [9]. Yellow water-soluble MTT (3-(4,5-dimethylthiazol-2-yl)-2,5-diphenyltetrazoliumbromid) is metabolically reduced in viable cells to a blue-violet insoluble formazan. The number of viable cells correlates to the colour intensity determined by photometric measurements after dissolving the formazan in alcohol.

C.2 Experimental procedure

C.2.1 Basic procedure

L929 cells are seeded into 96-well plates and maintained in culture for 24 h (~ 1 doubling period) to form a semi-confluent monolayer (see Reference [5] for more information on cell maintenance and culture procedures). They are then exposed to the test compound over a range of concentrations. After 24 h exposure, the formazan formation is determined for each treatment concentration and compared to that determined in control cultures. For each treatment the percentage inhibition of growth is calculated.

C.2.2 Material

C.2.2.1 Cell line

L-929 cells (NCTC clone 929: CCL 1, American Type Culture Collection [ATCC], Manassas, VA, USA; ECACC No. 88102702, European Collection of Cell Cultures, Salisbury, Wiltshire SP4 0JG, UK). Cell cultures shall be free of mycoplasma.

C.2.2.2 Technical equipment

C.2.2.2.1 Incubator, 37 °C, humidified, 5 % CO_2/air.

C.2.2.2.2 Laminar flow cabinet, standard: “biological hazard”.

C.2.2.2.3 Water bath, 37 °C.

C.2.2.2.4 Inverse phase contrast microscope.

C.2.2.2.5 Laboratory burner.

C.2.2.2.6 Centrifuge, optionally equipped with microtitre plate rotor.

C.2.2.2.7 Laboratory balance.

C.2.2.2.8 96-well plate photometer, equipped with 570 nm filter (reference 650 nm).

C.2.2.2.9 Shaker, for microtitre plates.

C.2.2.2.10 Cell counter or **hemacytometer**.

© ISO 2009 – All rights reserved

C.2.2.2.11 Pipetting aid.

C.2.2.2.12 Pipettes, 8-channel pipettes, dilution block.

C.2.2.2.13 Cryotubes.

C.2.2.2.14 Tissue culture flasks or **tissue culture petri dishes**.

C.2.2.2.15 96-well tissue culture microtitre plates.

C.2.2.3 Chemicals, media and sera

C.2.2.3.1 Eagle minimum essential medium (MEM), without phenol red, without glutamine and without $NaHCO_3$.

C.2.2.3.2 Foetal calf serum (FCS).

C.2.2.3.3 Trypsin/EDTA solution.

C.2.2.3.4 Phosphate-buffered saline (PBS).

C.2.2.3.5 MTT (3-(4,5-dimethylthiazol-2-yl)-2,5-diphenyltetrazoliumbromid).

C.2.2.3.6 Isopropanol, analytical grade.

C.2.2.4 Preparations

C.2.2.4.1 General

All solutions, glassware, etc., shall be sterile and all procedures should be carried out under aseptic conditions and in the sterile environment of a laminar flow cabinet (biological hazard standard).

C.2.2.4.2 Media

MEM (buffered with sodium bicarbonate) supplemented with (final concentrations in MEM are quoted):

(A) For freezing

— 20 % FCS

— 7 % to 10 % DMSO

(B) For routine culture

— 10 % FCS

— 4 mM glutamine or glutamax

— 100 IU/ml penicillin

— 100 µg/ml streptomycin

Complete media should be kept at 4 °C and stored for no longer than two weeks.

© ISO 2009 – All rights reserved

C.2.2.4.3 MTT Solution

MTT is soluted fresh in MEM without supplements and without phenol red at a concentration of 1 mg/ml. Solution is sterilized by sterile filtration using syringe filters (pore size $\leqslant$ 0,22 µm). The solution should be used the same day.

C.2.2.4.4 Preparation of sample extract

Samples are extracted in accordance with ISO 10993-12.

C.2.3 Methods

C.2.3.1 General

For routine cell culture methods, see Annex C of Reference [1].

C.2.3.2 Quality check of assay (I); positive control (PC) and negative control (NC)

Positive and negative controls should be included in every cytotoxicity test. Positive and negative reference materials are recommended, e.g. ZDEC and ZDBC (see footnote 1 on page 2).

C.2.3.3 Quality check of assay (II); blank

The absolute value of optical density, OD_{570}, obtained in the untreated blank indicates whether the 1×10^4 cells seeded per well have grown exponentially with normal doubling time during the two days of the assay.

A test meets the acceptance criteria if the mean OD_{570} of blanks is $\geqslant$ 0,2.

To check for systematic cell seeding errors, blanks are placed both at the left side (row 2) and the right side (row 11) of the 96-well plate (row 1 and row 12 shall not be used; for plate layout, see Annex E in Reference [1]).

A test meets acceptance criteria if the left and the right mean of the blanks do not differ by more than 15 % from the mean of all blanks.

Checks for cell seeding errors may also be performed by examining each plate under a phase contrast microscope to ensure that cell quantity is consistent. Microscopic evaluation obviates the need for two rows of blanks.

C.2.3.4 Test procedure

IMPORTANT — After thawing from stock, passage two to three times before using the cells in the test.

Table C.1 shows the work flow of the test procedure.

1st day after growing up the cells from frozen stock

— Cell cultures are removed from culture flasks by enzymatic digestion (trypsin/EDTA) and the cell suspension is centrifuged (200 g, 3 min). The cells are then resuspended in culture medium and the cell suspension is adjusted at a density of 1×10^5 cells/ml. Using a multichannel pipette, dispense 100 µl culture medium only (blank) into the peripheral wells of a 96-well tissue culture microtitre plate (= blanks, see Appendix E in Reference [1]). In the remaining wells, dispense 100 µl of a cell suspension of 1×10^5 cells/ml (= 1×10^4 cells/well).

— Incubate cells for 24 h (5 % CO_2, 37 °C, > 90 % humidity) so that cells form a half-confluent monolayer. This incubation period ensures cell recovery, and adherence and progression to exponential growth phase.

— Examine each plate under a phase contrast microscope to ensure that cell growth is relatively even across the microtitre plate. This check is performed to identify experimental errors.

© ISO 2009 – All rights reserved

2nd day

— After 24 h incubation, aspirate culture medium from the cells.

— Per well, add 100 µl of treatment medium containing either the appropriate concentration of sample extract, or the negative control, or the PC, or nothing but blank. At least four different concentrations of the test item extract or the positive control extract should be tested. The highest concentration used should be 100 % extract and the other concentrations will be adequately spaced within a single logarithmic range. For the negative control, only the 100 % extract should be tested. Culture medium should be used as blank.

— Incubate cells for 24 h (5 % CO_2, 37 °C, > 90 % humidity).

3rd day

After 24 h treatment, examine each plate under a phase contrast microscope to identify systematic cell seeding errors and growth characteristics of control and treated cells. Record changes in the morphology of the cells due to cytotoxic effects of the test sample extract, but do not use these records for any quantitative measure of cytotoxicity. Undesirable growth characteristics of control cells can indicate experimental error and can be cause for rejection of the assay.

After the examination of the plates, carefully remove the culture medium from the plates. This is an important step, because reductive chemicals in the extract can also reduce MTT, causing false negative results. 50 µl of the MTT solution (see C.2.2.4.3) is then added to each test well and the plates are further incubated for 2 h in the incubator at 37 °C. Then the MTT solution is discanted and 100 µl of isopropanol are added in each well. Sway this plate and subsequently transfer it to a microplate reader equipped with a 570 nm filter to read the absorbance (reference wavelength 650 nm).

Table C.1 — MTT cytotoxicity test work flow

Time<br>h	Procedure
00:00	Seed 96-well plates: 1×10^4 cells/100 µl MEM culture medium/well<br>Incubate (37 °C/5 % CO_2/22 h to 26 h)<br>↓
24:00	Remove culture medium<br>↓
24:00	Treat with ⩾ 4 concentrations of test sample extract in treatment medium (100 µl)<br>(untreated blank = treatment medium)<br>Incubate (37 °C/5 % CO_2/24 h)<br>↓
48:00	Microscopic evaluation of morphological alterations<br>Remove culture medium<br>Add 50 µl MTT solution<br>Incubate (37 °C/5 % CO_2/2 h)<br>↓
51:00	Remove MTT solution<br>Add 100 ml isopropanol to each well<br>Sway plate<br>↓
51:30	Detect absorption at 570 nm (reference 650 nm)

© ISO 2009 – All rights reserved

C.2.4 Data recording

The data generated will be recorded in the raw data file. The results will be presented in tabular form, including experimental groups with the test item, negative, blank and positive controls.

C.2.5 Data analysis

A decrease in number of living cells results in a decrease in the metabolic activity in the sample. This decrease directly correlates to the amount of blue-violet formazan formed, as monitored by the optical density at 570 nm. To calculate the reduction of viability compared to the blank Equation (C.1) is used:

$$\text{Viab. \%} = \frac{100 \times OD_{570\text{e}}}{OD_{570\text{b}}} \qquad \text{(C.1)}$$

where

$OD_{570\text{e}}$ is the mean value of the measured optical density of the 100 % extracts of the test sample;

$OD_{570\text{b}}$ is the mean value of the measured optical density of the blanks.

The lower the Viab.% value, the higher the cytotoxic potential of the test item is.

If viability is reduced to $<$ 70 % of the blank, it has a cytotoxic potential. The 50 % extract of the test sample should have at least the same or a higher viability than the 100 % extract; otherwise the test should be repeated.

© ISO 2009 – All rights reserved

Annex D
(informative)

XTT cytotoxicity test

D.1 General

The following test protocol is based on the measurement of the viability of cells via mitochondrial dehydrogenases, see Reference [9].

XTT (2,3-bis(2-methoxy-4-nitro-5-sulfophenyl)-5-[(phenylamino)carbonyl] -2H-tetrazolium hydroxide) is metabolically reduced in viable cells to a water-soluble formazan product. The number of viable cells correlates to the colour intensity determined by photometric measurements.

D.2 Experimental procedure

D.2.1 Basic procedure

L929 cells are seeded into 96-well plates and maintained in culture for 24 h (~ 1 doubling period) to form a semi-confluent monolayer (see Reference [5] for more information on cell maintenance and culture procedures). They are then exposed to the test compound over a range of concentrations. After 24 h exposure, the formazan formation is determined for each treatment concentration and compared to that determined in control cultures. For each treatment the percentage inhibition of growth is calculated.

D.2.2 Material

D.2.2.1 Cell line

L929 cells (NCTC clone 929: CCL 1, American Type Culture Collection [ATCC], Manassas, VA, USA; ECACC No. 88102702, European Collection of Cell Cultures, Salisbury, Wiltshire SP4 OJG, UK). Cell cultures have to be free of mycoplasma.

D.2.2.2 Technical equipment

D.2.2.2.1 **Incubator**, 37 °C, humidified, 5 % CO_2/air.

D.2.2.2.2 **Laminar flow cabinet**, standard: “biological hazard”.

D.2.2.2.3 **Water bath**, 37 °C.

D.2.2.2.4 **Inverse phase contrast microscope**.

D.2.2.2.5 **Laboratory burner**.

D.2.2.2.6 **Centrifuge**, optionally equipped with microtitre plate rotor.

D.2.2.2.7 **Laboratory balance**.

D.2.2.2.8 **96-well plate photometer**, equipped with 450 nm filter (reference 650 nm).

D.2.2.2.9 **Shaker**, for microtitre plates.

© ISO 2009 – All rights reserved

D.2.2.2.10 Cell counter or **hemacytometer**.

D.2.2.2.11 Pipetting aid.

D.2.2.2.12 Pipettes, 8-channel pipettes, dilution block.

D.2.2.2.13 Cryotubes.

D.2.2.2.14 Tissue culture flasks or **tissue culture petri dishes**.

D.2.2.2.15 96-well tissue culture microtitre plates.

D.2.2.3 Chemicals, media and sera

D.2.2.3.1 Eagle minimum essential medium (MEM), without phenol red, without glutamine and without $NaHCO_3$.

D.2.2.3.2 Foetal calf serum (FCS).

D.2.2.3.3 Trypsin/EDTA solution.

D.2.2.3.4 Phosphate-buffered saline (PBS).

D.2.2.3.5 XTT (2,3-bis[2-methoxy-4-nitro-5-sulfophenyl]-2H-tetrazolium-5-carboxyaniline inner salt).

D.2.2.3.6 PMS (phenazine metosulfate).

D.2.2.4 Preparations

D.2.2.4.1 General

All solutions, glassware, etc., shall be sterile and all procedures should be carried out under aseptic conditions and in the sterile environment of a laminar flow cabinet (biological hazard standard).

D.2.2.4.2 Media

MEM (buffered with sodium bicarbonate) supplemented with (final concentrations in MEM are quoted):

(A) For freezing

— 20 % FCS

— 7 % to 10 % DMSO

(B) For routine culture

— 10 % FCS

— 4 mM glutamine or glutamax

— 100 IU/ml penicillin

— 100 µg/ml streptomycin

Complete media should be kept at 4 °C and stored for no longer than two weeks.

© ISO 2009 – All rights reserved

D.2.2.4.3 XTT/PMS solution

XTT is soluted fresh in 56 °C to 60 °C MEM, without phenol red, at a concentration of 1 mg/ml with the aid of a shaker. Solution is sterilized by sterile filtration using syringe filters (pore size $\leqslant$ 0,22 µm). PMS (phenazine metosulfate) is made as a solution of 5 mM in PBS buffer and sterile filtered through a 0,22 µm sterile filter.

PMS solution is added to the XTT solution shortly before usage in a concentration of 25 µM (5 µml of a 5 mM PMS/ml XTT solution). The XTT/PMS solution is then immediately added to the test wells.

D.2.2.4.4 Preparation of sample extract

Samples are extracted in accordance with ISO 10993-12, using MEM without phenol red and with FBS.

D.2.3 Methods

D.2.3.1 General

For routine cell culture methods, see Annex C of Reference [1].

D.2.3.2 Quality check of assay (I); positive control (PC) and negative control (NC)

Positive and negative controls shall be included in every cytotoxicity test. Positive and negative reference materials are recommended, e.g. ZDEC and ZDBC (see footnote 1 on page 2).

D.2.3.3 Quality check of assay (II); blank

The absolute value of optical density (OD_{450}) obtained in the untreated blank indicates whether the 1×10^4 cells seeded per well have grown exponentially with normal doubling time during the two days of the assay.

A test meets the acceptance criteria if the mean OD_{450} of blanks is $\geqslant$ 0,2.

To check for systematic cell seeding errors, blanks are placed both at the left side (row 2) and the right side (row 11) of the 96-well plate (row 1 and row 12 shall not be used; for plate layout, see Annex E in Reference [1]).

A test meets acceptance criteria if the left and the right mean of the blanks do not differ by more than 15 % from the mean of all blanks.

Checks for cell seeding errors may also be performed by examining each plate under a phase contrast microscope to ensure that cell quantity is consistent. Microscopic evaluation obviates the need for two rows of blanks.

D.2.3.4 Test procedure

IMPORTANT — After thawing from stock, passage two to three times before using the cells in the test.

Table D.1 shows the work flow of the test procedure.

1st day after growing up the cells from frozen stock

— Cell cultures are removed from culture flasks by enzymatic digestion (trypsin/EDTA) and the cell suspension is centrifuged (200g, 3 min). The cells are then resuspended in culture medium and the cell suspension is adjusted at a density of 1×10^5 cells/ml. Using a multichannel pipette, dispense 100 µl culture medium only (blank) into the peripheral wells of a 96-well tissue culture microtitre plate (= blanks, see Appendix E in Reference [1]). In the remaining wells, dispense 100 µl of a cell suspension of 1×10^5 cells/ml (= 1×10^4 cells/well).

© ISO 2009 – All rights reserved

— Incubate cells for 24 h (5 % CO_2, 37 °C, > 90 % humidity) so that cells form a half-confluent monolayer. This incubation period ensures cell recovery, and adherence and progression to exponential growth phase.

— Examine each plate under a phase contrast microscope to ensure that cell growth is relatively even across the microtitre plate. This check is performed to identify experimental errors.

2nd day

— After 24 h incubation, aspirate culture medium from the cells.

— Per well, add 100 µl of treatment medium containing either the appropriate concentration of sample extract, or the negative control, or the PC, or nothing but blank. At least four different concentrations of the test item extract or the positive control extract should be tested. The highest concentration used should be 100 % extract and the other concentrations will be adequately spaced within a single logarithmic range. For the negative control, only the 100 % extract should be tested. Culture medium should be used as blank.

— Incubate cells for 24 h (5 % CO_2, 37 °C, > 90 % humidity).

3rd day

After 24 h treatment, examine each plate under a phase contrast microscope to identify systematic cell seeding errors and growth characteristics of control and treated cells. Record changes in morphology of the cells due to cytotoxic effects of the test sample extract, but do not use these records for any quantitative measure of cytotoxicity. Undesirable growth characteristics of control cells can indicate experimental error and can be cause for rejection of the assay.

After examination of the plates, 50 µl of the XTT/PMS solution is added to each test well and the plates are further incubated for 3 h to 5 h in the incubator at 37 °C. Plates should be kept in a dark environment. Then the plates are swayed carefully and an aliquot of 100 µl is transferred from each well into the corresponding well of a new plate and this plate is subsequently transferred to a microplate reader equipped with a 450 nm filter to read the absorbance (reference wavelength 630 nm).

Table D.1 — XTT cytotoxicity test work flow

Time<br>h	Procedure
00:00	Seed 96-well plates: 1×10^4 cells/100 µl MEM culture medium/well<br>Incubate (37 °C/5 % CO_2/22 h to 26 h)<br>↓
24:00	Remove culture medium<br>↓
24:00	Treat with ⩾ 4 concentrations of test sample extract in treatment medium (100 µl)<br>(untreated blank = treatment medium)<br>Incubate (37 °C/5 % CO_2/24 h)<br>↓
48:00	Microscopic evaluation of morphological alterations<br>Add 50 µl XTT/PMS solution<br>Incubate (37 °C/5 % CO_2/ 3 h to 5 h)<br>↓
51:00	Sway plate<br>Transfer 100 µl from each well to new plate<br>↓
51:30	Detect absorption at 450 nm (reference 630 nm)

© ISO 2009 – All rights reserved

D.2.4 Data recording

The data generated will be recorded in the raw data file. The results shall be presented in tabular form, including experimental groups with the test item, negative control, blank and positive controls.

D.2.5 Data analysis

A decrease in number of living cells results in a decrease in the overall activity of mitochondrial dehydrogenases in the sample. This decrease directly correlates to the amount of orange formazan formed, as monitored by the optical density at 450 nm. To calculate the reduction of viability compared to the blank, Equation (D.1) is used:

$$\text{Viab. \%} = \frac{100 \times OD_{450\text{e}}}{OD_{450\text{b}}} \tag{D.1}$$

where

$OD_{450\text{e}}$ is the mean value of the measured optical density of the 100 % extracts of the test sample;

$OD_{450\text{b}}$ is the mean value of the measured optical density of the blanks.

The lower the Viab. % value, the higher the cytotoxic potential of the test item is.

If viability is reduced to $<$ 70 % of the blank, it has a cytotoxic potential. The 50 % extract of the test sample should have at least the same or a higher viability than the 100 % extract; otherwise the test should be repeated.

© ISO 2009 – All rights reserved

Bibliography

[1] Guidance Document on Using *In Vitro* Data to Estimate *In Vivo* Starting Doses for Acute Toxicity, 2001. NIH Publication No. 01-4500 available under (http://iccvam.niehs.nih.gov/docs/acutetox_docs/guidance0801/iv_guide.pdf).

[2] Guidelines for preclinical biological evaluation of medical materials and devices. MHLW (Ministry of Health, Labour and Welfare, Japan) memorandum, JIMURENRAKU Iryokiki-Shinsa, **36,** 2003

[3] BORENFREUND, E. and PUERNER, J.A. Toxicity determination *in vitro* by morphological alterations and neutral red absorption, *Toxicological Letters,* **24**, pp. 119-124, 1985

[4] United States Pharmacopeia

[5] COECKE, S., BALLS, M., BOWE, G., DAVIS, J., CSTRAUNTHALER, G., HARTUNG, T., HAY, R., MERTEN, O., PRICE, A., SCHECTMAN, L., STACEY, G. and STOKES, W. Guidance on Good Cell Culture Practice, *A Report of the Second ECVAM Task Force on Good Cell Culture Practice*, ATLA, **33**, pp. 261-287, 2005

[6] ISAMA, K., MATSUOKA, A., HAISHIMA, Y. and TSUCHIYA, T. Proliferation and differentiation of normal human osteoblasts on dental Au-Ag-Pd casting alloy: Comparison with cytotoxicity using fibroblast L929 and V79 cells. *Mater. Trans.*, **43**, pp. 3155-3159, 2002

[7] TSUCHIYA, T. Studies on the standardization of cytotoxicity tests and new standard reference materials useful for evaluating the safety of biomaterials. *J. Biomaterials Applications*, **5**, pp. 139-157, 1994

[8] MOSMANN, T. Rapid colorimetric assay for cellular growth and survival: Application to proliferation and cytotoxicity assays. *J. Immun. Methods*, **65**, pp. 55-63, 1983

[9] SCUDIERO, D.A., SHOEMAKER, R.H., PAULL, K.D., MONKS, A., TIERNEY, S., NOFZIGER, T.H., CURRENS, M.J., SENIFF, D. and BOYD, M.R. Evaluation of a soluble tetrazolium/formazan assay for cell growth and drug sensitivity in culture using human and other tumor cell lines. *Cancer Res.*, **48**, pp. 4827-4833, 1988

[10] SPIELMANN, H, et al., *Interlaboratory assessment of alternatives to the Draize eye irritation test in Germany*, Toxicol. In Vitro, **5**, pp. 539-542, 1991

[11] HEXIG, B., NAKAOKA, R. and TSUCHIYA, T. Safety evaluation of surgical materials by cytotoxicity testing. *J. Artif. Organs*, **11**, pp. 204-211, 2008

 © ISO 2009 – All rights reserved

ICS 11.100.20
Price based on 34 pages

© ISO 2009 – All rights reserved
