# Supplementary material for: Endocytosis-mediated healing: recombinant human collagen type III chain-induced wound healing for scar-free recovery
Source: Regen Biomater. 2025 Jan 8;12:rbae149. doi: 10.1093/rb/rbae149 (PMC11930350; doi:10.1093/rb/rbae149)
Supplement: rbae149_Supplementary_Data [file rbae149_supplementary_data.zip › ff703_Supplementary 1-3.ISO 10993-10-2021.pdf]

INTERNATIONAL STANDARD

ISO 10993-10

Fourth edition
2021-11

Biological evaluation of medical devices —

Part 10:
Tests for skin sensitization

Évaluation biologique des dispositifs médicaux —

Partie 10: Essais de sensibilisation cutanée

Reference number
ISO 10993-10:2021(E)

© ISO 2021

COPYRIGHT PROTECTED DOCUMENT

© ISO 2021

All rights reserved. Unless otherwise specified, or required in the context of its implementation, no part of this publication may be reproduced or utilized otherwise in any form or by any means, electronic or mechanical, including photocopying, or posting on the internet or an intranet, without prior written permission. Permission can be requested from either ISO at the address below or ISO's member body in the country of the requester.

ISO copyright office
CP 401 • Ch. de Blandonnet 8
CH-1214 Vernier, Geneva
Phone: +41 22 749 01 11
Email: copyright@iso.org
Website: www.iso.org

Published in Switzerland

Contents

Page

Foreword v
Introduction vi
1 Scope 1
2 Normative references 1
3 Terms and definitions 1
4 General principles — Step-wise approach 3
5 Pretest considerations 4
5.1 General 4
5.2 Types of material 4
5.2.1 Initial considerations 4
5.2.2 Ceramics, metals and alloys 4
5.2.3 Polymers 4
5.2.4 Biologically derived materials 4
5.3 Information on chemical composition 4
5.3.1 General 4
5.3.2 Existing data sources 5
6 Skin sensitization tests 5
6.1 Choice of test methods 5
6.2 Murine local lymph node assay 6
6.2.1 Principle 6
6.2.2 Test sample preparation 6
6.2.3 Animals and husbandry 6
6.2.4 Test procedure 7
6.2.5 Treatment groups 8
6.2.6 Determination of cellular proliferation and tissue preparation 8
6.2.7 Results and interpretation 9
6.2.8 Test report 9
6.3 Guinea pig assays for the detection of skin sensitization 9
6.3.1 Principle 9
6.3.2 Choice of test sample concentrations 10
6.3.3 Induction 10
6.3.4 Challenge 10
6.4 Important factors affecting the outcome of the test 10
6.5 Guinea pig maximization test 11
6.5.1 Principle 11
6.5.2 Test sample preparation 11
6.5.3 Animals and husbandry 11
6.5.4 Test procedure 12
6.5.5 Observation of animals 14
6.5.6 Evaluation of results 14
6.5.7 Test report 15
6.6 Closed-patch test (Buehler test) 15
6.6.1 Principle 15
6.6.2 Test sample preparation 15
6.6.3 Animals and husbandry 15
6.6.4 Test procedure 16
6.6.5 Observation of animals 17
6.6.6 Evaluation of results 17
6.6.7 Test report 17
7 Key factors in interpretation of test results 18
Annex A (normative) **Preparation of materials for skin sensitization testing** 19

© ISO 2021 – All rights reserved

Annex B (informative) **Method for the preparation of extracts from polymeric test materials** 21
Annex C (informative) **Non-animal methods for skin sensitization** 24
Annex D (informative) **Background information on sensitization tests for skin sensitization** 37
Bibliography 40

 © ISO 2021 – All rights reserved

Foreword

ISO (the International Organization for Standardization) is a worldwide federation of national standards bodies (ISO member bodies). The work of preparing International Standards is normally carried out through ISO technical committees. Each member body interested in a subject for which a technical committee has been established has the right to be represented on that committee. International organizations, governmental and non-governmental, in liaison with ISO, also take part in the work. ISO collaborates closely with the International Electrotechnical Commission (IEC) on all matters of electrotechnical standardization.

The procedures used to develop this document and those intended for its further maintenance are described in the ISO/IEC Directives, Part 1. In particular the different approval criteria needed for the different types of ISO documents should be noted. This document was drafted in accordance with the editorial rules of the ISO/IEC Directives, Part 2 (see www.iso.org/directives).

Attention is drawn to the possibility that some of the elements of this document may be the subject of patent rights. ISO shall not be held responsible for identifying any or all such patent rights. Details of any patent rights identified during the development of the document will be in the Introduction and/or on the ISO list of patent declarations received (see www.iso.org/patents).

Any trade name used in this document is information given for the convenience of users and does not constitute an endorsement.

For an explanation on the voluntary nature of standards, the meaning of ISO specific terms and expressions related to conformity assessment, as well as information about ISO's adherence to the World Trade Organization (WTO) principles in the Technical Barriers to Trade (TBT) see the following URL: www.iso.org/iso/foreword.html.

This document was prepared by Technical Committee ISO/TC 194 *Biological and clinical evaluation of medical devices*, in collaboration with the European Committee for Standardization (CEN) Technical Committee CEN/TC 206, *Biological and clinical evaluation of medical devices*, in accordance with the Agreement on technical cooperation between ISO and CEN (Vienna Agreement).

This fourth edition cancels and replaces the third edition (ISO 10993-10:2010), which has been technically revised.

The main changes compared to the previous edition are as follows:

— this document now contains a description of skin sensitization testing only;

— Annex C on non-animal methods for skin sensitization (formerly Annex D) has been updated;

— the testing for irritation is now described in ISO 10993-23.

A list of all parts in the ISO 10993 series can be found on the ISO website.

Any feedback or questions on this document should be directed to the user's national standards body. A complete listing of these bodies can be found at www.iso.org/members.html.

© ISO 2021 – All rights reserved

Introduction

This document assesses possible contact hazards from chemicals released from medical devices, which may produce skin sensitization.

Some materials that are included in medical devices have been tested, and their skin sensitization potential has been documented. Especially for dental materials, sensitizing properties were reported —see Reference [51]. Other materials and their chemical components have not been tested and may induce adverse effects when in contact with human tissue. The manufacturer is thus obliged to evaluate each device for potential adverse effects prior to marketing.

Traditionally, small animal tests are performed prior to testing on humans to help predict human response (background information is provided in Annex D). Since 2015, several in chemico and in vitro assays have been validated and Organization for Economic Co-operation and Development (OECD) test guidelines released to assess the skin sentization potential of chemicals.[75][79][104] An overview of available alternative skin sensitization tests for neat chemicals is given in Annex C. These test methods, each developed to address a specific key event, can possibly not be sufficient alone to conclude on the presence or absence of skin sensitization potential of chemicals and should be considered in the context of integrated approaches such as integrated approaches to testing and assessment (IATA), combining them with other complementary information. Note that the in vitro and in chemico tests for skin sensitization in Annex C have thus far been validated only for neat chemicals and not for medical devices. To confirm that they are applicable for evaluation of the skin sensitization potential of medical devices, their assays need to be assessed and validated.

Where appropriate, the preliminary use of in vitro methods is encouraged for screening purposes prior to animal testing. To reduce the number of animals used, this document presents a step-wise approach, with review and analysis of test results at each stage. It is intended that, for regulatory submission, skin sensitization studies be conducted using GLP or ISO/IEC 17025 as applicable to the respective country and comply with regulations related to animal welfare. Statistical analyses of data are recommended and used whenever appropriate. This document includes important tools for the development of safe products and is intended for use by professionals, appropriately qualified by training and experience, who can interpret its requirements and judge the outcomes of the evaluation for each medical device, taking into consideration all the factors relevant to the device, its intended use and the current knowledge of the medical device provided by review of the scientific literature and previous clinical experience.

This document is based on numerous standards and guidelines, including OECD Guidelines, US Pharmacopoeia and the European Pharmacopoeia. It is intended to be the basic document for the selection and conduct of tests enabling the evaluation of dermal sensitization responses relevant to the safety of medical materials and devices.

© ISO 2021 – All rights reserved

Biological evaluation of medical devices —

Part 10:
Tests for skin sensitization

1 Scope

This document specifies the procedure for the assessment of medical devices and their constituent materials with regard to their potential to induce skin sensitization.

This document includes:

— details of in vivo skin sensitization test procedures;

— key factors for the interpretation of the results.

NOTE Instructions for the preparation of materials specifically in relation to the above tests are given in Annex A.

2 Normative references

The following documents are referred to in the text in such a way that some or all of their content constitutes requirements of this document. For dated references, only the edition cited applies. For undated references, the latest edition of the referenced document (including any amendments) applies.

ISO 10993-1, *Biological evaluation of medical devices — Part 1: Evaluation and testing within a risk management process*

ISO 10993-2, *Biological evaluation of medical devices — Part 2: Animal welfare requirements*

ISO 10993-12, *Biological evaluation of medical devices — Part 12: Sample preparation and reference materials*

ISO 10993-18, *Biological evaluation of medical devices — Part 18: Chemical characterization of medical device materials within a risk management process*

3 Terms and definitions

For the purposes of this document, the terms and definitions given in ISO 10993-1 and the following apply.

ISO and IEC maintain terminology databases for use in standardization at the following addresses:

— ISO Online browsing platform: available at https://www.iso.org/obp

— IEC Electropedia: available at https://www.electropedia.org/

3.1
allergen
sensitizer
substance or material that is capable of inducing a specific hypersensitivity reaction upon repeated contact with that substance or material

© ISO 2021 – All rights reserved

3.2
allergic contact dermatitis
clinical diagnosis based on an observed immunologically-mediated cutaneous reaction to a substance

3.3
blank
extraction *vehicle* (3.17) not containing the *test material* (3.15), retained in a vessel identical to that which holds the test material and subjected to identical conditions to which the test material is subjected during its extraction

Note 1 to entry: The purpose of the blank control is to evaluate possible confounding effects due to the extraction vessel, vehicle and extraction process.

3.4
challenge
process following the *induction* (3.8) phase, in which the immunological effects of subsequent exposures in an individual to the inducing material are examined

3.5
elicitation
immunological reaction to exposure to a sensitizer in a previously sensitized individual

3.6
erythema
reddening of the skin or mucous membrane

3.7
extract
liquid that results from extraction of the *test sample* (3.16) or control

[SOURCE: ISO 10993-12:2021, 3.6]

3.8
induction
process that leads to the *de novo* generation of an enhanced state of immunological activity in an individual, after initial exposure to a specific material

3.9
irritant
agent that produces *irritation* (3.10)

3.10
irritation
localized non-specific inflammatory response to single, repeated or continuous application of a substance/material

Note 1 to entry: Skin irritation is a reversible reaction and is mainly characterized by symptoms like local *erythema* (3.6) (redness), swelling, itching, peeling, cracking and scaling of the skin.

3.11
negative control
well-characterized material or substance that, when evaluated by a specific test method, demonstrates the suitability of the procedure to yield a reproducible, appropriately negative, non-reactive or minimal response in the test system

Note 1 to entry: In practice, negative controls include *blanks* (3.3), *vehicles* (3.17)/solvents and reference materials.

[SOURCE: ISO 10993-12:2021, 3.10, modified — Note 1 to entry has been replaced.]

© ISO 2021 – All rights reserved

3.12
oedema
swelling due to abnormal infiltration of fluid into the tissues

3.13
positive control
well-characterized material or substance that, when evaluated by a specific test method, demonstrates the suitability of the test system to yield a reproducible, appropriately positive or reactive response in the test system

3.14
skin sensitization
T-cell mediated delayed-type hypersensitivity reaction induced by low molecular weight reactive chemicals (allergens) comprising two phases, induction and elicitation

Note 1 to entry: In humans, the responses can be characterized by pruritis, *erythema* (3.6), *oedema* (3.12), papules, vesicles, bullae or a combination of these. In other species, the reactions can differ and only erythema and oedema can be seen.

3.15
test material
material, device, device portion or component thereof that is sampled for biological or chemical testing

3.16
test sample
material, device, device portion, component, *extract* (3.7) or portion thereof that is subjected to biological or chemical testing or evaluation

3.17
vehicle
liquid used to moisten, dilute, suspend, *extract* (3.7) or dissolve the test substance/material

4 General principles — Step-wise approach

The available methods for testing sensitization were developed specifically to detect skin sensitization potential. Other types of adverse effects are generally not predicted by these tests.

This document requires a step-wise approach, considering that any stage can result in the conclusion that further testing for skin sensitization is not necessary:

a) literature and supplier information review, including chemical and physical properties, and information on the skin sensitization potential of any medical device constituent as well as structurally-related chemicals and materials; refer to ISO 10993-1 for details; conduct risk assessment based on existing information to determine whether skin sensitization risk is acceptable or whether further testing is necessary;

b) additional characterization and risk assessment, if needed, of the device material, involving chemical characterization and analysis of the test sample according to the general principles described in ISO 10993-18;

c) in vitro tests shall be considered in preference to in vivo tests in accordance with ISO 10993-2, and the replacement of the latter as new in vitro tests are scientifically validated and become reasonably and practicably available;

   NOTE There are currently a number of internationally validated and accepted in vitro tests to detect the skin sensitization potential of chemicals; however, these in vitro tests are not yet validated for medical devices. Work is ongoing for some of these tests to qualify them for use with medical devices.

d) in vivo animal tests are only appropriate when test materials cannot be characterized and risk assessments cannot be undertaken using information obtained by the means set out in a), b) and c).

© ISO 2021 – All rights reserved

5 Pretest considerations

5.1 General

It is important to emphasize that pretest considerations can result in the conclusion that testing for skin sensitization is not necessary.

The requirements given in ISO 10993-1:2018, Clause 5, and the following apply.

In vivo, non-sterile samples shall be investigated by topical investigation only, as the possibility of microbial contamination of the test sample can confound the final assay interpretation. In cases where the sterility of a test sample cannot be guaranteed, but the sample is still considered to be free from microbial contamination, intradermal administration may be justified.

5.2 Types of material

5.2.1 Initial considerations

It shall be taken into consideration that during manufacture and assembly of medical devices, additional chemical components may be used as processing aids, e.g. lubricants or mould-release agents. In addition to the chemical components of the starting material and manufacturing process aids, adhesive/solvent residues from assembly and also sterilant residues or reaction products resulting from the sterilization process may be present in a finished product. Whether these components pose a risk depends on the leaching or degradation characteristics of the finished products. Those chemical components which have skin sensitization potential shall be identified.

5.2.2 Ceramics, metals and alloys

These materials are normally less complex than polymers and biologically derived materials in terms of the number of chemical constituents.

5.2.3 Polymers

The chemical composition of these materials is typically more complex than those in 5.2.2. A number of reaction products/impurities/additives/residual catalyst can be present and the degree or extent of polymerization can vary.

5.2.4 Biologically derived materials

These materials are inherently complex in their composition. They often also contain process residues, for example, cross-linkers and anti-microbial agents. Biological materials can be inconsistent from sample to sample.

The methods in this document have not been designed for testing of biologically derived materials and can therefore be less adequate. For example, the tests in this document do not consider cross-species sensitization.

5.3 Information on chemical composition

5.3.1 General

Full qualitative data on the chemical constituents of the material shall be established. Quantitative data on the chemical composition shall also be obtained. If quantitative data are not obtained, the rationale shall be documented and justified.

 © ISO 2021 – All rights reserved

5.3.2 Existing data sources

Qualitative and quantitative information on the composition shall be obtained where possible from the supplier of the starting material.

For polymers, this often requires access to proprietary information; provision should be made for the transfer and use of such confidential information.

Qualitative information about any additional processing additives (e.g. mould-release agents) shall also be obtained from appropriate members of the manufacturing chain, including converters and component manufacturers.

If information on composition is incomplete, a literature study to establish the likely nature of the starting material and any additives is recommended, so as to assist in the selection of the most appropriate methods of analysis for the material concerned.

The chemical composition of finalized products shall be determined in accordance with ISO 10993-18.

NOTE The composition of ceramics, metals and alloys can be specified in accordance with ISO or ASTM international standards and/or can be specified by the user. However, in order to obtain full qualitative and quantitative details on composition, it can be necessary to request these from the supplier or manufacturer of the starting material and also from component manufacturers to ensure that processing aids are also identified. Material master files held by regulatory authorities are another source of data, where they are accessible.

6 Skin sensitization tests

6.1 Choice of test methods

In vitro and in chemico alternative approaches have been developed for neat chemicals using a combination of different assays to identify skin sensitizers. Several of these methods have been included in the OECD test guidelines (TG 442C[75], TG 442D[79] and TG 442E[104]) or in the OECD test guideline program[121] (see Annex C).

Together, the assays described in these test guidelines cover three key events of the now identified adverse outcome pathway (AOP) for skin sensitization, including the molecular initiating event (protein binding), induction of inflammation, and activation of dendritic cells. These test methods developed to address a specific key event can possibly not alone be sufficient to conclude on the presence or absence of skin sensitization potential of chemicals and should be considered in the context of integrated approaches such as IATA, combining them with other complementary information.

In accordance with ISO 10993-2, such integrated approaches shall be taken into consideration for assessing skin sensitization potential of neat chemicals. Whether these approaches are also applicable for medical devices or medical device extracts is not yet known. An overview of available alternative skin sensitization tests for neat chemicals is presented in Annex C.

There are currently three animal assays available for the determination of the skin sensitizing potential of chemicals. These include two guinea pig assays and one murine assay. The two guinea pig assays are the guinea pig maximization test (GPMT) and the closed-patch test (Buehler test). Of these two assays, the maximization test is the most sensitive method. See Reference [9]. The closed-patch test is suitable for topical products.

The murine local lymph node assay (LLNA) was internationally accepted as an OECD test guideline in 2010[33] for testing single chemicals as a stand-alone alternative to the guinea pig assays, and is now the preferred in vivo assay for chemicals. See References [19] and [32]. In some instances, guinea pig assays can be necessary for the evaluation of the sensitizing potential of certain test samples. Such can be true for certain metals (see Reference [44]) that can give false negative findings in the LLNA or skin

© ISO 2021 – All rights reserved

irritants that can give false positive findings, as well as high molecular weight substances, which do not penetrate the skin or substances that are not soluble in the recommended vehicles.

NOTE All three animal assays were developed for the detection of skin sensitizing potential of chemicals, i.e. contact dermatitis, delayed type (type IV) hypersensitivity.

In view of the provisions laid down in ISO 10993-2 on animal welfare requirements, when an in vivo assay is performed, the LLNA shall be taken into consideration. In addition to animal welfare considerations, the LLNA has the advantage of providing objective quantitative data.

6.2 Murine local lymph node assay

6.2.1 Principle

Following topical treatment of a test sample on the dorsum of the ears, the extent of lymphocyte proliferation is measured in the lymph nodes that drain the sites of application (ears). A response in cellular proliferation of threefold or more compared with the activity of the controls is the threshold for designating a test material as a sensitizer.

The LLNA shall be performed using a dose response approach when substances are used. For final products/medical devices, it can be sufficient to test only the undiluted extract.

NOTE References [15] to [44] contain representative LLNA publications. Laboratories conducting this assay are encouraged to review these and other relevant publications available.

6.2.2 Test sample preparation

The test sample shall be a liquid, suspension, gel or paste such that it can be applied to the ears of the mice. Where possible, a series of doses (dilutions) shall be investigated. Otherwise, the highest concentration prepared as a chemical solution or suspension or as an extract should be used. When a strong response in the LLNA is detected with an extract, a follow-up study evaluating multiple doses may be necessary to evaluate the possible skin sensitization potency of the extract. Systemic toxicity and excessive local skin irritation can invalidate the test results; these reactions should therefore be avoided. In certain circumstances, pre-testing can be necessary.

A commonly used vehicle for substances/chemicals is an acetone olive oil (AOO) 4:1 mixture. Liquid samples that are hydrophilic and/or do not adequately adhere to the skin of the ear should be modified to adhere to the test site. This can be obtained by adding a thickening agent like carboxy methyl cellulose or hydroxyethyl cellulose (with a density of 0,5 %) or by a surfactant such as Pluronic® L92[1)] with a volume fraction of 1 %. For water soluble chemicals, dimethyl sulfoxide (DMSO) or dimethyl formamide (DMF) are preferred above the surfactant Pluronic® L92. See Reference [34]. Alternatively, other extract vehicles can be used, as mentioned. See Reference [33]. The effect of additions to the extract media and/or changes in vehicle composition shall be validated and documented. This can be done by experiments using weak to moderate skin sensitizers as commonly used as positive controls. In addition, spiking of the test sample with a positive control can be performed in order to demonstrate that the LLNA is still able to detect the presence of potential skin sensitizers in the prepared extract. Other fundamental aspects of test article extraction are specified in ISO 10993-12.

A separate extract shall be prepared for each daily application.

NOTE For polymeric materials, an optional extraction method is given in Annex B.

6.2.3 Animals and husbandry

Healthy female non-pregnant mice of the CBA/Ca, CBA/J or BALB/c strain shall be used, unless another strain is validated. See References [33], [41] and [42]. Several mouse strains have been reported as

1) Pluronic® L92 is an example of a suitable product available commercially. This information is given for the convenience of users of this document and does not constitute an endorsement by ISO of this product.

 © ISO 2021 – All rights reserved

acceptable (DBA/2, B6C3F1). See Reference [35]. The mice shall be 7 weeks to 12 weeks of age; the mice in each study shall be matched in age (within a one-week age range).

Husbandry and selection of animals shall be in accordance with ISO 10993-2. The mice, routinely acclimatized to the laboratory, shall be individually identified. For certain test samples, individual housing can be necessary. This shall be justified and documented.

Animals shall be uniquely identified by methods not to include ear punches or ear tags.

When group housing is performed, cross contamination and unwanted oral intake should be taken into consideration.

6.2.4 Test procedure

For chemicals, the LLNA is generally performed in a dose-response manner. For solid medical devices, samples to be tested shall be extracts. In these cases, only a single dose is available for testing. In general, the extract can be investigated undiluted. However, when the extract contains highly toxic components, this can result in a negative response in the LLNA due to toxicity. It is therefore recommended, to perform the LLNA in a dose-response manner and to dilute the extract when investigating cytotoxic extracts (see ISO 10993-5). In addition, when a strong response is detected in the LLNA, a dose response follow up can be conducted to evaluate the possible sensitization potency of the extract.

To ensure reproducibility and sensitivity, a test of a positive-control substance for skin sensitization shall be included by the testing laboratory in order to validate the test system and demonstrate a positive response. Well-known weak to moderate contact allergens (e.g. mercaptobenzothiazole, hexyl cinnamic aldehyde, or benzocaine), shall be used as positive control. The examples mentioned can possibly not be suitable for each vehicle used for sample preparation (e.g. water-based vehicle); in such cases, another positive control can be selected. ASTM F2148 indicates that in such circumstances formalin and 2,4-dinitrochlorobenzene (DNCB) should be used as positive controls. This shall be justified and documented.

While inclusion of a concurrent positive control group is recommended, there may be situations in which only periodic testing (i.e. at intervals ≤ 6 months) of the positive control test substance can be adequate. This is the case for laboratories that conduct the LLNA regularly (i.e. conduct the LLNA at a frequency of no less than once per month) and have an established historical positive control database that demonstrates the laboratory's ability to obtain reproducible and accurate results with positive controls. Adequate proficiency with the LLNA can be successfully demonstrated by generating consistent positive results with the positive control in at least 10 independent tests conducted within a reasonable period of time (i.e. less than one year).

The individual body weights shall be recorded at initiation and at the end of the study. In order to detect potential toxicity of the test sample, clinical observation shall be performed and recorded during the study.

Using a positive control only once every six months can have consequences for the results obtained in the previous six months period when this positive control shows a negative outcome. Reference [33] states that periodic testing (i.e. at intervals ≤ 6 months) of the positive control substance can be considered in laboratories that conduct the LLNA regularly (i.e. conduct the LLNA at a frequency of no less than once per month), and that have a history and a documented proficiency for obtaining consistent results with positive controls. It is important to realize that the decision to only include a positive control periodically instead of concurrently can have ramifications on the adequacy and acceptability of negative study results generated without a concurrent positive control during the interval between each periodic positive control study. For example, if a false-negative result is obtained in the periodic positive control test, all negative test substance results obtained in the interval between the last acceptable periodic positive control test and the unacceptable periodic positive control test can be questioned. In order to demonstrate that the prior negative test substance results are acceptable, a laboratory can be expected to repeat all negative tests, which requires additional expenses and increased animal use.

© ISO 2021 – All rights reserved

6.2.5 Treatment groups

When the LLNA is performed, the data of a minimum of five mice per group shall be available for evaluation. Lymph node responses may be determined either by individual measurement or by measurement of pooled lymph node samples. For statistical analysis, individual measurement is preferred.

When only a single dose is available for evaluation, for example, an extract, a minimum of five mice shall be used for each group, when individual responses are measured.

Treatment groups shall be assigned to:

— blank of each type of vehicle employed (see Annex A);

— when appropriate, positive control for each vehicle employed;

— test groups for each extract vehicle employed.

When testing a single chemical or substance, the LLNA shall be performed in a dose-response manner. For other types of test and sample-like extracts, a dose-response evaluation can possibly not be feasible. When only one test group is employed, this shall be justified and documented.

NOTE When sufficient data have been collected to demonstrate consistency for the dose response of the positive control, a single dose can be included to demonstrate the sensitivity of the assay. See Reference [32].

The appropriate sample shall be applied to the dorsal side of both ears of designated mice at a dose of 25 µl/d for three consecutive days. Each day, observe the ears for signs of irritation that can interfere with interpreting results. See References [23], [27] and [29].

6.2.6 Determination of cellular proliferation and tissue preparation

The proliferating cells in the draining lymph nodes can be labelled by either a radioactive or fluorescent label. Radiolabels commonly used are ^{3}H-methyl thymidine and ^{125}I-iododeoxyuridine, while for fluorescence, fluorodeoxyuridine can be used.

At (72 ± 2) h after the last treatment, record individual mouse weights and administer intravenously the label for cell proliferation. Inject 0,25 ml of phosphate buffered saline (PBS) containing 740 KBq (20 µCi) units of radioactivity of ^{3}H-methyl thymidine into all test and control mice via the tail vein. For ^{125}I-iododexyuridine, inject 0,25 ml PBS containing 74 KBq (2 µCi), and for fluorodeoxyuridine inject 0,25 ml containing 10^{-5} mol/l into the tail vein. See Reference [33].

Other alternative procedures not requiring radiolabelling are available and should be considered [e.g. adenosine triphosphate (ATP) (OECD TG 442A [122]) determination (DA method), bromodeoxyuridide BrdU (OECD TG 442B [123]) determination (ELISA or FCM method)].

NOTE 1 For more information, see References [33], [36], [42], [43] and [49].

Euthanize the mice (5 ± 0,75) h after the administration of the labelling solution according to ISO 10993-2. Remove the draining auricular lymph node. Care shall be taken to avoid cross contamination of the tissue samples. The lymph nodes of each group may be pooled, or pairs of lymph nodes of each individual animal may be pooled. Data from each individual animal is preferred as it provides the variability between each animal in a group. Single cell preparations are prepared by gently pressing the lymph nodes through a 200 µm stainless steel wire mesh or nylon mesh over a container. Rinse the strainer with chilled PBS into the container to remove cells from the mesh filter. The container now contains the cell preparation. Cell preparations are washed twice by centrifugation and resuspended in PBS. Cells are precipitated with 5 % trichloroacetic acid (TCA) at (4 ± 2) °C for (18 ± 1) h. After a final centrifugation step, pellets are resuspended in 1 ml of TCA and transferred to scintillation vials

© ISO 2021 – All rights reserved

containing 10 ml of scintillation fluid for ^{3}H-counting, or transferred directly to a gamma counter for ^{125}I-counting. See References [21], [35] and [36].

NOTE 2 Alternatively, labelling and determination of cellular proliferation can be performed ex vivo. See References [37] and [38].

6.2.7 Results and interpretation

Measure the level of radioactivity in the lymph node cells in counts per minute per mouse (cpm/mouse). Convert counts per minute (cpm) to disintegration per minute (dpm). Calculate the mean and standard deviation of dpm for at least three counts for each animal or each group of mice. Subtract the background value from each result.

When using the individual sampling method continue to calculate the mean and standard deviation of the dpm for each group of five mice. Determine the stimulation index (SI) by dividing the mean test dpm by the blank dpm. An SI of three or more (≥3,0) shall be considered positive for designating a test sample as a sensitizer. See Reference [16].

Positive control samples shall produce an SI that is greater than or equal to 3,0.

For a valid study, the positive control shall be conducted either concurrently or within the previous six months. See Reference [33].

6.2.8 Test report

The test report shall include:

a) a description of the test material(s) or device;

b) the intended use/application of the test sample or material;

c) the International Standard used (including its year of publication);

d) a detailed description of the method employed in preparing the test sample or test material or device;

e) a description of the test animals;

f) the method of application to the ears;

g) a description of the method for determining cellular proliferation;

h) any deviations from the procedure;

i) records of the observations, including clinical and body weight observations;

j) an assessment of the results, including positive control;

k) the date of the test.

6.3 Guinea pig assays for the detection of skin sensitization

6.3.1 Principle

The two guinea pig assays currently used for the detection of sensitizing activity of chemicals and medical devices are the Buehler assay and the GPMT. Both assays consist of an induction and challenge phase, thus covering all stages of hypersensitivity.

© ISO 2021 – All rights reserved

6.3.2 Choice of test sample concentrations

Current guidelines for testing the sensitizing potential of single chemicals recommend using only one concentration for the test.

NOTE For polymeric materials, an optional extraction method is given in Annex B.

6.3.3 Induction

The sensitization rate is highly dependent on the induction dose, which in guinea pig assays shall be mildly to moderately irritating, where possible. If the irritation threshold is not reached, then the highest possible concentration shall be used. However, it shall not interfere with the health of the animals. The induction dose in the guinea pig assays is normally selected based on preliminary tests as described for the individual guinea pig tests (see 6.5.4.2). Undiluted extracts with the usual solvents need not be subjected to a preliminary test.

6.3.4 Challenge

The challenge concentration in the guinea pig assays is also based on preliminary tests on animals previously not exposed to the test material. The highest non-irritant dose, as determined in the pre-test evaluations, shall be used. The use of more than one concentration is advised for the challenge procedure, in order to facilitate the evaluation of the results.

6.4 Important factors affecting the outcome of the test

The biochemical and physical characteristics of the test sample can influence the choice of test, since the maximization test requires intradermal injections. If the test sample cannot be injected intradermally, an alternative method shall be used (i.e. topical application). The extract solutions shall be prepared under aseptic conditions. Non-sterile samples shall be investigated by topical investigation only, as the possibility of microbial contamination of the test sample can confound the final assay interpretation. In cases where the sterility of a test sample cannot be guaranteed, but the sample is still considered to be non-contaminated, intradermal administration may be justified.

The bioavailability of the test material is influenced by the choice of vehicle. Although there is no vehicle that is optimal for all materials, a vehicle that optimizes exposure by solubilization and penetration should be selected. The concentration of test material should be the highest possible without affecting the interpretation of results. Most investigators prefer the test sample as a solution because dispersions are prone to form a sediment, making exact dosing difficult. Examples of vehicles for intradermal injection include saline, propylene glycol and vegetable oils.

Variation among results from different laboratories can have several sources. The following factors in the test procedure are important:

— ambient test conditions;

— test site on the animal;

— method of hair removal (clipping/shaving or chemical depilation);

— type of patch design;

— quantity of test material;

— quality of occlusion;

— exposure time and reading of the animals.

Animal responsiveness also varies according to genetic factors and husbandry.

Comparison of the number of test animals having a positive response at challenge with the appropriate controls is essential for indication of a positive test result, though the severity of reaction will aid in the

© ISO 2021 – All rights reserved

interpretation. Borderline reactions at challenge are best clarified by rechallenge. Histopathology has not been shown to be of help in the evaluation of test results.

To ensure reproducibility and sensitivity, a test of a positive-control substance for skin sensitization shall be included by the testing laboratory in order to validate the test system and demonstrate a positive response. Positive controls should preferably be weak to moderate contact allergens (e.g. mercaptobenzothiazole, hexyl cinnamic aldehyde and benzocaine). However, when consistency has been demonstrated over a six-month or more extended period, a positive control does not need to be included in every assay; but may be run at regular intervals which shall not exceed six months. Ten animals are normally used as positive controls in Guinea pig assays. Fewer uinea pigs may be used when an assay with a positive control substance is performed more frequently than once every six months. At least five test animals with a positive substance and five control animals should be used. See Reference [1].

NOTE In order to get a positive response, dilutions of moderate to strong skin sensitizers (e.g. formaldehyde and DNCB) can be used. However, this does not guarantee that the assay can also identify responses of weak sensitizers in extracts of medical devices.

6.5 Guinea pig maximization test

6.5.1 Principle

An assessment is made of the potential of the material under test to produce skin sensitization in the guinea pig using the technique applied for single chemicals in the guinea pig maximization test.

6.5.2 Test sample preparation

The test sample shall be prepared as specified in Annex A. The concentration of the test sample shall be the highest possible without affecting interpretation of the results (see 6.5.4.2).

NOTE For polymeric materials, an optional extraction method is given in Annex B.

6.5.3 Animals and husbandry

Healthy young adult albino guinea pigs of either sex from a single outbred strain, weighing 300 g to 500 g at the start of the test, shall be used. If female animals are used, they shall be nulliparous and not pregnant.

The animals shall be acclimatized and cared for as specified in ISO 10993-2. Preliminary tests, when necessary, should be carried out on one set of animals to determine the optimal test concentrations (see 6.5.4.2).

If the test material is powder or liquid, a minimum of 10 animals shall be treated with the test sample and a minimum of five animals shall act as a control group. If a preliminary test is needed, it shall be carried out on additional animals.

When testing extracts, a minimum of 10 animals shall be treated with each extract, and a minimum of five animals shall act as a solvent control group. If a preliminary test is needed, it shall be carried out on additional animals.

If testing on 10 test animals and five control animals produces completely negative results, it is unlikely that testing of a further 10 plus five animals will give positive results. However, if any equivocal responses develop, a rechallenge (see 6.5.6) shall be carried out. If equivocal responses remain, conduct a new study on a minimum of 20 test animals and 10 control animals.

© ISO 2021 – All rights reserved

6.5.4 Test procedure

6.5.4.1 Preparation

Clip and shave the fur on all treatment sites prior to all steps in the test procedure.

6.5.4.2 Preliminary tests

The preliminary tests are intended to determine the concentration of the test sample to be used in the main test given in 6.5.4.3.

Undiluted extracts using the usual solvents (e.g. saline or vegetable oil) need not be subjected to preliminary testing.

For topical application, saturate an appropriate filter paper or absorbent gauze patch (4 cm^2 to 8 cm^2) or chamber with the test sample and apply the patch to the clipped skin under an occlusive dressing secured by a wrap and/or jacket around the torso of the animal.

When wrapping an animal for securing an occlusive dressing, care should be taken to allow for normal breathing of the animal. A flexible wrapping is preferred, which should be applied by well-trained personnel.

Topically apply a range of dilutions of the test sample to the flanks of at least two animals. Remove the occlusive dressings and patches after 24 h and assess the application sites for erythema and oedema using the Magnusson and Kligman grading scale given in Table 1. It can also be appropriate to explore dilution of the article by intradermal injection when using non-usual solvents.

For the topical induction phase in the main test, select the highest concentration that causes mild to moderate erythema but does not otherwise adversely affect the animal in accordance with ISO 10993-2. It should be recognized that for extracts of medical devices, the irritating threshold can possibly not be obtained. In such cases, the highest concentration possible shall be used (e.g. the undiluted extract). For final products/medical devices, it may be sufficient to test only the undiluted extract.

For the challenge phase in the main test, select the highest concentration that produces no erythema (see Table 1).

Table 1 — Magnusson and Kligman scale

Patch test reaction	Grading scale
No visible change	0
Discrete or patchy erythema	1
Moderate and confluent erythema	2
Intense erythema and/or swelling	3

Consideration shall be given to the pre-treatment of all animals by injection with Freund's complete adjuvant (FCA).

6.5.4.3 Main test

6.5.4.3.1 Intradermal induction phase

Make a pair of 0,1 ml intradermal injections of each of the following, into each animal, at the injection sites (e.g. sites A, B and C), as shown in Figure 1, in the clipped intrascapular region.

— Site A: A 50:50 volume ratio stable emulsion of Freund's complete adjuvant mixed with the chosen solvent. Use physiological saline (BP, USP or equivalent) or extraction vehicle/solvent.

— Site B: The test sample (undiluted extract); inject the control animals with the extraction vehicle/solvent alone.

© ISO 2021 – All rights reserved

— Site C: The test sample at the concentration used at site B, emulsified in a 50:50 volume ratio stable emulsion of Freund's complete adjuvant and the solvent/extraction vehicle (the solution used at site A); inject the control animals with an emulsion of the blank liquid with adjuvant.

6.5.4.3.2 Topical induction phase

At (7 ± 1) d after the intradermal induction phase, administer the test sample by topical application to the intrascapular region of each animal, using a patch of area approximately 8 cm^2 (filter paper or absorbent gauze), so as to cover the intradermal injection sites (see Figure 1). Use the concentration selected in the preliminary test by topical application (if done; see 6.5.4.2). If the maximum concentration that can be achieved in 6.5.4.3.1 does not produce irritation, pretreat the area with 10 % sodium dodecyl sulfate (SDS) massaged into the skin (24 ± 2) h before the patch is applied. Any remaining SDS should be removed prior to application of the patches for the topical induction phase, as the remaining SDS can affect absorption of the extract. Secure the patches with an occlusive dressing. Remove the dressings and patches after (48 ± 2) h.

Freshly prepared extracts are preferred. If an extract is stored longer than (24 ± 2) h, then the stability of the extract under the conditions of storage should be verified.

Treat the control animals similarly, using the blank liquid alone.

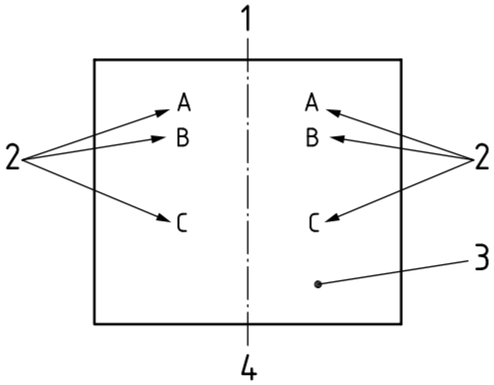

Key

1 cranial end

2 0,1 ml intradermal injections (see 6.5.4.3.1)

3 clipped intrascapular region

4 caudal end

A, B, C injection sites

Figure 1 — Location of intradermal injection sites

6.5.4.3.3 Challenge phase

For the challenge phase testing the procedure described below shall be followed:

a) All test and control animals shall be challenged at (14 ± 1) d after completion of the topical induction phase

b) For undiluted test extracts using standard solvents (e.g. saline or vegetable oil):

   1) both test and control animals shall be dosed with both test and control extracts; or

© ISO 2021 – All rights reserved

2) control animals shall be dosed with undiluted vehicle and test animals with undiluted test extract.

For the challenge phase, the use of highest non-irritating concentration of the test sample is recommended. For testing with medical device extracts, preliminary testing to determine the highest non-irritating concentration of the extracts is not generally conducted. It is stated in 6.5.4.2 that undiluted extracts using the usual solvents need not be subjected to preliminary testing.

The use of option b),1) allows one to determine if any skin reaction observed in the test animals is due to irritation rather than sensitization. Per option b),1), both test and control animals are dosed with both test and control extracts. If any skin reactions to the test extracts are observed in the control animals, the reaction(s) can be due to irritation rather than sensitization as the control animals have not been previously exposed to the test extracts.

Per option b),2), the control animals are not dosed with the test extract and test animals are not dosed with the control extract. In the event of skin reactions observed in the test animals where no preliminary testing was conducted to determine the highest non-irritating concentration of the extracts, additional testing can be needed to rule out a false positive.

c) When non-standard solvents are used, all test and control animals shall be dosed with both the test and control extracts.

d) The extracts shall be administered by topical application to shaved sites that were not treated during the induction stage, such as the upper flank of each animal, using appropriate patches or chambers soaked in the test sample at the concentration selected in 6.5.4.3.1 for site B. In case the concentration selected in 6.5.4.3.1 for site B is not the highest non-irritating concentration, the highest non-irritating concentration determined in the preliminary test (see 6.5.4.2) shall be used. The extract volume used for saturation of patches/chambers shall be specified and justified.

e) The patches/chambers shall be secured with an occlusive dressing.

f) Dressings and patches shall be removed after (24 ± 2) h.

6.5.5 Observation of animals

Observe the appearance of the challenge skin sites of the test and control animals (24 ± 2) h and (48 ± 2) h after removal of the dressings. Use of natural or full-spectrum lighting is highly recommended in order to visualize the skin reactions. Describe and grade the skin reactions for erythema and oedema according to the Magnusson and Kligman grading scale given in Table 1 for each challenge site and at each time interval. It is highly recommended that reading be done without knowledge of the treatment, in order to minimize bias in the evaluation of the results.

Clipping and shaving should be done before each step in the test procedure (see 6.5.4.1). However, reshaving may not be necessary after the challenge or re-challenge when an animal is shaved the day before.

6.5.6 Evaluation of results

Magnusson and Kligman grades of 1 or greater in the test group generally indicate sensitization, provided grades of less than 1 are seen in control animals. If grades of 1 or greater are noted in control animals, then the reactions of test animals which exceed the most severe reaction in control animals are presumed to be due to sensitization. If the response is equivocal, rechallenge is recommended to confirm the results from the first challenge. The outcome of the test is presented as the frequency of positive challenge results in test and control animals.

Occasionally, the test group has a greater number of animals showing a response than the controls, although the intensity of the reaction is not greater than that exhibited by the controls. In these instances, a rechallenge can be necessary to define the response clearly. A rechallenge shall be carried

 © ISO 2021 – All rights reserved

out 1 week to 2 weeks after the first challenge. The method used shall be as described for the first challenge, using a naïve side on the animal.

6.5.7 Test report

The test report shall include:

a) a description of the test material(s) or device;

b) the intended use/application of the test sample or material;

c) the International Standard used (including its year of publication);

d) a detailed description of the method employed in preparing the test sample or test material or device;

e) a description of the test animals;

f) the method of application to the test sites;

g) any deviations from the procedure;

h) how the sites were marked, and the readings performed;

i) records of the observations;

j) assessment of the results;

k) the date of the test.

6.6 Closed-patch test (Buehler test)

6.6.1 Principle

An assessment is made of the potential of the material under test to produce skin sensitization in guinea pigs.

6.6.2 Test sample preparation

The test sample shall be prepared as specified in Annex A. The concentration of test sample shall be the highest possible without affecting interpretation of the results (see 6.6.4.2). Where shape and size permit, topical devices (e.g. electrodes) can be used as they are.

6.6.3 Animals and husbandry

Healthy young adult albino guinea pigs of either sex from a single outbred strain, weighing 300 g to 500 g at the start of the test, shall be used. If female animals are used, they shall be nulliparous and not pregnant.

The animals shall be acclimatized and cared for as specified in ISO 10993-2. Preliminary tests should be carried out on one set of animals to determine concentrations of test sample (see 6.5.4.2).

For testing powders or liquids, a minimum of 10 animals shall be treated with the test material and a minimum of five animals shall act as a control group. If a preliminary test is needed, it shall be carried out on additional animals.

When testing extracts, a minimum of 10 animals shall be treated with each extract and a minimum of five animals shall act as a control for each solvent. If a preliminary test is needed, it shall be carried out on additional animals.

© ISO 2021 – All rights reserved

If testing in 10 test and five control animals produces completely negative results, it is unlikely that testing of a further 10 plus five animals will give positive results. However, if any equivocal responses develop, a rechallenge (see 6.5.6) shall be carried out. If equivocal responses remain, conduct a new study in a minimum of 20 tests and 10 control animals.

6.6.4 Test procedure

6.6.4.1 Preparation

Closely clip or shave the fur on all treatment sites prior to all steps in the test procedure.

6.6.4.2 Preliminary tests

The preliminary tests are intended to determine the concentrations of the test sample to be used in the main test described in 6.6.4.3.

Medical devices intended for topical use and undiluted extracts using the usual solvents need not be subjected to preliminary testing.

For all topical applications, saturate a patch (filter paper or absorbent gauze) of the appropriate dimensions with the test material or extract and apply the patch to the clipped area under an occlusive dressing for (6 ± 0,5) h. Restraint for each animal can be used to ensure occlusion of the test sites. If wrapping is used, its adequacy should be evaluated in every experiment. Assess the application sites for erythema and oedema using the Magnusson and Kligman grading given in Table 1 at (24 ± 2) h and (48 ± 2) h after patch removal.

Topically apply four concentrations of the test sample to the flanks of each of at least two animals using appropriate patches. Remove the occlusive dressings and patches after (6 ± 0,5) h. Assess the application sites for erythema and oedema using the Magnusson and Kligman grading given in Table 1 at (24 ± 2) h and (48 ± 2) h after patch removal.

Select:

a) for the induction phase in the main test, the highest concentration that causes no more than slight erythema but does not otherwise adversely affect the animals;

b) for the challenge phase in the main test, the highest concentration that produces no erythema.

6.6.4.3 Main test

6.6.4.3.1 Induction phase

Administer the test sample by topical application to the clipped left upper back region of each animal using appropriate patches soaked in the test sample at the concentration selected in 6.6.4.2 a). Remove the restrainer of any occlusive dressings and patches after (6 ± 0,5) h. Perform this procedure three days a week for three weeks. Treat the control animals similarly, using the blank liquid alone.

6.6.4.3.2 Challenge phase

At (14 ± 1) d after the last induction application, challenge all test and control animals with the test sample. Administer the test sample by a single topical application to a clipped untested area of each animal using appropriate patches soaked in the test sample at the concentration selected in 6.6.4.2 b). Remove the restrainer and occlusive dressings and patches after (6 ± 0,5) h.

 © ISO 2021 – All rights reserved

6.6.5 Observation of animals

If necessary, at (24 ± 2) h after the start of the primary challenge or rechallenge exposure, either

a) depilate all of the animals with a commercial depilatory by placing the material on the test site and surrounding areas according to the manufacturer's instructions or

b) shave all of the animals on the challenge sites and surrounding areas.

Thoroughly wash the depilated area with warm water and dry the animals with a towel before returning them to their cages. At (24 ± 2) h after removal of the challenge patches and a minimum of 2 h after removal of the hair, grade the test sites using the scale given in Table 1. Repeat the grading (48 ± 2) h after removal of the challenge patch. Use of natural or full-spectrum lighting is highly recommended in order to visualize the skin reactions. It is highly recommended that reading be done without knowledge of the treatment, in order to minimize bias in the evaluation of the results.

6.6.6 Evaluation of results

The Magnusson and Kligman grading scale given in Table 1 shall be applied.

Grades of 1 or greater in the test group generally indicate sensitization, provided grades of less than one are seen on control animals. If grades of 1 or greater are noted on control animals, then the reactions of test animals which exceed the most severe control reaction are presumed to be due to sensitization. Rechallenge is recommended to confirm the results from the first challenge. The outcome of the test is presented as the frequency of positive challenge results in test and control animals.

Occasionally, the test group has a greater number of animals showing a response than the controls, although the intensity of the reaction is not greater than that exhibited by the controls. In these instances, a rechallenge can be necessary to define the response clearly. A rechallenge shall be carried out 1 week to 2 weeks after the first challenge. The method used shall be as described for the first challenge, using an untested area on the flank of the animal.

In these situations, a new negative control group is recommended.

6.6.7 Test report

The test report shall include:

a) a description of the test material(s) or device;

b) the International Standard used (including its year of publication);

c) the intended use/application of the test material(s) or device;

d) a detailed description of the method employed in preparing the test samples and materials;

e) a description of the test animals;

f) the method of application to the test sites;

g) any deviations from the procedure;

h) a description of how the sites were marked, and the readings performed;

i) records of the observations;

j) assessment of the results, including statistical methods;

k) the date of the test.

© ISO 2021 – All rights reserved

7 Key factors in interpretation of test results

The tests included in this document are important tools for development of safe products and shall be executed in accordance with appropriate quality assurance measures (e.g. ISO/IEC 17025 or GLP) and interpreted by trained personnel. Evidence shall be provided that those planning, conducting and interpreting the tests are appropriately qualified by training and experience for the tasks undertaken.

Evidence of skin sensitization by any method does not necessarily exclude the test material or device from use because the amount of test material in the test procedure can be exaggerated compared with actual conditions of use. An adverse finding using any of the described procedures indicates the need for further analysis that can allow risk assessment of intended human exposure.

Predictive test results generated by the procedures described in this document cannot stand alone and need to be interpreted alongside other information to assess the risk of a hypersensitivity reaction or other forms of immunotoxicity. A negative test result does not exclude the possibility that a product can cause allergic skin reactions. The results should be compared with other sources of information, such as:

— industry and consumer complaint data;

— experience with devices containing similar components;

— diagnostic test results in dermatologic clinics;

— retrospective epidemiologic data.

© ISO 2021 – All rights reserved

Annex A
(normative)

Preparation of materials for skin sensitization testing

A.1 General

The conduct of the tests and interpretation of the data from skin sensitization tests shall take into account the nature, degree, frequency, duration and conditions of exposure of the medical device in humans. One of the parameters critical to these tests is the preparation of the test material.

A.2 Materials for direct-contact exposure

A.2.1 Solid test materials

Solid materials that have appropriate physical states (e.g. sheets, films) shall be tested without modification. Prepare samples 2,5 cm × 2,5 cm with a thickness that approximates normal use but is not greater than 0,5 cm. Prepare suitable negative control samples in the same way. The negative control shall physically resemble the test material closely and should be non-irritating. Absorbent gauze may be used as a substitute if a more suitable control cannot be identified.

The solid can be pulverized, care being taken to ensure no contamination occurs during this process, or moistened sufficiently with water or a suitable non-irritant solvent to ensure good contact with the tissues. In the case of ceramics where pulverization is required, remember that the physico-chemical properties of the ceramic can be altered by reducing the ceramic to a powder, with potentially marked effects on biological activity.

Powders (e.g. super-absorbents) shall be tested by direct deposition or by making a paste in an appropriate solvent. A control using the same solvent shall be evaluated in parallel with the moistened, diluted or suspended test material.

NOTE Either surface area or particle size, or both, are important factors in biological responses such as phagocytosis, which plays an important role in inflammatory and immune responses.

A.2.2 Liquid test materials

Liquids shall be tested undiluted by direct deposition or, if impractical, diluted with an appropriate solvent. A control using the same solvent shall be evaluated in parallel with the diluted test liquid.

A.3 Extracts of test materials

A solid can be tested by preparing extracts from the solid. If extracts are tested, they shall be prepared as specified in ISO 10993-12, using polar, non-polar and/or additional solvents when appropriate. A rationale shall be provided for the adequacy of an extraction method.

A blank sample, using the extracting solvent, shall be evaluated in parallel with the extract of the test material.

NOTE For polymeric materials, an optional extraction method is described in Annex B.

© ISO 2021 – All rights reserved

A.4 Solvents

If the test material has to be extracted, diluted, suspended or moistened, a suitable non-irritant and non-sensitizing solvent shall be used. ISO 10993-12 provides a list of appropriate solvents.

A.5 Sterile test materials

If the final product is supplied in a sterile condition, then the test material shall be sterilized using the same process prior to testing. Products sterilized by ethylene oxide present a technical difficulty in that ethylene oxide and its reaction products can produce a biological response in the tests described in this document.

To enable differentiation between effects produced by the test material and those produced by ethylene oxide residuals when an irritant reaction is observed, consideration shall be given to evaluations of this response to the device pre- and post-ethylene oxide sterilization.

© ISO 2021 – All rights reserved

Annex B
(informative)

Method for the preparation of extracts from polymeric test materials

B.1 General

This annex provides guidance on the preparation of extracts of a polymeric test material to be used in the GPMT. The extract preparation is originally described in Reference [3] and additional information is provided in ISO 10993-12:2021, Clause D.2 for “Points to be considered for exhaustive extraction of polymeric medical devices for biological testing”.

B.2 Preparation method

B.2.1 Preliminary extraction

A preliminary extraction procedure is performed on the test sample to determine the most suitable extraction process for use in the GPMT.

Methanol and acetone are the recommended solvents for extraction. The test sample is cut into small pieces (if possible) and placed in two separate flasks. A 10- to 20-fold volume (i.e. 10 ml to 20 ml of solvent for each gram of test sample) of each solvent is added to each flask and the flasks are shaken at room temperature for extraction. Extraction by shaking is performed three times [e.g. for (4 ± 1) h, (8 ± 1) h, or (24 ± 2) h] within a 24 h to 72 h period using the same volume of fresh solvent each time. The extract is collected from each extraction period and pooled. The solvent is removed by evaporation to obtain a residue.

The most suitable solvent for testing is determined based on the mass of residue obtained. The percent yield of the residue should be determined. The solvent yielding the highest amount of residue is chosen as the extraction solvent for the skin sensitization testing.

Determine the solubility of the residue by adding olive oil, acetone, methanol or dimethylsulfoxide (DMSO). The solution that dissolves most of the residue is used as a vehicle for testing in the GPMT.

NOTE If the test sample dissolves or degrades in acetone or methanol or if an adequate amount of residue cannot be obtained, *n*-hexane or a 1:1 mixture of cyclohexane and 2-propanol can be used as an extraction solvent.

B.2.2 Final extraction

B.2.2.1 General

There are two methods for preparing the test solution from the organic solvent extract.

Method 1 is applicable when the amount of residue obtained by solvent extraction of a test sample and the weight of a test sample are relatively high because sufficient amounts of residue have been obtained. In addition, Method 1 is especially recommended to evaluate the risk for the medical devices which are repeatedly used. See Reference [14].

Method 2 is applicable when the amount of residue obtained by solvent extraction of a test sample or the weight of a test sample is relatively low. Examples of the latter are contact lenses and intraocular lenses.

© ISO 2021 – All rights reserved

For both Methods 1 and 2, in parallel to the extraction of the test sample, the amount of solvent equal to the total volume used during the extraction of the test sample is subjected to the same concentration procedure as the test extracts. This solvent blank is used as negative control for each phase of testing.

B.2.2.2 Test sample preparation according to Method 1

For Method 1, the extraction is performed by covering the test sample with a 10- to 20-fold volume of the appropriate solvent (as determined in the preliminary extraction test) and agitating (shaking) at room temperature. The solvent is collected in another flask. The solvent is exchanged three times [e.g. after extraction for (4 ± 1) h, (8 ± 1) h or (24 ± 2) h] and repeated to agitate at room temperature within a 24 h to 72 h period depending on the leaching and stability of the substances extracted from the test material.

A residue is obtained by evaporating the collected solvent. A rotary evaporator is used at the lowest possible temperature that provides controlled evaporation under reduced pressure.

The residue is dissolved in an appropriate vehicle (olive oil/acetone/ethanol/DMSO) as determined by the solubility experiment in the preliminary extraction test, to prepare a mass fraction of mass fraction of 10 % test solution for the intradermal induction phase and a mass fraction of 20 % test solution for the intradermal induction phase and for the topical induction phase in the GPMT. For the challenge phase in the GPMT, a mass fraction of 10 % solution is prepared in the vehicle. The 10 % solution is further diluted with the vehicle to obtain 1 %, 0,1 %, 0,01 % and 0,001 % test solutions.

B.2.2.3 Test sample preparation according to Method 2

For Method 2, the extraction is performed by covering the test sample with a 10- to 20-fold volume of the appropriate solvent (as determined in the preliminary extraction test) and shaking at room temperature for (24 ± 2) h. The solvent is collected in one flask. The extraction procedure is repeated three times within a 24 h to 72 h period using the same volume of fresh solvent each time. The extracts are pooled in one flask and the solvent is evaporated.

For the intradermal induction phase, the extracts obtained are evaporated until the residual number of millilitres of the extract is equal to or slightly less than half the original number of grams of the sample used (i.e. if 10 g of test sample are extracted, then the combined solvent extract is evaporated down to around 5 ml), or evaporated completely to obtain a residue. When a residue is obtained, this is dissolved in the suitable vehicle (as determined in the preliminary extraction test) to 5 ml. This solution is considered as 200 % test solution.

In addition, 100 % test solution is prepared by diluting the 200 % test solution with the vehicle.

For the topical induction phase, the 100 % test solution is used. For both the intradermal and topical induction phase, the vehicle in the 200 % and 100 % test solutions is replaced with olive oil by combining the test solution with an equal volume of olive oil and evaporating the vehicle under a stream of nitrogen gas.

For the challenge phase, the 100 %, 50 %, 25 %, 12,5 % and 6,25 % test solutions are used. The 100 % test solution is diluted with the vehicle to obtain 50 %, 25 %, 12,5 % and 6,25 % test solutions. The vehicle in the test solutions is not replaced with olive oil for the challenge phase.

B.3 Guinea pig maximization test

B.3.1 General

The GPMT should be performed as described in 6.5 with the exception of the challenge phase which is described below. The challenge phase, using the solvent extraction method, should be performed without an occlusive dressing.

© ISO 2021 – All rights reserved

B.3.2 Challenge phase

Two weeks after the closed-patch application of the topical induction phase, all test and control animals are challenged with the test sample.

For Method 1, a 0,1 ml aliquot of the 10 % (w/w), 1 % and 0,1 % test solutions are topically applied on the right flank of each test and negative control animal. In addition, a 0,1 ml aliquot of the 0,01 % and 0,001 % test solutions and the negative control vehicle are applied topically to the left flank of each test and negative control animal.

For Method 2, a 0,1 ml aliquot of the 100 %, 50 % and 25 % test solutions are topically applied on the right flank of each test and negative control animal. In addition, a 0,1 ml aliquot of the 12,5 % and 6,25 % test solutions and the negative control vehicle are applied topically to the left flank of each test and negative control animal.

For both Method 1 and Method 2, the positive control animals are treated with a 0,1 ml aliquot of 0,1 % DNCB in ethanol on the right flank and ethanol on the left flank.

NOTE Occlusive challenge can be performed similarly.

© ISO 2021 – All rights reserved

Annex C
(informative)

Non-animal methods for skin sensitization

C.1 Introduction

C.1.1 Background on alternative methods for skin sensitization testing

The effort to reduce or replace the use of animals in toxicity testing has led to the development of many new non-animal methods. While they are accepted for testing of chemicals, interest in using these new methods for the safety assessment of drugs and medical devices has increased in recent years. For skin sensitization, in vivo test methods using animals or humans are often employed because they allow for observance of the adverse outcome (i.e. allergic contact dermatitis), in the living subject. However, as a greater understanding of the molecular and cellular mechanisms of the skin sensitization process have evolved, non-animal assays have been developed for the assessment of the skin sensitization potential of chemicals or materials[57]. These assays can be grouped in three categories:

— in chemico assays: study or procedure that is related to the intrinsic reactivity of a material involving physicochemical measurements rather than biological testing;

— in silico assays: study or procedure that is performed using a computer simulation (e.g. such as a computer model of a molecule or cell that accurately simulates its behaviour, or quantitative structure–activity relationship (QSAR) evaluation);

— in vitro assays: study or procedure that is conducted outside of a living organism and under controlled conditions (e.g. cell cultures in a dish).

To date, none of the validated assays in these categories is able to reproduce entirely the precise and intricate network of molecular, cellular, and organ level mechanisms that operate in living organisms. Consequently, each assay, when considered individually, cannot be able to completely replicate the events that trigger the adverse outcome. Rather, the prediction of the skin sensitization potency of a chemical in human is based on mechanistic approaches combining several individual assays in testing strategies[66][70].

C.1.2 OECD's adverse outcome pathway for skin sensitization

The OECD has described the sequential chain of linked events at the molecular, cellular, tissue and organ level leading to the skin sensitizing effect[5]. This series of events were structured to develop and generate a detailed and standardized picture of the pathway leading to the adverse outcome observed in the whole organism (animal or human). This series of events is called the AOP. The AOP links in a linear way existing knowledge along one or more series of causally connected key events (KE) between two points — a molecular initiating event (MIE) and the adverse outcome (AO)[56]. AOPs are the central element of a toxicological knowledgebase created to support chemical risk assessment built on mechanistic reasoning.

The AOP for skin sensitization begins after exposure to and absorption of a sensitizing agent. After the agent penetrates the stratum corneum of the skin, a series of events at the molecular, cellular and organ levels take place that lead to the adverse end point of allergic contact dermatitis or contact hypersensitivity. The four key events in the AOP for skin sensitization are:

— Key event 1: covalent binding to skin proteins: the molecular initiating event after penetration of the stratum corneum is the irreversible formation of the hapten-protein complex. In the animal, this event is associated with the production of a specific memory T-cell response.

© ISO 2021 – All rights reserved

— Key event 2: keratinocyte response: this key event involves the activation of biochemical pathways in the keratinocytes and includes inflammatory mediator responses as well as gene expression changes associated with cell signalling pathways such as the antioxidant/electrophile response element (ARE).

— Key event 3: activation of dendritic cells: the detailed biochemical events after formation of the hapten-protein complex have not been fully elucidated. However, the pathways involved are known to be inflammation-related such as the mitogen-activated protein kinase signalling pathway and the oxidative stress response pathway which occur especially in keratinocytes and dendritic cells. Effects at the cellular and tissue levels are also not completely known but involve epidermal responses which include:

  1) immune recognition of chemical allergens by keratinocytes, specialized epidermal dendritic cells (i.e. Langerhans cells) and dermal dendritic cells;

  2) responses in the form of expression of specific cell surface markers like adhesion molecules, chemokines, and cytokines such as IL-1β or IL-12p70 are typically taken as evidence of dendritic cell maturation.

— Key event 4: T-cell proliferation: at the organ level (lymph nodes and skin), the responses are:

  1) dendritic cell migration to the lymph node, where the antigen is presented by major histocompatibility complex (MHC) molecules to naïve T-lymphocytes (T-cells), and

  2) T-cell differentiation and proliferation as allergen-specific effector and memory T-cells.

The final physiological response is acquisition of sensitivity. The key organism response is dermal inflammation upon receiving the substance challenge in the elicitation phase. This response is associated with stimulation of specific memory T-cell produced in the induction phase. The overall effect on mammals is allergic contact dermatitis in humans, or its rodent equivalent contact hypersensitivity[65].

C.1.3 Integrated approaches to testing and assessment

IATA are pragmatic, science-based approaches for chemical hazard characterization that rely on an integrated analysis of existing information coupled with the generation of new information using testing strategies. To predict the skin sensitization potential of chemicals in humans, alternative non-animal methods are either used in combination or with existing relevant information to fully characterize the biological end point. The sensitization end point can be determined by utilizing an integrated approach whereby all existing and reliable information about a chemical or material are combined with data from biochemical (in chemico), computational (in silico), and/or cell-based (in vitro) methods to adequately predict the toxicity of the material or chemical. Sometimes, the integrated approach may require additional data to be generated using non-animal approaches to gain full acceptance as a predictor of a specific toxicity end point.

Examples of in vitro skin sensitization assays include KeratinoSens™, LuSens, U-SENS™, the human Cell Line Activation Test (h-CLAT), and the IL-8 Luc Assay. The direct peptide reactivity assay (DPRA) is an example of an in chemico skin sensitization assay. While examples of in silico sensitization computational methods include QSAR Toolbox and TImes MEtabolism Simulator platform used for predicting skin sensitization (TIMES SS)[56][67]. Several of these methods can be combined in an IATA to evaluate the skin sensitization potential of chemicals or materials.

To standardize the evaluation of IATAs in regulatory decision-making, guidance has been developed by OECD to provide principles and templates for reporting defined approaches (DA) and individual information sources[57][67]. A DA consists of a fixed data interpretation procedure (DIP) applied to data generated with a defined set of information sources to derive a result that can either be used on its own, or together with other information sources within an IATA, to satisfy a specific regulatory need.

To gain regulatory acceptance of IATAs for skin sensitization potential, integrated approaches include defined approaches (DA) whereby for each DA, the relevant information and specific non-animal methods to use in combination are clearly delineated and a procedure for interpreting the data from

© ISO 2021 – All rights reserved

these identified methods are provided. Thus, DAs are rule-based strategies that can obviate the need for expert judgment once a specific approach is described, validated, standardized and accepted[61].

OECD Guidance Document 256:2017, Annex I[124] (see Table C.1) outlines 12 illustrative case studies of DAs for skin sensitization. Some of them are currently under consideration by OECD for a future test guideline "Defined Approaches for Skin Sensitisation".

Table C.1 — Case studies of the OECD Guidance Document 256:2017, Annex I[124]

	Case study	Purpose
I	Adverse outcome pathway-based "2 out of 3" integrated testing strategy approach to skin hazard identification (BASF)	Hazard identification
II	Sequential testing strategy (STS) for hazard identification of skin sensitizers (RIVM)	Hazard identification
III	Non-testing pipeline approach for skin sensitization (G. Patlewicz)	Hazard identification
IV	Stacking meta-model for skin sensitization hazard identification (L'Oréal)	Hazard identification
V	Integrated decision strategy for skin sensitization hazard (ICCVAM)	Hazard identification
VI	Consensus of classification trees for skin sensitization hazard prediction (EC-JRC)	Hazard identification
VII	Sensitizer potency prediction based on Key event 1 + 2: Combination of kinetic peptide reactivity data and KeratinoSens® data (Givaudan)	Potency prediction
VIII	The artificial neural network model for predicting LLNA EC3 (Shiseido)	Potency prediction
IX	Bayesian network DIP (BN-ITS-3) for hazard and potency identification of skin sensitizers (P&G)	Potency prediction
X	STS for sensitizing potency classification based on in chemico and in vitro data (Kao Corporation)	Potency prediction
XI	Integrated testing strategy (ITS) for sensitizing potency classification based on in silico, in chemico, and in vitro data (Kao Corporation)	Potency prediction
XII	DIP for skin allergy risk assessment (SARA) (Unilever)	Potency prediction

C.2 In vitro assays for skin sensitization testing

C.2.1 General

Subclause C.2 provides summaries of in vitro skin sensitization assays that have been included in recent reviews and international evaluation studies[58][59][65][68].

C.2.2 Test methods

C.2.2.1 DPRA

The DPRA is an in chemico method that quantifies the reactivity of a test chemical through its depletion of synthetic peptides containing cysteine or lysine. Percent depletion values are calculated and a prediction model categorizes the chemical as a skin sensitizer or non-sensitizer. See Table C.2.

© ISO 2021 – All rights reserved

Table C.2 — DPRA

Key event 1: Molecular initiating event of colvalent protein binding	
OECD Guideline	OECD TG 442C[75]
Performance (yes/no answer)	80 % accuracy, 80 % sensitivity, 77 % specificity
Experimental system	The concentration of cysteine or lysine remaining in a synthetic peptide is determined following incubation at 22,5 °C to 30 °C for 24 h with the test chemical. To aid in detection, the synthetic peptides contain phenylalanine. Peptide concentrations are measured by high-performance liquid chromatography (HPLC) with gradient elution and UV detection at 220 nm.
Principle of the test	Cysteine and lysine peptide percent depletion values are determined and used in a prediction model that assigns the test chemical to one of four reactivity classes that categorize them as skin sensitizers or non-sensitizers.
Read out	Relative peptide concentrations are measured by HPLC with 220 nm detection.
Time of exposure	24 h
Applicability	Not applicable for testing metal compounds since they can react with proteins through mechanisms other than covalent binding. The test chemical needs to be completely dissolved in an appropriate solvent at a concentration of 100 mM. Testing of mixtures is possible if their constituents are known.
Application to medical devices	There is no data. DPRA works with acetonitrile, water, isopropanol and acetone. However, it can possibly not work with non-polar medical device solvents such as sesame, cottonseed, or olive oil. As the assay uses an excess of chemical versus the peptides used in the assay, it is unlikely that the assay is suited to detect skin sensitizers in medical device extracts.
See References [74] to [76].	

C.2.2.2 KeratinoSens™ 2)

The KeratinoSens™ assay makes use of an immortalised human keratinocyte adherent cell line containing a luciferase reporter gene controlled by the antioxidant response element (ARE) of the human AKR1C2 gene, which is known to be up regulated by skin sensitizers. See Table C.3.

Table C.3 — KeratinoSens™

Key event 2: Activation of epidermal keratinocytes	
OECD Guideline	OECD TG 442D[79]
Performance (yes/no answer)	77 % accuracy, 78 % sensitivity, 79 % specificity
Experimental system	KeratinoSens™ transgenic cell line: immortalised adherent cell line derived from human keratinocytes stably harbouring a luciferase reporter gene under the control of the ARE of the human AKR1C2 gene
Principle of the test	The KeratinoSens™ cell line contains the luciferase gene under the transcriptional control of a constitutive promoter fused with the ARE element. The luciferase signal indicates the activation of endogenous Nrf2 dependent genes by electrophilic skin sensitizers. Luciferase gene induction is determined quantitatively by measuring luminescence produced by light generating luciferase substrates. Test chemicals are considered skin sensitizers if they induce a statistically significant increase in luciferase activity (i.e. a 50 % increase), below a concentration which does not cause a significant reduction in cell viability.
Read out	Quantitative measurement (by luminescence detection) of luciferase gene induction
Time of exposure	48 h

2) KeratinoSens is the trademark of a product supplied by Givaudan Schweiz AG, CH-8310 Kemptthal. This information is given for the convenience of users of this document and does not constitute an endorsement by ISO of the product named. Equivalent products may be used if they can be shown to lead to the same results.

© ISO 2021 – All rights reserved

Table C.3 *(continued)*

Key event 2: Activation of epidermal keratinocytes	
Applicability	The KeratinoSens™ assay is applicable to test chemicals covering a variety of organic functional groups, reaction mechanisms, skin sensitization potency and physico-chemical properties. In addition, the assay applies to soluble chemicals or those that form stable dispersions (e.g. suspensions or colloids) in the exposure medium.
Application to medical devices	There is no data. KeratinoSens™ works with DMSO and aqueous solvents but can possibly not work with non-polar medical device solvents such as sesame, cottonseed, or olive oil because their log P values exceed 15,0.
See References [77] to [80].	

C.2.2.3 LuSens

The LuSens assay uses an immotalized human keratinocyte cell line harbouring a luciferase reporter gene under control of the antioxidant response element (ARE) of the rat quinone oxidoreductase 1 (NQO1) gene, which is known to be up-regulated by skin sensitizers. See Table C.4.

Table C.4 — LuSens

Key Event 2: Activation of epidermal keratinocytes	
OECD Guideline	OECD TG 442D
Performance (yes/no answer)	74 % accuracy, 74 % sensitivity, 74 % specificity
Experimental system	LuSens transgenic cell line: immortalised adherent cell line derived from human keratinocytes stably harbouring a luciferase reporter gene under the control of the ARE of the rat NQO1 gene.
Principle of the test	The LuSens transgenic cell line contains a luciferase reporter gene under the transcriptional control of a promoter fused with the ARE element. The luciferase signal reflects the activation by electrophiles of endogenous Nrf2 dependent genes. Luciferase gene induction is quantitatively determined by luminescence measurement of light producing luciferase substrates, as an indicator of the activity of the Nrf2 transcription factor in cells following exposure to electrophilic skin sensitizers.
Read out	Quantitative measurement (by luminescence detection) of luciferase gene induction
Time of exposure	48 h
Applicability	The LuSens assay is applicable to test chemicals covering a variety of organic functional groups, reaction mechanisms, skin sensitization potency and physico-chemical properties. In addition, the assay applies to soluble chemicals or those that form stable dispersions (e.g. suspensions or colloids) in the exposure medium.
Application to medical devices	There is no data. LuSens works with DMSO and aqueous solvents but can possibly not work with non-polar medical device solvents such as sesame, cottonseed, or olive oil because their Log P values exceed 15,0.
See References [81] to [84].	

C.2.2.4 SENS-IS

The SENS-IS assay is an in vitro model developed for chemical and mixtures that measures KE2 by assessing gene expression profiles in a human skin models (Episkin® RhE or SkinEthic™ RHE[3)]). SENS-IS allows classification of skin sensitizers into potency categories[84]. The SENS-IS assay has been validated in an industry-led study[86][87] and is currently being evaluated by EURL-ECVAM. See Table C.5.

3) Episkin® RhE and SkinEthic™ RHE (EPISKIN) are examples of suitable products available commercially. This information is given for the convenience of users of this document and does not constitute an endorsement by ISO of these products.

 © ISO 2021 – All rights reserved

Table C.5 — SENS-IS

Key Event 2: Activation of epidermal keratinocytes	
OECD Guideline	Project 4.107: New TG: Toxicogenomic analysis on 3D reconstituted epidermis for measuring skin sensitization potency — the SENS-IS assay
Performance (yes/no answer)	96,6 % accuracy, 97,7 % sensitivity, 95,2 % specificity[86][87]
Experimental system	Reconstructed human epidermis (RhE) models Episkin® RhE or SkinEthic™ RHE (EPISKIN)
Principe of the test	Gene expression of two groups of genes is measured: one group (REDOX group) includes a selection of 17 genes that have an antioxidant responsive element in their promoter and monitor the redox protective signals induced through the interaction of skin sensitizers binding to cysteine amino acids of the Keap1-NRF2 complex[89]. The second group (SENS-IS group) includes a selection of 21 genes involved in inflammation, danger signals and cell migration to address the complex cascade of events leading to activation of DCs by a skin sensitizing chemical.
Read out	Quantitative analysis of 64 gene expression measured by quantitative reverse transcription-polymerase chain reaction (qRT-PCR)
Time of exposure	15 min exposure; the RhE tissues are then rinsed and post-incubated for 6 h.
Applicability	The SENS-IS assay uses 3D RhE tissues to better take into account the step of skin penetration of products with different solubility or physical state. SENS-IS can quantitate not only pure chemicals but also natural products, mixtures, and finished products[90][91].
Application to medical devices	The SENS-IS assays works with polar (saline) and non-polar (sesame oil) extraction vehicles and can possibly be used for testing of medical device extracts. In a proof-of-concept study for assessment of skin sensitization for medical device extracts, five known skin sensitizers incorporated into medical-grade silicone and negative controls were analysed. All test items were extracted in polar (saline) and non-polar (sesame oil) solvents and correctly identified as skin sensitizers or non-sensitizers[88].
See References [85] to [91].	

C.2.2.5 IL-18 RhE assay

For the evaluation of the skin sensitization by measurement of basal IL-18 release after the application of a chemical to the air-liquid interface of the RhE, see Table C.6.

Table C.6 — IL-18 RhE assay

Key event 2: Activation of epidermal keratinocytes	
OECD Guideline	None at this time
Performance (yes/no answer)	On a limited set (<10) of compounds for the different models tested. Andres et al.[92] calculated performance on a set of 19 chemicals. Five prediction models giving different performances are discussed in the paper.
Experimental system	Reconstructed human epidermis models SkinEthic™ RHE (EPISKIN), VUmc-EE, EpiCS®[a] (CellSystems), and EpiDerm™ (MatTek Corp.)[b] [92][93][94][95]
Principle of the test	At the end of chemical exposure, the epidermises were subjected to the cell viability assay and the maintenance media was recovered for the IL-18 ELISA testing.
Read out	Quantification of IL-18 by ELISA in the medium of culture of the RhE; in parallel, cell viability is measured by MTT test
Time of exposure	24 h
Applicability	The assay is applicable for testing soluble contact allergens and cationic metals for skin sensitization potential[94].

[a] VUmc-EE, EpiCS® (CellSystems) is an example of a suitable product available commercially. This information is given for the convenience of users of this document and does not constitute an endorsement by ISO of this product.

[b] Epiderm™ EPI-200 (MatTek Corp.) is an example of a suitable product available commercially. This information is given for the convenience of users of this document and does not constitute an endorsement by ISO of these products.

© ISO 2021 – All rights reserved

Table C.6 *(continued)*

Key event 2: Activation of epidermal keratinocytes	
Application to medical devices	There is no data. Since this assay uses RhE tissues it can possibly work with polar and non-polar medical device extracts.
[a] VUmc-EE, EpiCS® (CellSystems) is an example of a suitable product available commercially. This information is given for the convenience of users of this document and does not constitute an endorsement by ISO of this product.	
[b] Epiderm™ EPI-200 (MatTek Corp.) is an example of a suitable product available commercially. This information is given for the convenience of users of this document and does not constitute an endorsement by ISO of these products.	

C.2.2.6 EpiSensA[4)]

The epidermal sensitization assay "EpiSensA" uses RhE tissue models. The assay is based on the induction of marker genes related to keratinocyte responses for inflammation and cytoprotection in the induction of skin sensitization. See Table C.7.

Table C.7 — EpiSensA

Key event 2: Activation of epidermal keratinocytes	
OECD Guideline	None at this time
Performance (yes/no answer)	90 % accuracy, 94 % sensitivity, 78 % specificity[96]
Experimental system	Reconstructed human epidermis models, LabCyte EPI-MODEL 24 (Japanese Tissue Engineering Co., Ltd.)[a] and Epiderm™ EPI-200 (MatTek Corp.)
Principle of the test	This assay is based on the induction of multiple marker genes (ATF3, IL-8, DNAJB4 and GCLM) related to two keratinocyte responses (inflammatory or cytoprotective) in the induction of skin sensitization. The mechanistic relevance of the marker genes has been confirmed by focusing on key molecules that regulate keratinocyte responses in vitro ($P2X_7$ for inflammatory and Nrf2 for cytoprotective responses). The upregulation of ATF3 and IL-8, or DNAJB4 and GCLM induced by 2,4-dinitrochlorobenzene in human keratinocytes is significantly suppressed by a $P2X_7$ specific antagonist KN-62, or by Nrf2 siRNA, respectively, which supports the mechanistic relevance of the marker genes.
Read out	Quantitative analysis of the expression of four marker genes as measured by quantitative reverse transcription-polymerase chain reaction (RT-PCR)
Time of exposure	6 h exposure
Applicability	EpiSensA can be a mechanism-based skin sensitization test applicable to broad sets of chemicals including lipophilic chemicals and pre/pro-haptens[97].
Application to medical devices	No data. Since this assay uses RhE tissues, it can possibly work with polar and non-polar medical device extracts.
[a] LabCyte EPI-MODEL 24 (Japanese Tissue Engineering Co., Ltd.) is an example of a suitable product available commercially. This information is given for the convenience of users of this document and does not constitute an endorsement by ISO of these products.	

C.2.2.7 SenCeeTox®[5)]

The SenCeeTox® assay measures the expression of 11 skin sensitization related genes in keratinocytes of 3D RhE tissues. Increased gene expression, plus reactivity and cytotoxicity determine skin sensitization potential. See Table C.8.

4) EpiSensA is an example of a suitable product available commercially. This information is given for the convenience of users of this document and does not constitute an endorsement by ISO of this product.

5) SenCeeTox® is an example of a suitable product available commercially. This information is given for the convenience of users of this document and does not constitute an endorsement by ISO of these products.

© ISO 2021 – All rights reserved

Table C.8 — SenCeeTox®

Key event 2: Activation of epidermal keratinocytes	
OECD Guideline	None at this time
Performance (yes/no answer)	84 % accuracy, 81 % sensitivity, 92 % specificity[99]
Experimental system	Reconstructed human epidermis assays EpiDerm™ EPI-200 (MatTek Corp.) and Skin-Ethic™ RhE (EPISKIN)
Principle of the test	The expression of eight Nrf2/ARE, one AhR/XRE and two Nrf1/MRE controlled gene are measured by quantitative reverse transcription-polymerase chain reaction (qRT-PCR). The fold-induction at each exposure concentration is combined with reactivity and cytotoxicity data to determine the sensitization potential.
Read out	Quantitative analysis of the expression of 11 genes is measured by qRT-PCR. Chemical reactivity and cytotoxic potential are also determined. The results of these individual assays are analysed using an algorithm that incorporates all the data from each assay to predict skin sensitization potential.
Time of exposure	24 h
Applicability	The SenCeeTox® assay may be used to evaluate both chemical and metal skin sensitizers of varying potency[98].
Application to medical devices	The SenCeeTox® assays works with polar (saline) and non-polar (sesame oil) extracts vehicles and can possibly be used for testing medical device extracts. In References [98] and [100], it was reported that the SenCeeTox® assay was capable of accurately identifying solutions of certain medical device-related chemical and metal skin sensitizers or sensitizers extracted from medical grade silicone using polar and non-polar solvents such as saline and sesame oil.

C.2.2.8 h-CLAT

The h-CLAT quantifies changes in the expression of cell surface markers associated with the process of activation of monocytes and dendritic cells following exposure to skin sensitizers. The expression levels of the surface markers are used to identify skin sensitizers and non-sensitizers. See Table C.9.

Table C.9 — h-CLAT

Key event 3: Activation of epidermal dendritic cells	
OECD Guideline	OECD TG 442E
Performance (yes/no answer)	85 % accuracy, 93 % sensitivity, 66 % specificity
Experimental system	hCLAT cell line: THP-1 human monocytic leukaemia cell line
Principle of the test	The h-CLAT assay measures changes in the expression of CD86 and CD54 cell surface markers on THP-1 cells after exposure to the test chemical for 24 h. These surface molecules are typical markers of monocytic THP-1 activation and can imitate dendritic cell activation, which plays an important role in T-cell priming. Changes in surface marker expression are measured by fluorescence-based flow cytometry. The relative fluorescence of the surface markers compared to control vehicles are determined and used to differentiate between skin sensitizers and non-sensitizers.[104]
Read out	Expression levels of CD86 and CD54 cell surface markers measured by flow cytometry following cell staining with fluorochrome-tagged antibodies
Time of exposure	24 h exposure
Applicability	The h-CLAT assay is applicable to test chemicals that are soluble in an appropriate solvent or that form stable dispersions in a suitable vehicle. Test chemicals with Log P values above 3,5 have tended to yield false-negative results[104][105].
Application to medical devices	There is no data. The h-CLAT works with saline and DMSO solvents but is unlikely to work with non-polar medical device solvents such as sesame, cottonseed, or olive oil because their log P values exceed 15,0.

© ISO 2021 – All rights reserved

C.2.2.9 U-SENS™ 6)

The U937 cell line activation test (U-SENS™) method is an in vitro assay that quantifies changes of CD86 cell surface marker expression by flow cytometry on a human histiocytic lymphoma cell line, U937 cells. See Table C.10.

Table C.10 — U-SENS™

U-SENS™ — Key event 3: Activation of epidermal dendritic cells	
OECD Guideline	OECD TG 442E
Performance (yes/no answer)	86 % accuracy, 91 % sensitivity, 65 % specificity
Experimental system	U-SENS™ cell line: U937, human histiocytic lymphoma cell line
Principle of the test	CD86 is known to be a co-stimulatory molecule that can mimic monocytic activation, which plays a critical role in T-cell priming. The changes of CD86 cell surface marker expression are measured by flow cytometry following cell staining typically with fluorescein isothiocyanate (FITC)-labelled antibodies. Cytotoxicity measurement is also conducted (e.g. by using PI) concurrently to assess whether up regulation of CD86 cell surface marker expression occurs at sub-cytotoxic concentrations. The stimulation index (SI) of CD86 cell surface marker compared to solvent/vehicle control is calculated and used in the prediction model, to support the discrimination between skin sensitizers and non-sensitizers. [104]
Read out	Changes of CD86 cell surface marker expression are measured by flow cytometry following cell staining typically with fluorescein isothiocyanate (FITC)-labelled antibodies.
Time of exposure	(45 ± 3) h exposure
Applicability	Applicable to test chemicals that are soluble or that form a stable dispersion (i.e. a colloid or suspension in which the test chemical does not settle or separate from the solvent/vehicle into different phases) in an appropriate solvent/vehicle.[104]
Application to medical devices	There is no data. U-SENS works with saline and DMSO solvents but is unlikely to work with non-polar medical device solvents such as sesame, cottonseed, or olive oil because their log P values exceed 15,0.

C.2.2.10 IL-8 Luc assay

The interleukin-8 reporter gene assay (IL-8 Luc assay) makes use of a THP-1-derived IL-8 reporter cell (THP-G8 cells) that allows quantitative measurement of luciferase gene induction by detecting luminescence from well-established light producing luciferase substrates as an indicator of the activity of the IL-8 and GAPDH in cells following exposure to skin sensitizing chemicals. See Table C.11.

6) U-SENS is the trademark of a product supplied by L'Oréal, 41 rue Marthe, Clichy, 92110. This information is given for the convenience of users of this document and does not constitute an endorsement by ISO of the product named. Equivalent products may be used if they can be shown to lead to the same results.

 © ISO 2021 – All rights reserved

Table C.11 — IL-8 Luc assay

Key event 3: Activation of epidermal dendritic cells	
OECD Guideline	OECD TG 442E
Performance (yes/no answer)	86 % accuracy, 96 % sensitivity, 41 % specificity
Experimental system	IL8 Luc cell line: THP-1-derived IL-8 reporter cell line (THP-G8)
Principle of the test	The IL-8 Luc assay uses a THP-1-derived IL-8 reporter cell line, THP-G8, that harbours the stable luciferase orange (SLO) and stable luciferase red (SLR) luciferase genes under the control of the IL-8 and glyceraldehyde 3-phosphate dehydrogenase (GAPDH) promoters, respectively (1). This allows quantitative measurement of luciferase gene induction by detecting luminescence from well-established light producing luciferase substrates as an indicator of the activity of the IL-8 and GAPDH in cells following exposure to skin sensitizing chemicals.[104]
Read out	Changes in IL-8 expression, a cytokine associated with the activation of dendritic cells
Time of exposure	16 h exposure
Applicability	Although the IL-8 Luc assay uses X-VIVO™ 15 as a solvent, it correctly evaluated chemicals with the n-octanol-water partition coefficient log K_{ow} > 3,5 and those with a water solubility of around 100 µg/ ml. However, negative results for test chemicals that are not dissolved at 20 mg/ml may produce false-negative results due to their inability to dissolve in XVIVO™15. Therefore, negative results for these chemicals should not be considered. A high false negative rate for anhydrides was seen in the validation study. Furthermore, because of the limited metabolic capability of the cell line (8) and the experimental conditions, pro-haptens (substances requiring metabolic activation) and pre-haptens (substances activated by air oxidation) can give negative results in the assay. The IL-8 Luc assay can classify chemicals as skin sensitizers or non-sensitizers, however, at this time it does not provide potency assessments[105].
Application to medical devices	There is no data. The X-VIVO™ 15 culture medium, DMSO, or water are used as solvents to dissolve the test chemicals. It is unknown whether the assay will work with non-polar medical device solvents such as sesame, cottonseed, or olive oil.

C.2.2.11 Genomic allergen rapid detection™ 7)

Genomic allergen rapid detection™ (GARD™) is a cell-based assay that utilizes the innate recognition of foreign substances by dendritic cells, as measured by a multivariate readout of genomic biomarkers. Following cellular stimulation, chemicals are classified as skin sensitizers or non-sensitizers based on induced transcriptional profiles. See Table C.12. The GARD assay has been validated in an industry-led study[118] and is currently being evaluated by EURL-ECVAM, and have received a positive scientific ESAC opinion on GARD™ skin.

7) GARD is the trademark of a product supplied by SenzaGen AB, Medicon Village (406), 22381 Lund, Sweden. This information is given for the convenience of users of this document and does not constitute an endorsement by ISO of the product named. Equivalent products may be used if they can be shown to lead to the same results.

© ISO 2021 – All rights reserved

Table C.12 — GARD™

Key event 3: Activation of dendritic cells	
OECD Guideline	Project 4.106: New TG: GARD™skin test: An in vitro method for identification of skin sensitizers based on a genomic interpretation of the impact of chemicals on human dendritic cell-like cells (AOP key event 3)[121]
Performance (yes/no answer)	94 % accuracy, 93 % sensitivity, 96 % specificity[118]<br>NOTE The GARD™skin assay can also be complemented with the GARD™potency to get classification according to European classification, labelling, and packaging (CLP), Regulation (e.g. weak or strong skin sensitizer).
Experimental system	The GARD™skin cell line: SenzaCells™ (SenzaGen) is of human myeloid origin with characteristics similar to dendritic cells.
Principle of the test	GARD™skin measures the gene expression of 200 genes induced in SenzaCells™ in response to chemical exposure. The 200 genes, referred to as GARD prediction signature (GPS) includes biomarkers for dendritic cell activation and maturation (KE3), several danger signal pathways and pattern recognition receptors (KE2), and antigen presenting molecules and cell proliferation pathways (KE4)[116][117].
Read out	The GPS includes mechanistically relevant genes involved in the skin sensitization process, such as upregulation of co-stimulatory molecules and induction of cellular and oxidative stress[115]. Gene expression of the 200 genes in the GPS is measured using the Nanostring®[a] analysis system. The resulting gene expression is analysed by pattern recognition, using a machine learning algorithm based on a fixed set of reference samples.[114] Each sample is classified as skin sensitizer or non-sensitizer with no subjective judgement required.
Time of exposure	24 h exposure
Applicability	GARD™skin is widely applicable across a broad chemical space covering a variety of organic functional groups and end uses including pre-/pro-haptens and substances with low water solubility[113][116].
Application to medical devices	The GARD™ assays works with polar (saline) and non-polar (sesame oil and olive oil) extraction vehicles and can possibly be used for testing of medical device extracts. In a proof-of-concept study for assessment of skin sensitization for medical device extracts, four known sensitizers incorporated into medical-grade silicone and polyurethane and negative controls were analysed. All test items were extracted in polar (saline) and non-polar (oil) solvents and correctly identified as skin sensitizers or non-sensitizers.[119]

[a] Nanostring® is an example of a suitable product available commercially. This information is given for the convenience of users of this document and does not constitute an endorsement by ISO or IEC of this product.

C.3 Discussion

C.3.1 OECD-validated assays

The six validated OECD 442 test guidelines in vitro assays[75][79][104] for identifying chemical skin sensitizers, which address key events 1, 2, and 3, appear to have potential utility for detecting chemical sensitizers in medical device extracts. They are the DPRA, KeratinoSens™, LuSens, h-CLAT, U-SENS™, and IL-8 Luc assay. However, since DPRA uses an excess of chemical to detect peptide reactivity, it can possibly not be capable of identifying low levels of sensitizers in dilute medical device extracts. These assays have been validated using pure test substances and not chemical mixtures. Because of the limited metabolic capability of the cell lines used in some in vitro assays, pro-haptens (i.e. substances requiring enzymatic activation for example via P450 enzymes) may also provide false-negative results.[82][104] Lastly, these test methods developed to address a specific key event may not alone be sufficient to conclude on the presence or absence of skin sensitization potential of chemicals and should be considered in the context of integrated approaches such as IATA, combining them with other complementary information.

Considering the specific context of medical devices biocompatibility assays used to evaluate finished products or materials either as is or after extraction in polar and non-polar solvents, the applicability of the OECD assays shall be confirmed. Many of them may have some limitations with non-polar medical

 © ISO 2021 – All rights reserved

device extraction solvents such as sesame, cottonseed, or olive oil. Besides the issue of mixtures and solvent incompatibility, some OECD-validated assays can possibly not be able to detect potential skin sensitizers in medical device extracts because they often are present at extremely low concentrations.

C.3.2 Genomic assays

The genomic assays listed in C.2.2, which address key events 2 and 3, are SenCeeTox®, SENS-IS, EpiSensA and GARD™. The first three work with RhE tissues, while the fourth is based on a proprietary cell line. They are all somewhat different, yet the common trait they share is their use of marker genes to identify skin sensitizers. In addition, all assays, except EpiSensA, have successfully completed pilot projects using polar and non-polar solvent extracts of medical device polymers spiked with skin sensitizers. The accuracy reported on the limited set of samples for the pilot projects has been comparable or better than the accuracy of the OECD-validated assays. Two of these assays, SENS-IS, and GARD™, are currently being reviewed under OECD's Test Guidelines Programme Section 4[121] (projects related to test the guidelines on health effects).

Based on their accuracy, their ability to work with polar and non-polar solvents, and potential to be "stand alone" methods for medical device skin sensitization testing, these assays may be performant methods for evaluating the skin sensitization potential of medical devices. Still, their ability to detect cationic metal sensitizers needs to be confirmed.

C.3.3 Other assays

The IL-18 RhE assay is the remaining assay addressing key event 2. This assay measures IL-18 release and cell viability. Testing data for the IL-18 RhE assay is limited. Since this assay use RhE tissues, it can possibly work with polar and non-polar medical device solvents. Still, since this assay has not been validated by OECD, or included in any of the major in vitro skin sensitization assay comparison studies, its use in medical device testing should be confirmed.

C.3.4 General considerations for validation of in vitro methods for medical device testing

There are currently six validated in chemico and in vitro assays (DPRA, Keratinosens, LuSens, h-Clat, U-Sens, IL-8 Luc Assay) in the OECD test guidelines for determining the skin sensitization potential of chemicals and two more (GARD™, SENS-IS) [75][79][104] included in the OECD Test Guideline Program[121]. The inclusion of any of these in vitro skin sensitization tests for medical device testing should be supported by the validation data confirming the equivalence/superiority of the in vitro method compared to the current in vivo methods used to evaluate the skin sensitization potential of medical devices/medical device extracts.

Such validation process shall be designed to allow performance comparisons between the candidate assays and to investigate their use as standalone or in combination within a testing strategy.

The following general points shall be considered in designing such validation studies:

— deciding whether to use skin sensitizing reference materials or extracts of non-sensitizing materials in which sensitizers will be added at known concentrations;

— identifying representative classes of skin sensitizing chemicals that may be present in medical devices;

— selecting a list of reference chemicals to build a validation database containing physicochemical charateristics, historical data from animal and human methods as well as in vitro and in chemico data from validated OECD assays;

— identifying negative and positive control materials to be used in the validation;

— defining the minimum reliability (within, between reproducibility) and accuracy (sensitivity, specificity) values compared to human and/or animal methods to validate an in vitro assay;

© ISO 2021 – All rights reserved

— identifying any chemical or physical properties of the medical device materials that can result in test interferences, false negative or false-positive results;

— defining the applicability domain of the considered in vitro assays e.g. for materials suitable for extraction or materials not suitable for extraction (e.g. liquids, gels, pastes, gases, vapors, aerosols, condensates, nanomaterials).

C.4 Conclusions

Approximately 20 % of the chemicals in world commerce are sensitizers.[72] Yet, historically only about 1 % of medical device extracts produce positive results in animal skin sensitization tests[94]. These facts, plus the availability of sophisticated in vitro skin sensitization assays, have led experts to believe that a paradigm shift towards acceptance of in vitro assays for medical device testing is inevitable.[73] Nevertheless, for this to happen well-designed evaluation/validation studies of the most promising in vitro skin sensitization assays or combination of assays will be necessary.

© ISO 2021 – All rights reserved

Annex D
(informative)

Background information on sensitization tests for skin sensitization

Sensitization in man occurs after single or multiple epicutaneous exposures and is initiated and elicited by components of the immune system. First, the hapten (chemical) must penetrate the skin. It then reacts with skin proteins to form immunogenic complexes. Langerhans cells at the epidermal/dermal border present the antigen to specific lymphocytes which are then activated to initiate the immune responses. A small percentage of these lymphocytes are long-lived memory cells, and these serve as the primary activators during the challenge phase. Thus, subsequent re-exposures can result in adverse reactions that are mediated by lymphokines released by the activated lymphocytes and other inflammatory cells that are attracted to the area of the lesion.

In 1895, Jadassohn employed the patch test to disclose contact allergy to mercury in a clinical patient. This innovative approach provided the scientific basis for subsequent tests aimed at diagnosis and prediction of contact allergy in man and animals. The development of prospective/predictive tests for evaluating the sensitizing potential of chemicals followed the pioneering work of Landsteiner and Chase[11], who firmly substantiated the use of the guinea pig for studying skin sensitization.

In 1969, Magnusson and Kligman[12] explored many of the variables of guinea pig testing and presented a procedure, the GPMT, based on intradermal injections (with and without Freund's complete adjuvant), followed by topical application of the test material to the same area. The original procedure requires pretreatment of the test site if the test material is a non-irritant. By definition, it reputedly detects weak skin sensitizers, because "weak" included a zero incidence of positive reactors. It is a sensitive test and has been extensively used. The use of Freund's complete adjuvant increases the sensitivity of the test method and it can in some cases overestimate the sensitizing potential of the compound in question.

In 1965, Buehler[7] advocated the use of the closed patch test to provide occlusion as a method to optimize exposure and to mimic the procedures used in humans [human repeat insult patch test (HRIPT)]. It was suggested that the occlusive patch procedure was sensitive and can accurately predict moderate to severe skin sensitizers, thus avoiding exposure of human subjects to the prospect of adverse reaction during HRIPTs. The data presented demonstrated the superiority of occlusion over intradermal injections and open-type topical application. Stimulation of the immune system by adjuvants was not used. This method is established as a technique that is sufficiently sensitive to detect most weak skin sensitizers and has been shown to be sufficiently flexible to be used in the risk assessment process. However, the closed-patch test (Buehler test) is less sensitive compared with the GPMT. See Reference [9].

These two tests, the closed-patch test in the United States and the GPMT in Europe, have been the most frequently used for safety assessment. The result from guinea pig sensitization assays depends on many animal-related and technical factors explaining the interlaboratory variation in test results, for example, animal strain, sex, age, ambient test conditions, test site on the animal, method of hair removal (clipping/shaving or chemical depilation), type of patch design, quantity of test material, quality of occlusion, exposure time and reading of the tissue response. Numerous other tests have been employed and investigated and all of these have their proponents. There are currently several procedures that have been recognized as acceptable for regulatory purposes, provided the procedure is properly documented and validated by the investigator. In all cases, the procedures should be performed in accordance with the original references. A list of other tests is given below.

Reference [8] gives an update on skin sensitization testing.

a) Freund's complete adjuvant test.

© ISO 2021 – All rights reserved

b) Split adjuvant test.

c) Open epicutaneous test.

d) Mauer optimization test.

e) Footpad test in guinea pig.

f) Cumulative contact enhancement test.

g) Scratched skin (adjuvant and patch) test.

h) Mouse ear swelling test.

The LLNA is now preferred to the GPMT and the Buehler occluded patch test for hazard identification of chemicals. LLNA has been accepted in 2010 by the OECD as a stand-alone alternative to the current guinea pig tests, and as an improvement for animal welfare. See Reference [33]. The LLNA has been validated for the determination of sensitizing activity of chemicals. See References [39] and [40].

The scientific basis for the test is measurement of the incorporation of ^{3}H-methyl thymidine into lymphocytes in draining lymph nodes of mice topically exposed to the test sample as a measurement of sensitization. It does not include a challenge phase. The end point of interest is a stimulation index giving the ratio of thymidine incorporation in lymph nodes from dosed animals compared with the incorporation in lymph nodes from control animals. The test is positive when the stimulation index exceeds 3 ($I_s > 3$). An intra- and inter-laboratory evaluation of the LLNA has demonstrated a reproducible dose-response relationship within and between laboratories. See References [16], [17], [21], [25], [26], [28] and [32]. However, difficulties in differentiating between irritating and allergenic substances with the LLNA have been reported. See References [18], [25] and [28]. Thus, the LLNA can give false-positive results with irritants and can overestimate the allergenicity of substances with both irritating and allergenic properties. See Reference [16]. However, the LLNA has advantages compared with the guinea pig assays because of shorter test duration, a more objective end point, less test substance required, and it omits the Freund's complete adjuvant injections. Improvements of the test procedure by use of analysis of cell activation markers and flow cytometry are possible. See References [23] and [24]. Whether they can be practically implemented in standard LLNA protocols for routine toxicology is not determined. On the other hand, the LLNA allows a more limited choice of test vehicles; most studies have used a mixture of acetone and olive oil. A recent study shows the variability of the results using different vehicles. See Reference [27]. Further, it is not possible with the LLNA to study the challenge phase or cross-reactivity patterns because the animals are sacrificed after induction treatment before the lymph nodes are harvested.

The popliteal lymph node assay (PLNA) using subcutaneous administration in the footpad (see References [19], [22] and [31]) is an alternative to the lymph node assay. In the latter assay, in addition to direct measurement of lymph node activation, reporter antigens may be used for further clarification of the immunomodulation caused by the chemical under investigation. See Reference [15].

The risk assessment process should not rely on a single model or approach but should be thoughtfully conducted to provide maximum assurance of safety to the consumer. Generally, this entails both animal or non-animal methods and human experimental models. There should be flexibility in the choice of models and approaches, as long as the rationale is documented and/or validated.

Negative tests in guinea pigs, when they are properly conducted, can generally be definitive if the test concentration has a sufficient safety factor over use conditions. However, one should avoid classifying test materials solely on the basis of incidence and/or severity, without due consideration of eventual product usage.

The risk, i.e. incidence and severity, of the allergic reaction to the product is determined mainly with the following four factors: the sensitizing potency of the chemical allergen, its amount in the product, bioavailability and the exposure conditions. The relative sensitizing potencies of chemicals can be defined in terms of the minimum induction concentration required to induce a given level of sensitization: the lower this concentration the more potent the sensitizer. See References [6] and [30]. It was shown that the significant incidence of allergic contact dermatitis was found in users when the

 © ISO 2021 – All rights reserved

residue level of the allergen in the product exceeded its minimum induction concentration obtained by GPMT. See Reference [14].

On the other hand, predictive testing of mixtures and products is much less validated and can be performed following testing of product ingredients. Accordingly, test design and result interpretation are subject to uncertainty, but several examples document this possibility. In animal experiments with acetone extracts from a sweater that had caused contact dermatitis in humans, allergens (phosgene chlorophenylhydrazones) were demonstrated. See Reference [11]. In another case, where animal experiments with acetone/chloroform extracts from rubber boots had caused contact dermatitis in man, mercaptobenzothiazole and dibenzothiazyldisulfide were eventually found to be the causative allergens. See Reference [10]. The importance of using an appropriate organic solvent was clearly demonstrated. The extracts made with organic solvent induced skin sensitization in the guinea pigs, while the saline extracts failed to do so.

Reference [4] adopts the sample preparation procedure with organic solvent followed by evaporation of the solvent to obtain the residue, and the risk assessment procedure by comparing the percent residue yield from the material with the minimum percent dilution of the residue (mixture) that still induced skin sensitization in animals.

In vitro methods for skin sensitization testing are not yet available for routine use, but in view of new regulations in Europe which ban the use of animal tests for cosmetics, it seems likely that novel strategies will become available for the identification of skin sensitizers. See Annex C.

© ISO 2021 – All rights reserved

Bibliography

General references for skin sensitization tests

[1] Organization for Economic Cooperation and Development (OECD), Guidelines for the testing of chemicals No. 406, Skin sensitization, OECD Publications, 1992

[2] de Silva O., Basketter D.A., Barratt M.D. et al. *Alternative methods for skin sensitization testing*, The Report and Recommendations of ECVAM Workshop 19, ATLA, 24, pp. 683-705, 1996

[3] MHLW Notification by Director, MDED, Yakuseikishin-hatsu 0106 No.1, Jan. 6, 2020. Amendment of Basic Principles of Biological Safety Evaluation Required for Application for Approval to Market Medical Devices

[4] *Japanese Guidelines of Basic Biological Tests of Medical Materials and Devices*, 1995

Bibliography for skin sensitization tests

[5] Organization for Economic Cooperation and Development (OECD), The Adverse Outcome Pathway for Skin Sensitisation Initiated by Covalent Binding to Proteins, OECD Series on Testing and Assessment, n° 168, Éditions OCDE, Paris, 2014, https://doi.org/10.1787/9789264221444-en

[6] Andersen K.E., Vølund A., Frankild S., The guinea pig maximization test with a multiple dose design, Acta Derm. Venereol., **75**, pp. 463-469, 1995

[7] Buehler E.V., Delayed contact hypersensitivity in the guinea pig, Arch. Dermatol., **91**, pp. 171-175, 1965

[8] European Chemical Industry Ecology and Toxicology Centre *Skin sensitization testing for the purpose of hazard identification and risk assessment*, Monograph 29, Brussels, Belgium, 2000

[9] Frankild S., Vølund A., Wahlberg J.E. et al., Comparison of the sensitivities of the Buehler test and the guinea pig maximization test for predictive testing of contact allergy, Acta Derm. Venereol., **80**, pp. 256-262, 2000

[10] Kaniwa M.A., Momma J., Ikarashi Y. et al., A method for identifying causative chemicals of allergic contact dermatitis using a combination of chemical analysis and patch testing in patients and animal groups: application to a case of rubber boot dermatitis, Contact Dermatitis, **27**, pp. 166-173, 1992

[11] Kojima S., Momma J., Kaniwa M.A., Phosgene (chlorophenyl) hydrazones, strong sensitizers found in yellow sweaters bleached with sodium hypochlorite, defined as causative allergens for contact dermatitis by an experimental screening method in animals, Contact Dermatitis, **23**, pp. 129-141, 1990 [published erratum appears in Contact Dermatitis, 23, p. 383]

[12] Landsteiner K., Chase M.W., Studies on the sensitization of animals with simple chemical compounds, J. Exp. Med., **69**, p. 767, 1939

[13] Magnusson B., Kligman A.M., The identification of contact allergens by animal assay. The guinea pig maximization test, J. Invest. Dermatol., **52**, pp. 268-276, 1969

[14] Nakamura A., Momma J., Sekignchi H. et al., A new protocol and criteria for quantitative determination of sensitization potencies of chemicals by guinea pig maximization test, Contact Dermatitis, **31**, pp. 72-85, 1994

Bibliography for LLNA

© ISO 2021 – All rights reserved

[15] Albers R., Broeders A., Van Der Pijl A. et al., The use of reporter antigens in the popliteal lymph node assay to assess immonomodulation by chemicals, Toxicol. Appl. Pharmacol., **143**, pp. 102-109, 1997

[16] Basketter D.A., Lea L.J., Cooper K. et al., Threshold for classification as a skin sensitizer in the local lymph node assay: a statistical evaluation, Food. Chem. Toxicol., **37**, pp. 1167-1174, 1999

[17] Basketter D.A., Roberts D.W., Cronin M. et al., The value of the local lymph node assay in quantitative structure-activity investigations, Contact Dermatitis, **27**, pp. 137-142, 1992

[18] Basketter D.A., Scholes E.W., Kimber I., The performance of the local lymph node assay with chemicals identified as contact allergens in the human maximization test, Food. Chem. Toxicol., **32**, pp. 543-547, 1994

[19] De Bakker J.M., Kammüller M.E., Muller E.S.M. et al., Kinetics and morphology of chemically induced popliteal lymph node reactions compared with antigen-mitogen-, and graft-versus-host-reaction-induced-responses, Virchows Archiv. B Cell Pathol., **58**, pp. 279-287, 1990

[20] Dean, J., Twerdok, L.E., Andersen, K.E. et al., *The murine local lymph node assay: A test method for assessing the allergic contact dermatitis potential of chemicals/compounds*, NIH publication No. 99-494, Research Triangle Park, 1999, available at https://ntp.niehs.nih.gov/iccvam/docs/immunotox_docs/llna/llnarep.pdf

[21] Dearman R.J., Basketter D.A., Kimber I., Local lymph node assay: use in hazard and risk assessment, J. Appl. toxicol., **19**, pp. 299-306, 1999

[22] Descotes J., Patriarca C., Vial T. et al., The popliteal lymph node assay in 1996, Toxicol., **119**, pp. 45-49, 1997

[23] Gerberick G.F., Gruse L.W., Ryan C.A., Local lymph node assay: differentiating allergic and irritant responses using flow cytometry, Methods, **19**, pp. 48-55, 1999(a)

[24] Gerberick G.F., Gruse L.W., Miller C.M. et al., Selective modulation of B-cell activation markers CD86 and I-AK on murine draining lymph node cells following allergen or irritant treatment, Toxicol. Appl. Pharmacol, **159**, pp. 142-151, 1999(b)

[25] Ikarashi Y., Tsuchiya T., Nakamura A., A sensitive mouse lymph node assay with two application phases for detection of contact allergens, Arch. Toxicol., **67**, pp. 629-636, 1993

[26] Kimber I., Hilton J., Dearman R.J. et al., An international evaluation of the murine local lymph node assay and comparison of modified procedures, Toxicol., **103**, pp. 63-73, 1995

[27] Lea L.J., Warbrick E.V., Dearman R.J. et al., The impact of vehicle on assessment of relative skin sensitization potency or 1,4-dihydroquinone in the local lymph node assay, Am. J. Contact Dermatitis, **10**, pp. 213-218, 1999

[28] Loveless S.E., Ladics G.S., Gerberick G.F. et al., Further evaluation of the local lymph node assay in the final phase of an international collaborative trial, Toxicol., **108**, pp. 141-52, 1996

[29] Montelius J., Wahlkvist H., Boman A. et al., Experience with the murine local lymph node assay: inability to discriminate between allergens and irritants, Acta Derm. Venereol., **74**, pp. 22-27, 1994

[30] Roberts D.W., Structure-activity relationships of skin sensitization potential of diacrylates and dimethacrylates, Contact Dermatitis, **17**, pp. 281-289, 1987

[31] Vial T., Carleer J., Legrain B. et al., The popliteal lymph node assay: results of a preliminary interlaboratory validation study, Toxicol., **122**, pp. 213-218, 1997

[32] Warbrick E.V., Dearman R.J., Lea L.J. et al., Local lymph node assay responses to paraphenylenediamine: intra- and inter-laboratory evaluations, J. Appl. Toxicol., **19**, pp. 225-260, 1999

© ISO 2021 – All rights reserved

[33] Organization for Economic Cooperation and Development (OECD), Guideline for the testing of chemicals No. 429, Skin sensitisation: Local lymph node assay, OECD Publications, 2010

[34] Ryan C.A., Cruse L.W., Skinner R.A. et al., Examination of a vehicle for use with water soluble materials in the murine local lymph node assay, Food Chem Toxicol., **40**, pp. 1719-1725, 2002

[35] Woolhiser M.R., Munson A.E., Meade B.J., Comparison of mouse strains using the local lymph node assay, Toxicology **146**, pp. 221-227, 2000

[36] Takeyoshi M., Noda S., Yamasaki K. et al., Advantage of using CBA/N strain mice in a non-radioisotopic modification of the local lymph node assay, J. Appl. Toxicol., **26**, pp. 5-9, 2006

[37] Van Och F.M.M., Slob W., De Jong W.H. et al., A quantitative method for assessing the sensitizing potency of low molecular weight chemicals using a local lymph node assay: employment of a regression method that includes determination of the uncertainty margins, Toxicology, **146**, pp. 49-59, 2000

[38] De Jong W.H., Van Och F.M.M., Den Hartog Jager C.F. et al., Ranking of allergenic potency of rubber chemicals in a modified local lymph node assay, Toxicol. Sc., **66**, pp. 226-232, 2002

[39] Dean J.H., Twerdok L.E., Tice R.R. et al., ICCVAM evaluation of the murine local lymph node assay. II Conclusions and recommendations of an independent scientific peer review panel, Regul. Toxicol. Pharmacol., **34**, pp. 258-273, 2001

[40] Hanek K.E., Tice R.R., Carson B.L. et al., ICCVAM evaluation of the murine local lymph node assay. III Data analysis completed by the National Toxicology Program Interagency Center for the Evaluation of Alternative Toxicological Methods, Regul. Toxicol. Pharmacol., **34**, pp. 274-286, 2001

[41] ASTM F2148-13, *Standard Practice for Evaluation of Delayed Contact Hypersensitivity Using the Murine Local Lymph Node Assay (LLNA)*

[42] Organization for Economic Cooperation and Development (OECD), Test No. 442B: Skin Sensitization: Local Lymph Node Assay: BrdU-ELISA or –FCM, OECD Guidelines for the Testing of Chemicals, Section 4, Éditions OCDE, Paris, 2018, https://doi.org/10.1787/9789264090996-en

[43] Lee J.K., Park J.H., Park S.H. et al., A nonradioisotopic endpoint for measurement of lymph node cell proliferation in a murine allergic contact dermatitis model, using bromodeoxyuridine immunohistochemistry, J. Pharmacol. Toxicol. Methods, **48**, pp. 53-61, 2002

[44] ICCVAM, 2010. ICCVAM Test Method Evaluation Report on Using the LLNA for Testing Pesticide Formulations, Metals, Substances in Aqueous Solutions, and Other Products. NIH Publication No. 10-7512. Research Triangle Park, NC: National Institute of Environmental Health Sciences.

Bibliography for in vitro skin sensitization test

[45] Basketter D., Maxwell G., *In vitro* approaches to the identification and characterization of skin sensitizers, Cutaneous and Ocular Toxicol., **26**, pp. 359-373, 2007

[46] Ashikaga T., Yoshida Y., Hirota M et al., Development of an *in vitro* skin sensitization test using human cell lines: The human Cell Line Activation Test (h-CLAT) I. Optimization of the h-CLAT protocol, Toxicology in vitro, **20**, pp. 767-773, 2006

[47] Sakaguchi H., Ashikaga T., Miyazawa M. et al., Development of an *in vitro* skin sensitization test using human cell lines: the human Cell Line Activation Test (h-CLAT) II. An inter-laboratory study of the h-CLAT, Toxicology in vitro, **20**, pp. 774-784, 2006

[48] Ashikaga T., Sakaguchi H., Okamoto K. et al., Assessment of the human Cell Line Activation Test (h-CLAT) for Skin Sensitization; Results of the First Japanese Inter-laboratory Study, Alternatives to Animal Testing and Experimentation, **13**, pp. 27-35, 2008

 © ISO 2021 – All rights reserved

[49] EURL CVAM, European Union Reference Laboratory for alternatives to animal testing, https://ec.europa.eu/jrc/en/eurl/ecvam

[50] Gibbs S, Kosten I, Veldhuizen R, Spiekstra S, Corsini E, Roggen E, Rustemeyer T, Feilzer AJ, Kleverlaan CJ, Assessment of metal sensitizer potency with the reconstructed human epidermis IL-18 assay. Toxicology. **393**, pp. 62-72, 2018

[51] HAUGEN, E. and HENSTEN-PETTERSEN, A., The sensitizing potential of periodontal dressings. J. Dent. Res., **57**, pp. 950-953. 1978

[52] Johansson H, Gradin R., Forreryd A, Agemark M., Zeller K., Johansson A., Larne O., van Vlier E., Borrebaeck C., Lindstedt M., Evaluation of the GARD assay in a blind Cosmetics Europe study. ALTEX, **34**, pp. 515-523. 2017

[53] Roberts D.W., Is a combination of assays really needed for non-animal prediction of skin sensitization potential? Performance of the GARD™ (Genomic Allergen Rapid Detection) assay in comparison with OECD guideline assays alone and in combination. Regul Toxicol Pharmacol, **98**, pp. 155-160. 2018

[54] Cottrez F, Boitel E, Ourlin JC, Peiffer JL, Fabre I, Henaoui IS, Mari B, Vallauri A, Paquet A, Barbry P, Auriault C, Aeby P, Groux H, SENS-IS, a 3D reconstituted epidermis based model for quantifying chemical sensitization potency: Reproducibility and predictivity results from an inter-laboratory study. Toxicol In Vitro, **32**, pp. 248-260, 2016

[55] Kimber I., Dearman R.J., Betts C.J., Gerberick G.F., Ryan C.A., Kern P.S., Patlewicz G.Y., Basketter D.A., The local lymph node assay and skin sensitization: a cut-down screen to reduce animal requirements? Contact Dermatitis, **54**, pp. 181-185. 2006

Bibliography for in vitro assays for sensitization testing (general)

[56] Ankley G. T., Bennett R. S., Erickson R. J., Hoff D. J., Hornung M. W., Johnson R. D., Mount D. R., Nichols J. W., Russom C. L., Schmieder P. K., Serrano J. A., Tietge J. E., Villeneuve D. L.2010. , Adverse outcome pathways: A conceptual framework to support ecotoxicology research and risk assessment. Environmental Toxicology and Chemistry **29**:730-741

[57] Casati S.2017. , Contact hypersensitivity: Integrated approaches to testing and assessment. Current Opinion in Toxicology **5**:1-5

[58] Ezendam J., Braakhuis H. M., Vandebriel R. J.2016. , State of the art in non-animal approaches for skin sensitization testing: from individual test methods towards testing strategies. Arch Toxicol. **90**(12):2861-2883

[59] Hoffmann S., Kleinstreuer N., Alépée N., Allen D., Api A. M., Ashikaga T., Clouet E., Cluzel M., Desprez B., Gellatly N., Goebel C., Kern P. S., Klaric M., Kühnl J., Lalko J. F., Martinozzi-Teissier S., Mewes K., Miyazawa M., Parakhia R., van Vliet E., Zang Q., Petersohn D.2018. , Non-animal methods to predict skin sensitization (I): the Cosmetics Europe database. Crit Rev Toxicol. **48**(5):344-358

[60] Kimber I., Travis M. A., Martin S. F., Dearman R. J., Immunoregulation of skin sensitization and regulatory T cells. 2012. Contact Dermatitis **67**(4):179-83

[61] Kleinstreuer N. C., Hoffmann S., Alépée N., Allen D., Ashikaga T., Casey W., Clouet E., Cluzel M., Desprez B., Gellatly N., Göbel C., Kern P. S., Klaric M., Kühnl J., Martinozzi-Teissier S., Mewes K., Miyazawa M., Strickland J., van Vliet E., Zang Q., Petersohn D.2018. , Non-animal methods to predict skin sensitization (II): an assessment of defined approaches. Crit Rev Toxicol. **48**(5):359-374

[62] Martin S. F., Esser P. R., Weber F. C., Jakob T., Freudenberg M. A., Schmidt M., Goebeler M.2011. , Mechanisms of chemical-induced innate immunity in allergic contact dermatitis. Allergy **66**(9):1152-63

© ISO 2021 – All rights reserved

[63] OECD, 2017. Revised Guidance Document on Developing and Assessing Adverse Outcome Pathways. Series on Testing & Assessment No. 184. ENV/JM/MONO(2013)6. Organization for Economic Co-operation and Development. Paris. 32 pp. Available at: http://search.oecd.org/officialdocuments/displaydocumentpdf/?cote=env/jm/mono(2013)6&doclanguage=en

[64] Organization for Economic Cooperation and Development (OECD), Guidance Document on the Reporting of Defined Approaches to be Used Within Integrated Approaches to Testing and Assessment, OECD Series on Testing and Assessment, n° 255, Éditions OCDE, Paris, 2017, Available at: https://doi.org/10.1787/9789264274822-en

[65] Reisinger K., Hoffmann S., Alépée N., Ashikaga T., Barroso J., Elcombe C., Gellatly N., Galbiati V., Gibbs S., Groux H., Hibatallah J., Keller D., Kern P., Klaric M., Kolle S., Kuehnl J., Lambrechts N., Lindstedt M., Millet M., Martinozzi-Teissier S., Natsch A., Petersohn D., Pike I., Sakaguchi H., Schepky A., Tailhardat M., Templier M., van Vliet E., Maxwell G.2015. , Systematic evaluation of non-animal test methods for skin sensitisation safety assessment. Toxicol In Vitro **29**(1):259-70

[66] Strickland J., Zang Q., Kleinstreuer N., Paris M., Lehmann D. M., Choksi N., Matheson J., Jacobs A., Lowit A., Allen D., Casey W.2016. , Integrated decision strategies for skin sensitization hazard. J Appl Toxicol. **36**(9):1150-62

[67] Urbisch D., Honarvar N., Kolle S. N., Mehling A., Ramirez T., Teubner W., Landsiedel R.2016. , Peptide reactivity associated with skin sensitization: The QSAR Toolbox and TIMES compared to the DPRA. Toxicol In Vitro **34**:194-203

[68] Urbisch D., Mehling A., Guth K., Ramirez T., Honarvar N., Kolle S., Landsiedel R., Jaworska J., Kern P. S., Gerberick F., Natsch A., Emter R., Ashikaga T., Miyazawa M., Sakaguchi H.2015. , Assessing skin sensitization hazard in mice and men using non-animal test methods. Regul Toxicol Pharmacol. **71**(2):337-51

[69] Van der Veen J. W., Pronk T. E., Van Loveren H., Ezendam J.2013. , Applicability of a keratinocyte gene signature to predict skin sensitizing potential. Toxicol In Vitro **27**(1):314–322

[70] Van der Veen J. W., Rorije E., Emter R., Natsch A., van Loveren H., Ezendam J.2014. , Evaluating the performance of integrated approaches for hazard identification of skin sensitizing chemicals. Regul Toxicol Pharmacol. **69**(3):371-9

[71] Van der Veen J. W., Vandebriel R., Van Loveren H., Ezendam J., 2011. *Keratinocytes, innate immunity and allergic contact dermatitis – opportunities for the development of in vitro assays to predict the sensitizing potential of chemicals.* DOI: 10.5772/28337. In: Ro YS, (ed) Contact dermatitis. DOI: 10.5772/1167. ISBN: 978-953-307-577-8

[72] Safford R.J.2008. , The dermal sensitisation threshold– a TTC approach for allergic contact dermatitis. Regul. Toxicol. Pharmacol. **51**:195–200

[73] Myers D.K., Goldberg A.M., Poth A., Wolf M.F., Carraway J., McKim J., Coleman K.P., Hutchinson R., Brown R., Krug H.F., Bahinski A., Hartung T.2017. , From In Vivo to In Vitro: The Medical Device Testing Paradigm Shift. ALTEX **34**(4):479-500

Bibliography for in vitro assays for sensitization testing (DPRA)

[74] Gerberick GF, Vassallo JD, Bailey RE, Chaney JG, Morrall SW, Lepoittevin JP2004. , Development of a peptide reactivity assay for screening contact allergens. Toxicol Sci. **81**(2):332-43

[75] Organization for Economic Cooperation and Development (OECD), Test No. 442C: In Chemico Skin Sensitisation: Assays addressing the Adverse Outcome Pathway key event on covalent binding to proteins, OECD Guidelines for the Testing of Chemicals, Section 4, Éditions OCDE, Paris, 2019, Available at: https://doi.org/10.1787/9789264229709-en

© ISO 2021 – All rights reserved

[76] Urbisch D., Mehling A., Guth K., Ramirez T., Honarvar N., Kolle S., Landsiedel R., Jaworska J., Kern P.S., Gerberick F., Natsch A., Emter R., Ashikaga T., Miyazawa M., Sakaguchi H.2015. , Assessing skin sensitization hazard in mice and men using non-animal test methods. Regul Toxicol Pharmacol. **71**(2):337-351

Bibliography for in vitro assays for sensitization testing (KeratinoSens™)

[77] EURL ECVAM, 2014. Recommendation on the KeratinoSens™ assay for skin sensitisation testing, 42 pp. Available at: https://ihcp.jrc.ec.europa.eu/our_labs/eurl-ecvam/eurl-ecvam-recommendations/recommendation-keratinosens-skin-sensitisation.

[78] Fabian E., Vogel D., Blatz V., Ramirez T., Kolle S., Eltze T., van Ravenzwaay B., Oesch F., Landsiedel R.2013. , Xenobiotic metabolizin enzyme activities in cells used for testing skin sensitization in vitro. Arch Toxicol **87**(9):1683-1969

[79] OECD, 2018. Test No. 442D: In Vitro Skin Sensitisation: ARE-Nrf2 Luciferase Test Method. OECD Guidelines for the Testing of Chemicals, Section 4. OECD Publishing, Paris. https://doi.org/10.1787/9789264229822-en

[80] Thorne N., Inglese J., Auld D.S.2010. , Illuminating Insights into Firefly Luciferase and Other Bioluminescent Reporters Used in Chemical Biology. Chemistry and Biology **17**(6):646-657

Bibliography for in vitro assays for sensitization testing (LuSens)

[81] EURL ECVAM, 2018. The LuSens test method Validation Study Report. Accessible at: https://tsar.jrc.ec.europa.eu/test-method/tm2011-10

[82] OECD, 2018. Test No. 442D: In Vitro Skin Sensitisation: ARE-Nrf2 Luciferase Test Method. OECD Guidelines for the Testing of Chemicals, Section 4. OECD Publishing, Paris. https://doi.org/10.1787/9789264229822-en

[83] Ramirez T., Mehling A., Kolle S.N., Wruck C.J., Teubner W., Eltze T., Aumann A., Urbisch D., van Ravenzwaay B., Landsiedel R.2014. , LuSens: a keratinocyte based ARE reporter gene assay for use in integrated testing strategies for skin sensitization hazard identification. Toxicol. In Vitro **28**, 1482-1497

[84] Ramirez T., Stein N., Aumann A., Remus T., Edwards A., Norman K.G., Ryan C., Bader J.E., Fehr M., Burleson F., Foertsch L., Wang X., Gerberick F., Beilstein P., Hoffmann S., Mehling A., van Ravenzwaay B., Landsiedel R.2016. , Intra- and inter-laboratory reproducibility and accuracy of the LuSens assay: A reporter gene-cell line to detect keratinocyte activation by skin sensitizers. Toxicol. In Vitro **32**, 278-286

Bibliography for in vitro assays for sensitization testing (SENS-IS)

[85] Cottrez F, Boitel E, Auriault C, Aeby P, Groux H2015. , Genes specifically modulated in sensitized skins allow the detection of sensitizers in a reconstructed human skin model. Development of the SENS-IS assay. Toxicol In Vitro **29** (4):787-802

[86] Cottrez F., Boitel E., Pellevoisin C., Alonso A., Seyler S., Groux H., 2016a. Evaluation of the SkinEthic RHE model in the SENS-IS assay for prediction of skin sensitization of chemicals. Society of Toxicology Annual Meeting, New Orleans, Louisiana, USA

[87] Cottrez F, Boitel E, Ourlin JC, Peiffer JL, Fabre I, Henaoui IS, Mari B, Vallauri A, Paquet A, Barbry P, Auriault C, Aeby P, Groux H2016b. , SENS-IS, a 3D reconstituted epidermis based model for quantifying chemical sensitization potency: Reproducibility and predictivity results from an inter-laboratory study. Toxicol In Vitro **32**:248-60

[88] Cottrez F., Pellevoisin C., Coleman K., Groux H., 2018. In vitro assessment of medical device extracts potential to produce skin sensitization. Toxicology Letters Volume 295, Supplement 1, 10 October, Page S179, https://doi.org/10.1016/j.toxlet.2018.06.827

© ISO 2021 – All rights reserved

[89] Uruno A., Motohashi H.2011. , The Keap1-Nrf2 system as an in vivo sensor for electrophiles. Nitric Oxide **25**(2):153-60

[90] Cottrez F., Boitel E., Berrada-Gomez M.P., Dalhuchyts H., Bidan C., Rattier S., Ferret P.J., Groux H.2020. , In Vitro Measurement of Skin Sensitization Hazard of Mixtures and Finished Products: Results Obtained With the SENS-IS Assays. Toxicol In Vitro **62**:104644

[91] Bergal M., Puginier M., Gerbeix C., Groux H., Roso A., Cottrez F., Milius A.2020. , In vitro testing strategy for assessing the skin sensitizing potential of “difficult to test” cosmetic ingredients. Toxicol In Vitro **65**:104781

Bibliography for in vitro assays for sensitization testing (IL-18 RhE Assay)

[92] Andres E., Barry M., Hundt A., Dini C., Corsini E., Gibbs S., Roggen E. L., Ferret P. J.2017. , Preliminary performance data of the RHE/IL-18 assay performed on SkinEthic™ RHE for the identification of contact sensitizers. Int J Cosmet Sci. **39**(2):121-132

[93] Galbiati V., Papale A., Marinovich M., Gibbs S., Roggen E.L., Corsini E.2017. , Development of an in vitro method to estimate the sensitization induction level of contact allergens. Toxicology Letters **271**:1-11

[94] Gibbs S., Kosten I., Veldhuizen R., Spiekstra S., Corsini E., Roggen E., Rustemeyer T., Feilzer A. J., Kleverlaan C. J.2018. , Assessment of metal sensitizer potency with the reconstructed human epidermis IL-18 assay. Toxicology **393**:62-72

[95] Gibbs S., Corsini E., Spiekstra S.W., Galbiati V., Fuchs H.W., Degeorge G., Troese M.J., Hayden P.M., Deng W., Roggen E.2013. , An epidermal equivalent assay for identification and ranking potency of contact sensitizers. Toxicology and Applied Pharmacology **272**(2):529-41

Bibliography for in vitro assays for sensitization testing (EpiSensA)

[96] Saito K, Nukada Y, Takenouchi O, Miyazawa M, Sakaguchi H, Nishiyama N2013. , Development of a new in vitro skin sensitization assay (Epidermal Sensitization Assay; EpiSensA) using reconstructed human epidermis. Toxicol In Vitro **27**(8):2213-24

[97] Saito K, Takenouchi O, Nukada Y, Miyazawa M, Sakaguchi H2017. , An in vitro skin sensitization assay termed EpiSensA for broad sets of chemicals including lipophilic chemicals and pre/pro-haptens. Toxicol In Vitro **40**:11-25

Bibliography for in vitro assays for sensitization testing (SenCeeTox®)

[98] Coleman K.P., McNamara L.R., Grailer T.P., Willoughby J.A.Sr, Keller D.J., Patel P., Thomas S., Dilworth C.2015. , Evaluation of an In Vitro Human Dermal Sensitization Test for Use with Medical Device Extracts. Applied In Vitro Toxicology **1**(2): 118-130

[99] McKim J.M.Jr, Keller D.J.III, Gorski J.R.2010. , A new in vitro method for identifying chemical sensitizers combining peptide binding with ARE/EpRE-mediated gene expression in human skin cells. Cutaneous and Ocular Toxicology **29**(3):171-192

[100] McKim J.M.Jr, Keller D.J.III, Gorski J.R.2012. , An in vitro method for detecting chemical sensitization using human reconstructed skin models and its applicability to cosmetic, pharmaceutical, and medical device safety testing. Cutaneous and Ocular Toxicology **31**(4):292-305

Bibliography for in vitro assays for sensitization testing (h-CLAT)

[101] Ashikaga T., Yoshida Y., Hirota M., Yoneyama K., Itagaki H., Sakaguchi H., Miyazawa M., Ito Y., Suzuki H., Toyoda H.2006. , Development of an in vitro skin sensitization test using human cell lines: The human Cell Line Activation Test (h-CLAT) I. Optimization of the h-CLAT protocol. Toxicol. In Vitro **20**:767–773

© ISO 2021 – All rights reserved

[102] EC EURL ECVAM, 2015. Re-analysis of the within and between laboratory reproducibility of the human Cell Line Activation Test (h-CLAT). Accessible at: https://eurl-ecvam.jrc.ec.europa.eu/eurl-ecvam-recommendations/eurl-ecvam-recommendation-on-the-human-cell-line-activation-test-h-clat-for-skin-sensitisation-testing

[103] FABIAN E., VOGEL D., BLATZ V., RAMIREZ T., KOLLE S., ELTZE T., VAN RAVENZWAAY B., OESCH F., LANDSIEDEL R.2013. , Xenobiotic metabolizing enzyme activities in cells used for testing skin sensitization in vitro. Arch Toxicol **87**:1683-1969

[104] OECD, 2018. Test No. 442E: In Vitro Skin Sensitisation: In Vitro Skin Sensitisation assays addressing the Key Event on activation of dendritic cells on the Adverse Outcome Pathway for Skin Sensitisation, OECD Guidelines for the Testing of Chemicals, Section 4, OECD Publishing, Paris, https://doi.org/10.1787/9789264264359-en

[105] TAKENOUCHI O., MIYAZAWA M., SAITO K., ASHIKAGA T., SAKAGUCHI H.2013. , Predictive performance of the human Cell Line Activation Test (h-CLAT) for lipophilic with high octanol-water partition coefficients. J. Toxicol. Sci. **38**:599-609

Bibliography for in vitro assays for sensitization testing (U-SENS™)

[106] ALÉPÉE N., PIROIRD C., AUJOULAT M., DREYFUSS S., HOFFMANN S., HOHENSTEIN A., MELONI M., NARDELLI L., GERBEIX C., COTOVIO J.2015. , Prospective multicentre study of the U-SENS test method for skin sensitization testing. Toxicol In Vitro **30**:373-382

[107] EURL ECVAM, 2017). The U-SENS™ test method Validation Study Report. Accessible at: https://ihcp.jrc.ec.europa.eu/our_labs/eurl-ecvam/eurl-ecvam-recommendations

[108] OECD, 2018. Test No. 442E: In Vitro Skin Sensitisation: In Vitro Skin Sensitisation assays addressing the Key Event on activation of dendritic cells on the Adverse Outcome Pathway for Skin Sensitisation, OECD Guidelines for the Testing of Chemicals, Section 4, OECD Publishing, Paris, https://doi.org/10.1787/9789264264359-en

[109] PIROIRD C., OVIGNE J.M., ROUSSET F., MARTINOZZI-TEISSIER S., GOMES C., COTOVIO J., ALÉPÉE N.2015. , The Myeloid U937 Skin Sensitization Test (U-SENS) addresses the activation of dendritic cell event in the adverse outcome pathway for skin sensitization. Toxicol. In Vitro **29**:901-916

Bibliography for in vitro assays for sensitization testing (IL-8 Luc assay)

[110] KIMURA Y., FUJIMURA C., ITO Y., TAKAHASHI T., NAKAJIMA Y., OHMIYA Y., AIBA S.2015. , Optimization of the IL-8 Luc assay as an in vitro test for skin sensitization, Toxicol In Vitro **29**:1816-30

[111] OECD, 2018. Test No. 442E: In Vitro Skin Sensitisation: In Vitro Skin Sensitisation assays addressing the Key Event on activation of dendritic cells on the Adverse Outcome Pathway for Skin Sensitisation, OECD Guidelines for the Testing of Chemicals, Section 4, OECD Publishing, Paris, https://doi.org/10.1787/9789264264359-en

[112] OECD, 2017. Report of the Peer Review Panel for the IL-8 Luciferase (IL-8 Luc) Assay for in vitro skin sensitisation. Series on Testing and Assessment No. 258, ENV/JM/MONO(2017)20. Organisation for Economic Cooperation and Development, Paris. Available at: http://www.oecd.org/env/ehs/testing/series-testing-assessment-publications-number.htm

Bibliography for in vitro assays for sensitization testing (GARD™)

[113] FORRERYD A., JOHANSSON H., ALBREKT A. S., LINDSTEDT M.2014. , Evaluation of high throughput gene expression platforms using a genomic biomarker signature for prediction of skin sensitization. BMC Genomics **15**:379

[114] FORRERYD A., ZELLER K. S., LINDBERG T., JOHANSSON H., LINDSTEDT M.2016. , From genome-wide arrays to tailor-made biomarker readout – Progress towards routine analysis of skin sensitizing chemicals with GARD. Toxicol In Vitro **37**:178–188

© ISO 2021 – All rights reserved

[115] JOHANSSON H., ALBREKT A. S., BORREBAECK C. A., LINDSTEDT M.2013. , The GARD assay for assessment of chemical skin sensitizers. Toxicol In Vitro **27**(3):1163-9

[116] JOHANSSON H, LINDSTEDT M, ALBREKT AS, BORREBAECK CA2011) , A genomic biomarker signature can predict skin sensitizers using a cell-based in vitro alternative to animal tests. BMC Genomics. **12**:399.

[117] ROBERTS D. W.2018. , Is a combination of assays really needed for non-animal prediction of skin sensitization potential? Performance of the GARD™ (Genomic Allergen Rapid Detection) assay in comparison with OECD guideline assays alone and in combination. Reg. Toxocol. Pharmacol. **98**:155-160

[118] JOHANSSON H., GRADIN R., JOHANSSON A., ADRIAENS E., EDWARDS A., ZUCKERSTÄTTER V., JERRE A., BURLESON F., GEHRKE H., ROGGEN E.2019. , Validation of the GARD™skin Assay for Assessment of Chemical Skin Sensitizers: Ring Trial Results of Predictive Performance and Reproducibility. Toxicological Sciences, **170** (2): 374–381

[119] JENVERT RM., JOHANSSON A, LARNE O., AALTONEN E., JERRE A., GRADIN R., JOHANSSON H. 2019. *In vitro* skin sensitization testing of Medical Devices using GARD®. Toxicology Letters, volume 31, Supplement 1, 10 October, 155.

[120] ISO 10993-5**,** *Biological evaluation of medical devices — Part 5: Tests for in vitro cytotoxicity*

[121] OECD, 2020. OECD Test Guidelines Programme, Organisation for Economic Cooperation and Development, Paris. Available at: http://www.oecd.org/chemicalsafety/testing/oecd-guidelines-testing-chemicals-related-documents.htm

[122] OCDE. 2010, Test No. 442A: Skin Sensitization : Local Lymph Node Assay: DA, OECD Guidelines for the Testing of Chemicals, Section 4, Éditions OCDE, Paris, https://doi.org/10.1787/9789264090972-en

[123] OCDE. 2018, Test No. 442B: Skin Sensitization : Local Lymph Node Assay: BrdU-ELISA or –FCM, OECD Guidelines for the Testing of Chemicals, Section 4, Éditions OCDE, Paris, https://doi.org/10.1787/9789264090996-en

[124] OCDE. 2017, Guidance Document on the Reporting of Defined Approaches and Individual Information Sources to be Used within Integrated Approaches to Testing and Assessment (IATA) for Skin Sensitisation, OECD Series on Testing and Assessment, n° 256, Éditions OCDE, Paris, https://doi.org/10.1787/9789264279285-en

[125] ISO/IEC 17025, *General requirements for the competence of testing and calibration laboratories*

© ISO 2021 – All rights reserved

ICS 11.100.20

Price based on 48 pages

© ISO 2021 – All rights reserved
